# In Silico Identification and Analysis of Potentially Bioactive Antiviral Phytochemicals against SARS-CoV-2: A Molecular Docking and Dynamics Simulation Approach

**DOI:** 10.1155/2023/5469258

**Published:** 2023-05-11

**Authors:** Sajal Kumar Halder, Ive Sultana, Md Nazmussakib Shuvo, Aparna Shil, Mahbubul Kabir Himel, Md. Ashraful Hasan, Mohammad Mahfuz Ali Khan Shawan

**Affiliations:** ^1^Department of Biochemistry and Molecular Biology, Jahangirnagar University, Savar, Dhaka 1342, Bangladesh; ^2^Department of Microbiology, Jahangirnagar University, Savar, Dhaka 1342, Bangladesh; ^3^Department of Botany, Jahangirnagar University, Savar, Dhaka 1342, Bangladesh

## Abstract

SARS-CoV-2, a deadly coronavirus sparked COVID-19 pandemic around the globe. With an increased mutation rate, this infectious agent is highly transmissible inducing an escalated rate of infections and death everywhere. Hence, the discovery of a viable antiviral therapy option is urgent. Computational approaches have offered a revolutionary framework to identify novel antimicrobial treatment regimens and allow a quicker, cost-effective, and productive conversion into the health center by evaluating preliminary and safety investigations. The primary purpose of this research was to find plausible plant-derived antiviral small molecules to halt the viral entrance into individuals by clogging the adherence of Spike protein with human ACE2 receptor and to suppress their genome replication by obstructing the activity of Nsp3 (Nonstructural protein 3) and 3CLpro (main protease). An in-house library of 1163 phytochemicals were selected from the NPASS and PubChem databases for downstream analysis. Preliminary analysis with SwissADME and pkCSM revealed 149 finest small molecules from the large dataset. Virtual screening using the molecular docking scoring and the MM-GBSA data analysis revealed that three candidate ligands CHEMBL503 (Lovastatin), CHEMBL490355 (Sulfuretin), and CHEMBL4216332 (Grayanoside A) successfully formed docked complex within the active site of human ACE2 receptor, Nsp3, and 3CLpro, respectively. Dual method molecular dynamics (MD) simulation and post-MD MM-GBSA further confirmed efficient binding and stable interaction between the ligands and target proteins. Furthermore, biological activity spectra and molecular target analysis revealed that all three preselected phytochemicals were biologically active and safe for human use. Throughout the adopted methodology, all three therapeutic candidates significantly outperformed the control drugs (Molnupiravir and Paxlovid). Finally, our research implies that these SARS-CoV-2 protein antagonists might be viable therapeutic options. At the same time, enough wet lab evaluations would be needed to ensure the therapeutic potency of the recommended drug candidates for SARS-CoV-2.

## 1. Introduction

The World Health Organization (WHO) reported that the prevalence of SARS-CoV-2 is spreading at an alarming rate, posing severe health problems. The most recent outburst of second wave of SARS-CoV-2 has turned into a worldwide catastrophe. Following the coronavirus (CoV) epidemic in China in December 2019, WHO classified SARS-CoV-2, as the newest candidate of the *Coronaviridae* family within Nidovirales order [1]. As of 3rd May 2023, WHO has received a report from around 765,222,932 diagnosed COVID-19 infections worldwide, with 6,921,614 fatalities [2, 3]. According to available information, the virus can be transmitted often by close, indirect, or direct exposure to infectious persons, as well as contaminated secretions such as nasal droplets and saliva, and respiratory secretions released when an infected individual sneezes, coughs, or speaks [3]. It has been linked to a wide range of signs and symptoms, consisting of minor to severe illness, which varies from patient to patient. Complications might appear anywhere from two to fourteen days after the virus has been infected. Fever, fatigue, chronic cough, sore throat, difficulty breathing, impairment of taste/odor, nausea, sputum production, headache, expectoration, diarrhea, anorexia, and some other symptoms might occur at separate phases of the disease [1, 4].

SARS-CoV-2 is a membrane-encased positive-sense single-stranded RNA ((+) ssRNA) virus having a diameter ranging from 60 to 140 nanometers [4, 5]. The envelope is surrounded by spike-shaped glycoprotein protrusions that resemble crowns under the electron microscope [6]. The spike (S), nucleocapsid (N), envelope (E), and membrane (M) proteins are among the four crucial targets encoded by the SARS-CoV-2 genome. Main protease (3CLpro), RNA-dependent RNA polymerase (RdRp), and papain-like protease (PLpro) are some of the nonstructural proteins synthesized by the viral DNA [7]. Nonstructural protein 3 (Nsp3) proteins containing macrodomains are pervasive and evolutionarily conserved and responsible for the transcription process [8]. Previous study has established that human angiotensin converting enzyme 2 (ACE2) receptor has a greater affinity for the RBD region of the spike protein [9]. The attachment of favorable ligands to the active pockets of human ACE2 receptor might alter the protein's structure. As a result, the viral ACE2 entrance region might be a feasible object for therapeutic advancement. Since the main protease of SARS-CoV-2 is vital for its growth and the consequent expression of the replicase polyproteins, it has turned into an obvious target for anti-COVID-19 therapeutic design [10]. As a result, focusing on these proteins might help with long-term COVID-19 infection management and eradication.

The viral disease is spreading at a surprising pace worldwide, and researchers are racing to develop effective drugs to use as therapeutic agents. The most promising choices appear to be natural compounds with substantial bioavailability and minimal cytotoxicity [1]. Clinically approved antiviral drugs are effective; however, some people become resistant to drugs. In contrast, it has been claimed that phytochemicals have more acceptable side effects and can be a satisfactory substitute for synthetic antiviral compounds for the suppression of viral life-cycle and penetration [10].

Humans have always relied on natural compounds, especially phytochemicals, to treat health problems since the dawn of time. Recently, Shawan et al. presented luteolin and abyssinone II as possible phytochemicals against SARS-CoV-2 [1]. Besides, Manojkumar et al. reported ervoside had anticoronavirus properties [11]. Similarly, Emran et al. identified phytochemicals medicagol, faradiol, and flavanthrin as the potential barrier of SARS-CoV-2 [12]. Computer-assisted drug development (CAD) entails the usage of computerized techniques to discover, design, and evaluate therapeutics and associated pharmacologically active substances [13]. CAD techniques have improved compound screening significantly over time, aimed at targeting structure prediction and model development, active site determination, comprehending the protein-ligand complex, testing a huge dataset of substances by estimating their pharmacokinetics characteristics, and analyzing the dynamics of proteins binding with ligands within biological settings [14]. Existing medicines like Molnupiravir and Paxlovid have been authorized by the FDA for utilization in emergencies; the treatment may be used either alone or combined with others [15]. For COVID-19 patients, the antiviral drug Molnupiravir has been recommended as a therapeutic for SARS-CoV-2 because it increases the likelihood of viral RNA alterations while also inhibiting viral replication [16]. Through inhibition of proteasome breakdown of viral proteins, Paxlovid inhibits protein production (RNA-dependent RNA polymerase, helicase, exoribonuclease, RNA-binding protein endoribonuclease). Consequently, the viral transcription and replication are halted [7].

The main focus of this in silico work was to utilize computational tools, i.e., molecular docking and MD simulation to examine the effective binding interactivity and affinities of repurposed antiviral phytochemicals with the human ACE2 receptor, Nsp3 macrodomain, and the main protease of the SARS-CoV-2 virus and identify the finest ligand hit [17]. Among all other crucial characteristics, absorption, distribution, metabolism, toxicity, and excretion (ADMET) were evaluated, and the best of them were selected. Finally, the most effective phytochemicals with higher binding energy to the target receptor and stronger stabilizing capacity were confirmed by employing molecular dynamics simulation.

## 2. Materials and Methods

**Figure 1:**
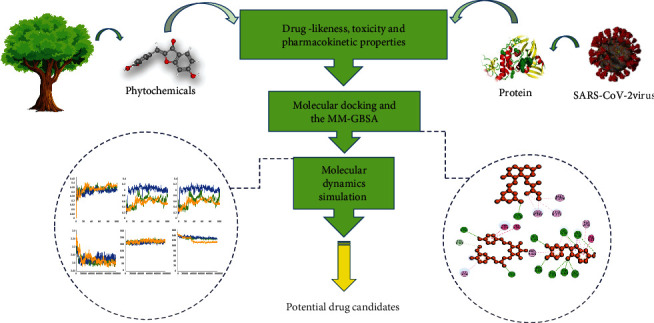
Complete work flow of the structure-based virtual screening study.

Virtual screening of natural bioactive molecules has become the standard method in the present therapeutic development workflow [18]. In this study, a wide range of repurposed phytochemicals were used from the NPASS (http://bidd.group/NPASS/) and PubChem (https://pubchem.ncbi.nlm.nih.gov/) servers as prospective ligands for SARS-CoV-2. The recently approved COVID-19 antiviral drugs Molnupiravir and Paxlovid were used as control drugs [19]. The workflow of our work was provided in Figure 1.

### 2.1. Characterization of Drug-Likeness Properties

A drug-like molecule can be considered a drug candidate by assessing its drug-like properties. The canonical SMILE sequence of the 1163 small molecules was fetched from the PubChem drug web server. The free accessible SwissADME was employed to compute the major physicochemical descriptors, pharmacokinetic properties, drug-like parameters, and associated factors [20]. To analyze the results, this application employs five principles of Lipinski's rule [21], Ghose's rule [22], Veber's rule [23], Egan rule [24], Muegge's rule [24], the number of rotatable bonds, and TPSA.

### 2.2. Characterization of ADMET Properties

pkCSM is an online tool that employs graph-based structural signatures for determining and improving pharmacokinetic characteristics and toxicity in small molecules. To devise an ADMET prediction benchmark for in silico drug discovery, pkCSM applies a cut-off scanning strategy [25]. The chosen criteria for the prediction model were hepatotoxicity, Ames toxicity, oral rat acute toxicity, human intestinal absorption (HI), hERG I inhibitor, hERG II inhibitor, P-glycoprotein I inhibitor, P-glycoprotein II inhibitor, P-glycoprotein substrate, BBB permeability (log BB), Caco-2 permeability, CYP2D6 substrate, CYP3A4 substrate, CYP2C19 inhibitor, CYP1A2 inhibitor, CYP3A4 inhibitor, CYP2C9 inhibitor, and CYP2D6 inhibitor.

### 2.3. Molecular Docking by AutoDock vina

#### 2.3.1. Ligand Preparation

At pH 7.4, polar hydrogen atoms were introduced to the downloaded 3D molecular ligands in SDF (spatial data file) format using the build module of the Avogadro 1.2.0. The same program was then used to conduct geometry optimization and energy reduction employing the MMFF94 force field and steepest descent option. These structures were retained in the PDB [26] extension for additional investigation. To add polar hydrogens and fix torsions of the ligands, AutoDockT tools-MGLTool 1.5.6 was used [27].

#### 2.3.2. Protein Preparation

The preferred structures of SARS-CoV-2 main protease in complex with FSCU015 (PDB ID: 7NT3), Nsp3 macrodomain in complex with ADP-ribose (PDB ID: 7KQP), and inhibitor bound human ACE2-related carboxypeptidase (PDB ID: 1R4L) were taken from RCSB repository (https://www.rcsb.org/). Initially, the 3D structures were prepared in the PyMOL program [1]. Swiss-PdbViewer was subsequently used to minimize the energy of the selected proteins [28]. Next, the energy-minimized structures were loaded into AutoDock-MGLTools 1.5.6 to incorporate polar hydrogen and convert the PDB to PDBQT format.

#### 2.3.3. Active Site Detection and Grid Box Preparation

Finding a ligand-binding region on a protein is the basic strategy for the molecular docking technique [29]. The possibility of protein-ligand attachment relies on numerous factors such as hydrogen bonds, hydrophobic or hydrophilic interactions, electrostatic and salt bridges. CASTp 3.0 website (http://sts.bioe.uic.edu/castp/) was employed to detect the active region of target proteins [30]. It applies an alpha shape detection approach to determine topographic properties and estimate protein area and volume for identifying ligand-binding cavities.

#### 2.3.4. Binding Affinity Prediction by AutoDock vina

Virtual screening via docking studies is extensively used in computer-led pharmaceutical research to uncover promising drug-like substances. Initially, AutoDock vina was exploited to conduct rigid molecular docking among the proteins and selected compounds (ligands and control drugs), including a search area of 27,000 m^3^ and exhaustiveness 10, and ligands being flexible while receptors remained rigid [18]. AutoDock vina calculates the binding energy and fixes the binding poses using the Lamarckian genetic algorithm. Here, in this study, 149 small molecules were docked with three target proteins (coordinates of geometry-optimized ligands of the best hits provided in Supplementary Table 5).

### 2.4. Glide Docking and MM-GBSA Analyses

Schrodinger was employed to perform glide docking and MM-GBSA analyses (Maestro 12.5, Schrodinger Suites 2020-3). Previously screened ligands having higher affinity for target proteins than the reference drugs were explored in this step.

#### 2.4.1. Preparation of Ligand Structures

The LigPrep module yields top hits of 3D configurations for small molecules, beginning from 1-dimensional/2-dimensional/3-dimensional structures in Maestro, Mol2, SMILES, or SD format [31]. By introducing hydrogens, ionizing at pH (7 ± 2.0), and subtracting salts, the LigPrep tool builds molecules and constructs 3D structures of them. Following that energy minimized and geometrically refined ligands were prepared by employing a built-in OPLS3e force field in Schrödinger Maestro 12.5 [32].

#### 2.4.2. Preparation of Protein Structures

The protein structures (main protease, Nsp3, human ACE2 receptor) were loaded straight into the protein preparation wizard [32]. Protein structures were preprocessed by setting up bond orders, adding hydrogens and cap termini, and filling the missing atoms by prime module. At pH 7.0, the PROPKA application was used to calculate the protonation phases. Following that, the water portion around the protein was eliminated above 3.0 Å, and restrained minimization was executed utilizing the OPLS3e force field.

#### 2.4.3. Preparation of Receptor Grid Box

The grid region directs small molecules to the binding center of the protein, making it an important part of molecular docking research. The grid model was created with the standard options of a Van der Waals radius scaling marker of 1.0 and a charge threshold score of 0.25 in the receptor grid generation package. The attached ligands UQZ, AR6, and XX5 within the protein structures main protease, Nsp3, and human ACE2 receptor, respectively, were used to define the region for the grid map.

#### 2.4.4. Glide Docking and MM-GBSA Studies

Glide is a combined molecular docking technology that can facilitate both ligand and receptor flexibility [33]. Glide XP was developed to retrieve the finest docking poses having the greatest-scoring compounds. For drug molecules, a minimum scoring of 0.15 and a Van der Waals radius scaling marker of 0.80 was applied.

(1) $$\begin{array}{c} \text{Docking score}=a\times \text{VdW}+b\times \text{Coul}+\text{Hbond}+\text{Metal}+\text{Lipo}+\text{BuryP}+\text{RotB}+\text{Site}. \end{array}$$

Here, *a* and *b* are coefficient constants for VdW and Coul, respectively, VdW is the Van der Waals energy, Coul is the Coulomb energy, H-bond is the hydrogen bonding with the receptor, Metal is the binding with metal, Lipo is the constant term for lipophilic, BuryP is the buried polar group penalty, RotB is the rotatable bond penalty, and Site is the active site polar interaction [1].

The binding free energy among the receptor and the collection of small molecules was measured using the prime MM-GBSA module. The binding energy of the ligand-protein constructs was estimated utilizing the OPLS3e force field, and the docked conformations were minimized utilizing Prime's native optimization tool.

(2) $$\begin{array}{c} \Delta \text{Gbind }\left(\text{Binding Free Energy}\right)=\Delta \text{EMM}+\Delta \text{Gsolv}+\Delta \text{GSA}. \end{array}$$

Here, *Δ*EMM represents lowered energy deviations among the totality of the energies of the protein and ligand and protein-ligand complex. *Δ*Gsolv displays the divergence in the GBSA solvation energy of the complex structure and the aggregate of the salvation energies for the ligand and protein. *Δ*GSA describes the deviation in the energies for the surface area of a complex and the total surface area of the ligand and protein complex [34].

### 2.5. Molecular Dynamics Simulation by GROMACS

The molecular dynamics program simulates the movements of a protein molecule utilizing the interaction potential to compute interatomic energies and equations of motion that regulate the machinery's dynamics in the drug design study. It illustrates the stability and flexibility data of ligand binding to a flexible target protein. GROMACS (https://simlab.uams.edu/) service was exploited to simulate the protein-ligand conformations, and the GROMOS96 43a1 force field was employed to produce the topological data of the complex constructs [35]. The PRODRG (http://davapc1.bioch.dundee.ac.uk/cgi-bin/prodrg) Server was employed to render small molecule topology and coordinate information [36]. The aqueous phase of macromolecules was produced sequentially using the SPC water model (simple point-charge) and subsequently neutralized using 0.15 M NaCl solution [37]. A triclinic box was used to contain the bimolecular environment, and 5000 iterations of steepest descent strategies were used to minimize energy. The equilibrium of ion molecules around the macromolecule was accomplished at 310 K and 1.0 bar utilizing NPT (constant pressure) and NVT (constant volume) setups. After 100 nanoseconds of simulation, it provided trajectories of simulated structures, including the root-mean-square deviation (RMSD), the root-mean-square fluctuation (RMSF), the solvent-accessible surface area (SASA), hydrogen bonds (HBs), and the radius of gyration (Rg) [38].

### 2.6. Molecular Dynamics Simulation and Post MM-GBSA Evaluation by Desmond

The molecular dynamics simulation provides evidence regarding the mobility and stability of the bound protein-ligand complex. On Desmond software, the MD simulation and post-MMGBSA analysis of the main-protease_ligand, Nsp3_ligand, and human ACE2 receptor_ligand complexes were performed [39]. These compounds were solvated on a cubic TIP3P water model using the system builder package. A minimal spacing of 10 was maintained between the protein and the solvated region. Subsequently, Na+ salts were supplied until the final system strength reached 0.15 M, which is the physiological salt concentration present in the human body. The integrated OPLS3e force field was used to optimize the final system's energy. To complete the MDS, we used the isothermal isobaric ensemble (NPT) at 1.013 bar and 310° K. The total duration of the simulation run was 100 nanoseconds (ns). It was paired with a recording duration of 100 picoseconds (ps), during which 1000 frames were incorporated into the trajectory. Next, we studied the trajectories in the simulation interaction diagram (SID) program, and the reported results comprised RMSD, RMSF, protein-ligand contact outline, and biophysical properties of ligands. After running the simulations, MM-GBSA was evaluated employing the thermal MM-GBSA.py program. During the assessment, a 0-1000 periodic frame was incorporated for the analysis [40].

### 2.7. Prediction of Molecular Target with SwissTargetPrediction Server

The anticipation of a molecular target for a small-molecule is vital for drug research and development. These studies are essential for assessing the potential for adverse reactions or cross-reactivity in *Homo sapiens* caused by the action of that bioactive small molecule. We employed SwissTargetPredcition (http://www.swisstargetprediction.ch/) to determine the human body receptors for small compounds that had previously been identified by molecular docking and shown stability via MD simulation investigations [41]. The canonical smiles of the small compounds were used in the server and analyzed.

### 2.8. Prediction of Biological Activity by PASS-Way2Drug Tool

The PASS-Way2Drug webserver (http://www.pharmaexpert.ru/passonline/) was employed to the prediction of biological activity scales for Lovastatin, Sulfuretin, and Grayanoside A [42]. For PASS recommendations to be reliable, the Pa (likelihood to be effective) threshold should be set at 70% or above. This is because surpassing the Pa>70% threshold yields very reliable predictions [42]. Calculated ligand activity was based on Pi and Pa scores.

## 3. Results

### 3.1. Analysis of Drug-Like Properties

In this experiment, 1163 drug-like substances were checked for their drug-like activities. All of them have been filtered using five principles of Lipinski's filtration technique, which included molecular mass (recommended value: <500), the number of hydrogen bond donors (ideal value: ≤5), the number of hydrogen bond acceptors (standard range: ≤10), lipophilicity (represented as LogP, normal value: <5), and molar refractivity (preferable range: 40–130). Additionally, the ligands were screened based on the criteria of Ghose, Veber, Egan, and Muegge's rule. Subsequently, 497 out of 1163 compounds were shortlisted for the following evaluation (Supplementary table1).  Table 1 represented the drug-like properties of the best-hit phytochemicals and control drugs.

### 3.2. Analysis of ADMET Properties

A total of 149 drug-like substances were qualified after this analysis. Moreover, from estimating distribution levels, all the compounds are impermeable to the blood-brain barrier. Metabolic inability could cause lower bioavailability and excretion, high toxicity, and drug-drug interactions. These 149 small substances function as isoforms of the CYP 2D6 and 3A4 enzymes. Diverse computational algorithms are used to evaluate toxicity: hERG inhibitors, AMES toxicity, maximum tolerated dosage Hepatotoxicity. Ligands with a negative value in these models were chosen for the following step. ADMET properties of the best-hit phytochemicals and control drugs were presented in Table 2. Finally, we filtered out 149 drug-like substances from this analysis (Supplementary table2).

### 3.3. Analysis of Molecular Docking Results by AutoDock vina

In structure-based pharmaceutical research, molecular docking is a commonly used strategy to identify the finest ligand hits against a particular protein. The docking method predicts the ligand orientation, location, conformation in the protein's active site, binding interaction, and affinity. AutoDock vina determines the binding energy and poses of trial ligands by employing a grid-based technique. Previously selected small molecules were docked with three SARS-CoV-2 target proteins. Out of the 149 small compounds, 97 small molecules exhibited a higher binding affinity for the main protease (Supplementary Table 3a), 75 small molecules for the Nsp3 (Supplementary Table 3b), and 106 small molecules for human ACE2 receptor compared to the control therapeutics (Supplementary Table 3c).

Lovastatin's binding energy for the main protease was -7.2 kcal/mol, which was considerably higher than that of the control ligands, Molnupiravir (-6.4 kcal/mol), and Paxlovid (-6.6 kcal/mol) (Table 3). Lovastatin produced a robust hydrogen interaction with the amino acids ARG131 (2.30102 Å) of the main protease, whereas Molnupiravir and Paxlovid formed three and six hydrogen bonds with the target protein, respectively, with THR26, HIS41, ASN119, ASN142, GLY143 (1.98365 Å), and CYS145 residues (AutoDock vina). Sulfuretin had binding energy of -8.8 kcal/mol for Nsp3 compared to the control ligands molnupiravir and Paxlovid, which had binding energies of -7.7 and -7.5 kcal/mol, respectively (Table 4). Sulfuretin formed seven strong hydrogen bonds with the Nsp3 protein (VAL49, LEU126, SER128, ALA129, GLY130, PHE156, and ALA38), whereas Molnupiravir and Paxlovid created six (ASN40, GLY47, VAL49, ALA50, LYS44, and ALA38 (1.90623 Å)) and three (LYS158, LEU160, and TYR161 (1.23877 Å)) amino acid residues. Sulfuretin also created seven hydrophobic bonds (ALA38, PHE132, VAL49, ALA38, ALA50, VAL49, and PRO125) with the same protein. For human ACE2 receptor, Grayanoside A showed a binding affinity of 7.8 kcal/mol compared to the control molecules Molnupiravir (-7.6 kcal/mol) and Paxlovid (-7.0 kcal/mol) (Table 5). Molnupiravir and Paxlovid formed five (ASP206, HIS378, ASN394, ARG514, and LYS562 (2.198 Å)) and six (ASP206, ALA348, TRP349 (1.978 Å), ASP350, HIS378, and ARG514) hydrogen bonds with the target protein, human ACE2 receptor, respectively. Grayanoside A formed three strong hydrogen bonds (ARG273, ARG273, and GLU406) and six hydrophobic bonds (VAL209, LYS562, TRP566, LEU95, LYS562, and ALA99) with the human ACE2 receptor.

### 3.4. Analysis of Glide and MM-GBSA Scores

**Figure 2:**
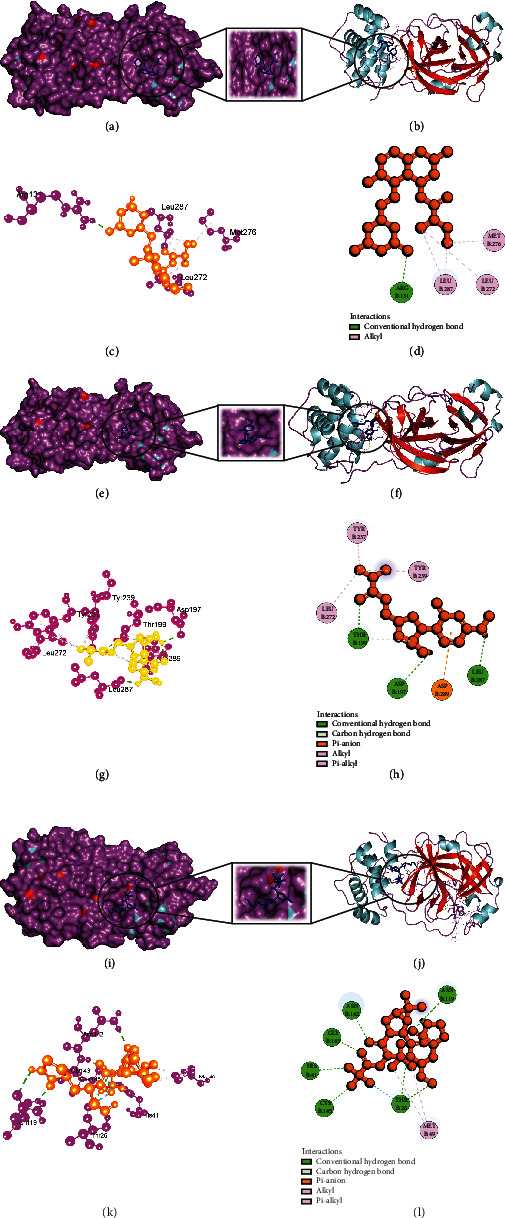
Schematic illustration of 7NT3_CHEMBL503 (Lovastatin), 7NT3_Molnupiravir, and 7NT3_Paxlovid complexes. (a, b) Share the pose and surface view of protein and ligand complex. Here, protein is in purple and cyan colors and ligand is in blue color. (c, d) Share 3D and 2D interactions of protein and ligand complex. Magenta color represents proteins, and yellow color presents ligands. (e, f) Share the pose and surface view of protein and ligand complex. Here, protein is in purple and cyan colors and ligand is in blue color. (g, h) Share 3D and 2D interactions of protein and ligand complex. Here, protein is in agenta color and ligand is in yellow color. (i, j) Share the pose and surface view of protein and ligand complex. Here, protein is in purple and cyan colors and ligand is in blue color. (k, l) Share 3D and 2D interactions of protein and ligand complex. Here, protein in magenta color and ligand in yellow color.

**Figure 3:**
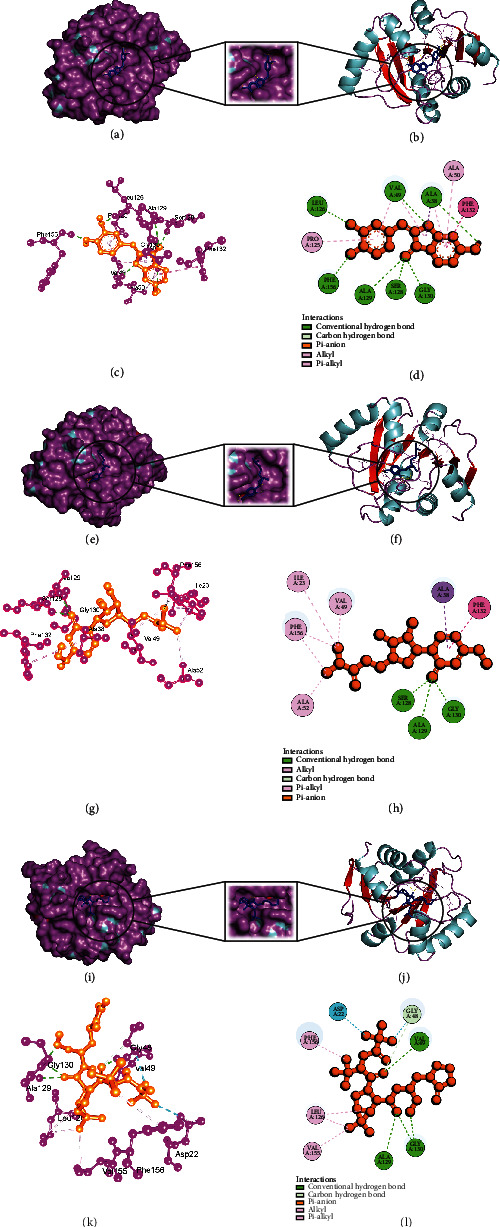
Schematic illustration of 7KQP_CHEMBL490355 (Sulfuretin), 7KQP_Molnupiravir, and 7KQP_Paxlovid complexes. (a, b) Share the pose and surface view of protein and ligand complex. Here, protein is in purple and cyan colors and ligand is in blue color. (c, d) Share 3D and 2D interactions of protein and ligand complex. Magenta color represents proteins and yellow color presents ligands. (e, f) Share the pose and surface view of protein and ligand complex. Here, protein is in purple and cyan colors and ligand is in blue color. (g, h) Share 3d and 2D interactions of protein and ligand complex. Here, protein in magenta color and ligand in yellow color. (i, j) share the pose and surface view of protein and ligand complex. Here, protein is in purple and cyan colors and ligand is in blue color. (k, l) Share 3D and 2D interactions of protein and ligand complex. Here, protein is in magenta color and ligand is in yellow color.

**Figure 4:**
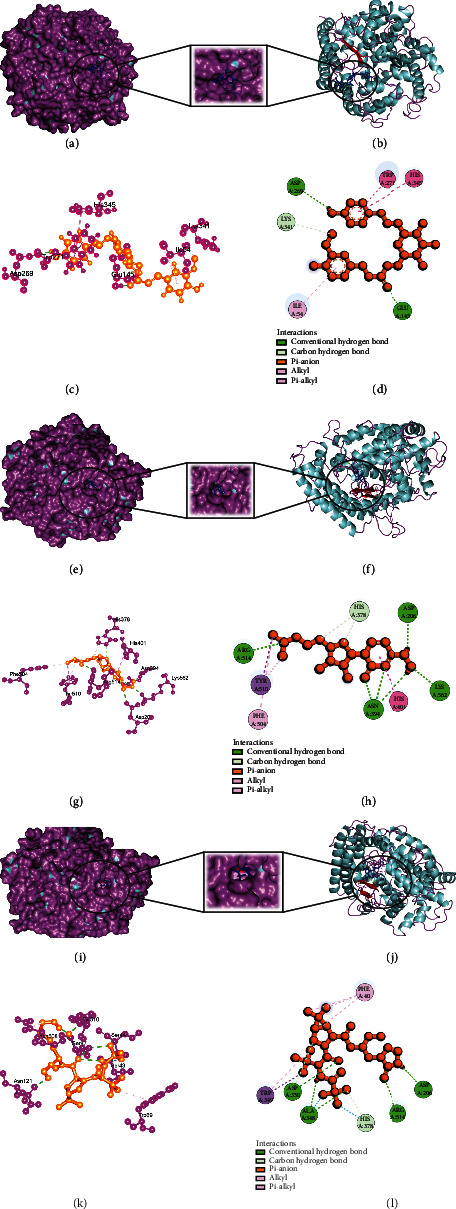
Schematic illustration of 1R4L_CHEMBL4216332 (Grayanoside A), 1R4L_Molnupiravir, and 1R4L_Paxlovid complexes. (a, b) Share the pose and surface view of protein and ligand complex. Here, protein is in purple and cyan colors and ligand is in blue color. (c,, d) Share 3D and 2D interactions of protein and ligand complex. Magenta color represents proteins and yellow color presents ligands. (e, f) Share the pose and surface view of protein and ligand complex. Here, protein is in purple and cyan colors, and ligand is in blue color. (g, h) Share 3D and 2D interactions of protein and ligand complex. Here, protein in magenta color and ligand in yellow color. (i, j) Share the pose and surface view of protein and ligand complex. Here, protein is in purple and cyan colors and ligand is in blue color. (k, l) Share 3D and 2D interaction of protein and ligand complex. Here, protein in magenta color and ligand in yellow color.

**Figure 5:**
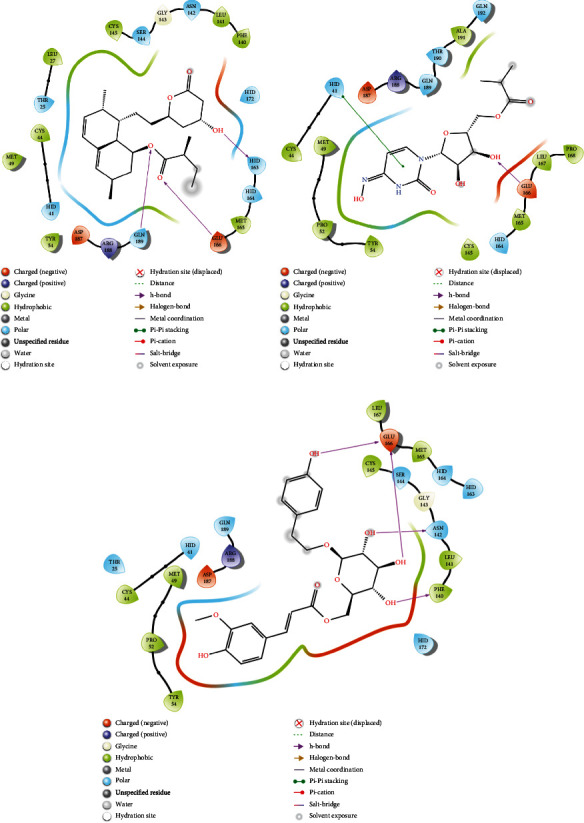
2D interaction of (a) 7NT3_Lovastatin, (b) 7NT3_Molnupiravir, and (c) 7NT3_Paxlovid complexes.

**Figure 6:**
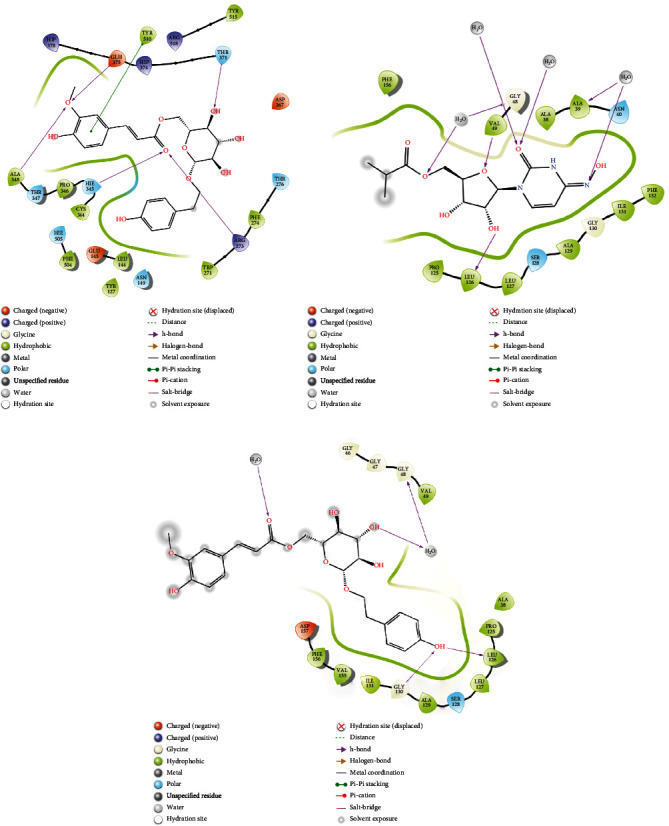
2D interaction of (a) 7KQP_Sulfuretin, (b) 7KQP_Molnupiravir, and (c) 7KQP_Paxlovid complexes.

**Figure 7:**
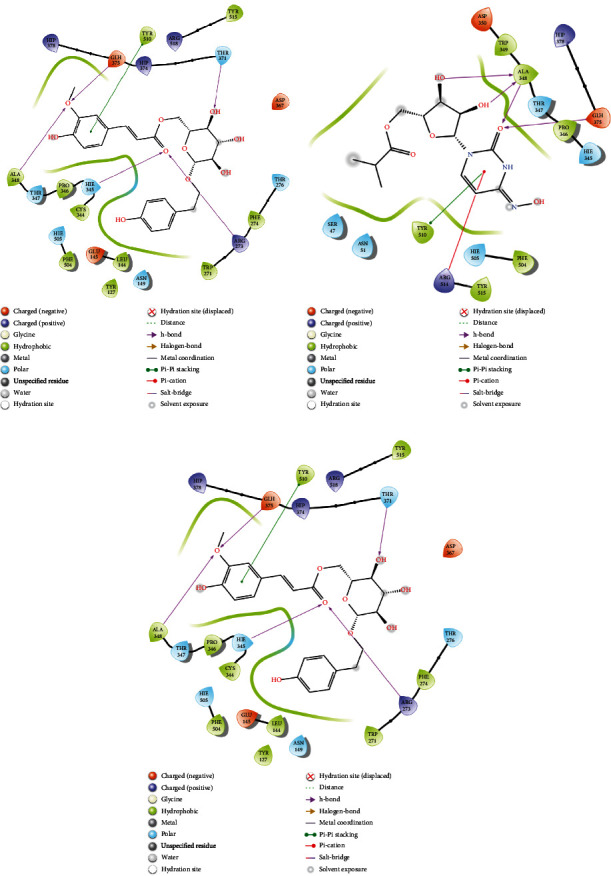
2D interaction of (a) 1R4L_Grayanoside A, (b) 1R4L_Molnupiravir, and (c) 1R4L_Paxlovid complexes.

**Figure 8:**
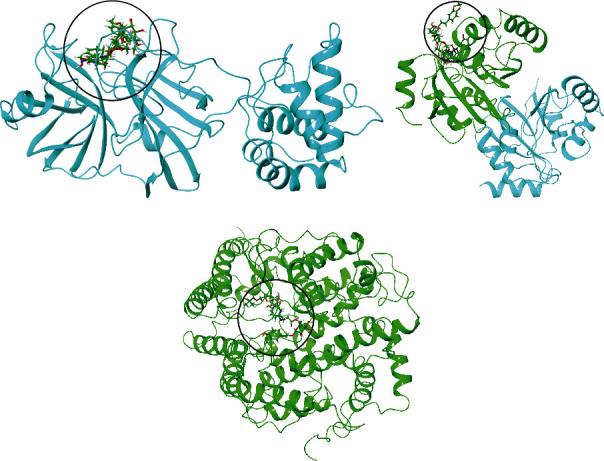
Illustration of 3D representation of (a) 7NT3_complexes, (b) 7KQP_complexes, and (c) 1R4L_complexes. Black circle portrays the binding pockets and incorporates ligands and cocrystallized compounds.

Glide incorporates high-throughput virtual screening (HVS), estimating protein-ligand interacting sites and grading ligands using experimental score systems. Out of the 149 small compounds, 120 small molecules exhibited greater binding energy for SARS-CoV-2 main protease (Supplementary Table 4a), 75 small molecules for Nsp3 (Supplementary Table 4b), and 99 small molecules for human ACE2 receptor compared to control therapeutics (Supplementary Table 4c) (Figures 2–4), and it showed the comparative representation of protein-ligand complexes of the best hit ligands and control drugs. Here, Tables 6–8 summarized the Glide score and MM-GBSA scores between three target proteins and the best hit phytochemicals and control drugs. Analysis of glide XP score and MMGBSA values, it was evident that Lovastatin is better candidate than other potential ligands. It formed three hydrogen bonds (HIS163, GLU166, and GLN189) and nine hydrophobic bonds (LEU27, CYS44, MET49, TYR54, PHE140, LEU141, CYS145, GLY154, and MET165) with the main protease (PDB ID: 7NT3). Sulfuretin showed glide and MMGBSA scores of -9.563 and -52.85 (kcal/mol). It created four hydrogen bonds (ALA38, ASN40, GLY47, and ALA50) and nine hydrophobic bonds (ALA39, VAL49, PRO125, LEU126, LEU127, ALA129, ILE131, PHE132, and PHE156). Grayanoside A managed a glide and MMGBSA scores of -7.87 and -63.54 (kcal/mol). It maintained four hydrogen bonds (ARG273, HIS345, ALA348, and GLN375) and ten hydrophobic bonds (TYR127, LEU144, TRP271, PHE274, CYS344, PRO346, ALA348, PHE504, TYR510, and TYR515) with the human ACE2 receptor (PDB ID: 1R4L) (Figures 5–7). Lovastatin, Sulfuretin, and Grayanoside A were found inside the binding cavity with the cocrystallized compound (Figure 8). As a result, they were proved to be the best candidate for main protease and Nsp3 of SARS-CoV-2 and human ACE2 proteins, respectively.

### 3.5. Analysis of Molecular Dynamics Simulation

**Figure 9:**
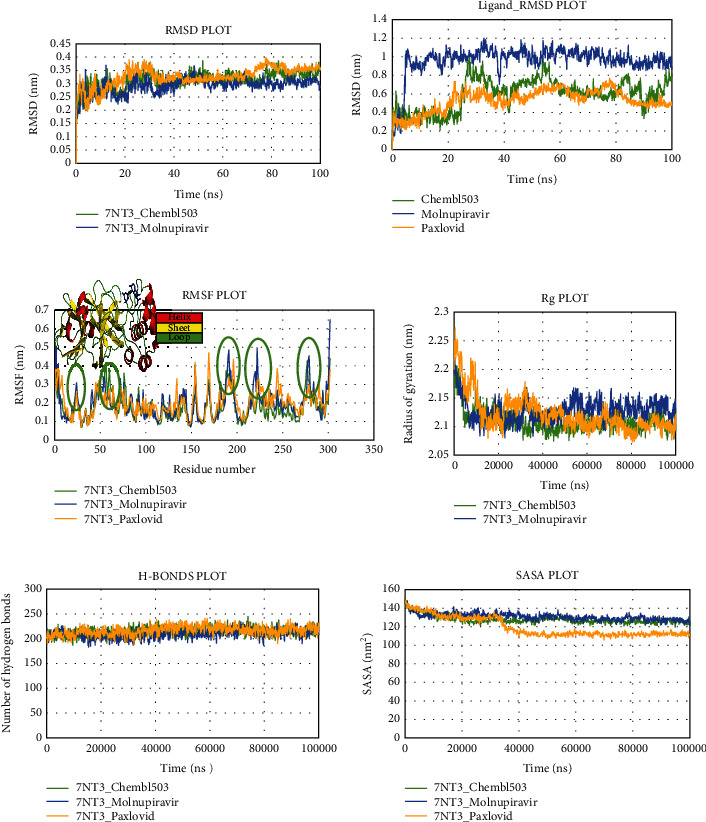
Schematic illustration of 100 ns molecular dynamics simulation of 7NT3_CHEMBL503 (Lovastatin) (green), 7NT3_Molnupiravir (blue), and 7NT3_Paxlovid complexes (yellow). Representations (a, b, c, d, e, and f) share the RMSD, RMSF, Rg, hydrogen bonds, and SASA values of 7NT3_CHEMBL503 (Lovastatin), 7NT3_Molnipiravir, and 7NT3_Paxlovid complexes. Representation b shares ligand RMSD value of Chembl503, Molnupiravir and Paxlovid.

To circumvent the fundamental drawback of molecular docking, we ran a computational MD simulation, which incorporated the dynamic character of the protein following inhibitor binding. This experiment produced statistical figures for the RMSD, RMSF, hydrogen bonds, SASA, and Rg values of protein-ligand complexes. The average RMSD of main protease_Lovastatin, main protease_Molnupiravir, and main protease_Paxlovid complexes for the main protease was 0.312696293 nm, 0.291836715 nm, and 0.326214306 nm, respectively, indicating that the chosen drug candidate Lovastatin exhibited an identical result compared to Molnupiravir and Paxlovid (Figure 9). As per Figure 9, the main protease_Lovastatin and main protease_Molnupiravir complexes were stable with a fixed RMSD value less than 0.35 from 30 to 80 ns, but the main protease_Paxlovid complex had an increased RMSD value more than 0.35 after 75 ns. Similarly, the predicted average RMSD values of the ligands Lovastatin, Molnupiravir, and Paxlovid were 0.594898993 nm, 0.96096714 nm, and 0.531292037 nm, respectively. Throughout 100 ns simulation, the RMSF value of amino acids for backbone components of the main protease_Lovastatin complex was less than 0.4 nm, but the main protease_Molnupiravir and main protease_Paxlovid complexes showed some inconsistent higher fluctuation. The RMSD fluctuation of the ligands inside the first three loop regions between 50 and 80 amino acids was less than 0.40 nm. However, the RMSF oscillation inside the other three considerably larger loop areas was higher than 0.40 for Molnupiravir and Paxlovid. The average Rg values of the complexes main protease_Lovastatin, main protease_Molnupiravir, and main protease_Paxlovid were 2.109437 nm, 2.128492 nm, and 2.122325654 nm, respectively, describing the increased compactness of the Lovastatin complex. Main protease_Lovastatin, main protease_Molnipiravir, and main protease_Paxlovid complexes had an average of 215.0, 209.0, and 216.0 hydrogen bonds, respectively, showing a significant dynamic interaction of the main protease_Lovastatin complex.  Figure 9(f) represented the solvent-accessible surface area (SASA) of structures. While the main protease_Lovastatin and main protease_Molnupiravir complexes had an average SASA value of 127.8404086 nm^2^ and 130.891962 nm^2^, the main protease_Paxlovid complex had a lower value of 119.4976923 nm^2^.

**Figure 10:**
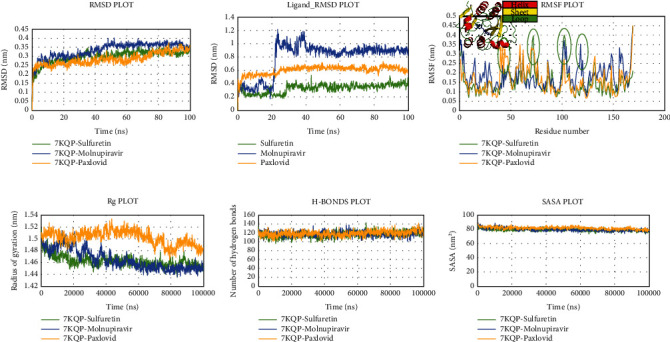
Schematic illustration of 100 ns molecular dynamics simulation of 7KQP_CHEMBL490355 (Sulfuretin) (green), 7KQP_Molnupiravir (blue), and 7KQP_Paxlovid complexes (yellow). Representations (a, b, c, d, e, and f) shares the RMSD, RMSF, Rg, hydrogen bonds, and SASA values of 7KQP_CHEMBL490355 (Sulfuretin), 7KQP_Molnipiravir, and 7KQP_Paxlovid complexes. Representation b share Ligand RMSD value of CHEMBL490355, Molnupiravir, and Paxlovid.

The average RMSD value of the Nsp3_Sulfuretin and Nsp3_Paxlovid complexes for SARS-CoV-2 Nsp3 protein was 0.297815 nm and 0.284552759 nm, respectively, though the Nsp3_Molnupiravir complex displayed an increased variation of RMSD value exceeding 0.35 nm after 45 ns (Figure 10). During the 100 ns simulation timeline with Nsp3 protein, control drugs molnupiravir and Paxlovid had an RMSD value above 0.6 nm and 0.8 nm. However, after an initial equilibration phase, Sulfuretin stayed below 0.4 nm, indicating the most stable of the three ligands. Except for the C-terminal and N-terminal areas, the RMSF value of the Nsp3_Sulfuretin, Nsp3_Molnupiravir, and Nsp3_Paxlovid complexes was less than 0.4 nm. Furthermore, there was higher fluctuation among the structures inside larger loop sections between 41 and 46, 83 and 91, 97 and 105, and 116 and 135 amino acids. The Rg values of the Nsp3_Sulfuretin and Nsp3_Molnupiravir complexes stabilized after initial equilibration steps, but the Rg value of Nsp3_Paxlovid complexes oscillated more during the whole run time. According to Figure 10(e), the average count of hydrogen bonds among the complexes Nsp3_Sulfuretin, Nsp3_Molnupiravir, and Nsp3_Paxlovid were 116.0, 117.0, and 119.0, indicating a similar course of interaction within the 100 ns timeframe. The SASA value of the Nsp3_Sulfuretin, Nsp3_Molnipiravir, and Nsp3_Paxlovid complexes were stable with an average value of 79.26847 nm, 79.74635 nm, and 81.74065634 nm respectively.

**Figure 11:**
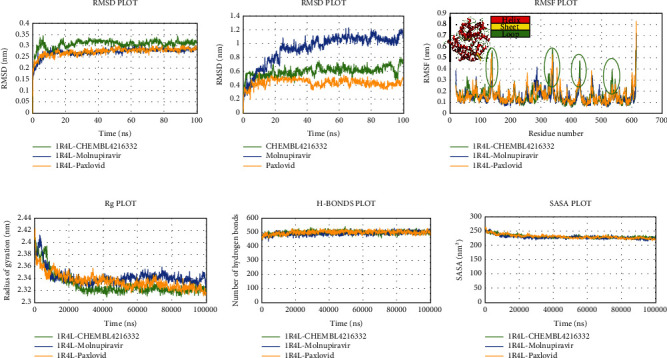
Schematic illustration of 100 ns molecular dynamics simulation of 1R4L_CHEMBL4216332 (Grayanoside A) (green), 1R4L_Molnupiravir (blue), and 1R4L_Paxlovid (yellow). Representations (a, b, c, d, e, and f) share the RMSD, RMSF, Rg, hydrogen bonds, and SASA values of 1R4L_CHEMBL4216332 (Grayanoside A), 1R4L_Molnipiravir, and 1R4L_Paxlovid complexes. Representation b share ligand RMSD value of CHEMBL4216332, Molnupiravir and Paxlovid.

The RMSD value of the human ACE2 receptor_Grayanoside A, human ACE2 receptor_Molnupiravir, and human ACE2 receptor_Paxlovid complexes for human ACE2 protein stayed under 0.35 nm, and stable throughout the 100 ns run (Figure 11). Likewise, the ligands Grayanoside A, Molnupiravir, and Paxlovid had average RMSD values of 0.601344 nm, 0.933326 nm, and 0.43800 nm, respectively. The RMSF of backbone heteroatoms per residue of the human ACE2 receptor_Grayanoside A complex stayed within 0.4 nm, with higher RMSF oscillation inside loops from 137 to 139 and 338 to 340 residues. On the other hand, peaks inside loop regions beyond 0.4 nm were evident from 137 to 140 and 331 to 345 residues for human ACE2 receptor_Molnupiravir and human ACE2 receptor_Paxlovid complexes, respectively. The average Rg values of human ACE2 receptor_Grayanoside A, human ACE2 receptor_Molnipiravir, and human ACE2 receptor_Paxlovid complexes were 2.329435, 2.342172667, and 2.335405325 nm, respectively. The average hydrogen bond interactions for the complexes human ACE2 receptor_Grayanoside A and human ACE2 receptor_Molnupiravir were 499.0 and 492.0, respectively, whereas the complex human ACE2 receptor_Paxlovid had a higher value of 498.0. Figure 11 shows that the SASA values of the human ACE2 receptor_Grayanoside A, human ACE2 receptor_Molnupiravir, and human ACE2 receptor_Paxlovid complexes were stable throughout the simulation, suggesting that the protein's surface area remained unaltered.

### 3.6. Evaluation of MD Simulation and Post-MD Simulation MM-GBSA Results from Desmond

**Figure 12:**
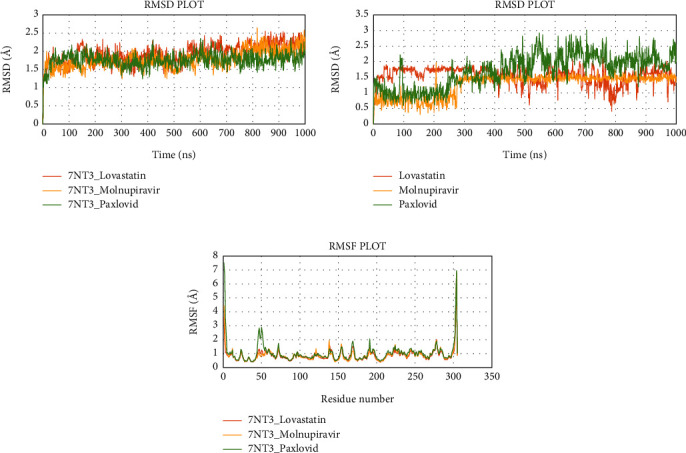
Simulation graph of root-mean-square deviation (RMSD) showing Lovastatin_7NT3 (orange), Molnupiravir_7NT3 (yellow), and Paxlovid_7NT3 (green). (b) Simulation graph of root-mean-square deviation (RMSD) showing Lovastatin (orange), Molnupiravir (yellow), and Paxlovid (green). (c) Simulation findings showing of root-mean-square fluctuation (RMSF) of Lovastatin_7NT3 (orange), Molnupiravir_7NT3 (yellow), and Paxlovid_7NT3 (green).

**Figure 13:**
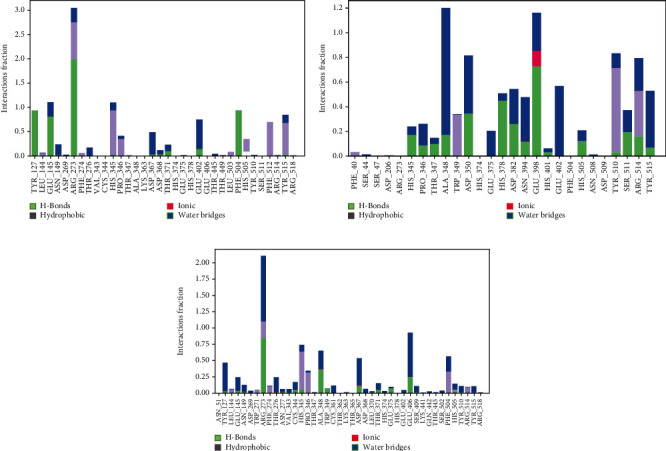
Contact maps of Lovastatin_7NT3 (a), Molnupiravir_7NT3 (b), and Paxlovid_7NT3 (c) complexes.

Analyzing the simulation trajectory, we plotted the RMSF, RMSD, biophysical properties of ligands, and protein-ligand network. We found an average RMSD plot of 1.92, 1.78, and 1.75 Å for Lovastatin_7NT3, Molnupiravir_7NT3, and Paxlovid_7NT3 complexes. The protein structure of the Sulfuretin_7NT3 complex remained under 3 Å throughout the simulation. The ligands Sulfuretin, Molnupiravir, and Paxlovid had average RMSD of 1.55, 1.27, and 1.71 Å respectively, indicating a stable conformation with protein. Similarly, the average RMSF of Lovastatin_7NT3, Molnupiravir_7NT3, and Paxlovid_7NT3 complexes was 0.87, 0.91, and 1.04 Å respectively. Except for N-terminal and C-terminal zones, all complexes stayed under 3 Å (Figure 12). Sulfuretin interacted with 7NT3 creating bonds with THR26 (hydrogen bonds and water bridges), GLY143 (hydrogen bonds and water bridges), SER144 (hydrogen bonds and water bridges), CYS145 (hydrogen bonds and water bridges), and GLU166 (hydrogen bonds, water bridges, and ionic bonds) amino acids for 30%, 20%, 30%, 40%, 20%, and 100% of 100 ns timeframe. Molnupiravir interacted with HIS41 (hydrophobic), GLU166 (water bridges), VAL186 (hydrogen bonds and water bridges), and GLN189 (hydrogen bonds and water bridges) for 80%, 100%, 70%, and 90% of 100 ns timescale. Paxlovid had bonds with HIS41 (hydrophobic, hydrogen bonds, and water bridges), GLY143 (hydrogen bonds and water bridges), SER144 (hydrogen bonds and water bridges), and GLU166 (hydrogen bonds, water bridges, and ionic bonds) for 50%, 90%, 40%, and 300% of the simulation period (Figure 13).

**Figure 14:**
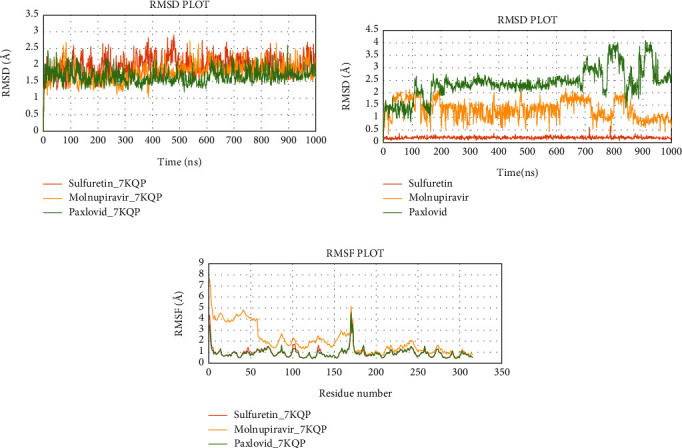
Simulation graph of root-mean-square deviation (RMSD) showing Sulfuretin_7KQP (orange), Molnupiravir_7KQP (yellow), and Paxlovid_7KQP (green). (b) Simulation graph of root-mean-square deviation (RMSD) showing Sulfuretin (orange), Molnupiravir (yellow), and Paxlovid (green). (c) Simulation findings showing of root-mean-quare fluctuation (RMSF) of Sulfuretin_7KQP (orange), Molnupiravir_7KQP (yellow), and Paxlovid_7KQP (green).

**Figure 15:**
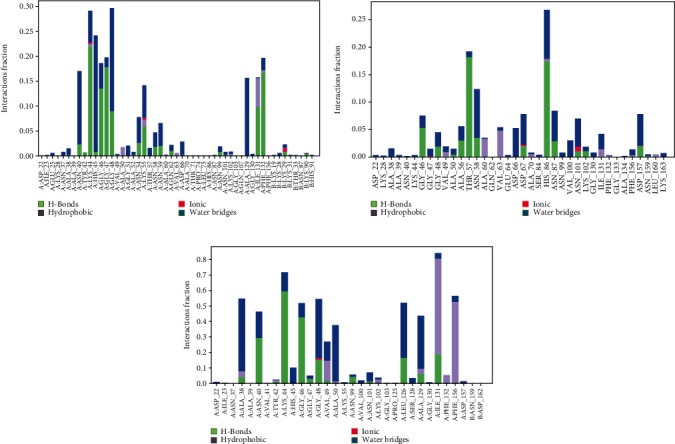
Contact maps of Sulfuretin_7KQP (a), Molnupiravir_7KQP (b), and Paxlovid_7KQP (c) complexes.

Protein structures of Sulfuretin_7KQP, Molnupiravir_7KQP, and Paxlovid_7KQP showed an average RMSD value of 1.97, 1.77, and 1.65 Å. All the complex structures remained under 3 Å which suggested that the ligands were tightly bound inside the binding pocket of receptor structures. The ligands Sulfuretin, Molnupiravir, and Paxlovid displayed an average RMSD of 0.19, 1.33, and 2.37 Å respectively. RMSF plot presented an average of 0.94, 1.97, and 0.92 Å for Sulfuretin_7KQP, Molnupiravir_7KQP, and Paxlovid_7KQP complexes implying structural stability (Figure 14). Sulfuretin made bonds with ASN40 (hydrogen bonds and water bridges), LYS44 (hydrogen bonds, water bridges, and ionic bonds), HIS45 (hydrogen bonds and water bridges), GLY48 (hydrogen bonds and water bridges), PHE156 (hydrogen bonds and water bridges) residues of 7KQP for 17%, 30%, 25%, 30%, and 20% of simulation timeframe. Molnupiravir_7KQP complex formed bonds with THR57 (hydrogen bonds and water bridges), ASN58 (hydrogen bonds and water bridges), HIS86 (hydrophobic, hydrogen bonds, and water bridges) residues for 20%, 11%, and 26% of simulation timeframe. Paxlovid_7KQP complex maintained binding network with ALA38 (hydrophobic, hydrogen bonds, and water bridges), ASN40 (hydrogen bonds and water bridges), LYS44 (hydrogen bonds and water bridges), GLY46 (hydrogen bonds and water bridges), GLY48 (hydrogen bonds, water bridges, and ionic bonds), ILE131 (hydrophobic, hydrogen bonds, and water Bridges), and GLU156 (hydrophobic and water bridges) residues for 55%, 45%, 70%, 52%, 55%, 80%, and 55% of the simulation run (Figure 15).

**Figure 16:**
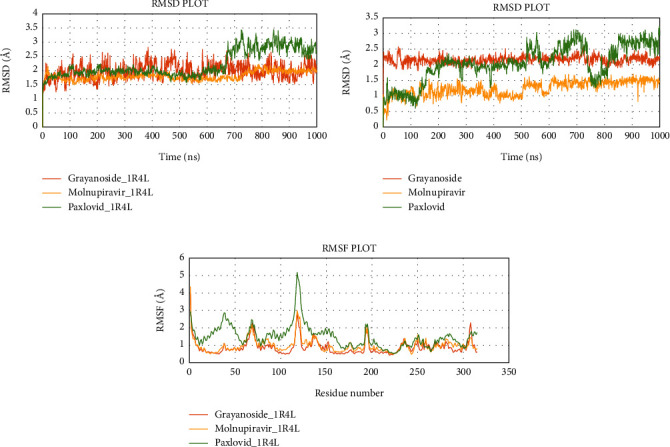
Simulation graph of root-mean-square deviation (RMSD) showing Grayanoside A_1R4L (orange), Molnupiravir_1R4L (yellow), and Paxlovid_1R4L (green). (b) Simulation graph of root-mean-square deviation (RMSD) showing Grayanoside A (orange), Molnupiravir (yellow), and Paxlovid (green). (c) Simulation findings showing of root-mean-square fluctuation (RMSF) of Grayanoside A_1R4L (orange), Molnupiravir_1R4L (yellow), and Paxlovid_1R4L (green).

**Figure 17:**
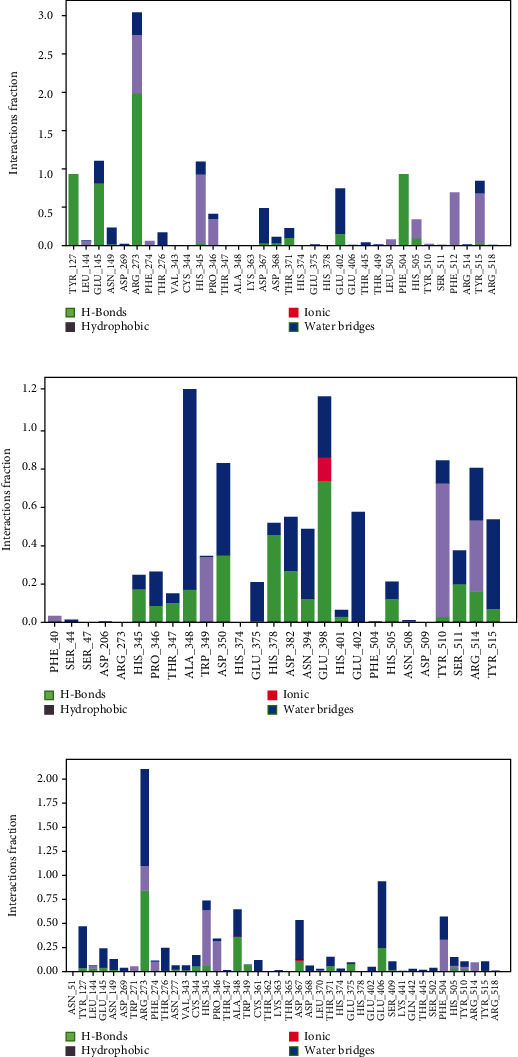
Contact maps of Grayanoside A_1R4L (a), Molnupiravir_1R4L (b), and Paxlovid_1R4L (c) complexes.

**Figure 18:**
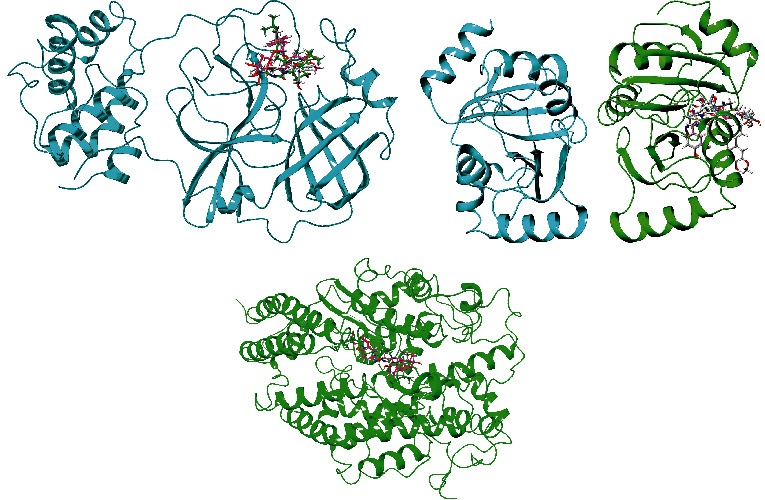
Superimposed representation of the pre-MD and post-MD structures of Ligand_7NT3, Ligand_7KQP, and Ligand_1R4L complexes.

Next, the Grayanoside A_1R4L, Molnupiravir_1R4L, and Paxlovid_1R4L complex structures maintained an average RMSD of 1.97, 1.80, and 2.22 Å respectively. Grayanoside A_1R4L complex remained 2.7 Å throughout the timeframe demonstrating a stable protein-ligand association. The average RMSD of the ligands Grayanoside A, Molnupiravir, and Paxlovid was 2.15, 1.23, and 2.03 Å respectively. The proteins of Grayanoside A_1R4L, Molnupiravir_1R4L, and Paxlovid_1R4L complexes maintained an average RMSD of 0.83, 0.93, and 1.31 Å respectively. A small steep was observed between 115 to 125 residues for Grayanoside A_1R4L and Molnupiravir_1R4L complexes (Figure 16). Grayanoside A had TYR127 (hydrogen bonds), GLU145 (hydrogen bonds and water bridges), ARG273 (hydrophobic, hydrogen bonds, and water bridges), HIS345 (hydrophobic and water bridges), GLU402 (hydrogen bonds and water bridges), PHE504 (hydrogen bonds), and HIS505 (hydrophobic, hydrogen) binding residues with 1R4L protein for 90%, 110%, 330%, 110%, 80%, 100%, and 40% of simulation cycle. Molnupiravir_1R4L complex formed interaction with ALA348, ASP350, GLU398, TYR510, and ARG514 for 120%, 80%, 119%, 82%, and 80% of the simulation period. On the other hand, Paxlovid_1R4L complex had interactions with ARG273, HIS345, ALA348, and GLU406 residues for 200%, 70%, 65%, and 90% of the simulation timescale (Figure 17). We also superimposed the pre_MD and post_MD structures of protein-ligand complexes in Desmond and found less than 2 Å deviation (Figure 18).

The average postsimulation MM-GBSA of Lovastatin_7NT3, Molnupiravir_7NT3, and Paxlovid_7NT3 complexes were −52.56 ± 8.93, −50.52 ± 12.75, and −49.68 ± 16.27 kcal/mol, respectively. Sulfuretin_7KQP, Molnupiravir_7KQP, and Paxlovid_7KQP complexes had average postsimulation MM-GBSA scores of −66.17 ± 11.62, -36.51 ± 13.74, and −54.30 ± 15.45 kcal/mol, respectively. Grayanoside A_1R4L, Molnupiravir_1R4L, and Molnupiravir_1R4L complexes showed an average MM-GBSA value of −74.94 ± 8.50, −34.23 ± 12.82, and −57.50 ± 24.35 kcal/mol, respectively, (Tables 9–11).

### 3.7. Analysis of Target within Human

**Figure 19:**
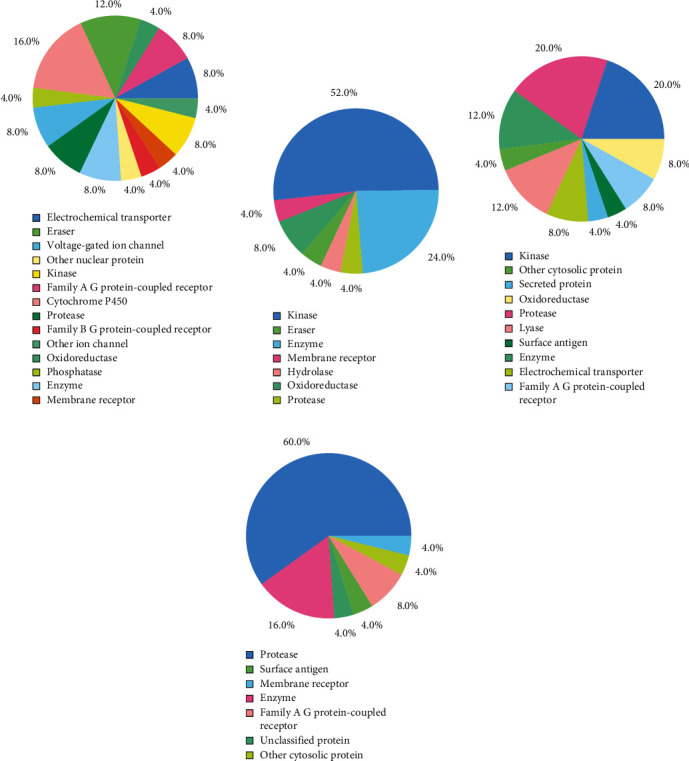
Predicted top 25 classes of *H. sapiens* molecular targets for (a) Lovastatin, (b) Sulfuretin, (c) Grayanoside A, and (d) Paxlovid.

The target sites in humans where Lovastatin binds (in humans) are cytochrome p450, oxidoreductase, kinase, family A of G protein-coupled receptor, enzyme, and membrane receptor and the possibility of binding, respectively, are 16%, 12%, 8%, 8%, 8%, and 4% respectively. For Sulfuretin, they they may bind with kinase (52%), enzyme (24%), and membrane receptor (4%); and for Grayanoside A, they they may bind with protease (20%), kinase (20%), surface antigen (4%), enzyme (12%), and family A of G protein-coupled receptor (4%). Control drug Paxlovid provides the binding possibility in target sites are protease (60%), enzyme (16%), family A of G protein-coupled receptor (8%), membrane receptor (4%), and surface antigen (4%). The prediction tool did not show any human target for Molnupiravir (Figure 19).

### 3.8. Analysis of Activity Spectra of the Phytochemicals

Using the identified compounds, prediction of activity spectra for substances (PASS) was carried out and is shown in Supplementary Tables 6a, 6b, 6c. In our study, we used PASS to build a predictive model, and we kept the Pa (likelihood of activity) that was higher than 70%; since an absolutely durable forecast may be made using the Pa > 70% (0.7) criteria. Lovastatin had 18 biological activities, Sulfuretin showed 27 activities, and Grayanoside A possessed 30 biological features. Lovastatin, Sulfuretin, and Grayanoside A present Pa values greater than 0.70 across the board to be considered for use as an active biological agent in the treatment of SARS-CoV-2.

## 4. Discussion

In recent years, pandemics and epidemics caused by viruses have become one of the most prevalent reasons for infections and mortality worldwide. SARS-CoV-2, the updated variant of coronaviruses, has led to a catastrophe, with 665,518,891 and 6,714,212 confirmed cases and fatalities, respectively (11th January,2023; https://covid19.who.int/). Surprisingly, currently, a limited amount of effective anti-SARS-CoV-2 therapeutics are available, and most of them are under investigation [43].

Following the outbreak of SARS-CoV-2, Mpro, also regarded as 3CLpro (main protease), became a viable therapeutic focus due to its involvement in the development of replication-translation mechanisms. Furthermore, given the accessibility of high-resolution protein structures, these proteins have a highly conserved sequence and no homology with human proteins [44]. Nsp3 is a multidomain protein with a Glu-rich acidic domain at the N-terminus, an X domain, a SUD domain, a papain-like protease domain, and a transmembrane domain. Nsp3 is responsible for viral multiplication and pathogenesis in humans and facilitates immune evasion via its hydrolyzing capability [43]. The attachment of the SARS-CoV-2 Spike protein to human ACE2 receptor on the cellular wall permits the virus to enter cells, which is required for infection to begin [45]. To inhibit these viral proteins, we utilized phytochemicals with drug-like properties.

This research was divided into three sections, namely, virtual screening (VS) of the physicochemical and pharmacokinetic features of drug-like compounds, virtual screening by molecular docking of proteins and ligands, and simulation of the best hit complexes. In the initial stage, we studied the drug-like characteristics of the ligands utilizing the five principles of Lipinski. We stuck to the established guidelines, i.e., hydrogen bond donors ≤ 5, hydrogen bond acceptors ≤ 10, molecular mass < 500, and logP < 5. The molecular weight of a small molecule can influence its absorption, bile excretion ratio, blood-brain barrier passage, and engagements involving receptors [46]. Likewise, hydrogen donor and hydrogen acceptor groups are mostly responsible for the permeability and polarity of the drug-like molecules. Lipophilicity is an indicator that influences the metabolism and solubility of those molecules. A lower or higher score might impede this characteristic [47]. TPSA refers to the area belonging to polar atoms like nitrogen, oxygen, and their associated hydrogens [48]. Out of 1163 small molecules, 497 of them passed the criteria. We tested the pharmacokinetic figure of the ligands before analyzing their binding affinity and orientation. The characteristics of a small molecule in terms of ADMET properties make it a viable candidate. Using the human intestinal absorption (HI) prediction score and the Caco-2 permeable theory, the probability that the small molecules would reach systemic circulation and exert their function was calculated [49]. P-glycoprotein serves as a drug carrier and eliminating compounds from different organs [50]. The main subfamily (2D6, 2C9, and 3A4) of cytochrome P450 monooxygenase (CYP) enzymes is crucial in the metabolism of the drug-like molecules [51]. In the initial phases of pharmaceutical research, AMES mutagenicity is commonly used to determine the probability of genotoxicity and teratogenicity [52]. Cardiovascular poisoning might be caused by inhibiting the cardiac human ether-a-go-go-related (hERG) gene [53]. We also tested the maximum tolerated dose of chemical substances for the human body. Eventually, only 149 drug-like molecules passed the ADMET evaluation.

The importance of virtual screening employing molecular docking has grown significantly in the field of drug development over time. According to the study, 24 small molecules had a greater binding affinity against the main protease (7NT3) than the reference compounds: Molnupiravir and Paxlovid (-5.035 and -5.185 kcal/mol, respectively). The MM-GBSA approach is recognized for its impressive precision in estimating the free binding energy of small molecules to target proteins. Both analyses indicated that Lovastatin (CHEMBL503) had a higher glide score and binding-free energy value of -6.01 kcal/mol and -52.85 kcal/mol, respectively. Recently, Mashraqi et al. found fenoterol had a glide score and MM-GBSA values of −7.14 and -38.733 kcal/mol [54]. We found Lovastatin attached to the active site residues (His41, Cys145) in the docking study and after MD simulation. Though CHEMBL182992, CHEMBL1909923, CHEMBL1972346, CHEMBL249454, CHEMBL477778, CHEMBL557501, and CHEMBL4216332 had better glide XP scores over 6 kcal/mol, the binding-free energy is higher for CHEMBL503 (Lovastatin). Similarly, two small compounds, CHEMBL490355 (Sulfuretin) and CHEMBL226683, showed binding energies greater than 9.0 Kcal/mol than control therapeutics against Nsp3 (7KQP). But the binding free energy (-46.31 kcal/mol) and the number of hydrogen bonds were higher for CHEMBL490355 (Sulfuretin). So, Sulfuretin was selected as the best candidate against Nsp3. Recently, Mishra et al. reported ZINC82673 as the potential inhibitor of Nsp3 with glide and MM-GBSA values of −9.348 and 50.175 kcal/mol [55]. It was also found inside the binding pocket (Asp22, Ile23, Gly48, Val49, Gly130, or Phe156) [43]. A total of 20 phytochemicals had higher glide XP scores over 6 kcal/mol and 2 of them showed binding-free energy above –50 kcal/mol against human ACE2 receptor. Based on the glide and MMGBSA scores, as well as the number of hydrogen bonds, we selected Grayanoside A as the lead candidate against human ACE2 receptor (1R4L). Most of the residues of the active site (Tyr515, Arg514, His505, Phe504, Glu402, His378, Glu375, His374, Asp368, Cys361, His345, Cys344, and Glu145) were found attached to Grayanoside A [56]. Pai et al. found that iso-chlorogenic acid showed inhibition activity against human ACE2 receptor with a glide score MM-GBSA values of −8.799 and −44.248.

MD simulations offer a plethora of energetic data on protein and ligand binding, as well as a wealth of structural figures on biological macromolecules. This type of knowledge is crucial for comprehending the structural and functional correlation of the receptor and the basis of protein-ligand association, and also for steering the therapeutic research [51]. During the simulation trajectory, the RMSD of the protein C*α* and RMSF of the amino acids, also the ligand-protein H-bonding association, the solvent-accessible surface area (SASA), and the radius of gyration (Rg), were assessed to determine the steadiness of the complex structures [52]. The RMSD value is considered to indicate the flexibility and dynamic character of the protein. It showcased the movement of amino acids along with the MD simulation [38]. Thus, a relatively large RMSD value suggested more motion, whereas a relatively low RMSD value of protein showed less movement. The RMSD results suggested that the RMSD value of the main protease_Lovastatin backbone was identical to those of the reference complexes main protease_Molnupiravir and main protease_Paxlovid. The ligands Lovastatin and Paxlovid remained steady, with two short peaks. As a result, the protein might remain stable during the simulation, after the attachment of the Lovastatin molecule. A detailed investigation of the RMSF demonstrated the specific fluctuation of amino acids in the catalytic and noncatalytic areas of the protein-ligand complexes. The RMSF value confirmed that the attachment of Lovastatin to the receptor might not increase flexibility. The Rg values display the compactness of the protein with folding and unfolding nature by the thermodynamic concept. The interaction of the ligand Lovastatin did not modify the structure of the protein. Hydrogen bonds are another vital determinant of protein-ligand stability. Protein-ligand interaction is stronger with more hydrogen bonds. When compared to the reference complexes, the main protease_Lovastatin complex had a similar amount of hydrogen bonds, indicating a stable protein-ligand construct. The unfolding of the protein during the denaturation process exposes nonpolar hydrophobic interactions to the aqueous system. As a result, the protein's structure is disrupted. The SASA value computing determines the fluctuation in the accessibility of protein to solvent [57]. The SASA analysis revealed a similar tendency, with both main protease_Lovastatin and main protease_Molnupiravir complexes exhibiting significant similarities throughout the simulation.

In the context of Nsp3, the ligand Sulfuretin did not produce conformational changes to the protein. Firstly, the RMSD value revealed that Nsp3_Sulfuretin was consistently stable compared to the reference complexes. Throughout the simulation, the Sulfuretin molecule remained relatively stable. Upon binding of the Sulfuretin molecule, the Nsp3_Sulfuretin complex displayed lesser fluctuation in comparison to the reference complexes. According to the Rg value of the Nsp3_Sulfuretin complex, it remained steady during the simulation timeframe, suggesting the compactness of the protein following inhibitor binding. Nsp3_Sulfuretin complex displayed a similar amount of hydrogen interactions as the reference complexes demonstrating excellent protein-ligand stability. Similarly, the SASA value revealed that the Nsp3_Sulfuretin complex remained unchanged throughout the simulation, supporting earlier findings. The simulation results for human ACE2 protein showed that the binding of the Grayanoside A molecule caused a small consequence on the structure of human ACE2 protein. The RMSD graph of protein-ligand complexes and ligands implied that the ligand (CHEMBL1909923) might not destabilize the protein. The RMSF results revealed that there was a similar fluctuation, suggesting the identical nature of the binding of the three ligands (CHEMBL1909923, Molnupiravir, and Paxlovid). The plots of Rg, hydrogen bond, and SASA value also confirmed the previous viewpoint, indicating that the Grayanoside A molecule's attachment did not impair the stability of human ACE2 protein. In case of Desmond, we found similar results that further validate our findings. The RMSD values suggested that Lovastatin, Sulfuretin, and Grayanoside A were firmly bound to the proteins. The RMSF plots implied that the main protease (3CLpro), Nsp3, and human ACE2 receptor were structurally stable while bound with respective ligands. The protein-ligand attachment maps continuously showed that the proposed ligands-maintained contact with active site residues. Lastly, the postsimulation MM-GBSA results stated that Lovastatin, Sulfuretin, and Grayanoside A had a higher free-binding affinity for their respective proteins.

Previous structure-based computational research yielded similar findings, demanding wet-lab investigation. According to study lead by Gurung et al., bonducellpin D was found as a potential inhibitor for SARS-CoV-2 3CLpro protein [58]. In another study, Eissa et al. identified vidarabine as prospective antiviral agent for SARS-Cov-2 nonstructural protein-10 [59]. Ottavia Spiga et al. found simeprevir and lumacaftor the most potent inhibitors of Spike protein on the basis of their computational findings [60]. Kusumaningsih et al. found luteolin and naringenin as the probable drug candidates for main protease of SARS-CoV-2 [61]. Lovastatin, Sulfuretin, and Grayanoside A have been reported as antiviral agents [62–64]. Our structure-based strategy again showed antiviral activity of these small substances against SARS-CoV-2 critical protein targets. However, these compounds should be examined further in the pharmaceutical research facility to evaluate their potency, inhibitory power, and toxicity against their respective targets. While there is no denying the enormous success of drug repurposing, the in silico approach is not without its limitations. In a similar fashion, one disadvantage of molecular docking is the lack of proper scoring functions and algorithms, which may compromise molecular screening. Another challenge that researchers face is the difficulty in selecting the most effective target combinations due to the absence of quantifiable data for assessing network dynamics, as well as the inability to construct the molecular network of the disease [18, 65]. Apart from those certain constraints due to data reliability, biasness and irregularities in the available current data, our study shows a comparison between established compounds and screened compounds using several bioinformatics tools and introduces in silico models that can swiftly present a summary of prospective therapeutic options economically and expediently for a microorganism such as SARS-CoV-2, which is constantly mutating and without any established therapy.

## 5. Conclusion

Repurposing drug-like phytochemicals is a secure means of building new therapeutics, with the main benefit of lowering the cost and duration of preclinical trials for novel candidates. The COVID-19 infectious disease induced by the SARS-CoV-2 outbreak has caused a worldwide medical catastrophe and finding a suitable cure for the virus continues to be a primary concern. The findings of our work indicated that using a structure-based strategy such as molecular docking and MD simulations, novel therapeutic candidates may be developed that selectively address the nonstructural protein 3, the main protease from SARS-CoV-2, and the human ACE2 protein. A preliminary screening of 1163 small phytochemicals combining drug-likeness and ADMET characteristics resulted in the identification of 149 of them. The degree of binding interaction and energy between the filtered compounds and the main protease, nonstructural protein 3, and human ACE2 receptor was estimated utilizing the docking procedure on the AutoDock vina and Schrodinger Suites. Compounds Lovastatin, Sulfuretin, and Grayanoside A outperformed Molnupiravir and Paxlovid in terms of binding score and hydrogen bond numbers against the main protease, Nsp3, and human ACE2 receptor, respectively. Eventually, 100 ns MD simulation studies of 3CLpro_ligand, Nsp3_ligand, Grayanoside A_ligand complexes were completed to evaluate and improve our proposed design. This investigation is aimed at determining the promising inhibitors and devise protocols for continual improvement of COVID-19 medications. To summarize, all the repurposed compounds suggested here may provide a holistic understanding of structure-based drug development for SARS-CoV-2 given that they continue to remain potent in further drug development processes.

## Figures and Tables

**Table 1:** Drug-like properties of the best hit phytochemicals and control drugs.

Phytochemicals/ Drugs	MW (g/mol)	Rotatable bonds	H-bond acceptors	H-bond donors	Lipinski violation	Ghose violation	Veber violation	Egan violation	Muegge violation
CHEMBL503 (Lovastatin)	404.54	7	5	1	0	0	0	0	0
CHEMBL490355 (Sulfuretin)	270.24	1	5	3	0	0	0	0	0
CHEMBL4216332 (Grayanoside A)	476.47	10	10	5	0	0	0	0	0
Molnupiravir	329.31	6	8	4	0	1	1	1	0
Paxlovid	501.54	12	8	4	1	1	1	0	0

**Table 2:** ADMET properties of the best hit phytochemicals and control drugs.

Phytochemicals/ Drugs	Human intestinal absorption (% absorbed)	Caco-2 permeability (log Papp in 10-6 cm/s)	BBB permeability (log BB)	CYP2D6 substrate	CYP1A2 inhibitor	AMES toxicity	hERG I inhibitor	hERG II inhibitor	Hepatotoxicity
CHEMBL503 (Lovastatin)	94.656	0.924	-0.366	Yes	No	No	No	No	No
CHEMBL490355 (Sulfuretin)	98.77	1.795	-0.618	Yes	No	No	No	No	No
CHEMBL4216332 (Grayanoside A)	49.67	48.681	-1.266	No	No	No	No	No	No
Molnupiravir	53.464	0.531	-1.057	No	No	No	No	No	Yes
Paxlovid	61.975	0.081	-0.907	No	No	Yes	No	No	Yes

**Table 3:** Binding affinity and nonbonded interaction between the main protease (PDB ID: 7NT3) and the best hit phytochemical and control drugs.

Phytochemicals/ Drugs	Affinity (kcal/mol)	No. of H bonds	Interacting amino acids	No. of hydrophobic bonds	Interacting amino acids	No. of halogen bonds	Interacting amino acids	No. of electrostatic bonds	Interacting amino acids
Lovastatin	-7.2	1	ARG131 (2.30102 Å)	2	LEU28, PRO293	×	×	×	×
Molnupiravir	-6.4	3	ASP197, THR199 (2.04463 Å), LEU287	3	LEU27, TYR23, TYR239	×	×	1	ASP289
Paxlovid	-6.6	6	THR26, HIS41, ASN11, ASN14, GLY143 (1.98365 Å), CYS145	8	HIS49, MET49, ILE249, PRO29, HIS41	3	GLY10, GLN11, ASN203	1	GLU166

**Table 4:** Binding affinity and nonbonded interaction between the Nsp3 (PDB ID: 7KQP) and the best hit phytochemical and control drugs.

Phytochemicals/ Drugs	Affinity (kcal/mol)	No. of H bonds	Interacting amino acids	No. of hydrophobic bonds	Interacting amino acids	No. of halogen bonds	Interacting amino acids
Sulfuretin	-8.8	7	VAL49, LEU126, SER128, ALA129, GLY130, PHE156, ALA38	7	ALA38, PHE132, VAL49, ALA38, ALA50, VAL49, PRO125	×	×
Molnupiravir	-7.7	6	ASN40, GLY47, VAL49, ALA50, LYS44, ALA38 (1.90623 Å)	7	ALA38, PHE132, ALA52, ILE23, VAL49, PHE156	×	×
Paxlovid	-7.5	4	LYS158, LEU160, TYR161 (1.23877 Å)	8	ALA38, VAL49, ALA129, VAL155, LEU160, LEU126, LEU160	1	GLY48

**Table 5:** Binding affinity and nonbonded interaction between the human ACE2 receptor (PDB ID: 1R4L) and the best hit phytochemical and control drugs.

Phytochemicals/ Drugs	Affinity (k cal/Mol)	No of H bonds	Interacting amino acids	No of hydrophobic bonds	Interacting amino acids	No of halogen bonds	Interacting amino acids
Grayanoside A	-7.8	3	ARG273, ARG273, GLU406 (1.84129 Å)	7	Val209, LYS562, TRP566, LEU95, LYS562, ALA99	×	×
Molnupiravir	-7.6	5	ASP206, HIS378, ASN394, ARG514, LYS562 (2.198 Å)	4	TYR51, HIS401, PHE504, TYR510	×	×
Paxlovid	-7	6	ASP206, ALA348, TRP349 (1.978 Å), ASP350, HIS378, ARG514	×	×	1	SER43

**Table 6:** XP Gscore and MM-GBSA values between the main protease (PDB ID: 7NT3) and the best hit phytochemical and control drugs.

Drug	XP Gscore (kcal Mol−1)	MM-GBSA scores (kcal Mol−1)	Hydrogen bonds	Hydrophobic bonds
Lovastatin	-6.01	-52.85	HIS163, GLU166, GLN189	LEU27, CYS44, MET49, TYR54, PHE140, LEU141, CYS145, GLY154, MET165
Molnupiravir	-5.035	-43.48	GLU166	CYS44, MET49, PRO52, TYR54, CYS145, MET165, LEU167, PRO168
Paxlovid	-5.185	-43.34	GLU166, ASN142	CYS44, MET49, PRO52, TYR54, PHE140, LEU141, GLY143, MET165, LEU167

**Table 7:** XP Gscore and MM-GBSA values between the Nsp3 (PDB ID: 7KQP) and the best hit phytochemical and control drugs.

Drug	XP Gscore (kcal Mol−1)	MM-GBSA scores (kcal Mol−1)	Hydrogen bonds	Hydrophobic bonds
Sulfuretin	-9.563	-52.85	ALA38, ASN40, GLY47, ALA50	ALA39, VAL49, PRO125, LEU126, LEU127, ALA129, ILE131, PHE132, PHE156
Molnupiravir	-7.604	-43.48	VAL49, ALA39, LEU126	ALA38, ALA39, PRO125, LEU126, LEU127, ALA129, ILE131, PHE132, PHE156
Paxlovid	-2.727	-43.34	GLY48, GLY130, LEU126	ALA38, VAL49, PRO125, LEU126, LEU127, ALA129, ILE131, VAL155, PHE156

**Table 8:** XP Gscore and MM-GBSA values between the human ACE2 receptor (PDB ID: 1R4L) and the best hit phytochemical and control drugs.

Drug	XP Gscore (kcal mol−1)	MM-GBSA scores (kcal mol−1)	Hydrogen bonds	Hydrophobic bonds
Grayanoside A	-7.87	-63.54	ARG273, HIS345, ALA348, GLN375	TYR127, LEU144, TRP271, PHE274, CYS344, PRO346, ALA348, PHE504, TYR510, TYR515
Molnupiravir	-6.02	-40.53	ALA348, GLN375, ARG514	PRO346, TRP349, PHE504, TYR510, TYR515
Paxlovid	-5.679	-32.02	ARG273, HIS345, ALA348, GLN375,	TYR127, LEU144, TRP271, PHE274, CYS344, PRO346, ALA348, PHE504, TYR510, TYR515

**Table 9:** Post MD MM-GBSA based binding free energy evaluation for main protease (3CLpro) (PDB ID: 7NT3) and the best hit phytochemical along with control drugs.

Name of complex	MM-GBSA (kcal/mol)	
	*Δ*G _Bind_	*Δ*G _Bind_ range
Lovastatin_7NT3	−52.56 ± 8.93	-61.49 to –43.63
Molnupiravir_7NT3	−50.52 ± 12.75	-63.27 to –37.77
Paxlovid_7NT3	−49.68 ± 16.27	-65.95 to –33.41

**Table 10:** Post MD MM-GBSA based binding free energy evaluation for Nsp3 (PDB ID: 7KQP) and the best hit phytochemical along with control drugs.

Name of complex	MM-GBSA (kcal/mol)	
	*Δ*G _Bind_	*Δ*G _Bind_ range
Sulfuretin_7KQP	−66.17 ± 11.62	-77.79 to –54.55
Molnupiravir_7KQP	−36.51 ± 13.74	-50.25 to –22.77
Paxlovid_7KQP	−54.30 ± 15.45	-81.85 to –33.15

**Table 11:** Post MD MM-GBSA based binding free energy evaluation for human ACE2 receptor (PDB ID: 1R4L) and the best hit phytochemical along with control drugs.

Name of complex	MM-GBSA (kcal/mol)	
	*Δ*G _Bind_	*Δ*G _Bind_ range
Grayanoside A_1R4L	−74.94 ± 8.50	-83.57 to –66.40
Molnupiravir_1R4L	−34.23 ± 12.82	-46.22 to –21.72
Paxlovid_1R4L	−57.50 ± 24.35	-81.85 to –33.15

## Data Availability

Availability of additional data (supplementary files) will be provided on request.

## Supplementary Materials

Supplementary 1 Supplementary Table 1: drug-like properties of all the selected phytochemicals.

Supplementary 2 Supplementary Table 2: ADMET properties of the selected phytochemicals.

Supplementary 3 Supplementary Table 3a: binding affinity of the main protease (PDB ID: 7NT3) and the phytochemicals and control drugs. Supplementary Table 3b: binding affinity of the NSP3 (PDB ID: 7KQP) and the phytochemicals and control drugs. Supplementary Table 3c: binding affinity of human ACE2 receptor(PDB ID: 1R4L) and phytochemicals.

Supplementary 4 Supplementary Table 4a: XP Gscore and MM-GBSA values between the main protease (PDB ID: 7NT3) and phytochemicals and control drugs. Supplementary Table 4b: XP Gscore and MM-GBSA values between the NSP3 (PDB ID: 7KQP) and the phytochemicals and control drugs. Supplementary Table 4c: XP Gscore and MM-GBSA values between the human ACE2 receptor (PDB ID: 1R4L) and phytochemicals and control drugs.

Supplementary 5 Supplementary Table 5a: coordinates of geometry-optimized ligand (Lovastatin) after molecular docking study. Supplementary Table 5b: coordinates of geometry-optimized ligand (Sulfuretin) after molecular docking study. Supplementary Table 5c: coordinates of geometry-optimized ligand (Grayanoside A) after molecular docking study.

Supplementary 6 Supplementary Table 6a: predicted biological activities of Lovastatin. Supplementary table 6b: predicted biological activities of Sulfuretin. Supplementary Table 6c: predicted biological activities of Grayanoside A.
