# Supplementary material for: In Silico Identification and Analysis of Potentially Bioactive Antiviral Phytochemicals against SARS-CoV-2: A Molecular Docking and Dynamics Simulation Approach
Source: Biomed Res Int. 2023 May 11;2023:5469258. doi: 10.1155/2023/5469258 (PMC10195178; doi:10.1155/2023/5469258)
Supplement: Supplementary 1 — Supplementary Table 1: drug-like properties of all the selected phytochemicals. [file 5469258.f1.docx]

**Supplementary table1** Drug-like properties of all the selected phytochemicals

| **Chemblmid** | **Canonical Smiles** | **MW** | **#Rotatable bonds** | **#H-bond acceptors** | **#H-bond donors** | **MR** | **TPSA** | **Consensus Log P** | **CYP1A2 inhibitor** | **CYP2C19 inhibitor** | **CYP2C9 inhibitor** | **CYP2D6 inhibitor** | **CYP3A4 inhibitor** | **Lipinski #violations** | **Ghose #violations** | **Veber #violations** | **Egan #violations** | **Muegge #violations** | **Bioavailability Score** |
| --- | --- | --- | --- | --- | --- | --- | --- | --- | --- | --- | --- | --- | --- | --- | --- | --- | --- | --- | --- |
| CHEMBL1229889 | BrCBr | 173.83 | 0 | 0 | 0 | 22.66 | 0 | 1.75 | No | No | No | No | No | 0 | 2 | 0 | 0 | 3 | 0.55 |
| CHEMBL1911631 | CCCCC/C=C/C=C/[C@@]12O[C@H]3[C@](O1)(C(=C)C)[C@@H]([C@H]([C@@]1(O2)[C@H]3[C@@H]2O[C@@]2([C@H]([C@]2([C@H]1C=C(C2=O)C)O)O)CO)C)OC(=O)C | 586.67 | 10 | 10 | 3 | 150.86 | 144.28 | 3.05 | No | No | No | No | No | 1 | 3 | 1 | 1 | 0 | 0.55 |
| CHEMBL190927 | O=Cc1ccc(cc1)C | 120.15 | 1 | 1 | 0 | 36.8 | 17.07 | 1.96 | Yes | No | No | No | No | 0 | 3 | 0 | 0 | 2 | 0.55 |
| CHEMBL1208793 | COc1cc(O)c2c(c1c1cc(ccc1O)c1cc(=O)c3c(o1)cc(cc3O)OC)oc(cc2=O)c1ccc(cc1)O | 566.51 | 5 | 10 | 4 | 155.91 | 159.8 | 4.4 | No | No | Yes | No | No | 1 | 3 | 1 | 1 | 2 | 0.55 |
| CHEMBL1170486 | CCCCCCCCCCCCCCCC(=O)O[C@H]1CC[C@]2([C@H](C1(C)C)CC[C@@]1([C@@H]2CC=C2[C@@]1(C)CC[C@@]1([C@@H]2CC(C)(C)CC1)C)C)C | 665.13 | 16 | 2 | 0 | 211.92 | 26.3 | 12.5 | No | No | No | No | No | 2 | 4 | 1 | 1 | 3 | 0.17 |
| CHEMBL1644933 | CC(=CCc1cc(ccc1O)/C=C/C(=O)c1ccc(c(c1O)CC=C(C)C)O)C | 392.49 | 7 | 4 | 3 | 119.76 | 77.76 | 5.32 | Yes | No | Yes | No | Yes | 0 | 1 | 0 | 0 | 1 | 0.55 |
| CHEMBL450533 | O[C@@H]1C[C@@]2(C)[C@H](C[C@@H]1O)CC[C@@H]1[C@@H]2[C@@H](O)C[C@]23[C@H]1C[C@H]1[C@@H]3[C@](OC2=O)(C)[C@]2(O1)C[C@@H](C(O2)(C)C)C | 490.63 | 0 | 7 | 3 | 128.45 | 105.45 | 2.83 | No | No | No | No | No | 0 | 2 | 0 | 0 | 0 | 0.55 |
| CHEMBL13045 | COc1c(OC)ccc2c1c[n+](C)c1c2ccc2c1cc1OCOc1c2 | 348.37 | 2 | 4 | 0 | 101.6 | 40.8 | 3.02 | Yes | Yes | Yes | No | Yes | 0 | 0 | 0 | 0 | 0 | 0.55 |
| CHEMBL459615 | OC[C@@H](C#CC#CC#CC#CCCCCCCCC(=O)O)O | 300.35 | 8 | 4 | 3 | 85.38 | 77.76 | 2.72 | Yes | No | No | No | No | 0 | 0 | 0 | 0 | 0 | 0.56 |
| CHEMBL1080979 | COc1ccc2c(c1)O[C@@H](CC2)c1ccc(cc1)O | 256.3 | 2 | 3 | 1 | 73.59 | 38.69 | 3.02 | Yes | No | No | Yes | No | 0 | 0 | 0 | 0 | 0 | 0.55 |
| CHEMBL458391 | C[C@@H]([C@H](c1c(O)c2oc3cc(=O)c(c4c3c(c2c(c1C)O)O[C@@H]([C@H]4C)C)C)C)O | 398.45 | 2 | 6 | 3 | 112.59 | 100.13 | 3.35 | No | No | No | No | Yes | 0 | 0 | 0 | 0 | 0 | 0.55 |
| CHEMBL462951 | CN1CCc2c3C1=CC(=O)C(=O)c3[nH]c2 | 202.21 | 0 | 2 | 1 | 58.44 | 53.17 | 0.66 | Yes | No | No | No | No | 0 | 0 | 0 | 0 | 0 | 0.55 |
| CHEMBL2071229 | COc1ccc(c2c1cc(C)cc2O)OC | 218.25 | 2 | 3 | 1 | 63.92 | 38.69 | 2.76 | Yes | No | No | No | No | 0 | 0 | 0 | 0 | 0 | 0.55 |
| CHEMBL465407 | CC(=CCc1c(O)cc2c(c1O)c(=O)c1c(o2)c(O)cc(c1CC=C(C)C)O)C | 396.43 | 4 | 6 | 4 | 115.52 | 111.13 | 4.23 | Yes | No | Yes | No | No | 0 | 0 | 0 | 0 | 1 | 0.55 |
| CHEMBL1087928 | COc1ccc(cc1)c1c(C)c(C)cc2c1cc1OCOc1c2 | 306.36 | 2 | 3 | 0 | 91.87 | 27.69 | 4.57 | Yes | Yes | Yes | No | No | 0 | 0 | 0 | 0 | 1 | 0.55 |
| CHEMBL557704 | COc1ccc(c(c1CC=C(C)C)OC)[C@]1(O)COc2c(C1=O)c(O)cc(c2CC=C(C)C)O | 468.54 | 7 | 7 | 3 | 131.02 | 105.45 | 4.48 | No | No | No | No | Yes | 0 | 1 | 0 | 0 | 1 | 0.55 |
| CHEMBL1164833 | CC[C@H]1CCC[C@@]([C@@H]2[C@H]1[C@@H](C)C=C2)(C)CO | 222.37 | 2 | 1 | 1 | 70.42 | 20.23 | 3.52 | No | Yes | Yes | No | No | 0 | 0 | 0 | 0 | 1 | 0.55 |
| CHEMBL470467 | OC[C@H]1O[C@@H](OC[C@H]2NC[C@H]([C@@H]2O)O)[C@@H]([C@H]([C@@H]1O)O)O | 295.29 | 4 | 9 | 7 | 66.62 | 151.87 | -3.42 | No | No | No | No | No | 1 | 1 | 1 | 1 | 3 | 0.55 |
| CHEMBL1904496 | C1Oc2c(O1)cc(cc2)C1OCC2C1COC2c1ccc2c(c1)OCO2 | 354.35 | 2 | 6 | 0 | 90 | 55.38 | 2.79 | No | Yes | No | Yes | Yes | 0 | 0 | 0 | 0 | 0 | 0.55 |
| CHEMBL1651023 | CCO[C@@H]1OC[C@@]2([C@H]3[C@]1(CCC2)c1cc(O)c(cc1C(=O)C3)C(C)C)C | 358.47 | 3 | 4 | 1 | 101.73 | 55.76 | 3.99 | No | No | No | Yes | No | 0 | 0 | 0 | 0 | 0 | 0.55 |
| CHEMBL422711 | Oc1cc(O)c2c(c1)O[C@@H]([C@@H]([C@H]2c1c(O)cc(c2c1O[C@@H]([C@@H](C2)OC(=O)c1cc(O)c(c(c1)O)O)c1cc(O)c(c(c1)O)O)O)OC(=O)c1cc(O)c(c(c1)O)O)c1cc(O)c(c(c1)O)O | 914.73 | 9 | 22 | 16 | 222.17 | 394.74 | 1.59 | No | No | No | No | No | 3 | 3 | 1 | 1 | 5 | 0.17 |
| CHEMBL465475 | CO[C@@]12CC[C@]3([C@@](C1)(C(=O)C2=C)CC[C@H]1[C@]23CCC[C@]1(C(=O)O2)C)C | 344.44 | 1 | 4 | 0 | 93.88 | 52.6 | 3.39 | No | No | No | No | No | 0 | 0 | 0 | 0 | 0 | 0.55 |
| CHEMBL517700 | COc1ccc(c(c1)O)c1coc2c(c1=O)cc(c(c2)O)OC | 314.29 | 3 | 6 | 2 | 84.95 | 89.13 | 2.3 | Yes | No | Yes | Yes | Yes | 0 | 0 | 0 | 0 | 0 | 0.55 |
| CHEMBL508185 | CC(=O)OC(/C=C/C(=O)[C@@]([C@H]1[C@H](O)C[C@@]2([C@]1(C)CC(=O)[C@@]1([C@H]2CC=C2[C@H]1C[C@H](O)C(=O)C2(C)C)C)C)(O)C)(C)C | 558.7 | 6 | 8 | 3 | 150.94 | 138.2 | 3.23 | No | No | No | No | Yes | 1 | 3 | 0 | 1 | 0 | 0.55 |
| CHEMBL517213 | CC(=CCc1c(O)cccc1C(=O)c1cc2C=CC(Oc2cc1O)(C)C)C | 364.43 | 4 | 4 | 2 | 108.2 | 66.76 | 4.53 | Yes | Yes | Yes | No | Yes | 0 | 0 | 0 | 0 | 1 | 0.55 |
| CHEMBL1689089 | COC(=O)[C@@H]([C@H]1CC[C@@H]2[C@]1(C)CC[C@H]1[C@H]2CC[C@@H]2[C@]1(C)C=CC(=O)C2)CCCC(C)C | 428.65 | 7 | 3 | 0 | 128.75 | 43.37 | 6.15 | No | No | Yes | No | No | 1 | 2 | 0 | 1 | 1 | 0.55 |
| CHEMBL479791 | Cc1cnccn1 | 94.11 | 0 | 2 | 0 | 27 | 25.78 | 0.62 | No | No | No | No | No | 0 | 3 | 0 | 0 | 1 | 0.55 |
| CHEMBL2334478 | O[C@H]1C[C@@H]2C[C@]3([C@@H]1[C@]1(C)CC[C@@H](C([C@H]1C(=O)C3)(C)C)O)[C@@H](C2=C)O | 334.45 | 0 | 4 | 3 | 92.23 | 77.76 | 2.04 | No | No | No | No | No | 0 | 0 | 0 | 0 | 0 | 0.55 |
| CHEMBL540458 | O=C[C@]1(C)[C@H](CC[C@]2([C@H]1CC[C@@]1([C@@H]2CC=C2[C@@]1(C)CC[C@@]1([C@H]2CC(CC1)(C)C)C(=O)O)C)C)O[C@@H]1OC[C@@H]([C@@H]([C@H]1O)O)O | 602.8 | 4 | 8 | 4 | 163.27 | 133.52 | 3.97 | No | No | No | No | No | 1 | 3 | 0 | 1 | 1 | 0.56 |
| CHEMBL482045 | OC[C@H]1O[C@@H](Oc2ccc(cc2O)/C=C/C(=O)OC)[C@@H]([C@H]([C@@H]1O)O)O | 356.32 | 6 | 9 | 5 | 83.6 | 145.91 | -0.41 | No | No | No | No | No | 0 | 1 | 1 | 1 | 0 | 0.55 |
| CHEMBL502427 | O=C1O[C@@H]2C[C@@]3([C@H]1CO[C@]14C(=O)[C@@H]3[C@@]3([C@@]2(C)OC(=O)[C@]3(O)CC[C@H]2[C@H]4C[C@@H](O)[C@@]3([C@]2(C)C(=O)C=CC3)O)O1)C | 544.55 | 0 | 11 | 3 | 127.62 | 165.89 | 0.01 | No | No | No | No | No | 2 | 2 | 1 | 1 | 3 | 0.17 |
| CHEMBL518106 | COC(=O)CC[C@@]1(C)[C@@H](CC=C2[C@H]1CC[C@]1([C@@]2(C)CC[C@@H]1[C@@H](C[C@H]1OC(=O)[C@@H](C1)C)C)C)C(=C)C | 484.71 | 8 | 4 | 0 | 143.52 | 52.6 | 6.65 | No | No | Yes | No | No | 1 | 4 | 0 | 1 | 1 | 0.55 |
| CHEMBL254956 | O=Cc1cc(Br)c(c(c1)Br)O | 279.91 | 1 | 2 | 1 | 49.25 | 37.3 | 2.5 | Yes | No | Yes | No | No | 0 | 1 | 0 | 0 | 0 | 0.55 |
| CHEMBL463558 | CO[C@@H]1O[C@@H](c2c1c([C@H](C(=O)O)C)c(cc2)[C@@]1(C)CCCC(C1)(C)C)OC | 376.49 | 5 | 5 | 1 | 104.41 | 64.99 | 4.16 | No | No | Yes | Yes | Yes | 0 | 0 | 0 | 0 | 0 | 0.85 |
| CHEMBL1651069 | CO[C@@H]1OC[C@@]2([C@H]3[C@]1(CCC2)c1cc(O)c(cc1CC3)C(C)C)C | 330.46 | 2 | 3 | 1 | 96.5 | 38.69 | 4.3 | No | No | Yes | Yes | No | 0 | 0 | 0 | 0 | 0 | 0.55 |
| CHEMBL505172 | C=C/C(=C/C[C@]1(C)[C@H](C)CC[C@@]23[C@H]1C[C@@H](OC(=O)c1ccc(c(c1)OC)O)C=C3[C@H](O[C@H]2OC(=O)C)OC(=O)C)/C | 568.65 | 11 | 9 | 1 | 152.17 | 117.59 | 4.87 | No | No | No | No | Yes | 1 | 4 | 1 | 0 | 1 | 0.55 |
| CHEMBL3105546 | COc1cc(ccc1O[C@@H]([C@@H](c1ccc(c(c1)OC)OC)O)C)C(=O)[C@@H]([C@@H](C(=O)c1ccc(c(c1)OC)O[C@@H]([C@@H](c1ccc2c(c1)OCO2)O)C)C)C | 730.8 | 17 | 12 | 2 | 196.31 | 148.44 | 5.47 | No | Yes | No | No | No | 2 | 4 | 2 | 2 | 4 | 0.17 |
| CHEMBL3184678 | CC(=C)C1CCC(CC1)(C)O | 154.25 | 1 | 1 | 1 | 48.8 | 20.23 | 2.44 | No | No | No | No | No | 0 | 1 | 0 | 0 | 2 | 0.55 |
| CHEMBL1996175 | CCCCCCCCCCC([C@H]1CCC(O1)C1CC[C@@H](O1)[C@@H](CCCCCCCCCCC(CC1=CC(OC1=O)C)O)O)O | 622.92 | 25 | 7 | 3 | 180.1 | 105.45 | 7.6 | No | No | No | No | Yes | 1 | 4 | 1 | 1 | 3 | 0.55 |
| CHEMBL1778158 | COc1cc(cc(c1OC)OC)[C@H]1[C@H]2C(=O)OC[C@@H]2[C@H](c2c1cc1OCOc1c2)O[C@@H]1O[C@H](COC(=O)C)[C@H]([C@@H]([C@H]1O)O)O | 618.58 | 9 | 14 | 3 | 145.97 | 177.9 | 0.96 | No | No | No | Yes | No | 2 | 3 | 1 | 1 | 3 | 0.17 |
| CHEMBL445810 | COc1cc(ccc1O)C=C(/C(=C\c1ccc(c(c1)OC)O)/C(=NCCc1ccc(cc1)O)O)C(=NCCc1ccc(cc1)O)O | 624.68 | 13 | 10 | 6 | 180.36 | 164.56 | 5.23 | No | No | No | No | No | 2 | 4 | 2 | 2 | 4 | 0.17 |
| CHEMBL2335721 | Oc1ccc(cc1)[C@H]1Oc2cc(O)cc(c2[C@@H](C1)c1c(O)cc(c2c1O[C@H](c1ccc(cc1)O)[C@@H](C2)O)O)O | 530.52 | 3 | 9 | 7 | 141.5 | 160.07 | 2.94 | No | No | No | No | Yes | 2 | 2 | 1 | 1 | 2 | 0.17 |
| CHEMBL517308 | C/C=C(\C(=O)O[C@H]1[C@H](O)[C@H]2C(=C)C(=O)O[C@@H]2[C@@]2(C(=C1C)C(=O)C=C2C)O)/C | 374.38 | 3 | 7 | 2 | 95.15 | 110.13 | 1.22 | No | No | No | No | No | 0 | 0 | 0 | 0 | 0 | 0.55 |
| CHEMBL3287734 | O[C@@H]1CCc2c([C@@]1(C)O)ccc1c2C(=O)C(=O)c2c1occ2C | 312.32 | 0 | 5 | 2 | 82.22 | 87.74 | 1.82 | Yes | No | No | No | No | 0 | 0 | 0 | 0 | 0 | 0.55 |
| CHEMBL1765403 | CCOC(=O)C[C@H](c1ccc(cc1)O)N=C([C@H](N(C(=O)[C@@H](N=C([C@H](C/C(=C/[C@@H](C[C@@H](O)C)C)/C)C)O)C)C)Cc1c(Br)[nH]c2c1cccc2)O | 755.74 | 19 | 9 | 5 | 203.62 | 168.04 | 6.03 | No | No | No | No | Yes | 2 | 4 | 2 | 2 | 4 | 0.17 |
| CHEMBL424 | OC(=O)c1ccccc1O | 138.12 | 1 | 3 | 2 | 35.42 | 57.53 | 1.24 | No | No | No | No | No | 0 | 3 | 0 | 0 | 1 | 0.85 |
| CHEMBL446760 | OC[C@H]1O[C@@H](OCCc2ccc(c(c2)O)O)[C@@H]([C@H]([C@@H]1O)OC(=O)Cc1ccc(c(c1)O)O)O | 466.44 | 9 | 11 | 7 | 112.08 | 186.37 | 0.21 | No | No | No | No | No | 2 | 0 | 1 | 1 | 3 | 0.17 |
| CHEMBL575533 | COC1=C[C@@H]2CCN3[C@@H]2[C@@H]([C@@H]1O)c1cc2OCOc2cc1C3 | 301.34 | 1 | 5 | 1 | 83.13 | 51.16 | 1.56 | No | No | No | Yes | No | 0 | 0 | 0 | 0 | 0 | 0.55 |
| CHEMBL485988 | O=CO[C@H]1CC[C@]2([C@H](C1(C)C)CC[C@@]1([C@@H]2C=C[C@]23[C@@]1(C)CC[C@@]1([C@H]3[C@@H](C)[C@H](C)CC1)C(=O)O2)C)C | 482.69 | 2 | 4 | 0 | 139.67 | 52.6 | 6.08 | No | No | No | No | No | 1 | 4 | 0 | 1 | 1 | 0.55 |
| CHEMBL512443 | COc1c(O)cc2c3c1c1ccccc1cc3n(c2=O)C | 279.29 | 1 | 3 | 1 | 84.18 | 51.46 | 2.9 | Yes | No | No | Yes | No | 0 | 0 | 0 | 0 | 0 | 0.55 |
| CHEMBL1357750 | O[C@H]1C[C@]2(C)[C@@H]([C@@]3([C@@H]1C(C)(C)[C@@H](O)CC3)C)C[C@H]([C@H]1[C@@]2(C)CC[C@@H]1[C@]1(C)CCCC(O1)(C)C)O | 476.73 | 1 | 4 | 3 | 139.36 | 69.92 | 4.99 | No | No | No | No | No | 1 | 3 | 0 | 0 | 1 | 0.55 |
| CHEMBL1224728 | CC(=O)O[C@H]1CC[C@]2([C@H](C1(C)C)CC[C@]1([C@H]2CC(=O)/C/1=C(\C=C[C@H]1[C@@H](C[C@H]([C@]1(C)O)O)C(=C)C)/C)C)C | 512.72 | 5 | 5 | 2 | 149.13 | 83.83 | 5.39 | No | No | No | No | Yes | 2 | 4 | 0 | 1 | 1 | 0.17 |
| CHEMBL513565 | COc1cc(ccc1O)C[C@H]1C(=O)OC[C@@H]1[C@@H](c1ccc(c(c1)OC)O)O | 374.38 | 6 | 7 | 3 | 97.29 | 105.45 | 2.1 | No | No | No | No | No | 0 | 0 | 0 | 0 | 0 | 0.55 |
| CHEMBL2023564 | CC(=CCC[C@@]([C@H]1CCC(=CC1)C)(O[C@@H]1O[C@H](C)[C@@H]([C@@H]([C@H]1O)O)O)C)C | 368.51 | 6 | 5 | 3 | 103.58 | 79.15 | 2.68 | No | No | No | No | No | 0 | 0 | 0 | 0 | 0 | 0.55 |
| CHEMBL3039223 | CC(=C)[C@@H]1CCC(=CC1)C(=O)O | 166.22 | 2 | 2 | 1 | 48.89 | 37.3 | 2.27 | No | No | No | No | No | 0 | 0 | 0 | 0 | 1 | 0.85 |
| CHEMBL449905 | NC1=CC2=NCCc3c2c(C1=O)n(C)c3 | 201.22 | 0 | 2 | 1 | 60.45 | 60.38 | 0.58 | Yes | No | No | No | No | 0 | 0 | 0 | 0 | 0 | 0.55 |
| CHEMBL1396912 | COc1ccc2c(c1)[nH]c1c2CCN2[C@H]1C[C@@H]1[C@H](C2)C[C@@H]([C@H]([C@@H]1C(=O)OC)OC)OC(=O)c1cc(OC)c(c(c1)OC)OC | 608.68 | 10 | 10 | 1 | 165.52 | 117.78 | 3.51 | No | No | No | No | No | 2 | 3 | 0 | 0 | 1 | 0.17 |
| CHEMBL1200861 | O[C@@H]([C@@H](C(=O)O)O)C(=O)O | 150.09 | 3 | 6 | 4 | 27.21 | 115.06 | -1.78 | No | No | No | No | No | 0 | 4 | 0 | 0 | 2 | 0.56 |
| CHEMBL453563 | O=C(C(O)(C)C)CCc1c(C)ccc2c1C(=O)C(=O)C(=C2)C(C)C | 328.4 | 5 | 4 | 1 | 94.6 | 71.44 | 3.02 | No | Yes | No | No | No | 0 | 0 | 0 | 0 | 0 | 0.55 |
| CHEMBL362076 | Cc1ccc(c2c1cccc2)C | 156.22 | 0 | 0 | 0 | 53.88 | 0 | 3.8 | Yes | Yes | No | No | No | 1 | 1 | 0 | 0 | 2 | 0.55 |
| CHEMBL3087308 | CC[C@@H]([C@@H](c1oc([C@@H]([C@@H]([C@H]([C@H](/C(=C/[C@H](c2oc(CC)c(c(=O)c2C)C)C)/C)OC(=O)C)C)OC(=O)CC(C)C)C)c(c(=O)c1C)C)C)OC(=O)C | 712.91 | 19 | 10 | 0 | 201.51 | 139.32 | 7.17 | No | No | No | No | No | 1 | 4 | 1 | 2 | 3 | 0.55 |
| CHEMBL469537 | O=CC1=CCC(CC1)C(=C)C | 150.22 | 2 | 1 | 0 | 47.32 | 17.07 | 2.5 | No | No | No | No | No | 0 | 1 | 0 | 0 | 2 | 0.55 |
| CHEMBL476873 | Br/C=C/C#C/C=C/CCCC/C=C/C#CCCCC(=O)O | 349.26 | 8 | 2 | 1 | 93.18 | 37.3 | 4.9 | Yes | Yes | Yes | No | No | 1 | 0 | 0 | 0 | 1 | 0.85 |
| CHEMBL3590541 | O[C@@H]1C=CC(=O)N2[C@H]1[C@H]1CCCN3[C@H]1[C@H](C2)CCC3 | 262.35 | 0 | 3 | 1 | 80.27 | 43.78 | 0.87 | No | No | No | No | No | 0 | 0 | 0 | 0 | 0 | 0.55 |
| CHEMBL219459 | OC[C@H]1O[C@@H](Oc2ccc(c(c2)O)c2c(=O)oc3c(c2O)ccc(c3)O)[C@@H]([C@H]([C@@H]1O)O)O | 448.38 | 4 | 11 | 7 | 108.13 | 190.28 | 0.02 | No | No | No | No | No | 2 | 0 | 1 | 1 | 3 | 0.17 |
| CHEMBL1170899 | O=Cc1c(C=O)cc(c2c1O[C@]1(C2)[C@H](C)CC[C@@H]2[C@]1(C)CC[C@H](C2(C)C)O)O | 386.48 | 2 | 5 | 2 | 107.53 | 83.83 | 3.32 | No | No | No | No | Yes | 0 | 0 | 0 | 0 | 0 | 0.55 |
| CHEMBL2332142 | COc1ccc2c(c1)N(C)C(=O)[C@]12C[C@@H]2N[C@H]1C[C@@H]1[C@H]2COC=C1C(=O)C | 368.43 | 2 | 5 | 1 | 107.07 | 67.87 | 1.59 | No | No | No | Yes | No | 0 | 0 | 0 | 0 | 0 | 0.85 |
| CHEMBL412890 | OCCc1ccc(cc1)OC | 152.19 | 3 | 2 | 1 | 43.87 | 29.46 | 1.49 | Yes | No | No | No | No | 0 | 1 | 0 | 0 | 1 | 0.55 |
| CHEMBL448125 | CC1=CCCC(=CCC(=C(C)C)CC1)C | 204.35 | 0 | 0 | 0 | 70.68 | 0 | 4.6 | No | No | Yes | No | No | 1 | 0 | 0 | 0 | 2 | 0.55 |
| CHEMBL484231 | CC1=C[C@H]2OC3[C@@]4([C@H]1CC(=O)c1cocc1)[C@@H]2[C@](C)(CCC4)C(=O)O3 | 342.39 | 3 | 5 | 0 | 89.12 | 65.74 | 2.82 | No | No | No | No | Yes | 0 | 0 | 0 | 0 | 0 | 0.55 |
| CHEMBL172350 | COc1c(O)cc2c(c1O)c(=O)cc(o2)c1ccc(c(c1)O)O | 316.26 | 2 | 7 | 4 | 82.5 | 120.36 | 1.74 | Yes | No | No | Yes | Yes | 0 | 0 | 0 | 0 | 0 | 0.55 |
| CHEMBL1773759 | CO[C@@H]1OCC2=CC[C@@H]3[C@]([C@@H]12)(C)[C@H](O)C[C@H]1[C@@]3(C)CC[C@@H]2[C@]1(C)CCCC2(C)C | 402.61 | 1 | 3 | 1 | 118.34 | 38.69 | 4.94 | No | No | No | No | No | 1 | 1 | 0 | 0 | 1 | 0.55 |
| CHEMBL2385627 | C=C[C@@]1(C)C[C@@H](O)[C@H]2C(=C1)CC[C@@H]1[C@]2(C)CCC[C@@]1(C)COC(=O)C | 346.5 | 4 | 3 | 1 | 102.24 | 46.53 | 4.41 | No | No | Yes | No | Yes | 0 | 0 | 0 | 0 | 0 | 0.55 |
| CHEMBL2334473 | O=CC(=C)[C@H]1C[C@@H](OC(=O)C)[C@H]2[C@](C1)(O)[C@H](OC(=O)C)C(=O)[C@@H]1[C@]2(C)[C@H](OC(=O)C)C[C@H](C1(C)C)O | 508.56 | 8 | 10 | 2 | 126.38 | 153.5 | 1.56 | No | No | No | No | No | 1 | 2 | 1 | 1 | 1 | 0.55 |
| CHEMBL1811940 | CC[C@@H](C(=O)O[C@@H]1CC[C@@]2([C@]3([C@H]1[C@@](C)(C[C@@H](C1=CC(=O)OC1)OC(=O)[C@H](CC)C)[C@H](C)C[C@@H]3OC(=O)C)COC(=O)C)CO2)C | 634.75 | 16 | 11 | 0 | 163.65 | 144.03 | 4.48 | No | Yes | No | No | No | 2 | 3 | 2 | 1 | 3 | 0.17 |
| CHEMBL451784 | CC(=O)O[C@H]1CC[C@H]([C@@]23[C@]1(C)[C@H](C[C@H]([C@H]3O)C(O2)(C)C)OC(=O)c1ccccc1)C | 416.51 | 5 | 6 | 1 | 111.64 | 82.06 | 3.36 | No | Yes | No | Yes | Yes | 0 | 0 | 0 | 0 | 0 | 0.55 |
| CHEMBL483030 | COc1cc(O)c2c(c1)oc(c(c2=O)OC)c1cc(O)c(cc1OC)OC | 374.34 | 5 | 8 | 2 | 97.93 | 107.59 | 2.52 | Yes | No | Yes | No | Yes | 0 | 0 | 0 | 0 | 0 | 0.55 |
| CHEMBL517324 | O=C1CC[C@@H]2[C@]([C@H]1C)(C)CC[C@H]1[C@@]2(C)CC[C@@]2([C@]1(C)CC[C@@]1([C@H]2CC(C)(C)CC1)C(=O)O)C | 456.7 | 1 | 3 | 1 | 136.17 | 54.37 | 6.35 | No | No | No | No | No | 1 | 3 | 0 | 1 | 1 | 0.85 |
| CHEMBL443402 | CCCCCCCCCCCC[C@H]([C@H]1CC[C@@H](O1)[C@@H](CCCCCCCCCCC[C@@H](CCC1=C[C@@H](OC1=O)C)O)O)O | 608.93 | 28 | 6 | 3 | 181.13 | 96.22 | 8.6 | No | No | No | No | No | 2 | 4 | 1 | 1 | 3 | 0.17 |
| CHEMBL3109429 | OC[C@H]1O[C@@H](Oc2c(O)cc(cc2[C@@H]([C@H](c2c(O)cc(c(c2/C=C/c2ccc(cc2)O)O[C@@H]2O[C@H](CO)[C@H]([C@@H]([C@H]2O)O)O)O)c2ccc(cc2)O)O)O)[C@@H]([C@H]([C@@H]1O)O)O | 828.77 | 12 | 19 | 15 | 202.15 | 340.37 | -0.92 | No | No | No | No | No | 3 | 4 | 2 | 1 | 4 | 0.17 |
| CHEMBL2392323 | CO[C@@H]1O[C@H](C[C@H]1[C@@H]1CC[C@]2([C@@]1(C)CC[C@H]1C2=CC[C@@H]2[C@]1(C)CCC(=O)C2(C)C)C)[C@@H](C(O)(C)C)O | 502.73 | 4 | 5 | 2 | 143.78 | 75.99 | 4.91 | No | No | No | No | No | 1 | 4 | 0 | 0 | 1 | 0.55 |
| CHEMBL463312 | OC[C@H]1O[C@H]([C@@H]([C@H]([C@@H]1O)O)O)c1c(O)cc2c(c1O)c(=O)cc(o2)C | 354.31 | 2 | 9 | 6 | 84.12 | 160.82 | -0.54 | No | No | No | No | No | 1 | 1 | 1 | 1 | 2 | 0.55 |
| CHEMBL513024 | COc1cc2c(cc1O)occ(c2=O)c1ccc(cc1)O | 284.26 | 2 | 5 | 2 | 78.46 | 79.9 | 2.3 | Yes | No | No | Yes | Yes | 0 | 0 | 0 | 0 | 0 | 0.55 |
| CHEMBL14060 | Oc1ccccc1 | 94.11 | 0 | 1 | 1 | 28.46 | 20.23 | 1.41 | Yes | No | No | No | No | 0 | 3 | 0 | 0 | 2 | 0.55 |
| CHEMBL504766 | COc1cc(OC)cc2c1c(O)c1c(c2)oc(c(c1=O)C)C | 300.31 | 2 | 5 | 1 | 84.93 | 68.9 | 3.04 | Yes | Yes | Yes | Yes | Yes | 0 | 0 | 0 | 0 | 0 | 0.55 |
| CHEMBL1345092 | C/C=C(\C(=O)O[C@@H]1[C@H](OC(=O)C=C(C)C)c2c(OC1(C)C)ccc1c2oc(=O)cc1)/C | 426.46 | 6 | 7 | 0 | 115.98 | 92.04 | 3.98 | No | Yes | Yes | No | Yes | 0 | 0 | 0 | 0 | 0 | 0.55 |
| CHEMBL45354 | CC(CCC(=O)C)C | 114.19 | 3 | 1 | 0 | 35.96 | 17.07 | 1.87 | No | No | No | No | No | 0 | 2 | 0 | 0 | 2 | 0.55 |
| CHEMBL450121 | OC[C@H]1O[C@@H](OCCc2ccc(c(c2)O)O)[C@@H]([C@H]([C@@H]1OC(=O)/C=C/c1ccc(c(c1)OC)O)O[C@@H]1O[C@@H](C)[C@@H]([C@H]([C@H]1O)O)O)O | 638.61 | 12 | 15 | 8 | 152.89 | 234.29 | -0.12 | No | No | No | No | No | 3 | 4 | 2 | 1 | 4 | 0.17 |
| CHEMBL2057716 | OCC1=C2C(=C3CC(=O)[C@@H]([C@]3(CC[C@@]2(CC[C@@H]1O)C)C)C(C)C)O | 334.45 | 2 | 4 | 3 | 94.54 | 77.76 | 2.64 | No | No | No | No | No | 0 | 0 | 0 | 0 | 0 | 0.55 |
| CHEMBL3754295 | O=C1C2=C(O[C@]3([C@@H](C2)[C@@H](CC3)C(O)(C)C)C)[C@@H]2C[C@@]1(O)CO2 | 308.37 | 1 | 5 | 2 | 79.71 | 75.99 | 1.33 | No | No | No | No | No | 0 | 0 | 0 | 0 | 0 | 0.56 |
| CHEMBL3577255 | C=C[C@@](/C=C/c1ccc(cc1)O)(CC[C@H](C(O)(C)C)O)C | 290.4 | 7 | 3 | 3 | 88.25 | 60.69 | 3.34 | Yes | No | No | Yes | No | 0 | 0 | 0 | 0 | 0 | 0.55 |
| CHEMBL3609100 | OC[C@H]1O[C@@H](Oc2c(oc3c(c2=O)c(O)c(c(c3)O)OC)c2ccc(c(c2)OC)O)[C@@H]([C@H]([C@@H]1O)O)O | 508.43 | 6 | 13 | 7 | 121.12 | 208.74 | 0.1 | No | No | No | No | No | 3 | 1 | 1 | 1 | 3 | 0.17 |
| CHEMBL445435 | C=C[C@H]1[C@@H](OC(=O)c2ccccc2)[C@H]2[C@@](C(=O)[C@@]1(C)O)(O)[C@H](OC(=O)C)C[C@@H]1[C@]2(C)C=C(OC(=O)C)C(=O)C1(C)C | 568.61 | 8 | 10 | 2 | 145.84 | 153.5 | 2.81 | No | No | No | No | Yes | 1 | 3 | 1 | 1 | 1 | 0.55 |
| CHEMBL456138 | C=C[C@H]1[C@@H](OC=C2[C@@]1(O)CCOC2=O)O[C@@H]1O[C@H](CO)[C@H]([C@@H]([C@H]1O)O)O | 374.34 | 4 | 10 | 5 | 82.12 | 155.14 | -1.32 | No | No | No | No | No | 0 | 1 | 1 | 1 | 1 | 0.11 |
| CHEMBL564585 | COC(=O)[C@@]12CC(=O)[C@@H](CC(=O)/C(=C/CC/C(=C/C(=O)[C@H]2CC(=C2[C@@H]1/C=C(\C)/CC[C@H](O)[C@]1(O[C@@H]([C@@]([C@H](C2)O)(C)O)CC1)C)C)/C)/C)C(C)C | 696.91 | 3 | 9 | 3 | 195.12 | 147.43 | 4.36 | No | No | No | No | Yes | 1 | 4 | 1 | 2 | 1 | 0.55 |
| CHEMBL471071 | C/C/1=C/C(=O)O[C@@H]2C[C@@H](CC[C@@H](C=CCC1)C)O[C@@](C2)(O)[C@H]1CSC(=N1)O | 395.51 | 1 | 6 | 2 | 110.71 | 113.65 | 2.77 | No | No | No | No | No | 0 | 0 | 0 | 0 | 0 | 0.55 |
| CHEMBL516550 | CC(=CCc1c2OC(C)(C)C=Cc2c(c2c1oc1c3c(O)cc(c4c3C(Cc1c2=O)C(O4)(C)C)O)O)C | 502.56 | 2 | 7 | 3 | 143.68 | 109.36 | 5.08 | No | Yes | Yes | No | No | 1 | 3 | 0 | 1 | 1 | 0.55 |
| CHEMBL454428 | OC[C@H]1O[C@@H](Oc2cccc3c2C(=O)c2c(C3=O)cc(cc2O)CO)[C@@H]([C@H]([C@@H]1O)O)O | 432.38 | 4 | 10 | 6 | 102.05 | 173.98 | -0.28 | No | No | No | No | No | 1 | 1 | 1 | 1 | 2 | 0.55 |
| CHEMBL1079685 | C=C1CC[C@@H]([C@@]2([C@@H]1C[C@H](CC2)C(O)(C)C)C)O | 238.37 | 1 | 2 | 2 | 71.62 | 40.46 | 2.69 | No | No | No | No | No | 0 | 0 | 0 | 0 | 0 | 0.55 |
| CHEMBL1082104 | O=Cc1ccc([nH]1)CCCCCCCCCCCCCC(C)C | 319.52 | 15 | 1 | 1 | 103.25 | 32.86 | 6.27 | Yes | Yes | No | Yes | No | 0 | 1 | 1 | 1 | 1 | 0.55 |
| CHEMBL511568 | CO[C@@H]1OC[C@]2([C@@H]1[C@]1(CCC2)COC(=O)[C@]23[C@H]1CC[C@H](C2)[C@@H](C3=O)C)C | 362.46 | 1 | 5 | 0 | 95.37 | 61.83 | 3.04 | No | No | No | No | No | 0 | 0 | 0 | 0 | 0 | 0.55 |
| CHEMBL425723 | OC[C@@H](c1ccccc1)NC[C@H]1NC[C@@H]([C@@H]1O)O | 252.31 | 5 | 5 | 5 | 71.14 | 84.75 | -0.17 | Yes | No | No | No | No | 0 | 1 | 0 | 0 | 0 | 0.55 |
| CHEMBL3181995 | CCCC=C(C=O)CC | 126.2 | 4 | 1 | 0 | 40.3 | 17.07 | 2.18 | No | No | No | No | No | 0 | 1 | 0 | 0 | 2 | 0.55 |
| CHEMBL480873 | N=c1[nH]cc(c2c1cncc2)c1ccccc1 | 221.26 | 1 | 2 | 2 | 68.06 | 52.53 | 2.33 | Yes | No | No | No | No | 0 | 0 | 0 | 0 | 0 | 0.55 |
| CHEMBL15605 | c1nccs1 | 85.13 | 0 | 1 | 0 | 22.11 | 41.13 | 0.73 | No | No | No | No | No | 0 | 3 | 0 | 0 | 2 | 0.55 |
| CHEMBL3397954 | OC(=O)[C@H]1O[C@@H](Oc2c(O)cc(c3c2oc(cc3=O)c2ccccc2)O)[C@@H]([C@H]([C@@H]1O)O)O | 446.36 | 4 | 11 | 6 | 106.72 | 187.12 | 0.23 | No | No | No | No | No | 2 | 0 | 1 | 1 | 3 | 0.11 |
| CHEMBL1814385 | OC1=NC2=C(C)C(=O)c3c(C2=O)cc(c(c3C(=O)/C(=C/[C@H](C)[C@@H]([C@H]([C@H]([C@@H]([C@@H]([C@@H]([C@H]([C@H](/C=C/C=C1)C)O)C)O)C)O)C)O)/C)O)C | 623.73 | 0 | 10 | 6 | 176.87 | 184.95 | 2.99 | No | No | No | No | Yes | 2 | 3 | 1 | 1 | 3 | 0.17 |
| CHEMBL480677 | O[C@H]1C[C@H]2[C@]3(C)[C@@H](O)CCC([C@H]3C[C@H]([C@]32[C@@H]([C@@H]1C(=C)C3=O)O)O)(C)C | 350.45 | 0 | 5 | 4 | 93.39 | 97.99 | 1.33 | No | No | No | No | No | 0 | 0 | 0 | 0 | 0 | 0.55 |
| CHEMBL601 | NCC(=O)CCC(=O)O | 131.13 | 4 | 4 | 2 | 30.83 | 80.39 | -1.15 | No | No | No | No | No | 0 | 4 | 0 | 0 | 2 | 0.55 |
| CHEMBL518379 | COc1cc(O)ccc1[C@H]1Oc2cc3oc(=O)ccc3cc2[C@H]2[C@@H]1[C@@H](CC(=C2)C)c1ccc(cc1)O | 482.52 | 3 | 6 | 2 | 137.59 | 89.13 | 4.69 | No | No | No | No | No | 0 | 3 | 0 | 0 | 1 | 0.55 |
| CHEMBL399036 | C=C1CC[C@@H]2O[C@@]2(CC[C@@H]2[C@@H]1CC2(C)C)C | 220.35 | 0 | 1 | 0 | 68.27 | 12.53 | 3.68 | No | Yes | Yes | No | No | 0 | 0 | 0 | 0 | 1 | 0.55 |
| CHEMBL1087720 | COc1ccc(cc1)c1cc(=O)c2c(o1)cc(cc2OC)OC | 312.32 | 4 | 5 | 0 | 87.4 | 57.9 | 3.1 | Yes | Yes | Yes | Yes | Yes | 0 | 0 | 0 | 0 | 0 | 0.55 |
| CHEMBL464176 | C=C[C@](CC[C@H]1[C@](C)(O)CC[C@@H]2[C@]1(C)CCCC2(C)C)(O)C | 308.5 | 4 | 2 | 2 | 95.43 | 40.46 | 4.34 | No | No | No | Yes | No | 0 | 0 | 0 | 0 | 0 | 0.55 |
| CHEMBL572482 | COc1c2c(CC=C(C)C)c3OCOc3c(c2nc2c1cco2)O | 327.33 | 3 | 6 | 1 | 89.81 | 73.95 | 3.46 | Yes | Yes | Yes | Yes | No | 0 | 0 | 0 | 0 | 0 | 0.55 |
| CHEMBL1957109 | COc1ccc2c(c1)[nH]cc2C(=O)c1nccc2c1[nH]c1c2ccc(c1)OC | 371.39 | 4 | 4 | 2 | 108.31 | 80 | 3.45 | Yes | Yes | Yes | Yes | Yes | 0 | 0 | 0 | 0 | 0 | 0.55 |
| CHEMBL496643 | COc1ccccc1c1coc2c(c1=O)c(O)c(c(c2)O)C | 298.29 | 2 | 5 | 2 | 83.42 | 79.9 | 2.79 | Yes | No | Yes | Yes | Yes | 0 | 0 | 0 | 0 | 0 | 0.55 |
| CHEMBL469932 | CO[C@H]1C[C@H](C)Cc2cccc(c2)N=C(O)/C(=C/CC[C@@H]([C@H](/C(=C/[C@@H]([C@H]1O)C)/C)OC(=N)O)OC)/C | 502.64 | 4 | 8 | 4 | 148.17 | 124.59 | 3.67 | No | No | No | No | Yes | 1 | 3 | 0 | 0 | 0 | 0.55 |
| CHEMBL550232 | COc1cc2c(cc1O)O[C@@H]1[C@H]2COc2c1cc1c(c2)OC([C@@H](C1)O)(C)C | 370.4 | 1 | 6 | 2 | 98.13 | 77.38 | 2.69 | No | No | No | Yes | Yes | 0 | 0 | 0 | 0 | 0 | 0.55 |
| CHEMBL1075910 | Oc1ccc(cc1)/C=C/C(=O)O[C@H]1[C@@H](OC(=O)/C=C/c2ccc(cc2)O)[C@@H](O[C@H]([C@@H]1O)C)Oc1c(oc2c(c1=O)c(O)cc(c2)O)c1ccc(cc1)O | 724.66 | 11 | 14 | 6 | 189.72 | 222.65 | 3.63 | No | No | Yes | No | No | 3 | 3 | 2 | 1 | 5 | 0.17 |
| CHEMBL341800 | O=C1C=CC=C[C@@H](OCC[C@H]([C@@H](C(=O)OC[C@]23[C@]4([C@H](O1)C[C@H]([C@]14CO1)O[C@@H]3C=C(CC2)C)C)O)C)[C@@H](O)C | 532.62 | 1 | 9 | 2 | 137.19 | 124.05 | 2.35 | No | No | No | No | No | 1 | 3 | 0 | 0 | 0 | 0.55 |
| CHEMBL1641925 | CCCCCCCCCCCCCCCC(=O)O[C@H]1CC[C@]2([C@H](C1(C)C)CC=C1[C@@H]2CC[C@@]2([C@]1(C)CC[C@H]2[C@@H]1C[C@@H](O[C@@H]1OC)[C@@H](C(O)(C)C)O)C)C | 743.15 | 20 | 6 | 2 | 221.78 | 85.22 | 10.29 | No | No | No | No | Yes | 2 | 4 | 1 | 1 | 3 | 0.17 |
| CHEMBL1078436 | OC[C@@]1(C)[C@@H](O)CC[C@@H]2C1=C[C@H](O)[C@@H]1[C@@]2(C)CC[C@]2([C@@]1(C)CC[C@@H]2[C@@H](C[C@H](C=C(C)C)O)C)C | 474.72 | 5 | 4 | 4 | 140.53 | 80.92 | 4.95 | No | No | No | No | No | 1 | 2 | 0 | 0 | 1 | 0.55 |
| CHEMBL400074 | OC[C@]12CC[C@@]3(C(=CC[C@H]4[C@@]3(C)CC[C@@H]3[C@]4(C)CC[C@@H](C3(C)C)O)[C@@H]2CC(CC1)(C)C)C | 442.72 | 1 | 2 | 2 | 136.04 | 40.46 | 6.27 | No | No | No | No | No | 1 | 3 | 0 | 1 | 1 | 0.55 |
| CHEMBL1254762 | OC[C@@]1(C)[C@@H](O)CC[C@@H]2C1=CC(=O)[C@@H]1[C@@]2(C)CC[C@]2([C@@]1(C)CC[C@@H]2[C@@H](CC(=O)C=C(C)C)C)C | 470.68 | 5 | 4 | 2 | 138.6 | 74.6 | 5.13 | No | No | No | No | Yes | 0 | 3 | 0 | 0 | 1 | 0.55 |
| CHEMBL520763 | OC[C@@]12O[C@@]34O[C@@H]1CC[C@]2(C)[C@@]3(C)CC(=O)C(=C4)C | 264.32 | 1 | 4 | 1 | 68.38 | 55.76 | 1.68 | No | No | No | No | No | 0 | 0 | 0 | 0 | 0 | 0.55 |
| CHEMBL1078266 | C#C[C@@H](COc1ccc(cc1)COC(=O)C)O | 234.25 | 6 | 4 | 1 | 62.54 | 55.76 | 1.72 | Yes | No | No | No | No | 0 | 0 | 0 | 0 | 0 | 0.55 |
| CHEMBL1689215 | C=CC(=C)CC[C@H]1[C@](C)(O)CC[C@@H]2[C@]1(C)CCC[C@]2(C)COC(=O)/C=C/c1ccc(cc1)O | 452.63 | 9 | 4 | 2 | 136.29 | 66.76 | 5.91 | No | No | Yes | No | Yes | 1 | 3 | 0 | 1 | 1 | 0.55 |
| CHEMBL1087520 | COc1cccc2c1[nH]c1c2c(OC)cnc1[C@@H]1CC[C@H]1c1ncc(c2c1[nH]c1c2cccc1)OC | 478.54 | 5 | 5 | 2 | 141.99 | 85.05 | 4.82 | No | No | Yes | Yes | Yes | 0 | 2 | 0 | 1 | 0 | 0.55 |
| CHEMBL465006 | C=CCCCCCC=CCC#CC#C[C@H](C=C)O | 242.36 | 8 | 1 | 1 | 79.9 | 20.23 | 4.4 | Yes | No | Yes | No | No | 1 | 0 | 0 | 0 | 1 | 0.55 |
| CHEMBL387672 | CC(=O)OCC(=CCC/C(=C/COC(=O)C)/C)CCC[C@@H]([C@@H](CC=C(C)C)O)C | 408.57 | 16 | 5 | 1 | 119.79 | 72.83 | 5.22 | Yes | Yes | Yes | No | Yes | 0 | 0 | 1 | 0 | 2 | 0.55 |
| CHEMBL3088138 | CO[C@@H]1OC[C@@H]([C@H]1Cc1ccc(c(c1)OC)O)[C@@H](c1ccc(c(c1)OC)O)O | 390.43 | 7 | 7 | 3 | 102.98 | 97.61 | 2.35 | No | No | No | Yes | No | 0 | 0 | 0 | 0 | 0 | 0.55 |
| CHEMBL465135 | CC(=O)O[C@H]1[C@@H](O)[C@H](OC[C@H]2O[C@@H](Oc3cc(O)c4c(c3)oc(cc4=O)c3ccc(cc3)O)[C@@H]([C@H]([C@@H]2O)O)O)O[C@H]([C@@H]1O)C | 620.56 | 8 | 15 | 7 | 147.07 | 235.04 | -0.02 | No | No | No | No | No | 3 | 4 | 1 | 1 | 4 | 0.17 |
| CHEMBL573138 | O=C[C@H]1C(=CC[C@@H]2[C@]1(C)CC[C@H]1[C@@]2(C)CC[C@@H]2[C@@]1(CCCC2(C)C)COC(=O)C)C(=O)OC | 458.63 | 6 | 5 | 0 | 129.51 | 69.67 | 5.14 | No | No | Yes | No | No | 1 | 1 | 0 | 0 | 1 | 0.55 |
| CHEMBL477855 | COC1=CC(=O)C=C(C1(O)CC(=O)C)OC | 226.23 | 4 | 5 | 1 | 55.7 | 72.83 | 0.31 | No | No | No | No | No | 0 | 0 | 0 | 0 | 0 | 0.85 |
| CHEMBL459914 | OCC1=C[C@H]2[C@@H](C[C@@H]([C@]3(O[C@]1(OC)C=C3)C)OC(=O)/C=C/c1ncn(c1)C)C(=CC[C@@H]2C(C)C)C | 482.61 | 7 | 6 | 1 | 136.26 | 82.81 | 3.28 | No | No | No | Yes | Yes | 0 | 3 | 0 | 0 | 0 | 0.55 |
| CHEMBL1076818 | OC[C@H]1O[C@@H](O[C@@H]2OC=C(C(C2=CC)CC(=O)OC[C@H]2O[C@@H](OCCc3ccc(cc3)O)[C@@H]([C@H]([C@@H]2O)O)O)C(=O)OC)[C@@H]([C@H]([C@@H]1O)O)O | 686.65 | 14 | 17 | 8 | 157.64 | 260.59 | -1.33 | No | No | No | No | No | 3 | 4 | 2 | 1 | 5 | 0.11 |
| CHEMBL1097666 | C#CCCCC[C@H](C(=O)N([C@H](C(=O)N([C@H](C(=N[C@H](C(=O)N([C@H](C(=O)N([C@H](C(=N)O)Cc1ccccc1)C)C)C)C(C)C)O)C(C)C)C)C(C)C)C)C | 724.97 | 24 | 8 | 3 | 211 | 157.91 | 5.05 | No | No | No | No | No | 2 | 3 | 2 | 1 | 4 | 0.17 |
| CHEMBL523569 | OC[C@H]1Oc2c(OC)cc(cc2O[C@@H]1c1cc(OC)c(c(c1)OC)O)c1cc(=O)c2c(o1)cc(cc2O)O | 524.47 | 6 | 11 | 4 | 134.79 | 157.28 | 2.59 | No | No | Yes | No | Yes | 2 | 2 | 1 | 1 | 2 | 0.17 |
| CHEMBL406972 | OC[C@H]([C@H]1N[C@@H]([C@H]([C@@H]1O)O)CO)O | 193.2 | 3 | 6 | 6 | 46.17 | 113.18 | -2.13 | No | No | No | No | No | 1 | 1 | 0 | 0 | 3 | 0.55 |
| CHEMBL446725 | COc1c(CC=C(C)C)c2OC(C)(C)C=Cc2c(c1C(=O)C)O | 316.39 | 4 | 4 | 1 | 92.99 | 55.76 | 3.75 | No | No | Yes | No | Yes | 0 | 0 | 0 | 0 | 0 | 0.55 |
| CHEMBL519603 | COc1cc(cc(c1OC)OC)[C@H]1[C@@H]2C(=O)OC[C@@H]2Cc2c1cc1OCOc1c2 | 398.41 | 4 | 7 | 0 | 102.68 | 72.45 | 3.08 | No | Yes | Yes | Yes | Yes | 0 | 0 | 0 | 0 | 0 | 0.55 |
| CHEMBL212947 | COc1c2C(=O)c3ccccc3C(=O)c2c(c(c1C=O)O)O | 298.25 | 2 | 6 | 2 | 75.67 | 100.9 | 1.68 | No | No | No | No | Yes | 0 | 0 | 0 | 0 | 0 | 0.55 |
| CHEMBL479911 | O[C@]12CO[C@@H]([C@H]1CO[C@@H]2c1ccc2c(c1)OCO2)c1ccc2c(c1)OCO2 | 370.35 | 2 | 7 | 1 | 91.2 | 75.61 | 2.1 | Yes | No | No | Yes | Yes | 0 | 0 | 0 | 0 | 0 | 0.55 |
| CHEMBL455474 | Br/C=C(\C(=C)Cl)/CCC(=C(C)C)Br | 328.47 | 4 | 0 | 0 | 69.3 | 0 | 4.69 | No | No | Yes | No | No | 1 | 0 | 0 | 0 | 2 | 0.55 |
| CHEMBL451077 | CCCCCCCCCC(=O)OCC1=C[C@H]2[C@H]3[C@@](C3(C)C)(OC(=O)CCCCCCC)C[C@H]([C@@]2([C@H]2[C@@](C1)(O)C(=O)C(=C2)C)O)C | 628.88 | 19 | 7 | 2 | 180.32 | 110.13 | 7.14 | No | No | No | No | Yes | 2 | 4 | 1 | 1 | 3 | 0.17 |
| CHEMBL3186786 | CC(=C)CC(C)(C)C | 112.21 | 2 | 0 | 0 | 39.84 | 0 | 3.26 | No | No | No | No | No | 0 | 2 | 0 | 0 | 2 | 0.55 |
| CHEMBL165509 | COc1c(oc2c(c1=O)c(O)c(c(c2)OC)OC)c1ccc(cc1)O | 344.32 | 4 | 7 | 2 | 91.44 | 98.36 | 2.43 | Yes | No | Yes | Yes | Yes | 0 | 0 | 0 | 0 | 0 | 0.55 |
| CHEMBL3093767 | C=C1[C@@H]2CC[C@@H]3[C@](C1=O)(C2)C(=O)O[C@@H]1[C@]23CO[C@H]([C@@H]2C(CC1)(C)C)O | 346.42 | 0 | 5 | 1 | 90.16 | 72.83 | 2.45 | No | No | No | No | No | 0 | 0 | 0 | 0 | 0 | 0.55 |
| CHEMBL3746714 | O[C@H]1CC[C@]2([C@@]3(C1)OO[C@@]1([C@@H]2CC[C@]2([C@H]1CC[C@@H]2[C@@H](/C=C/[C@@H](C(=C)C)C)C)C)C=C3)C | 426.63 | 4 | 3 | 1 | 127.61 | 38.69 | 5.79 | No | No | No | No | No | 1 | 2 | 0 | 1 | 1 | 0.55 |
| CHEMBL1783110 | CC(=CCOc1cc(C)c2c(c1)oc(=O)cc2OCC=C(C)C)C | 328.4 | 6 | 4 | 0 | 97.94 | 48.67 | 4.5 | No | Yes | Yes | No | Yes | 0 | 0 | 0 | 0 | 0 | 0.55 |
| CHEMBL3314602 | Oc1ccc2c(c1)c(=O)c1c(o2)cc2c(c1O)C=CC(O2)(C)C | 310.3 | 0 | 5 | 2 | 88.16 | 79.9 | 2.99 | Yes | No | Yes | Yes | Yes | 0 | 0 | 0 | 0 | 0 | 0.55 |
| CHEMBL334648 | O=C1C=CC=C[C@@H](OCC[C@H]([C@@H](C(=O)OC[C@]23[C@]4([C@H](O1)C[C@H]([C@]14CO1)O[C@@H]3C=C(CC2)C)C)O)C)[C@H](O)C | 532.62 | 1 | 9 | 2 | 137.19 | 124.05 | 2.35 | No | No | No | No | No | 1 | 3 | 0 | 0 | 0 | 0.55 |
| CHEMBL1784266 | CN=C(O[C@H](c1scc(n1)[C@H]1OC(=O)/C(=C/C/C(=C/[C@@H](O[C@@H]2O[C@H](CO)[C@H]([C@@H]([C@H]2O)O)O)[C@@H](C)/C=C(/C)\C=C(/C=C/[C@H]([C@@H]([C@@H](/C(=C/C=C/[C@@H]1C)/C)OC)C)O)C)/C)/C)CC(C)C)O | 901.16 | 10 | 14 | 6 | 247.89 | 238.09 | 4.16 | No | No | No | No | Yes | 3 | 4 | 1 | 2 | 5 | 0.17 |
| CHEMBL3288841 | CC(=CCc1c(O)ccc(c1O)[C@]12Oc3cc(cc(c3[C@H]3[C@@H]2[C@H](c2c(O1)cc(O)cc2)CC(=C3)C)O)c1oc2c(c1)ccc(c2)O)C | 630.68 | 4 | 8 | 5 | 179.51 | 132.75 | 6.37 | No | No | No | No | No | 2 | 4 | 0 | 2 | 3 | 0.17 |
| CHEMBL482243 | COc1cc2c(cc1O)oc1c(c2=O)c(O)cc(c1)O | 274.23 | 1 | 6 | 3 | 72.55 | 100.13 | 1.74 | Yes | No | No | Yes | Yes | 0 | 0 | 0 | 0 | 0 | 0.55 |
| CHEMBL465364 | COc1cc(O)c(c(c1OC)OC)C(=O)/C=C/c1ccc(c(c1)O)O | 346.33 | 6 | 7 | 3 | 91.79 | 105.45 | 2.32 | No | No | Yes | No | Yes | 0 | 0 | 0 | 0 | 0 | 0.55 |
| CHEMBL1487 | O[C@@H](C[C@H](CC(=O)O)O)CCn1c(C(C)C)c(c(c1c1ccc(cc1)F)c1ccccc1)C(=Nc1ccccc1)O | 558.64 | 12 | 7 | 4 | 159.31 | 115.28 | 5.34 | No | No | No | Yes | No | 1 | 4 | 1 | 1 | 1 | 0.56 |
| CHEMBL2409394 | OC[C@H]1O[C@@H](OC[C@H]2O[C@@H](Oc3cc(OC)cc4c3c(=O)c3c(o4)c(OC)ccc3O)[C@@H]([C@H]([C@@H]2O)O)O)[C@@H]([C@H]([C@@H]1O)O)O | 612.53 | 8 | 16 | 8 | 141.53 | 247.43 | -1.41 | No | No | No | No | No | 3 | 4 | 1 | 1 | 4 | 0.17 |
| CHEMBL1079408 | COc1cc2c(cc1O)O[C@@H]1[C@H]2COc2c1cc(c(c2)O)CC=C(C)C | 354.4 | 3 | 5 | 2 | 98.91 | 68.15 | 3.61 | Yes | Yes | Yes | Yes | Yes | 0 | 0 | 0 | 0 | 0 | 0.55 |
| CHEMBL452224 | OC/C=C(\CC[C@H]1C(=C)[C@H](O)C[C@@H]2[C@]1(C)C[C@@H](O)C[C@]2(C)COC(=O)C)/C | 380.52 | 7 | 5 | 3 | 106.94 | 86.99 | 3 | No | No | No | No | Yes | 0 | 0 | 0 | 0 | 0 | 0.55 |
| CHEMBL2436433 | COc1cc(ccc1O[C@@H]1O[C@H](COC(=O)/C=C/c2ccc(cc2)O)[C@H]([C@@H]([C@H]1O)O)O)C(=O)C | 474.46 | 9 | 10 | 4 | 118.65 | 151.98 | 1.09 | No | No | No | No | No | 0 | 0 | 1 | 1 | 1 | 0.55 |
| CHEMBL1668342 | O[C@@H]1C[C@@H](O[C@@H]([C@H]1O[C@H]1C[C@@H](O)[C@@H]([C@H](O1)C)O)C)Oc1ccc(c2c1C(=O)c1ccc3c(c1C2=O)c(O)cc(c3)C)O | 580.58 | 4 | 11 | 5 | 148.15 | 172.21 | 2.29 | No | No | No | No | No | 2 | 3 | 1 | 1 | 2 | 0.17 |
| CHEMBL453193 | CCC[C@H](C1=C(Br)[C@](OC1=O)(CI)OC)O | 405.02 | 5 | 4 | 1 | 72 | 55.76 | 2.32 | No | Yes | No | No | No | 0 | 0 | 0 | 0 | 0 | 0.55 |
| CHEMBL567987 | OC(=O)c1nc2c(O)nc(=N)[nH]c2[nH]c1=O | 223.15 | 1 | 7 | 5 | 50.02 | 155.81 | -0.71 | No | No | No | No | No | 0 | 1 | 1 | 1 | 1 | 0.11 |
| CHEMBL1077503 | OC[C@H]1O[C@@H](O[C@H]2C[C@@]3(C)[C@H](C([C@H]2O)(C)C)CC[C@@]2([C@@H]3C(=O)C=C3[C@@]2(C)[C@@H](O)C[C@@]2([C@H]3[C@@H](C)[C@H](C)C(=O)C2)C)C)[C@@H]([C@H]([C@@H]1O)O)O | 648.82 | 3 | 10 | 6 | 170.25 | 173.98 | 2.14 | No | No | No | No | No | 2 | 3 | 1 | 1 | 3 | 0.17 |
| CHEMBL1651033 | O=C1CC[C@]2([C@H](C(O1)(C)C)CC[C@@]1([C@@H]2CC[C@H]2[C@@]1(C)CC[C@@H]2[C@@]1(C)CC[C@H](O1)C(O)(C)C)C)C | 474.72 | 2 | 4 | 1 | 138.62 | 55.76 | 5.82 | No | No | No | No | No | 1 | 3 | 0 | 1 | 1 | 0.55 |
| CHEMBL590549 | O=C1C[C@@H]2[C@]([C@@H]3C1=CC(=CC3)C(C)C)(C)CCC[C@@]2(C)C(=O)O | 316.43 | 2 | 3 | 1 | 92.42 | 54.37 | 3.65 | No | Yes | Yes | No | Yes | 0 | 0 | 0 | 0 | 0 | 0.85 |
| CHEMBL3109412 | COc1ccc(cc1)C=CC(=NCCc1ccc(cc1)O[C@@H]1O[C@@H](C)[C@@H]([C@H]([C@H]1O[C@@H]1O[C@H](COC(=O)C)[C@H]([C@@H]([C@H]1O)O)O)O[C@@H]1O[C@H](COC(=O)C)[C@H]([C@@H]([C@H]1O)O)O)OC(=O)C)O | 893.88 | 20 | 21 | 7 | 214.15 | 297.48 | 0.35 | No | No | No | No | No | 3 | 4 | 2 | 1 | 5 | 0.17 |
| CHEMBL474628 | Oc1cc(CCc2ccccc2O)cc(c1)O | 230.26 | 3 | 3 | 3 | 66.77 | 60.69 | 2.5 | Yes | No | No | Yes | Yes | 0 | 0 | 0 | 0 | 0 | 0.55 |
| CHEMBL491374 | COc1cc(c(cc1Br)Br)[C@H]1[C@@H](O)N(C(=O)[C@@]21CCC=N2)C | 432.11 | 2 | 4 | 1 | 97.94 | 62.13 | 2.33 | No | No | No | No | Yes | 0 | 0 | 0 | 0 | 0 | 0.55 |
| CHEMBL461614 | COc1ccc(c(c1)/C=C/Cc1ccc(c(c1OC)OC)O)O | 316.35 | 6 | 5 | 2 | 89.35 | 68.15 | 3.17 | Yes | Yes | Yes | Yes | Yes | 0 | 0 | 0 | 0 | 0 | 0.55 |
| CHEMBL3634637 | CC(=CCC[C@]([C@H]1CC[C@@]2([C@@H]1[C@H](O)C[C@H]1[C@@]2(C)C[C@@H]([C@@H]2[C@]1(C)CC[C@@H](C2(C)C)O)O)C)(O)C)C | 476.73 | 4 | 4 | 4 | 141.04 | 80.92 | 4.93 | No | No | No | No | No | 1 | 2 | 0 | 0 | 1 | 0.55 |
| CHEMBL253688 | COc1c(OC)cc2c(c1OC)c1c(cc(c(c1OC)OC)OC)C[C@@H]([C@@](C2)(C)O)C | 432.51 | 6 | 7 | 1 | 119.08 | 75.61 | 3.59 | No | No | No | Yes | No | 0 | 0 | 0 | 0 | 0 | 0.55 |
| CHEMBL459851 | O=C1OC[C@H](C1=Cc1ccc2c(c1)OCO2)Cc1ccc2c(c1)OCO2 | 352.34 | 3 | 6 | 0 | 91.54 | 63.22 | 3.28 | Yes | Yes | Yes | Yes | Yes | 0 | 0 | 0 | 0 | 0 | 0.55 |
| CHEMBL589325 | OC[C@H]1c2cc(cc(c2O[C@@H]1c1ccc(c(c1)OC)O)O)[C@H]1Oc2cc(O)cc(c2C(=O)[C@@H]1O)O | 482.44 | 4 | 10 | 6 | 121.05 | 166.14 | 1.44 | No | No | No | No | Yes | 1 | 1 | 1 | 1 | 2 | 0.55 |
| CHEMBL2431896 | Oc1ccc(cc1)[C@@H]1Oc2c(C[C@H]1O)c(O)cc1c2[C@@H]2c3c(O)cc(cc3O[C@](O1)([C@H]2O)c1ccc(cc1)O)O | 544.51 | 2 | 10 | 7 | 140.1 | 169.3 | 2.29 | No | No | Yes | No | Yes | 2 | 2 | 1 | 1 | 2 | 0.17 |
| CHEMBL2337574 | C=C1[C@H]2CC[C@@H]3[C@](C1=O)([C@@H]2O)CC[C@H]1[C@@]3(C)CCCC1(C)C | 302.45 | 0 | 2 | 1 | 89.91 | 37.3 | 3.38 | No | No | Yes | No | No | 0 | 0 | 0 | 0 | 0 | 0.55 |
| CHEMBL2270656 | O=C[C@@H]1C[C@@H](O)C[C@H]([C@]21CC[C@H](C2)C(=C)C)C | 236.35 | 2 | 2 | 1 | 70.62 | 37.3 | 2.88 | No | No | Yes | No | No | 0 | 0 | 0 | 0 | 0 | 0.55 |
| CHEMBL492224 | O[C@@H]1C=C2CC=CC(=O)[C@@]2([C@@H]2[C@@H]1[C@@]1(O)O[C@]34[C@@H](C1=O)[C@]1(C)C[C@H]([C@]4(C)OC(=O)[C@]3(CC2)O)OC(=O)[C@H]1C)C | 528.55 | 0 | 10 | 3 | 128.14 | 156.66 | 0.96 | No | No | No | No | No | 1 | 1 | 1 | 1 | 1 | 0.55 |
| CHEMBL1628279 | OC[C@@]12CC[C@@H](C[C@@]2(O)CC[C@@H]2[C@@H]1CC[C@]1([C@]2(O)CC[C@@H]1C1=CC(=O)OC1)C)O | 406.51 | 2 | 6 | 4 | 107.12 | 107.22 | 1.83 | No | No | No | No | No | 0 | 0 | 0 | 0 | 0 | 0.55 |
| CHEMBL1288770 | CC(C1CC[C@@]2([C@@H](C1)[C@](C)(O)CCC2)C)(O)C | 240.38 | 1 | 2 | 2 | 72.13 | 40.46 | 2.94 | No | No | No | No | No | 0 | 0 | 0 | 0 | 0 | 0.55 |
| CHEMBL60533 | CO/C=C(\[C@H]1C[C@@H]2N(C[C@H]1CC)CCc1c2[nH]c2c1cccc2)/C(=O)OC | 368.47 | 5 | 4 | 1 | 110.39 | 54.56 | 3.21 | No | No | No | Yes | Yes | 0 | 0 | 0 | 0 | 0 | 0.85 |
| CHEMBL1079671 | O[C@H]1C[C@@H](C)C2=CC(=O)[C@@]3([C@@H]([C@]2([C@@H]1O)C)[C@@H](O)[C@@H]1OC(=O)[C@H]3[C@H]1C)C | 350.41 | 0 | 6 | 3 | 88.97 | 104.06 | 0.71 | No | No | No | No | No | 0 | 0 | 0 | 0 | 0 | 0.55 |
| CHEMBL1813582 | CC(=O)O[C@@]12C[C@@H](C)[C@]3([C@H]([C@@H]1C2(C)C)C=C(CO[C@@H]1O[C@H](COC(=O)c2cc(O)c(c(c2)O)O)[C@H]([C@@H]([C@H]1O)O)O)C[C@]1([C@H]3C=C(C1=O)C)O)O | 704.71 | 9 | 15 | 8 | 171.38 | 249.97 | 0.56 | No | No | No | No | No | 3 | 3 | 1 | 1 | 4 | 0.17 |
| CHEMBL1287907 | C[C@H]1Cc2c(O1)c(O)c1c(c2O)C(=O)C[C@@H]2[C@]1(C)CC[C@@H]1[C@@]2(C)C1 | 328.4 | 0 | 4 | 2 | 91.54 | 66.76 | 3.64 | Yes | No | Yes | Yes | No | 0 | 0 | 0 | 0 | 0 | 0.55 |
| CHEMBL226995 | CC(=CCCC([C@H]1CC[C@@]2([C@]1(C)CC=C1C2=CC[C@@H]2[C@]1(C)CC[C@@H](C2(C)C)O)C)C(=O)O)C | 454.68 | 5 | 3 | 2 | 138.34 | 57.53 | 6.14 | No | No | Yes | No | No | 1 | 3 | 0 | 1 | 1 | 0.85 |
| CHEMBL155625 | OCc1c[nH]c2c1cccc2 | 147.17 | 1 | 1 | 2 | 44.43 | 36.02 | 1.45 | Yes | No | No | No | No | 0 | 1 | 0 | 0 | 1 | 0.55 |
| CHEMBL401904 | CC(=CCC[C@H]([C@H]1CC[C@@]2([C@]1(C)CC[C@@]13[C@H]2CC[C@@H]2[C@]3(C1)[C@@H](OC(=O)C)[C@@H](O)[C@@H](C2(C)C)O)C)C)C | 500.75 | 6 | 4 | 2 | 147.2 | 66.76 | 5.97 | No | No | No | No | No | 2 | 4 | 0 | 1 | 1 | 0.17 |
| CHEMBL482040 | C[C@@H]1CCC2=C3C1=COC[C@@]3(C)[C@@H](C2(C)C)O | 234.33 | 0 | 2 | 1 | 68.66 | 29.46 | 2.73 | No | No | No | No | No | 0 | 0 | 0 | 0 | 0 | 0.55 |
| CHEMBL2368274 | OC[C@H]1O[C@@H](OC2=C[C@@]3(C)[C@H]([C@@H](C2=O)C)C[C@@H]2[C@@]45[C@@H]3[C@@H](O)[C@H](O)[C@]([C@@H]5[C@H](C(=O)O2)OC(=O)c2ccccc2)(OC4)C(=O)OC)[C@@H]([C@H]([C@@H]1O)O)O | 704.67 | 8 | 16 | 6 | 162.06 | 245.04 | -0.25 | No | No | No | No | No | 3 | 4 | 1 | 1 | 4 | 0.17 |
| CHEMBL272485 | CCCCOC(=O)c1ccccc1C(=O)OCCCC | 278.34 | 10 | 4 | 0 | 77.84 | 52.6 | 3.69 | Yes | Yes | No | No | No | 0 | 0 | 0 | 0 | 0 | 0.55 |
| CHEMBL463255 | COc1cc(O)c2c(c1CC=C(C)C)O[C@@H](CC2)c1ccc(cc1O)O | 356.41 | 4 | 5 | 3 | 101.36 | 79.15 | 3.61 | Yes | No | Yes | Yes | Yes | 0 | 0 | 0 | 0 | 0 | 0.55 |
| CHEMBL1651328 | COC(=O)/C=C/NC(=O)c1nc(C(=O)C)c2c(c1)c1ccccc1[nH]2 | 337.33 | 6 | 5 | 2 | 92.02 | 101.15 | 2.21 | Yes | No | Yes | No | Yes | 0 | 0 | 0 | 0 | 0 | 0.55 |
| CHEMBL464484 | CC(Cc1ncc(o1)c1c[nH]c2c1cccc2)C | 240.3 | 3 | 2 | 1 | 73.18 | 41.82 | 3.32 | Yes | Yes | No | Yes | Yes | 0 | 0 | 0 | 0 | 0 | 0.55 |
| CHEMBL2063158 | OC[C@H]1O[C@@H](OC(=O)[C@]23CC[C@@]4([C@@H]([C@H]3[C@@H](C[C@@H]2O)C(=C)C)C[C@H]([C@H]2[C@@]4(C)CC[C@@H]3[C@]2(C)[C@@H](CC(=O)OC)OC3(C)C)O)C)[C@@H]([C@H]([C@@H]1O)O)O | 694.85 | 8 | 12 | 6 | 177.52 | 192.44 | 2.45 | No | No | No | No | No | 3 | 3 | 1 | 1 | 4 | 0.17 |
| CHEMBL261634 | OC[C@H]1NC[C@@H]([C@@H]1O)O | 133.15 | 1 | 4 | 4 | 34.24 | 72.72 | -1.27 | No | No | No | No | No | 0 | 3 | 0 | 0 | 1 | 0.55 |
| CHEMBL445702 | COc1cc(O)cc(c1CC=C(C)C)/C=C/c1ccccc1 | 294.39 | 5 | 2 | 1 | 94.05 | 29.46 | 4.73 | Yes | Yes | Yes | Yes | Yes | 1 | 0 | 0 | 0 | 1 | 0.55 |
| CHEMBL391546 | CCC(=O)O[C@H]1CC(=C2C([C@@H]1[C@@H](OC(=O)C)[C@@H]1C(=C)[C@H](CC[C@]1(C[C@@H]2OC(=O)C)C)OC(=O)C)(C)C)C | 518.64 | 9 | 8 | 0 | 138.85 | 105.2 | 4.01 | No | No | No | No | No | 1 | 3 | 0 | 0 | 0 | 0.55 |
| CHEMBL447517 | ClC1=C(N)C(=O)c2c3C1=NCCc3cn2C | 235.67 | 0 | 2 | 1 | 65.25 | 60.38 | 1.08 | Yes | No | No | No | No | 0 | 0 | 0 | 0 | 0 | 0.55 |
| CHEMBL365375 | O[C@H]1CC[C@]2(C(C1(C)C)CC[C@@]1(C2CC=C2[C@@]1(C)CC[C@@]1(C2CC(C)(C)CC1)C(=O)O)C)C | 456.7 | 1 | 3 | 2 | 136.65 | 57.53 | 6.07 | No | No | No | No | No | 1 | 3 | 0 | 1 | 1 | 0.85 |
| CHEMBL449362 | BrC(C(=O)O)Br | 217.84 | 1 | 2 | 1 | 29.24 | 37.3 | 1.11 | No | No | No | No | No | 0 | 2 | 0 | 0 | 1 | 0.85 |
| CHEMBL1773216 | CC(=C)[C@@H]1CC[C@]2([C@H]1[C@H]1CC[C@H]3[C@@]([C@]1(C)CC2)(C)CC[C@@H]1[C@]3(C)C[C@@H](O)[C@@H](C1(C)C)O)C | 442.72 | 1 | 2 | 2 | 136.3 | 40.46 | 6.41 | No | No | No | No | No | 1 | 3 | 0 | 1 | 1 | 0.55 |
| CHEMBL447875 | CO[C@@H]1CO[C@H]([C@@H]([C@H]1O)O)OCC[C@H](C(C)C)CC[C@H]([C@H]1[C@@H](O)[C@H]([C@@H]2[C@]1(C)CC[C@H]1[C@H]2C[C@@H]([C@@H]2[C@]1(C)CC[C@@H](C2)O)O)O)C | 626.86 | 10 | 9 | 6 | 169.49 | 149.07 | 3.2 | No | No | No | No | No | 2 | 3 | 1 | 1 | 2 | 0.17 |
| CHEMBL462770 | O[C@H]1CC[C@]2([C@]3(C1)O[C@@H]3C[C@@H]1[C@@H]2CC[C@]2([C@H]1CC[C@@H]2[C@@H](CCC(=C)C(C)C)C)C)COC(=O)C | 472.7 | 8 | 4 | 1 | 138.33 | 59.06 | 6.02 | No | No | No | No | No | 1 | 3 | 0 | 1 | 1 | 0.55 |
| CHEMBL400142 | CC(=O)OC[C@]1(C)CCC[C@@]23[C@@H]1[C@H](O)[C@](O)(OC2)[C@@]12[C@H]3CC[C@H]([C@H]2O)C(=C)C1=O | 406.47 | 3 | 7 | 3 | 102.14 | 113.29 | 0.95 | No | No | No | No | No | 0 | 0 | 0 | 0 | 0 | 0.55 |
| CHEMBL2171348 | O=Cc1nccc2c1[nH]c1c2cccc1 | 196.2 | 1 | 2 | 1 | 58.99 | 45.75 | 1.94 | Yes | No | No | No | No | 0 | 0 | 0 | 0 | 1 | 0.55 |
| CHEMBL425900 | C=C1C(=O)O[C@H]2[C@@H]1CC1=C([C@H](C2)C)C(=O)C[C@@H]1C | 246.3 | 0 | 3 | 0 | 68.41 | 43.37 | 2.35 | No | No | No | No | No | 0 | 0 | 0 | 0 | 0 | 0.55 |
| CHEMBL2208204 | CO[C@H]1C=C[C@]23[C@H](C1)N(C)C(=O)[C@H]2N(C)Cc1c3cc2OCOc2c1 | 342.39 | 1 | 5 | 0 | 98.48 | 51.24 | 1.46 | No | No | No | Yes | No | 0 | 0 | 0 | 0 | 0 | 0.55 |
| CHEMBL1605700 | CC(=CCc1c2OC34C(C(=O)c2c(c2c1OC(C)(CCC=C(C)C)C=C2)O)CC1CC3C(OC4(C/C=C(\C(=O)O)/C)C1O)(C)C)C | 632.78 | 8 | 8 | 3 | 179.05 | 122.52 | 6.03 | No | No | No | No | Yes | 1 | 4 | 0 | 1 | 2 | 0.56 |
| CHEMBL442720 | OC(=O)[C@H](CCS(=O)(=O)C)N | 181.21 | 4 | 5 | 2 | 39.59 | 105.84 | -1.47 | No | No | No | No | No | 0 | 1 | 0 | 0 | 2 | 0.55 |
| CHEMBL3317744 | CC1=CC(=O)[C@H]([C@]2([C@H]1C[C@H]1OC(=O)[C@@H]([C@@]3([C@]41[C@@H]2[C@](O)(OC4)[C@@H]([C@]13CO1)O)O)O)C)O | 424.4 | 0 | 10 | 5 | 94.15 | 166.28 | -1.62 | No | No | No | No | No | 0 | 1 | 1 | 1 | 2 | 0.55 |
| CHEMBL252790 | Oc1ccc(cc1)C[C@@H](C(=O)c1ccc(cc1O)O)O | 274.27 | 4 | 5 | 4 | 73.16 | 97.99 | 1.61 | Yes | No | No | No | Yes | 0 | 0 | 0 | 0 | 0 | 0.55 |
| CHEMBL1575074 | CC(=CC=CC=C(C=CC=C(C(=O)C[C@]12O[C@]2(C)C[C@H](CC1(C)C)O)C)C)/C=C/C=C(/C=C=C1C(C)(C)C[C@@H](C[C@@]1(C)O)OC(=O)C)\C | 658.91 | 12 | 6 | 2 | 197.07 | 96.36 | 7.8 | No | No | No | No | No | 2 | 4 | 1 | 1 | 2 | 0.17 |
| CHEMBL465565 | COc1cc(ccc1O[C@@H]1O[C@H](COC(=O)c2cc(OC)c(c(c2)OC)O)[C@H]([C@@H]([C@H]1O)O)O)C(=O)OC[C@H]1O[C@@H](Oc2ccc(cc2OC)O)[C@@H]([C@H]([C@@H]1O)O)O | 794.71 | 16 | 20 | 8 | 184.02 | 288.28 | -0.37 | No | No | No | No | No | 3 | 4 | 2 | 1 | 5 | 0.17 |
| CHEMBL443893 | CNC[C@@H](c1ccc(cc1)O)O | 167.21 | 3 | 3 | 3 | 47.01 | 52.49 | 0.55 | No | No | No | No | No | 0 | 0 | 0 | 0 | 1 | 0.55 |
| CHEMBL1087283 | C/C(=C/C=C/C=C/C=C/C=C/C(=O)O)/Cl | 224.68 | 5 | 2 | 1 | 64 | 37.3 | 3.12 | No | No | Yes | No | No | 0 | 0 | 0 | 0 | 0 | 0.85 |
| CHEMBL2409055 | COc1cc(ccc1O)[C@@H]1CCc2c(O1)cc(c(c2)O)OC | 302.32 | 3 | 5 | 2 | 82.11 | 68.15 | 2.7 | Yes | No | No | Yes | Yes | 0 | 0 | 0 | 0 | 0 | 0.55 |
| CHEMBL1077634 | OC[C@H]1O[C@@H](OC(=O)[C@@]23CC[C@H](C(=C)[C@H]3C3=CC[C@H]4[C@@]([C@@]3(CC2)C)(C)CC[C@@H]2[C@]4(C)C[C@H]([C@@H]([C@@]2(C)CO)O)O)C)[C@@H]([C@H]([C@@H]1O)O)O | 648.82 | 5 | 10 | 7 | 170.74 | 177.14 | 2.57 | No | No | No | No | No | 2 | 3 | 1 | 1 | 3 | 0.17 |
| CHEMBL255459 | COc1cc(cc(c1O)OC)[C@H]1OC[C@@H]([C@@H]1COC(=O)C=Cc1ccc(c(c1)OC)O)Cc1cc(OC)c(c(c1)OC)O | 596.62 | 13 | 11 | 3 | 157.94 | 142.37 | 3.89 | No | No | No | No | No | 2 | 3 | 2 | 1 | 1 | 0.17 |
| CHEMBL403894 | CC(C[C@H](C(=N[C@H]1[C@@H](C)OC(=O)[C@@H](N=C(O)[C@H](Cc2ccc(cc2)O)N(C)C(=O)[C@@H](N2C(=O)[C@@H](N=C([C@@H](N=C1O)CCCNC(=N)N)O)CC[C@H]2O)CC(C)C)C(C)C)O)N=C([C@@H](COS(=O)(=O)O)O)O)C | 1041.18 | 19 | 22 | 12 | 286.48 | 424.44 | 0.9 | No | No | No | No | No | 3 | 3 | 2 | 1 | 5 | 0.17 |
| CHEMBL1231671 | O[C@@H]1[C@H](O)[C@@H](O)[C@@H]([C@H]([C@H]1O)O)O | 180.16 | 0 | 6 | 6 | 35.81 | 121.38 | -2.67 | No | No | No | No | No | 1 | 2 | 0 | 0 | 3 | 0.55 |
| CHEMBL436993 | CC(=O)O[C@H]1CC[C@@]2([C@@H](C1=C)[C@H](OC(=O)C)[C@@H]1[C@@H](OC(=O)C)CC(=C([C@H](C2)OC(=O)C)C1(C)C)C)C | 504.61 | 8 | 8 | 0 | 134.04 | 105.2 | 3.85 | No | No | No | No | No | 1 | 3 | 0 | 0 | 0 | 0.55 |
| CHEMBL243089 | CC(=O)Oc1ccc2c(c1)oc(c(c2=O)c1ccccc1)C | 294.3 | 3 | 4 | 0 | 84.38 | 56.51 | 3.32 | Yes | Yes | Yes | No | No | 0 | 0 | 0 | 0 | 0 | 0.55 |
| CHEMBL552509 | OC/C(=C/1\Oc2c(C1=O)c(OC)ccc2)/[C@@H]1OC(=O)C[C@H]1C | 304.29 | 3 | 6 | 1 | 76.21 | 82.06 | 1.64 | No | No | No | No | No | 0 | 0 | 0 | 0 | 0 | 0.55 |
| CHEMBL1965265 | COc1ccc2c(c1)c(=O)c1c(o2)c(OC)c(c(c1)OC)OC | 316.31 | 4 | 6 | 0 | 85.96 | 67.13 | 2.72 | Yes | Yes | Yes | Yes | Yes | 0 | 0 | 0 | 0 | 0 | 0.55 |
| CHEMBL467203 | COC1=C(c2ccccc2)C(=O)C(=C(C1=O)c1ccccc1)OC | 320.34 | 4 | 4 | 0 | 90.64 | 52.6 | 3.07 | Yes | Yes | Yes | No | Yes | 0 | 0 | 0 | 0 | 0 | 0.85 |
| CHEMBL491589 | C[C@H](C(C)C)CC[C@H]([C@H]1CC[C@@H]2[C@]1(C)C[C@@H](O)[C@H]1[C@H]2C[C@H](C2=CC=CC(=O)[C@]12C)O)C | 428.65 | 5 | 3 | 2 | 129.31 | 57.53 | 5.17 | No | No | No | No | No | 1 | 1 | 0 | 0 | 1 | 0.55 |
| CHEMBL402213 | C[C@@H]1C[C@@H]2[C@@]([C@@H](C1=O)O)(C)CC[C@]1([C@@]2(C)CC[C@@]2([C@@H]1CCc1c2cc(O)c(c1C)O)C)C | 440.61 | 0 | 4 | 3 | 128.73 | 77.76 | 4.93 | No | No | No | No | No | 0 | 1 | 0 | 0 | 1 | 0.55 |
| CHEMBL3298992 | CC[C@@H](C(=O)O[C@H]1[C@@H](OC(=O)/C=C(/CC)\C)C(=C)[C@H]2[C@H]([C@@H]1[C@@]1(C)CO1)[C@@H]([C@H](OC(=O)C)C)C(=O)C2)C | 504.61 | 12 | 8 | 0 | 134.6 | 108.5 | 3.9 | No | No | No | No | Yes | 1 | 3 | 1 | 0 | 0 | 0.55 |
| CHEMBL511511 | COc1c(CC=C(C)C)c(O)c(c(c1C(=O)C)O)C=O | 278.3 | 5 | 5 | 2 | 76.28 | 83.83 | 2.55 | Yes | No | No | No | Yes | 0 | 0 | 0 | 0 | 0 | 0.55 |
| CHEMBL597425 | OCC1(C)CCc2c(O1)cc1c(c2OC)cc(c(=O)o1)c1ccc(cc1O)O | 384.38 | 3 | 7 | 3 | 103.42 | 109.36 | 2.69 | No | No | Yes | Yes | Yes | 0 | 0 | 0 | 0 | 0 | 0.55 |
| CHEMBL513108 | NCCC1=C(SC)C(=O)c2c3c1nc1ccccc1c3ccn2 | 321.4 | 3 | 4 | 1 | 95.13 | 94.17 | 2.6 | Yes | Yes | Yes | Yes | Yes | 0 | 0 | 0 | 0 | 0 | 0.55 |
| CHEMBL212301 | NC(=N)NCCC[C@H](C(=O)O)N | 174.2 | 6 | 4 | 5 | 44.54 | 125.22 | -2.06 | No | No | No | No | No | 0 | 1 | 0 | 0 | 2 | 0.55 |
| CHEMBL457153 | O=C1C[C@H]2[C@]3(C)CC[C@@H]4[C@]([C@H]3C[C@H]([C@@]2(c2c1coc2)C)O)(C)CCCC4(C)C | 384.55 | 0 | 3 | 1 | 112.2 | 50.44 | 4.88 | Yes | No | No | No | No | 0 | 1 | 0 | 0 | 1 | 0.55 |
| CHEMBL254599 | COc1ccc2c(c1O)CCc1c2c(OC)c(c(c1)O)OC | 302.32 | 3 | 5 | 2 | 83.22 | 68.15 | 2.71 | Yes | No | No | Yes | Yes | 0 | 0 | 0 | 0 | 0 | 0.55 |
| CHEMBL2005772 | CCC=CCC=CCC=CCCCCCCCC(=O)OCC | 306.48 | 15 | 2 | 0 | 98.12 | 26.3 | 5.82 | Yes | No | Yes | No | No | 1 | 1 | 1 | 1 | 1 | 0.55 |
| CHEMBL492158 | O[C@H]1CC[C@]2([C@@]3(C1)O[C@H]3[C@H](C1=C3[C@@](CC[C@H]21)(C)[C@H](CC3)[C@@H](/C=C/[C@@H](C(C)C)C)C)O)C | 428.65 | 4 | 3 | 2 | 128.12 | 52.99 | 5.12 | No | No | No | No | No | 1 | 2 | 0 | 0 | 1 | 0.55 |
| CHEMBL1096781 | CC(=O)O[C@H]1CC[C@@](C)(O)[C@@H]2O[C@H](CC1=C)[C@@H]1[C@H]2[C@H](CCC1=C)C(C)C | 362.5 | 3 | 4 | 1 | 104.15 | 55.76 | 3.6 | No | No | No | No | No | 0 | 0 | 0 | 0 | 0 | 0.55 |
| CHEMBL463922 | CO[C@@H]1OC[C@]2([C@@H]1[C@]1(CCC2)COC(=O)[C@]23[C@H]1[C@H](O)C[C@H](C2)C(=C)C3=O)C | 376.44 | 1 | 6 | 1 | 96.05 | 82.06 | 2.14 | No | No | No | No | No | 0 | 0 | 0 | 0 | 0 | 0.55 |
| CHEMBL141117 | COc1cc(cc(c1O)OC)[C@H]1[C@H]2C(=O)OCC2Cc2c1cc1OCOc1c2 | 384.38 | 3 | 7 | 1 | 98.22 | 83.45 | 2.72 | No | No | Yes | Yes | Yes | 0 | 0 | 0 | 0 | 0 | 0.55 |
| CHEMBL325752 | COc1c(ccc2c1C(=O)OCc1c(O2)c(O)cc(c1)C)[C@H](CC(C)C)O | 372.41 | 4 | 6 | 2 | 101.12 | 85.22 | 3.51 | No | No | Yes | No | No | 0 | 0 | 0 | 0 | 0 | 0.55 |
| CHEMBL25308 | OC/C=C(/CC/C=C(/CCC=C(C)C)\C)\C | 222.37 | 7 | 1 | 1 | 73.96 | 20.23 | 3.51 | No | No | Yes | No | No | 0 | 0 | 0 | 0 | 0 | 0.55 |
| CHEMBL479323 | CC1=CC[C@H]2[C@@]([C@]31Oc1c(C3)c(=O)oc(c1)c1cccnc1)(C)CC[C@@H]1[C@]2(C)CCC(=O)C1(C)C | 473.6 | 1 | 5 | 0 | 136.72 | 69.4 | 5.04 | No | No | No | No | Yes | 0 | 2 | 0 | 1 | 0 | 0.55 |
| CHEMBL464753 | C#CC=CCCCCC=CC#CC#CCCCC=CC#CCO | 304.43 | 8 | 1 | 1 | 100.26 | 20.23 | 5.34 | Yes | No | Yes | No | No | 1 | 0 | 0 | 0 | 2 | 0.55 |
| CHEMBL507146 | Oc1ccc(cc1)C=C1C(=O)C(=C2C1=Nc1c2cccc1)C1=C2C(=Nc3c2cccc3)/C(=C\c2ccc(cc2)O)/C1=O | 544.55 | 3 | 6 | 2 | 170.46 | 99.32 | 5.29 | Yes | No | No | No | No | 1 | 3 | 0 | 0 | 2 | 0.55 |
| CHEMBL506581 | CC(C[C@@H]1N=C(O)[C@@H]2CCCN2C(=O)[C@@H](N=C(O)c2csc(CN=C(c3nc([C@@H](N=C1O)CCC(=N)O)sc3)O)n2)C(C)C)C | 660.81 | 6 | 13 | 6 | 195.59 | 277.01 | 2.75 | No | No | No | No | No | 3 | 3 | 1 | 1 | 4 | 0.17 |
| CHEMBL110441 | CCCCCCCCCCCC[C@@H]([C@@H]1CC[C@H](O1)[C@H](CCCCCCC(CCCCC[C@H](CC1=C[C@@H](OC1=O)C)O)O)O)O | 624.93 | 28 | 7 | 4 | 182.29 | 116.45 | 7.6 | No | No | No | No | Yes | 1 | 4 | 1 | 1 | 3 | 0.55 |
| CHEMBL1275976 | CCCCCCCCCCC#CCCCCC(=O)O | 266.42 | 12 | 2 | 1 | 83.77 | 37.3 | 5.08 | Yes | No | No | No | No | 1 | 0 | 1 | 0 | 1 | 0.85 |
| CHEMBL507971 | C=C1CC[C@@H]2[C@]([C@H]1C)(C)CCCC2(C)C | 206.37 | 0 | 0 | 0 | 69 | 0 | 4.69 | No | Yes | Yes | No | No | 1 | 0 | 0 | 0 | 2 | 0.55 |
| CHEMBL573376 | CC(=CCc1c(C)[nH]c2c(c1=O)cccc2)CC/C=C(/CCC=C(C)C)\C | 363.54 | 8 | 1 | 1 | 120.38 | 32.86 | 6.34 | No | Yes | No | No | Yes | 1 | 1 | 0 | 1 | 1 | 0.55 |
| CHEMBL499268 | CCCC(=O)O[C@@H]1[C@H](C(C)C)[C@H]2[C@]([C@H]1OC(=O)CCC)(C)[C@@H](OC(=O)CCC)C[C@@]1([C@@H]2C=C(C)[C@@H]([C@@H]([C@H]1OC(=O)CCC)OC(=O)C)O)C | 692.88 | 19 | 11 | 1 | 185.03 | 151.73 | 5.53 | No | Yes | No | No | Yes | 2 | 4 | 2 | 2 | 5 | 0.17 |
| CHEMBL464885 | COc1cc2c(cc1OC)OC[C@@H]1[C@H]2C(=O)c2c(O1)c1c(cc2)OC([C@@H]([C@H]1O)O)(C)C | 428.43 | 2 | 8 | 2 | 108.99 | 103.68 | 1.98 | No | No | No | Yes | Yes | 0 | 0 | 0 | 0 | 0 | 0.55 |
| CHEMBL348446 | C[C@@H](c1ccccc1)O | 122.16 | 1 | 1 | 1 | 37.38 | 20.23 | 1.7 | Yes | No | No | No | No | 0 | 3 | 0 | 0 | 2 | 0.55 |
| CHEMBL2023567 | CC(=CC[C@]12O[C@H]1C(=O)c1c(C2=O)c(O)ccc1)C | 258.27 | 2 | 4 | 1 | 69.31 | 66.9 | 2.35 | Yes | Yes | Yes | No | No | 0 | 0 | 0 | 0 | 0 | 0.55 |
| CHEMBL165064 | COc1c(oc2c(c1=O)c(O)cc(c2)O)c1ccc(cc1)O | 300.26 | 2 | 6 | 3 | 80.48 | 100.13 | 1.94 | Yes | No | No | Yes | Yes | 0 | 0 | 0 | 0 | 0 | 0.55 |
| CHEMBL2289234 | O=Cc1ccc2c(c1)cccc2 | 156.18 | 1 | 1 | 0 | 49.34 | 17.07 | 2.49 | Yes | No | No | No | No | 0 | 1 | 0 | 0 | 2 | 0.55 |
| CHEMBL1169874 | CC(=CCc1cc(/C=C/C(=O)c2cc(CC=C(C)C)c(cc2O)O)ccc1O)C | 392.49 | 7 | 4 | 3 | 119.76 | 77.76 | 5.18 | Yes | No | Yes | No | Yes | 0 | 1 | 0 | 0 | 1 | 0.55 |
| CHEMBL497275 | C/C=C(\C(=O)OCc1nccc2c1C(=O)C(=C(C2=O)C)NC)/C | 314.34 | 5 | 5 | 1 | 84.27 | 85.36 | 1.8 | Yes | Yes | No | No | No | 0 | 0 | 0 | 0 | 0 | 0.55 |
| CHEMBL3401056 | COc1c(ccc2c1C=CC(O2)(C)C)C(=O)/C=C(/c1ccccc1)\O | 336.38 | 4 | 4 | 1 | 98.43 | 55.76 | 3.81 | Yes | Yes | Yes | No | Yes | 0 | 0 | 0 | 0 | 0 | 0.85 |
| CHEMBL462956 | COc1ccc2c(c1O)[C@H](Cc1ccc(cc1)O)N(CC2)C | 299.36 | 3 | 4 | 2 | 90.52 | 52.93 | 2.57 | No | No | No | Yes | No | 0 | 0 | 0 | 0 | 0 | 0.55 |
| CHEMBL3184929 | O=C(C(C)C)OCCc1ccccc1 | 192.25 | 5 | 2 | 0 | 56.73 | 26.3 | 2.81 | No | No | No | No | No | 0 | 0 | 0 | 0 | 1 | 0.55 |
| CHEMBL471524 | OC[C@H]1O[C@H]([C@@H]([C@@H]1O)O)c1[nH]nc2c1ncnc2N | 267.24 | 2 | 7 | 5 | 62.74 | 150.4 | -1.26 | No | No | No | No | No | 0 | 1 | 1 | 1 | 1 | 0.55 |
| CHEMBL500386 | CC/C=C(/[C@H]1OC(=O)[C@H](C)N(C)C(=O)[C@@H](N=C(O)CN(C)C(=O)[C@H]([C@@H](CC)C)N(C(=O)[C@@H](N=C([C@H](OC(=O)/C(=C/C[C@@H]([C@@H]1C)O)/C)[C@H](CC)C)O)C(C)C)C)C(C)C)\C | 834.09 | 8 | 12 | 3 | 252.49 | 198.94 | 4.73 | No | No | No | No | No | 2 | 3 | 1 | 1 | 4 | 0.17 |
| CHEMBL464570 | O=C(C[C@@H](O[C@@H]1OC[C@H]([C@@H]([C@H]1O)O)O)CCc1ccc(c(c1)O)O)CCc1ccc(c(c1)O)O | 478.49 | 10 | 10 | 7 | 120.61 | 177.14 | 0.84 | No | No | No | No | No | 1 | 0 | 1 | 1 | 2 | 0.55 |
| CHEMBL526377 | OC[C@H]1O[C@@H](Oc2ccc3c(c2)oc(=O)cc3)[C@@H]([C@H]([C@@H]1O)O)O | 324.28 | 3 | 8 | 4 | 76.63 | 129.59 | -0.29 | No | No | No | No | No | 0 | 1 | 0 | 0 | 0 | 0.55 |
| CHEMBL1951300 | CC(=CCc1c(O)ccc2c1O[C@@H](CC2)c1ccc(cc1O)O)C | 326.39 | 3 | 4 | 3 | 94.87 | 69.92 | 3.61 | Yes | No | Yes | Yes | No | 0 | 0 | 0 | 0 | 0 | 0.55 |
| CHEMBL485477 | COc1cc2[C@H]3O[C@H]([C@@H]([C@@H]3C)C)c3c(c2c(c1OC)OC)c(OC)c1c(c3)OCO1 | 414.45 | 4 | 7 | 0 | 109.93 | 64.61 | 3.53 | No | No | No | Yes | No | 0 | 0 | 0 | 0 | 0 | 0.55 |
| CHEMBL492824 | CCCCCCCCCCCCCC(=O)O[C@H]([C@H]([C@H](C[C@H](c1coc(n1)Cc1cnco1)O)O)O)C | 508.65 | 21 | 9 | 3 | 137.09 | 139.05 | 4.51 | No | No | No | No | Yes | 1 | 3 | 1 | 1 | 2 | 0.55 |
| CHEMBL465959 | OCC(=O)[C@]1(C)CC[C@H]2C(=C1)CC[C@@H]1[C@]2(C)C=C(O)C(=O)C1(C)C | 332.43 | 2 | 4 | 2 | 93.32 | 74.6 | 2.79 | No | No | No | No | No | 0 | 0 | 0 | 0 | 0 | 0.55 |
| CHEMBL1668334 | CC(CC(=O)C[C@@H](c1ccc(cc1)C)C)C | 218.33 | 5 | 1 | 0 | 70.22 | 17.07 | 3.88 | No | No | No | Yes | No | 0 | 0 | 0 | 0 | 1 | 0.55 |
| CHEMBL515346 | OC[C@]12CC[C@@H](C[C@H]1CC[C@@H]1[C@@H]2CC[C@]2([C@]31O[C@@H]3C[C@@H]2c1ccc(=O)oc1)C)O | 400.51 | 2 | 5 | 2 | 108.83 | 83.2 | 3.07 | No | No | No | Yes | No | 0 | 0 | 0 | 0 | 0 | 0.55 |
| CHEMBL477533 | COC1=C(OC)C(=O)c2c(C1=O)c(O)cc(c2O)OC | 280.23 | 3 | 7 | 2 | 66.57 | 102.29 | 1.03 | Yes | No | No | No | No | 0 | 0 | 0 | 0 | 0 | 0.56 |
| CHEMBL452632 | CCCCCCCCCCCC[C@@H]([C@H]1CC[C@@H](O1)[C@@H](CCC[C@H]([C@@H](CCCCC[C@H](CC1=C[C@@H](OC1=O)C)O)O)O)O)O | 612.88 | 26 | 8 | 5 | 173.84 | 136.68 | 6.17 | No | No | No | No | Yes | 1 | 4 | 1 | 2 | 3 | 0.55 |
| CHEMBL3600945 | C/C=C(/C(=O)C[C@H]1[C@](C)(O)CC[C@@]2([C@]1(C)[C@@H](O)CCC2(C)C)O)\C | 338.48 | 3 | 4 | 3 | 96.79 | 77.76 | 2.79 | No | No | No | No | No | 0 | 0 | 0 | 0 | 0 | 0.55 |
| CHEMBL504221 | CC(=O)OC[C@@]1(OC[C@H]2O[C@H](COC(=O)C)[C@H]([C@@H]([C@H]2O)OC(=O)C)OC(=O)C)O[C@@H]([C@@H]([C@H]1O)OC(=O)C)COC(=O)/C=C/c1ccc(cc1)O | 712.65 | 20 | 18 | 3 | 163.02 | 246.18 | 0.4 | No | No | No | Yes | No | 2 | 4 | 2 | 1 | 4 | 0.17 |
| CHEMBL53418 | Oc1cccc2c1C(=O)c1c(C2=O)cccc1O | 240.21 | 0 | 4 | 2 | 63.8 | 74.6 | 2.04 | Yes | No | No | No | Yes | 0 | 0 | 0 | 0 | 0 | 0.55 |
| CHEMBL1253219 | Br/C=C/1\OC(=O)C=C1Br | 253.88 | 0 | 2 | 0 | 40.11 | 26.3 | 1.81 | No | No | No | No | No | 0 | 1 | 0 | 0 | 0 | 0.55 |
| CHEMBL1172613 | CC[C@H]([C@H]1O[C@H]([C@@H](C[C@@H]1C)C)c1c(O)c(cn(c1=O)OC)c1ccccc1)C | 385.5 | 5 | 4 | 1 | 112.61 | 60.69 | 4.02 | No | Yes | No | No | Yes | 0 | 0 | 0 | 0 | 0 | 0.55 |
| CHEMBL3560966 | CCOC(=O)[C@@H](O)C | 118.13 | 3 | 3 | 1 | 28.6 | 46.53 | 0.36 | No | No | No | No | No | 0 | 3 | 0 | 0 | 1 | 0.55 |
| CHEMBL400981 | CO[C@@H](C[C@H]([C@H]1CC[C@@]2([C@]1(C)CC[C@@]13[C@H]2C=C[C@]2([C@H]3CC[C@@H](C2(C)C)O[C@@H]2O[C@H](CO)[C@H]([C@H]([C@H]2O)O)O)OC1)C)C)C=C(C)C | 632.87 | 8 | 8 | 4 | 174.33 | 117.84 | 4.46 | No | No | No | No | No | 1 | 3 | 0 | 0 | 2 | 0.55 |
| CHEMBL487724 | CO[C@@H]1CC(=O)c2c3[C@]41Oc1ccc(c5c1[C@@](O4)(Oc3ccc2)[C@@H]1O[C@@H]1[C@@H]5O)O | 396.35 | 1 | 8 | 2 | 94.1 | 106.98 | 1.25 | No | No | No | Yes | Yes | 0 | 0 | 0 | 0 | 0 | 0.55 |
| CHEMBL514518 | CC1=CCC[C@@](C)(O)[C@H]2C[C@H](CC[C@]([C@@H](CC1)O)(C)O)[C@H](C(=O)O2)C | 354.48 | 0 | 5 | 3 | 98.4 | 86.99 | 2.38 | No | No | No | No | No | 0 | 0 | 0 | 0 | 0 | 0.55 |
| CHEMBL521975 | COc1cc2[C@@H](O)[C@@H](C)[C@@H](C)Cc3c(c2c(c1OC)OC)c(OC)c1c(c3)OCO1 | 416.46 | 4 | 7 | 1 | 112.12 | 75.61 | 3.47 | No | No | No | Yes | No | 0 | 0 | 0 | 0 | 0 | 0.55 |
| CHEMBL602899 | O=C(C[C@H](C(=O)O)C)C[C@H]([C@H]1C[C@@H]([C@@]2([C@]1(C)CC(=O)C1=C2[C@@H](O)C[C@@H]2[C@]1(C)CCC(=O)C2(C)C)C)O)C | 516.67 | 6 | 7 | 3 | 141.05 | 128.97 | 3.25 | No | No | No | No | Yes | 1 | 3 | 0 | 0 | 0 | 0.56 |
| CHEMBL516320 | OC[C@]1(C)CC[C@]2([C@@H](C1)[C@]1(C)CC[C@@]3([C@@H]([C@]1(CC2)C)CC[C@]1([C@H]3CCC(=O)[C@@H]1C)C)C)C | 442.72 | 1 | 2 | 1 | 135.56 | 37.3 | 6.57 | No | No | No | No | No | 1 | 3 | 0 | 1 | 1 | 0.55 |
| CHEMBL1089962 | O[C@@H]1[C@@H](COP(=O)(O)O)O[C@@]([C@H]1O)(O)COP(=O)(O)O | 340.12 | 6 | 12 | 7 | 57.59 | 223.06 | -3.62 | No | No | No | No | No | 2 | 1 | 1 | 1 | 4 | 0.11 |
| CHEMBL2419311 | CC(=CC(=Nc1cc2c3n1c1ccccc1c1c3c(c(c2)O)ncc1)O)C | 355.39 | 2 | 4 | 2 | 110.45 | 70.12 | 4.05 | Yes | Yes | Yes | No | No | 0 | 0 | 0 | 0 | 0 | 0.55 |
| CHEMBL1794791 | OC(=O)CC(=O)C(=O)O | 132.07 | 3 | 5 | 2 | 25.09 | 91.67 | -0.88 | No | No | No | No | No | 0 | 4 | 0 | 0 | 2 | 0.56 |
| CHEMBL37460 | CCCCCCCCCCCCCCCC(=N[C@H]([C@@H](/C=C/CC/C=C/CCCCCCCCC)O)CO)O | 535.88 | 29 | 4 | 3 | 172.18 | 73.05 | 9.44 | No | No | No | No | No | 2 | 4 | 1 | 1 | 2 | 0.17 |
| CHEMBL1079510 | C=C1CCC(=O)[C@@]2([C@@H]1C[C@H](CC2)C(O)(C)C)C | 236.35 | 1 | 2 | 1 | 70.66 | 37.3 | 2.72 | No | No | No | No | No | 0 | 0 | 0 | 0 | 0 | 0.55 |
| CHEMBL480268 | CC(=CCC1=C2OC(C)(C)C=CC2(C(=O)C(=C1O)C(=O)CC(C)C)CC=C(C)C)C | 412.56 | 7 | 4 | 1 | 123.33 | 63.6 | 5.2 | No | Yes | Yes | No | Yes | 0 | 1 | 0 | 1 | 1 | 0.85 |
| CHEMBL385256 | COc1cc(O)cc2c1c1ccc(cc1C2=O)O | 242.23 | 1 | 4 | 2 | 65.85 | 66.76 | 1.97 | Yes | No | No | No | Yes | 0 | 0 | 0 | 0 | 0 | 0.55 |
| CHEMBL481051 | OCC(=C)[C@@H]1CC[C@]2([C@H]1[C@H]1CC[C@H]3[C@@]([C@]1(C)CC2)(C)CC[C@@H]1[C@]3(C)CCC(=O)C1(C)C)C | 440.7 | 2 | 2 | 1 | 135.34 | 37.3 | 6.47 | No | No | No | No | No | 1 | 3 | 0 | 1 | 1 | 0.55 |
| CHEMBL388788 | COC(=O)CC(=O)O[C@@H]1CC[C@]2([C@H](C1(C)C)CC[C@@]1([C@@H]2CC[C@H]2[C@@]1(C)CC[C@@H]2[C@]1(C)CC[C@@H](O1)C(O)(C)C)C)C | 560.8 | 7 | 6 | 1 | 158.83 | 82.06 | 6.26 | No | No | No | No | No | 2 | 4 | 0 | 1 | 1 | 0.17 |
| CHEMBL464202 | COc1ccc2c(c1C)O[C@@H]([C@@H](C2)O)c1ccc(cc1)O | 286.32 | 2 | 4 | 2 | 79.72 | 58.92 | 2.5 | No | Yes | No | Yes | No | 0 | 0 | 0 | 0 | 0 | 0.55 |
| CHEMBL1077625 | COC1=CC(=O)[C@@]2([C@H](C1=O)CC=C([C@H]2C[C@H]1C(=C)C(=O)C[C@@H]2[C@]1(C)CCCC2(C)C)C)c1cc(=O)c2c(o1)cc(cc2O)OC | 614.72 | 5 | 8 | 1 | 172.12 | 120.11 | 5.24 | No | No | No | No | Yes | 1 | 4 | 0 | 1 | 2 | 0.56 |
| CHEMBL491368 | C=C[C@H]1CC[C@@H]2[C@]1(C)CC[C@H]1[C@H]2CC[C@@H]2[C@]1(CO)CC[C@@H](C2)O | 318.49 | 2 | 2 | 2 | 95.93 | 40.46 | 4.1 | No | No | Yes | No | No | 1 | 0 | 0 | 0 | 1 | 0.55 |
| CHEMBL462719 | COc1cc(C/C=C/c2ccc(c(c2OC)O)OC)ccc1O | 316.35 | 6 | 5 | 2 | 89.35 | 68.15 | 3.18 | Yes | Yes | Yes | Yes | Yes | 0 | 0 | 0 | 0 | 0 | 0.55 |
| CHEMBL452234 | COc1c(C(=O)CCc2ccccc2)c(O)c(c(c1C)O)C | 300.35 | 5 | 4 | 2 | 86.4 | 66.76 | 3.33 | Yes | Yes | No | Yes | Yes | 0 | 0 | 0 | 0 | 0 | 0.55 |
| CHEMBL1097683 | COc1c(oc2c(c1=O)c(O)cc(c2)OC)c1ccccc1 | 298.29 | 3 | 5 | 1 | 82.93 | 68.9 | 2.87 | Yes | Yes | Yes | Yes | Yes | 0 | 0 | 0 | 0 | 0 | 0.55 |
| CHEMBL2409150 | CCC[C@H]1O[C@H](CC(=O)OC)[C@@H](C2=C1C(=O)c1c(C2=O)cccc1O)O | 360.36 | 5 | 7 | 2 | 90.95 | 110.13 | 1.64 | No | No | No | No | No | 0 | 0 | 0 | 0 | 0 | 0.55 |
| CHEMBL484440 | COc1ccc(cc1)c1c(C)c(C)cc2c1cc(O)cc2 | 278.35 | 2 | 2 | 1 | 87.83 | 29.46 | 4.41 | Yes | Yes | No | Yes | No | 0 | 0 | 0 | 0 | 1 | 0.55 |
| CHEMBL1669578 | O=C(N1CCCC1)/C=C/C=C/CC/C=C/c1ccc2c(c1)OCO2 | 325.4 | 7 | 3 | 0 | 99.42 | 38.77 | 3.84 | Yes | Yes | Yes | No | Yes | 0 | 0 | 0 | 0 | 0 | 0.55 |
| CHEMBL1735595 | C=C[C@@]1(C)CC[C@@H]2C(=C1)CC[C@H]1[C@@]2(C)CCC[C@@]1(C)C(=O)O | 302.45 | 2 | 2 | 1 | 91.96 | 37.3 | 4.57 | No | Yes | Yes | No | No | 1 | 0 | 0 | 0 | 1 | 0.85 |
| CHEMBL2331810 | COc1c(c(O)cc(c1O)c1ccccc1)c1ccccc1 | 292.33 | 3 | 3 | 2 | 87.85 | 49.69 | 3.87 | Yes | Yes | Yes | Yes | No | 0 | 0 | 0 | 0 | 0 | 0.55 |
| CHEMBL2409054 | COc1cc2O[C@@H](CCc2cc1OC)c1ccc(c(c1)OC)O | 316.35 | 4 | 5 | 1 | 86.58 | 57.15 | 3.02 | Yes | Yes | No | Yes | Yes | 0 | 0 | 0 | 0 | 0 | 0.55 |
| CHEMBL2332433 | CC(=C1C[C@@]2(OC1=O)[C@@H](C)CC[C@H]2C(=C)C)C | 234.33 | 1 | 2 | 0 | 70.37 | 26.3 | 3.56 | No | Yes | Yes | No | No | 0 | 0 | 0 | 0 | 0 | 0.55 |
| CHEMBL478797 | Cn1c(O)nc2c1c(NCCS(=O)(=O)O)ncn2 | 273.27 | 4 | 7 | 3 | 63.26 | 138.61 | -0.43 | No | No | No | No | No | 0 | 0 | 0 | 1 | 0 | 0.56 |
| CHEMBL476679 | COc1cc(cc(c1OC)OC)[C@H]1[C@@H]2C(=O)OC[C@H]2Cc2c1cc1OCOc1c2 | 398.41 | 4 | 7 | 0 | 102.68 | 72.45 | 3.08 | No | Yes | Yes | Yes | Yes | 0 | 0 | 0 | 0 | 0 | 0.55 |
| CHEMBL1092399 | COC1=CC(=NC1=CNCCC(C)C)c1ccc[nH]1 | 259.35 | 6 | 2 | 2 | 81.66 | 49.41 | 2.43 | Yes | Yes | No | No | No | 0 | 0 | 0 | 0 | 0 | 0.55 |
| CHEMBL22053 | CN1CCc2c(C1)cccc2 | 147.22 | 0 | 1 | 0 | 50.69 | 3.24 | 1.92 | No | No | No | No | No | 0 | 1 | 0 | 0 | 2 | 0.55 |
| CHEMBL428082 | C[C@H]([C@H](c1ccc2c(c1)OCO2)O)[C@@H](Cc1ccc2c(c1)OCO2)C | 342.39 | 5 | 5 | 1 | 93.22 | 57.15 | 3.7 | No | Yes | Yes | Yes | No | 0 | 0 | 0 | 0 | 0 | 0.55 |
| CHEMBL463570 | O[C@H]1C[C@H](C)[C@@]2(C(=C1)CC[C@@H]([C@@H]2C(=O)C)O)C | 238.32 | 1 | 3 | 2 | 66.97 | 57.53 | 1.53 | No | No | No | No | No | 0 | 0 | 0 | 0 | 0 | 0.55 |
| CHEMBL219937 | Brc1cc(O)c(c(c1)Br)Oc1cc(Br)cc(c1O)Br | 517.79 | 2 | 3 | 2 | 87.8 | 49.69 | 4.86 | No | No | Yes | No | No | 2 | 2 | 0 | 1 | 1 | 0.17 |
| CHEMBL2152446 | OC[C@@]1(O)CO[C@H]([C@@H]1O)OC[C@H]1O[C@@H](OC[C@@]23OCC(=C)[C@H]3CC=C2C)[C@@H]([C@H]([C@@H]1O)O)O | 460.47 | 7 | 11 | 6 | 106.13 | 167.53 | -1.3 | No | No | No | No | No | 2 | 1 | 1 | 1 | 4 | 0.17 |
| CHEMBL2347638 | CCCCCCCCCCCCCCC/C=C/C=C/C(=NCC(C)C)O | 363.62 | 18 | 2 | 1 | 121.79 | 32.59 | 7.73 | Yes | No | No | No | No | 1 | 2 | 1 | 1 | 2 | 0.55 |
| CHEMBL559282 | CCC/C=C/C=C/C1=C(CO)[C@@H](O)[C@@H]2[C@@](C1=O)(C/C=C(/C(=O)O)\C)O2 | 348.39 | 8 | 6 | 3 | 92.74 | 107.36 | 1.86 | No | No | No | No | No | 0 | 0 | 0 | 0 | 0 | 0.56 |
| CHEMBL479669 | OC/C=C/c1cc(OC)cc(c1OC)O[C@@H]1O[C@H](CO)[C@H]([C@@H]([C@H]1O)O)O | 372.37 | 7 | 9 | 5 | 89.63 | 138.07 | -0.41 | No | No | No | No | No | 0 | 1 | 0 | 1 | 0 | 0.55 |
| CHEMBL2442459 | OC[C@H]1O[C@@H](O[C@@H]2[C@@H](Oc3c2cc(cc3)C(=O)C)C(=C)CO)[C@@H]([C@H]([C@@H]1O)O)O | 396.39 | 6 | 9 | 5 | 94.63 | 145.91 | -0.55 | No | No | No | No | No | 0 | 1 | 1 | 1 | 0 | 0.55 |
| CHEMBL568737 | CC(CC(=O)O)C | 102.13 | 2 | 2 | 1 | 27.92 | 37.3 | 0.98 | No | No | No | No | No | 0 | 3 | 0 | 0 | 1 | 0.85 |
| CHEMBL1169628 | COc1cc2CCN([C@H]3c2c(c1O)Oc1ccc(cc1)C[C@H]1N(C)CCc2c1c(Oc1ccc(C3)cc1)c(O)c(c2)OC)C | 594.7 | 2 | 8 | 2 | 177.14 | 83.86 | 4.79 | No | No | No | No | No | 1 | 3 | 0 | 0 | 1 | 0.55 |
| CHEMBL109034 | OC/C=C/c1ccc(cc1)O | 150.17 | 2 | 2 | 2 | 44.52 | 40.46 | 1.45 | No | No | No | No | No | 0 | 1 | 0 | 0 | 1 | 0.55 |
| CHEMBL382408 | O=C(c1cc(O)c(c(c1)O)O)O[C@@H]1O[C@H](COC(=O)c2cc(O)c(c(c2)O)O)[C@H]([C@@H]([C@H]1OC(=O)c1cc(O)c(c(c1)O)O)OC(=O)c1cc(O)c(c(c1)O)O)OC(=O)c1cc(O)c(c(c1)O)O | 940.68 | 16 | 26 | 15 | 214.27 | 444.18 | 0.22 | No | No | No | No | No | 3 | 3 | 2 | 1 | 5 | 0.17 |
| CHEMBL132827 | OC(=O)/C=C(\c1c2OC(C)(C)C=Cc2c2c(c1O)C(=O)[C@@H]([C@H](O2)C)C)/c1ccccc1 | 420.45 | 3 | 6 | 2 | 117.93 | 93.06 | 3.99 | No | No | Yes | No | Yes | 0 | 0 | 0 | 0 | 1 | 0.56 |
| CHEMBL452113 | OC[C@@H]1O[C@H]([C@@H]([C@H]1O)OC(=O)c1cc(O)c(c(c1)O)O)OC[C@H]1O[C@@H](OC([C@H]2CCC(=CC2)C)(C)C)[C@@H]([C@H]([C@@H]1O)O)O | 600.61 | 10 | 14 | 8 | 143.3 | 225.06 | -0.1 | No | No | No | No | No | 3 | 3 | 1 | 1 | 4 | 0.17 |
| CHEMBL2386704 | CC(=O)O[C@H]1CCC(C2=CC(=O)c3c([C@@]12C)cc1occc1c3C)(C)C | 352.42 | 2 | 4 | 0 | 100.62 | 56.51 | 4.14 | No | Yes | Yes | No | Yes | 0 | 0 | 0 | 0 | 0 | 0.55 |
| CHEMBL2333540 | O=CN1c2ccccc2[C@@]2([C@H]1C=C1CC[C@@H]3[C@]([C@H]1C2)(C)CCCC3(C)C)C[C@@H]1N=C(O)[C@@H](N(C1=O)C)C(C)C | 531.73 | 4 | 4 | 1 | 168.42 | 73.21 | 5.13 | No | No | Yes | No | Yes | 2 | 3 | 0 | 0 | 1 | 0.17 |
| CHEMBL105184 | COC(=O)c1cc(OC)c2c(c1c1c(cc(c3c1OCO3)OC)C(=O)OC)OCO2 | 418.35 | 7 | 10 | 0 | 99.55 | 107.98 | 2.63 | Yes | Yes | Yes | No | Yes | 0 | 0 | 0 | 0 | 0 | 0.55 |
| CHEMBL480675 | OC[C@]12CCCC([C@H]2C[C@H]([C@]23[C@H]1C[C@H](O)[C@H]([C@H]2O)C(=C)C3=O)O)(C)C | 350.45 | 1 | 5 | 4 | 93.39 | 97.99 | 1.47 | No | No | No | No | No | 0 | 0 | 0 | 0 | 0 | 0.55 |
| CHEMBL239211 | C[C@H](c1oc2c(c1)C(=O)c1c(C2=O)cccc1O)O | 258.23 | 1 | 5 | 2 | 64.97 | 87.74 | 1.47 | No | No | No | No | No | 0 | 0 | 0 | 0 | 0 | 0.55 |
| CHEMBL2035090 | COC(=O)C[C@@H]1[C@](C)(C=CC(=O)C1(C)C)[C@H]1[C@@H](OC(=O)C)[C@H](OC(=O)C)[C@@]2([C@]3(C1=C)O[C@@H]3C[C@H]2C1=CC(=O)OC1O)C | 586.63 | 9 | 11 | 1 | 145.98 | 155.03 | 2.36 | No | No | No | No | No | 2 | 3 | 1 | 1 | 2 | 0.17 |
| CHEMBL462795 | CC(=C)[C@@H]1CC[C@]2([C@H]1[C@H]1CC[C@H]3[C@@]([C@]1(C)C[C@@H]2O)(C)CC[C@@H]1[C@]3(C)CC[C@@H](C1(C)C)O)C | 442.72 | 1 | 2 | 2 | 136.3 | 40.46 | 6.34 | No | No | No | No | No | 1 | 3 | 0 | 1 | 1 | 0.55 |
| CHEMBL1271888 | Oc1ccc2c(c1)occ(c2=O)c1ccc2c(c1)C=CC(O2)(C)C | 320.34 | 1 | 4 | 1 | 94.06 | 59.67 | 3.53 | Yes | Yes | Yes | No | Yes | 0 | 0 | 0 | 0 | 0 | 0.55 |
| CHEMBL1969511 | COc1cc2oc(c3ccc4c(c3)OCO4)c(c(=O)c2c(c1OC)OC)OC | 386.35 | 5 | 8 | 0 | 99.95 | 85.59 | 2.91 | No | Yes | Yes | Yes | Yes | 0 | 0 | 0 | 0 | 0 | 0.55 |
| CHEMBL564146 | COCn1c2ccccc2c2c1CC[C@@]1(CC)CCCN(CC2)C1 | 326.48 | 3 | 2 | 0 | 104.61 | 17.4 | 3.79 | No | No | No | Yes | Yes | 0 | 0 | 0 | 0 | 0 | 0.55 |
| CHEMBL3039078 | OC(=O)[C@@H]([C@](C(=O)O)(Cc1ccc(cc1)O)O)O | 256.21 | 5 | 7 | 5 | 58.56 | 135.29 | -0.4 | No | No | No | No | No | 0 | 1 | 0 | 1 | 0 | 0.56 |
| CHEMBL428593 | CCCCCCCCC=CCCCCCCCC(=O)OCC(CO)O | 356.54 | 19 | 4 | 2 | 106.2 | 66.76 | 5.07 | Yes | No | No | Yes | No | 0 | 0 | 1 | 0 | 2 | 0.55 |
| CHEMBL1642040 | COC(=O)CC[C@@]1(C)[C@@H](CC[C@@]2([C@@H]1CC=C1[C@@]2(C)CC[C@@]2([C@H]1C[C@](C)(CC2)C(=O)O)C)C)C(=C)C | 484.71 | 6 | 4 | 1 | 143.48 | 63.6 | 6.48 | No | No | Yes | No | No | 1 | 4 | 0 | 1 | 1 | 0.85 |
| CHEMBL511768 | OC/C=C(\C(=O)O)/CC[C@@H]1C(=C)CC[C@H]2[C@@]1(C)CC[C@H]([C@@]2(C)CO)O | 352.47 | 6 | 5 | 4 | 97.82 | 97.99 | 2.55 | No | No | No | No | No | 0 | 0 | 0 | 0 | 0 | 0.56 |
| CHEMBL2334483 | CC(=O)O[C@H]1CC[C@@]2([C@@H](C1(C)C)C(=O)C[C@]13[C@H]2[C@@H](O)[C@H](O)[C@H](C1)C(=C)C3=O)C | 390.47 | 2 | 6 | 2 | 102.17 | 100.9 | 1.82 | No | No | No | No | No | 0 | 0 | 0 | 0 | 0 | 0.55 |
| CHEMBL556108 | O=C[C@]12C[C@H]3O[C@@]4(O)[C@H](O)C[C@H](O[C@H]4O[C@@H]3C[C@@H]1CC[C@@H]1[C@@H]2CC[C@]2([C@]1(O)C[C@H]([C@@H]2C1=CC(=O)OC1)O)C)C | 548.62 | 2 | 10 | 4 | 135.19 | 151.98 | 1.05 | No | No | No | No | No | 1 | 3 | 1 | 1 | 1 | 0.55 |
| CHEMBL498836 | OC[C@H]1O[C@@H](O[C@H]2CC[C@]3([C@H](C2(C)C)CC[C@@]2([C@@H]3CC=C3[C@@]2(C)C[C@H]([C@@]2([C@H]3CC(C)(C)CC2)C(=O)O[C@@H]2OC[C@@H]([C@@H]([C@H]2O)O)O)O)C)C)[C@@H]([C@H]([C@@H]1O)O)O | 766.95 | 6 | 13 | 8 | 196.2 | 215.83 | 2.26 | No | No | No | No | No | 3 | 3 | 1 | 1 | 4 | 0.17 |
| CHEMBL476286 | C=C[C@@]1(C)CC[C@@H](C[C@@H]1C(=C)C)/C(=C/C[C@H](C(O)(C)C)OC(=O)C)/C | 348.52 | 8 | 3 | 1 | 106.56 | 46.53 | 4.86 | No | Yes | Yes | Yes | Yes | 0 | 0 | 0 | 0 | 1 | 0.55 |
| CHEMBL430928 | Cn1c2nc(n(c2c(nc1=O)O)C)O | 196.16 | 0 | 5 | 2 | 48.36 | 93.17 | -0.47 | No | No | No | No | No | 0 | 1 | 0 | 0 | 1 | 0.55 |
| CHEMBL239028 | COc1ccc(cc1)c1coc2c(c1=O)cc(c(c2)O)O | 284.26 | 2 | 5 | 2 | 78.46 | 79.9 | 2.3 | Yes | No | No | Yes | Yes | 0 | 0 | 0 | 0 | 0 | 0.55 |
| CHEMBL519761 | CC(=O)O[C@H]1[C@@H](O[C@H]([C@@H]([C@H]1OC(=O)C)OC(=O)C)C)Oc1c(oc2c(c1=O)c(O)cc(c2)O)c1ccc(cc1)O | 558.49 | 9 | 13 | 3 | 136.18 | 188.26 | 1.97 | No | No | No | No | No | 2 | 2 | 1 | 1 | 2 | 0.17 |
| CHEMBL481057 | COc1cc2c(cc1OC)CC[C@@H]1[C@@]2(O)C(=O)c2c(O1)cc1c(c2)cco1 | 366.36 | 2 | 6 | 1 | 96.96 | 78.13 | 2.82 | No | Yes | Yes | Yes | Yes | 0 | 0 | 0 | 0 | 0 | 0.55 |
| CHEMBL489557 | COC(=O)c1[nH]cc(c1c1c[nH]c2c1cc(Cl)cc2)c1c[nH]c2c1cc(Cl)c(c2)Cl | 458.72 | 4 | 2 | 3 | 121.69 | 73.67 | 5.49 | Yes | Yes | No | Yes | Yes | 0 | 1 | 0 | 1 | 1 | 0.55 |
| CHEMBL591023 | Oc1cc(O)c2c(c1)occ(c2=O)c1ccc2c(c1)OCO2 | 298.25 | 1 | 6 | 2 | 78.03 | 89.13 | 2.29 | Yes | No | Yes | Yes | Yes | 0 | 0 | 0 | 0 | 0 | 0.55 |
| CHEMBL457149 | CC(=O)O[C@H]1CCC([C@]2([C@@]1(C)[C@H]1Cc3occc3[C@@H]3[C@@H]1[C@H](C2)OC3=O)O)(C)C | 388.45 | 2 | 6 | 1 | 100.35 | 85.97 | 2.77 | No | No | No | Yes | No | 0 | 0 | 0 | 0 | 0 | 0.55 |
| CHEMBL1278178 | CC[C@@H](C(C)C)CC[C@H]([C@H]1CC[C@@H]2[C@]1(C)CC[C@H]1[C@H]2C[C@H]([C@@]2([C@]1(C)CC[C@@H](C2)O)O)O)C | 448.72 | 6 | 3 | 3 | 136.06 | 60.69 | 5.85 | No | No | No | No | No | 1 | 3 | 0 | 1 | 1 | 0.55 |
| CHEMBL1553601 | O=c1ccc2c(o1)c1CC(=O)C(Oc1cc2)(C)C | 244.24 | 0 | 4 | 0 | 66.49 | 56.51 | 2.26 | Yes | Yes | No | No | No | 0 | 0 | 0 | 0 | 0 | 0.55 |
| CHEMBL557698 | C=C[C@@]1(C)CC[C@H]2[C@@](C1)(O)CC[C@@H]1[C@]2(C)CCC[C@]1(C)C(=O)O | 320.47 | 2 | 3 | 2 | 93.63 | 57.53 | 3.96 | No | No | Yes | No | No | 0 | 0 | 0 | 0 | 0 | 0.85 |
| CHEMBL505286 | O[C@@H](C#C)COc1ccc(cc1)C(=O)O | 206.19 | 4 | 4 | 2 | 53.64 | 66.76 | 1.34 | No | No | No | No | No | 0 | 0 | 0 | 0 | 0 | 0.85 |
| CHEMBL517016 | COC1=C[C@@H](C)[C@H]2[C@@](C1=O)(C)[C@H]1C(=O)C(=C([C@H]3[C@@]1([C@@H](C2)OC(=O)C3)C)C)OC | 388.45 | 2 | 6 | 0 | 101.8 | 78.9 | 2.4 | No | No | No | No | No | 0 | 0 | 0 | 0 | 0 | 0.55 |
| CHEMBL468863 | COC1=CC(=O)c2c(C1=O)c1c(cc2)cc(c(c1O)OC)OC | 314.29 | 3 | 6 | 1 | 82.65 | 82.06 | 2.03 | Yes | Yes | Yes | No | Yes | 0 | 0 | 0 | 0 | 0 | 0.56 |
| CHEMBL510341 | COc1cc(/C=C/C(=O)OC[C@H]2O[C@@H](Oc3c(oc4c(c3=O)c(O)cc(c4)O)c3ccc(cc3)O)[C@@H]([C@H]([C@@H]2O)OC(=O)C=Cc2ccc(cc2)O)O)ccc1O | 770.69 | 13 | 16 | 7 | 197.37 | 252.11 | 3.04 | No | No | Yes | No | No | 3 | 3 | 2 | 1 | 4 | 0.17 |
| CHEMBL508425 | CCCCCCCCCCCCCCCC(=O)O[C@H]1[C@H](O)[C@H](Cc2oc([C@@H]([C@H]3C=C1C(=O)O3)C(=C)C)cc2C(=O)OC)C(=C)C | 626.82 | 20 | 8 | 1 | 177.4 | 112.27 | 7.49 | No | No | No | No | Yes | 2 | 4 | 1 | 1 | 3 | 0.17 |
| CHEMBL480874 | O=C1O[C@H](C(=C1)[C@@H]1OCC(=CC1)CC[C@@]1(C)[C@@H](C)CC[C@@]2([C@@H]1CCC=C2C)C)O | 400.55 | 4 | 4 | 1 | 115.42 | 55.76 | 4.42 | No | No | Yes | No | Yes | 1 | 0 | 0 | 0 | 0 | 0.55 |
| CHEMBL427288 | O[C@@H]1C[C@H](CCCCCCCCCCCCc2ccc3c(c2)OCO3)OC2=C1C(=O)CCC2 | 456.61 | 13 | 5 | 1 | 131.36 | 64.99 | 6.01 | No | No | No | Yes | Yes | 0 | 3 | 1 | 1 | 1 | 0.85 |
| CHEMBL425554 | CC(=O)O[C@H]1[C@H]2[C@H](OC(=O)[C@H]2C)C[C@H]([C@H]2[C@@]1(C)C(=O)C=C2)C | 306.35 | 2 | 5 | 0 | 79.53 | 69.67 | 1.88 | No | No | No | No | No | 0 | 0 | 0 | 0 | 0 | 0.55 |
| CHEMBL126079 | Oc1cc(O)c2c(c1)O[C@@H]([C@H](C2)OC(=O)c1cc(O)c(c(c1)O)O)c1cc(O)c(c(c1)O)O | 458.37 | 4 | 11 | 8 | 112.06 | 197.37 | 1.04 | No | No | No | No | No | 2 | 0 | 1 | 1 | 3 | 0.17 |
| CHEMBL1077834 | OC[C@H]1O[C@@H](OCCc2ccc(c(c2)O)O)[C@@H]([C@H]([C@@H]1OC(=O)/C=C/c1ccc(c(c1)O)O)O[C@@H]1OC[C@]([C@H]1O)(O)CO)O[C@@H]1OC[C@H]([C@@H]([C@H]1O)O)O | 742.68 | 14 | 19 | 11 | 170.07 | 304.21 | -1.72 | No | No | No | No | No | 3 | 4 | 2 | 1 | 5 | 0.17 |
| CHEMBL1627201 | S=C=NCCCC[S@@](=O)C | 177.29 | 5 | 2 | 0 | 48.4 | 80.73 | 1.94 | No | No | No | No | No | 0 | 0 | 0 | 0 | 1 | 0.55 |
| CHEMBL480657 | OC[C@H]1O[C@@H](Oc2cc3oc(cc(=O)c3c(c2OC)O)c2ccc(cc2)O)[C@@H]([C@H]([C@@H]1O)O)O | 462.4 | 5 | 11 | 6 | 112.6 | 179.28 | 0.46 | No | No | No | No | No | 2 | 0 | 1 | 1 | 3 | 0.17 |
| CHEMBL512168 | COc1c(CC=C(C)C)c(/C=C/c2ccccc2)cc(c1CC=C(C)C)O | 362.5 | 7 | 2 | 1 | 117.77 | 29.46 | 6.21 | Yes | Yes | No | Yes | Yes | 1 | 1 | 0 | 1 | 1 | 0.55 |
| CHEMBL1970129 | O=C1CC2OCC=C3C4C2C2N1c1ccccc1C12CCN(C1(C4)O)C3 | 350.41 | 0 | 4 | 1 | 102.25 | 53.01 | 1.65 | No | No | No | Yes | No | 0 | 0 | 0 | 0 | 0 | 0.55 |
| CHEMBL524906 | Oc1ccc(cc1)[C@@H]1[C@@H](c2cc(O)cc3c2[C@H](c2cc(O)cc(c2)O)[C@H](O3)c2ccc(cc2)O)c2c3[C@H]1[C@H](c1ccc(cc1)O)c1c(O)cc(cc1[C@@H]1c3c(cc2O)O[C@H]1c1ccc(cc1)O)O | 906.93 | 6 | 12 | 10 | 252.25 | 220.76 | 6.55 | No | No | No | No | No | 3 | 4 | 1 | 2 | 6 | 0.17 |
| CHEMBL506398 | OC[C@H]1O[C@@H](Oc2cc3O[C@@H](CC(=O)c3c(c2)O)c2ccc(c(c2)O)OC)[C@@H]([C@H]([C@@H]1O)O)O[C@@H]1O[C@@H](C)[C@@H]([C@H]([C@H]1O)O)O | 610.56 | 7 | 15 | 8 | 141.41 | 234.29 | -1.02 | No | No | No | No | No | 3 | 4 | 1 | 1 | 4 | 0.17 |
| CHEMBL398304 | CC[C@H]1OC(=O)[C@H](Cc2ccccc2)N(C)C(=O)[C@H](OC(=O)[C@@H](N(C(=O)[C@H](OC(=O)[C@@H](N(C1=O)C)Cc1ccccc1)CC)C)Cc1ccccc1)C(C)C | 755.9 | 9 | 9 | 0 | 218.52 | 139.83 | 4.75 | No | No | No | No | No | 2 | 3 | 0 | 1 | 2 | 0.17 |
| CHEMBL6509 | NCc1c[nH]c2c1cccc2 | 146.19 | 1 | 1 | 2 | 45.97 | 41.81 | 1.32 | Yes | No | No | No | No | 0 | 1 | 0 | 0 | 1 | 0.55 |
| CHEMBL1271155 | O[C@@H]1[C@H](Oc2c(C1=O)c(O)c(c(c2C)O)C)c1ccccc1 | 300.31 | 1 | 5 | 3 | 80.64 | 86.99 | 2.1 | No | No | No | Yes | No | 0 | 0 | 0 | 0 | 0 | 0.55 |
| CHEMBL1642567 | COc1cc(ccc1OC12OCOC1=CC(=O)[C@H]([C@@H]2O)[C@@H]1OC[C@H]2[C@@H]1CO[C@H]2c1ccc(c(c1)OC)OC)[C@H]1OC[C@@H]2[C@H]1CO[C@H]2c1ccc(c(c1)OC)O | 760.78 | 10 | 14 | 2 | 191.34 | 159.06 | 2.95 | No | No | No | No | No | 2 | 3 | 1 | 1 | 4 | 0.11 |
| CHEMBL604041 | OC(=O)CCC(=O)OC[C@]1(C)CCC[C@]2([C@H]1CCc1c2ccc(c1)C(O)(C)C)C | 402.52 | 7 | 5 | 2 | 112.94 | 83.83 | 3.98 | No | No | No | Yes | Yes | 0 | 0 | 0 | 0 | 0 | 0.56 |
| CHEMBL471186 | Oc1cc(O)cc(c1)Oc1cc(O)c2c(c1)Oc1c(O2)c(Oc2cc(O)cc(c2)O)c(cc1O)O | 480.38 | 4 | 11 | 7 | 120.22 | 178.53 | 2.7 | No | No | Yes | No | Yes | 2 | 1 | 1 | 1 | 3 | 0.17 |
| CHEMBL481047 | CC(=CCc1c(O)cc(c(c1O)C(=O)c1ccccc1)O)CCC=C(C)C | 366.45 | 7 | 4 | 3 | 109.67 | 77.76 | 4.74 | Yes | No | Yes | No | Yes | 0 | 0 | 0 | 0 | 1 | 0.55 |
| CHEMBL255684 | CC(=O)[C@H]1[C@H](C[C@@H](C2=CCC[C@@H]([C@@]12C)C)O)C(=O)C | 264.36 | 2 | 3 | 1 | 75.63 | 54.37 | 2.1 | No | No | No | No | No | 0 | 0 | 0 | 0 | 0 | 0.55 |
| CHEMBL518044 | OC(=O)[C@@H]1C[C@H](O)[C@]2([C@@H]([C@H]1C)C1=CC[C@H]3[C@@]([C@@]1(CC2)C)(C)CC[C@@H]1[C@]3(C)CCC(=O)C1(C)C)C | 470.68 | 1 | 4 | 2 | 137.11 | 74.6 | 5.15 | No | No | No | No | No | 1 | 3 | 0 | 1 | 1 | 0.85 |
| CHEMBL479686 | COC(=O)/C=C/[C@H]([C@H]1CC[C@@H]2[C@]1(C)CC[C@H]1[C@H]2CCC2=CC(=O)C=C[C@]12C)C | 382.54 | 4 | 3 | 0 | 113.38 | 43.37 | 4.92 | No | No | Yes | No | Yes | 1 | 0 | 0 | 0 | 1 | 0.55 |
| CHEMBL463561 | CC([C@H](CC[C@H]([C@H]1C[C@H]([C@@H]2[C@]1(C)CC[C@H]1[C@@]2(O)C[C@@H]([C@@H]2[C@]1(C)CC[C@@H]([C@@H]2O)O)O)O)C)O)C | 468.67 | 5 | 6 | 6 | 129.94 | 121.38 | 2.64 | No | No | No | No | No | 1 | 1 | 0 | 0 | 1 | 0.55 |
| CHEMBL2437370 | COc1c(/C=C/C(=O)c2ccc(c(c2)O)O)ccc(c1CC=C(C)C)O | 354.4 | 6 | 5 | 3 | 102.53 | 86.99 | 3.64 | Yes | No | Yes | No | Yes | 0 | 0 | 0 | 0 | 0 | 0.55 |
| CHEMBL512767 | COc1c(oc2c(c1=O)c(O)c(c(c2OC)OC)OC)c1ccc(cc1)O | 374.34 | 5 | 8 | 2 | 97.93 | 107.59 | 2.46 | Yes | No | Yes | No | Yes | 0 | 0 | 0 | 0 | 0 | 0.55 |
| CHEMBL448502 | C=C[C@]1(C)CC[C@H](C[C@H]1C(=C)C)C(=C)C | 204.35 | 3 | 0 | 0 | 70.42 | 0 | 4.65 | No | Yes | Yes | No | No | 1 | 0 | 0 | 0 | 2 | 0.55 |
| CHEMBL1077108 | CC(=CCC/C(=C/CCc1cocc1)/C)CCCC1=CC(=O)OC1 | 328.45 | 10 | 3 | 0 | 98.34 | 39.44 | 4.86 | No | Yes | Yes | Yes | No | 0 | 0 | 0 | 0 | 1 | 0.55 |
| CHEMBL499113 | OC[C@H]1O[C@@H](Oc2cc(O)c3c(c2)O[C@@H](CC3=O)c2ccc(c(c2)O)O)[C@@H]([C@H]([C@@H]1O)O)O[C@@H]1O[C@H](CO)[C@H]([C@@H]([C@H]1O)O)O | 612.53 | 7 | 16 | 10 | 138.1 | 265.52 | -1.73 | No | No | No | No | No | 3 | 4 | 1 | 1 | 4 | 0.17 |
| CHEMBL387663 | OC(=NCCCCCN(C(=O)CC(C)C)O)CCC(=O)O | 302.37 | 12 | 6 | 3 | 80.27 | 110.43 | 1.56 | No | No | No | No | No | 0 | 0 | 1 | 0 | 0 | 0.56 |
| CHEMBL191935 | CCCCCC1CCC(=O)O1 | 156.22 | 4 | 2 | 0 | 44.55 | 26.3 | 2.24 | No | No | No | No | No | 0 | 1 | 0 | 0 | 1 | 0.55 |
| CHEMBL80139 | CC(=O)OC[C@@H]1[C@@H](OC(=O)C)C[C@@H]([C@@]2([C@@H]1[C@H](O)[C@@H]1C[C@H](OC(=O)C)C(=C(C1(C)C)[C@H]([C@@H]2OC(=O)C)OC(=O)C)C)C)OC(=O)C | 638.7 | 13 | 13 | 1 | 157.47 | 178.03 | 2.45 | No | No | No | Yes | No | 2 | 3 | 2 | 1 | 3 | 0.17 |
| CHEMBL556677 | OC/C(=C\1/Oc2c(C1=O)c(OC)ccc2)/[C@@H]1OC(=O)C[C@H]1C | 304.29 | 3 | 6 | 1 | 76.21 | 82.06 | 1.55 | No | No | No | No | No | 0 | 0 | 0 | 0 | 0 | 0.55 |
| CHEMBL255712 | CC(=O)O[C@]12OC[C@@]3([C@H]1[C@](C)([C@@H](CC3=O)O)[C@@H]1[C@](C2=O)(C)[C@@]23O[C@@H]2C[C@H]([C@@]3([C@H]([C@@H]1O)OC(=O)C(C)C)C)c1ccoc1)C | 600.65 | 6 | 11 | 2 | 147.28 | 162.1 | 2.19 | No | No | No | Yes | No | 2 | 3 | 1 | 1 | 3 | 0.17 |
| CHEMBL2011671 | COc1cc(O)c2c(c1)C(=O)c1c(C2=O)c(c(c(c1)C)O)c1c(OC)cc2c(c1O)C(=O)C1=C(C2=O)[C@@H](O)[C@]([C@@H]([C@H]1O)O)(C)O | 618.54 | 3 | 13 | 7 | 153.61 | 228.35 | 1.05 | No | No | Yes | No | No | 3 | 3 | 1 | 1 | 4 | 0.17 |
| CHEMBL454588 | C/C=C(/c1cc(O)c(c(c1)OC(=O)c1c(cc(c(c1O)C)O)/C(=C/C)/C)C)\C | 382.45 | 5 | 5 | 3 | 113.25 | 86.99 | 5.06 | No | No | Yes | No | No | 0 | 0 | 0 | 0 | 1 | 0.55 |
| CHEMBL1084896 | O[C@H]1CCCc2cc(O)cc(c2C(=O)O[C@@H](C1)C)O | 266.29 | 0 | 5 | 3 | 69.82 | 86.99 | 1.69 | No | No | No | No | No | 0 | 0 | 0 | 0 | 0 | 0.55 |
| CHEMBL771 | N[C@@H]1CON=C1O | 102.09 | 0 | 4 | 2 | 26.8 | 67.84 | -0.53 | No | No | No | No | No | 0 | 4 | 0 | 0 | 2 | 0.55 |
| CHEMBL1079033 | O=C1OCC(=C1)CC[C@@]1(O)[C@H](C)C[C@H]([C@@H]2[C@]1(C)CCCC2(C)C)O | 336.47 | 3 | 4 | 2 | 94.56 | 66.76 | 3.22 | No | No | No | No | No | 0 | 0 | 0 | 0 | 0 | 0.55 |
| CHEMBL560417 | CCCCCc1c(C/C=C(/CCC=C(C)C)\C)c(O)cc(c1OC(=O)C)O | 374.51 | 11 | 4 | 2 | 113.46 | 66.76 | 5.62 | Yes | No | Yes | No | No | 1 | 1 | 1 | 1 | 1 | 0.55 |
| CHEMBL469280 | OC[C@]12CC[C@@]3([C@]([C@H]2[C@@H](C[C@H]1C(C)C)O)(C)CC=C1[C@H]3[C@@H](O)C[C@@H]2[C@]1(C)C[C@H]([C@@H](C2(C)C)O)O)C | 490.72 | 2 | 5 | 5 | 139.79 | 101.15 | 3.65 | No | No | No | No | No | 0 | 3 | 0 | 0 | 0 | 0.55 |
| CHEMBL1574061 | OCC1OC(Oc2cc3occ(c(=O)c3cc2OC)c2ccc(cc2)O)C(C(C1O)O)O | 446.4 | 5 | 10 | 5 | 110.58 | 159.05 | 0.5 | No | No | No | No | Yes | 0 | 0 | 1 | 1 | 1 | 0.55 |
| CHEMBL1916186 | Oc1ccc2c(c1)OC[C@@H](C2=O)Cc1ccc(c(c1)O)O | 286.28 | 2 | 5 | 3 | 76.38 | 86.99 | 1.94 | No | No | No | No | No | 0 | 0 | 0 | 0 | 0 | 0.55 |
| CHEMBL26687 | NCCc1cc(OC)c(c(c1)OC)OC | 211.26 | 5 | 4 | 1 | 58.4 | 53.71 | 1.43 | No | No | No | No | No | 0 | 0 | 0 | 0 | 0 | 0.55 |
| CHEMBL1933679 | OC[C@H]1OC[C@@H]([C@H]([C@@H]1O)OC(=O)c1cc(O)c(c(c1)O)O)O | 316.26 | 4 | 9 | 6 | 70.28 | 156.91 | -0.89 | No | No | No | No | No | 1 | 1 | 1 | 1 | 2 | 0.55 |
| CHEMBL1956008 | OC1=NC[C@]2(C1)C=Cc1c(O2)c2ccccc2oc1=O | 269.25 | 0 | 5 | 1 | 77.88 | 72.03 | 1.92 | No | No | No | No | No | 0 | 0 | 0 | 0 | 0 | 0.55 |
| CHEMBL463315 | C[C@@H]1Oc2c([C@H]1C)c(C)c(cc2O)O | 194.23 | 0 | 3 | 2 | 54.41 | 49.69 | 2.03 | Yes | No | No | No | No | 0 | 0 | 0 | 0 | 1 | 0.55 |
| CHEMBL377937 | Oc1ccc(c(c1)O)[C@@H]1CC(=C[C@@H]([C@H]1C(=O)c1ccc(cc1O)O)c1c(O)ccc(c1O)[C@@H]1CC(=O)c2c(O1)cc(cc2O)O)C | 626.61 | 5 | 11 | 8 | 167.11 | 205.21 | 3.55 | No | No | No | No | No | 3 | 3 | 1 | 1 | 5 | 0.17 |
| CHEMBL109609 | Cc1cc(O)c2c(c1)C(=O)c1c(C2=O)c(ccc1)O[C@@H]1O[C@H](COC(=O)c2cc(O)c(c(c2)O)O)[C@H]([C@@H]([C@H]1O)O)O | 568.48 | 6 | 13 | 7 | 136.59 | 220.51 | 0.74 | No | No | No | No | No | 3 | 2 | 1 | 1 | 3 | 0.17 |
| CHEMBL1451874 | Oc1cc(C)c(c(c1)O)C(=O)Oc1cc(C)c(c(c1)O)C(=O)O | 318.28 | 4 | 7 | 4 | 80.8 | 124.29 | 2.26 | No | No | No | No | No | 0 | 0 | 0 | 0 | 0 | 0.56 |
| CHEMBL490356 | O=C1CCCC[C@@H]1Cc1c(Br)c(O)c(c(c1Br)Br)O | 456.95 | 2 | 3 | 2 | 85.48 | 57.53 | 3.99 | Yes | No | Yes | No | Yes | 0 | 0 | 0 | 0 | 0 | 0.55 |
| CHEMBL226338 | O=C1C2=C(C[C@H](O2)C(O)(C)C)C(=O)c2c1cccc2 | 258.27 | 1 | 4 | 1 | 68.45 | 63.6 | 1.76 | Yes | Yes | No | No | No | 0 | 0 | 0 | 0 | 0 | 0.85 |
| CHEMBL2419883 | CSCC[C@@H]1N=C(O)[C@@H](Cc2ccccc2)N(C)C(=O)[C@H](OC(=O)[C@@H](C)[C@@H](CCC)N=C([C@@H](N=C([C@@H](N(C(=O)[C@@H](N=C1O)Cc1ccccc1)C)CC(C)C)O)C)O)C(C)C | 865.13 | 12 | 12 | 4 | 269.64 | 222.58 | 5.58 | No | No | No | No | No | 2 | 3 | 2 | 1 | 4 | 0.17 |
| CHEMBL475652 | COc1cc(O)cc2c1c1c(O)cccc1CC2 | 242.27 | 1 | 3 | 2 | 70.23 | 49.69 | 2.75 | Yes | No | No | Yes | Yes | 0 | 0 | 0 | 0 | 0 | 0.55 |
| CHEMBL463760 | O=CC(=C(C)C)CCC1=C[C@H](OC1=O)[C@H]([C@]1(OC)OC(=O)C(=C1)C)C(=C)C | 374.43 | 8 | 6 | 0 | 100.83 | 78.9 | 3.31 | No | No | Yes | Yes | No | 0 | 0 | 0 | 0 | 0 | 0.55 |
| CHEMBL473031 | CC(=O)O[C@H]1C(=C)[C@H](OC(=O)C)[C@H]2[C@@H](OC(=O)C)[C@H](C[C@]2(OC(=O)C)C(=O)[C@H](C=CC(C(=O)[C@@H]1OC(=O)C)(C)C)C)C | 592.63 | 10 | 12 | 0 | 147.75 | 165.64 | 2.62 | No | No | No | Yes | No | 2 | 3 | 1 | 1 | 2 | 0.17 |
| CHEMBL1972346 | Oc1cc(C=Cc2ccc(c(c2)O)O)cc(c1)O | 244.24 | 2 | 4 | 4 | 69.9 | 80.92 | 2.14 | Yes | No | Yes | No | Yes | 0 | 0 | 0 | 0 | 0 | 0.55 |
| CHEMBL1797816 | CCCC[C@H](c1nc(O)c2c(n1)cccc2)CC | 244.33 | 5 | 3 | 1 | 75.37 | 46.01 | 3.64 | Yes | Yes | Yes | Yes | No | 0 | 0 | 0 | 0 | 0 | 0.55 |
| CHEMBL176570 | COc1cc(C(=O)O)c2c(c1C)OC(=O)c1c(O2)c(C=O)c(cc1C)O | 358.3 | 3 | 8 | 2 | 88.71 | 119.36 | 2.36 | No | No | Yes | No | No | 0 | 0 | 0 | 0 | 0 | 0.56 |
| CHEMBL2286985 | CC([C@@]12CC[C@@](OO1)(C=C2)C)C | 168.23 | 1 | 2 | 0 | 47.73 | 18.46 | 2.34 | No | No | No | No | No | 0 | 0 | 0 | 0 | 1 | 0.55 |
| CHEMBL499143 | O=C1C(=C[C@H]2[C@](C1=C)(C)CC[C@@H]1[C@]2(C)CC[C@](C1)(C)C(=O)O)O | 318.41 | 1 | 4 | 2 | 88.92 | 74.6 | 3.17 | No | No | Yes | No | No | 0 | 0 | 0 | 0 | 0 | 0.85 |
| CHEMBL480286 | CNC1=CC(=O)c2c(C1=O)cncc2 | 188.18 | 1 | 3 | 1 | 49.65 | 59.06 | 0.61 | Yes | No | No | No | No | 0 | 0 | 0 | 0 | 1 | 0.55 |
| CHEMBL500186 | CCCCCCCCCC(=CC(=NCCc1c[nH]c2c1cccc2)O)C | 354.53 | 12 | 2 | 2 | 115.35 | 48.38 | 6.03 | No | Yes | No | Yes | Yes | 1 | 1 | 1 | 1 | 1 | 0.55 |
| CHEMBL520981 | OC[C@H]1O[C@@H](Oc2cc(cc(c2O)O)C(=O)OC)[C@@H]([C@H]([C@@H]1O)O)O | 346.29 | 5 | 10 | 6 | 75.91 | 166.14 | -1.12 | No | No | No | No | No | 1 | 1 | 1 | 1 | 2 | 0.55 |
| CHEMBL254807 | COc1cc2cc(O)c(c(c2c2c1cccc2O)OC)O | 286.28 | 2 | 5 | 3 | 80.51 | 79.15 | 2.54 | Yes | No | Yes | Yes | Yes | 0 | 0 | 0 | 0 | 0 | 0.55 |
| CHEMBL459603 | C=CCC=CCC=CCCCCCCCc1cc(O)cc(c1)O | 314.46 | 12 | 2 | 2 | 101.33 | 40.46 | 5.67 | Yes | Yes | Yes | No | Yes | 1 | 1 | 1 | 1 | 1 | 0.55 |
| CHEMBL463794 | OC[C@]1(C)[C@@H](O)CC[C@]2([C@H]1CC[C@@]1([C@@H]2CC(=O)[C@]23[C@@]1(C)CC[C@@]1([C@H]3[C@@H](C)[C@@H](CC1)C)CO2)C)C | 472.7 | 1 | 4 | 2 | 135.99 | 66.76 | 4.98 | No | No | No | No | No | 1 | 2 | 0 | 0 | 1 | 0.55 |
| CHEMBL443832 | CCc1ccc(cc1)c1ccc(cc1)C(=O)O | 226.27 | 3 | 2 | 1 | 68.61 | 37.3 | 3.59 | No | No | No | No | No | 0 | 0 | 0 | 0 | 0 | 0.85 |
| CHEMBL3747112 | COc1cc(CCc2ccccc2O)cc(c1OC)OC | 288.34 | 6 | 4 | 1 | 82.2 | 47.92 | 3.28 | Yes | Yes | No | Yes | No | 0 | 0 | 0 | 0 | 0 | 0.55 |
| CHEMBL450145 | COC(=O)[C@]12OC[C@]34[C@H]2[C@@H](OC(=O)/C=C(/C(OC(=O)C)(C)C)\C)C(=O)O[C@@H]4C[C@@H]2[C@]([C@H]3[C@H]([C@@H]1O)O)(C)CC(=O)C(=C2C)O | 606.61 | 8 | 13 | 3 | 144.68 | 192.19 | 0.63 | No | No | No | No | No | 2 | 3 | 1 | 1 | 3 | 0.17 |
| CHEMBL451046 | COc1cc(/C=C/C(=O)O[C@H]2CC[C@]3([C@H](C2(C)C)CC[C@@]2([C@@H]3CC[C@H]3[C@@]2(C)CC[C@@]2([C@@H]3[C@@H](CC2)C(=C)C)C(=O)O)C)C)ccc1O | 632.87 | 7 | 6 | 2 | 184.78 | 93.06 | 7.63 | No | No | No | No | Yes | 2 | 4 | 0 | 1 | 2 | 0.56 |
| CHEMBL453761 | C/C=C/C#CC#Cc1cccs1 | 172.25 | 0 | 0 | 0 | 53.98 | 28.24 | 3.36 | Yes | No | Yes | No | No | 0 | 0 | 0 | 0 | 2 | 0.55 |
| CHEMBL511842 | O=C1OC(=Cc2cc(Br)c(c(c2)Br)O)C(=C1)c1cc(Br)c(c(c1)Br)O | 595.86 | 2 | 4 | 2 | 109.78 | 66.76 | 5.1 | No | No | Yes | No | No | 2 | 2 | 0 | 1 | 1 | 0.17 |
| CHEMBL456751 | OC[C@H]1O[C@@H](Oc2cc(O)cc(c2C(=O)C(C)C)O)[C@@H]([C@H]([C@@H]1O)O)O | 358.34 | 5 | 9 | 6 | 84.44 | 156.91 | -0.3 | No | No | No | No | No | 1 | 1 | 1 | 1 | 2 | 0.55 |
| CHEMBL516549 | C=C1CC[C@@H]2O[C@]2(CC[C@H]2[C@H]1COC(=O)C2=CC=CC(O)(C)C)C | 332.43 | 2 | 4 | 1 | 94.1 | 59.06 | 3.12 | No | No | No | No | No | 0 | 0 | 0 | 0 | 0 | 0.55 |
| CHEMBL2338628 | OCC1=C[C@@]2(O[C@H](/C=C(/CCC(=O)C(C)C)\C)CC(=C2)C)O[C@H]2[C@@H]1CC(=O)C(=C2)C | 414.53 | 6 | 5 | 1 | 117.82 | 72.83 | 3.39 | No | No | No | No | Yes | 0 | 0 | 0 | 0 | 0 | 0.55 |
| CHEMBL44658 | CCC(O)(C)C | 88.15 | 1 | 1 | 1 | 27.35 | 20.23 | 1.11 | No | No | No | No | No | 0 | 3 | 0 | 0 | 2 | 0.55 |
| CHEMBL451903 | COc1cc(ccc1O)C[C@H]([C@@H](Cc1ccc(c(c1)OC)O)COC(=O)C=Cc1ccc(c(c1)OC)O)COC(=O)C=Cc1ccc(c(c1)OC)O | 714.75 | 19 | 12 | 4 | 195.01 | 170.44 | 5.5 | No | Yes | Yes | No | No | 2 | 4 | 2 | 1 | 5 | 0.17 |
| CHEMBL253558 | OC[C@@]1(C)CCC[C@@]2([C@@H]1CC[C@]13[C@H]2CC[C@H](C1)C(=C)C3=O)C | 302.45 | 1 | 2 | 1 | 89.91 | 37.3 | 3.99 | No | No | Yes | No | No | 0 | 0 | 0 | 0 | 0 | 0.55 |
| CHEMBL432530 | O[C@H]1C[C@@]2(C)[C@@H]([C@]([C@H]1O)(C)C(=O)O)CC[C@@]1([C@@H]2CC=C2[C@@]1(C)CC[C@@]1([C@H]2CC(C)(C)CC1)C(=O)O)C | 502.68 | 2 | 6 | 4 | 139.59 | 115.06 | 4.26 | No | No | No | No | No | 1 | 3 | 0 | 0 | 1 | 0.56 |
| CHEMBL515179 | O=C1CN[C@H]2N1c1ccccc1[C@]12C[C@@H]2C(=N[C@@]1(C)c1n2c(=O)c2c(n1)cccc2)O | 413.43 | 0 | 6 | 2 | 124.7 | 99.82 | 1.57 | No | Yes | No | No | No | 0 | 0 | 0 | 0 | 0 | 0.55 |
| CHEMBL335955 | C[C@H]1Oc2c3c(O[C@H]4[C@@H]3C4(C)C)c3c(c2[C@H]([C@H]1C)O)oc(=O)cc3c1ccccc1 | 404.46 | 1 | 5 | 1 | 114.24 | 68.9 | 4.12 | No | Yes | No | Yes | No | 0 | 0 | 0 | 0 | 0 | 0.55 |
| CHEMBL463849 | C=CCc1cc(O[C@@H]2O[C@H](COC(=O)/C=C/c3cc(OC)c(c(c3)OC)O)[C@H]([C@@H]([C@H]2O)O)O)c(c(c1)OC)O | 548.54 | 12 | 12 | 5 | 137.56 | 173.6 | 1.59 | No | No | No | No | No | 2 | 3 | 2 | 1 | 2 | 0.17 |
| CHEMBL451866 | COc1ccc2c(c1)OC(C=C2)(C)C | 190.24 | 1 | 2 | 0 | 57.05 | 18.46 | 2.77 | Yes | No | No | Yes | No | 0 | 0 | 0 | 0 | 1 | 0.55 |
| CHEMBL432995 | OC(=N[C@H]1COC(=O)[C@H](COC(=O)[C@H](COC1=O)N=C(c1cccc(c1O)O)O)N=C(c1cccc(c1O)O)O)c1cccc(c1O)O | 669.55 | 6 | 18 | 9 | 162.91 | 298.05 | 1.28 | No | No | No | No | No | 3 | 3 | 1 | 1 | 4 | 0.17 |
| CHEMBL1814589 | CCCCCN=C=S | 129.22 | 4 | 1 | 0 | 40.12 | 44.45 | 2.86 | No | No | No | No | No | 0 | 2 | 0 | 0 | 1 | 0.55 |
| CHEMBL3289674 | COc1cc2OCC=C(c2cc1O)c1ccccc1 | 254.28 | 2 | 3 | 1 | 73.91 | 38.69 | 2.99 | Yes | Yes | No | Yes | Yes | 0 | 0 | 0 | 0 | 0 | 0.55 |
| CHEMBL371946 | OC(=O)[C@H](C[C@H](C(=O)O)O)N | 163.13 | 4 | 6 | 4 | 33.56 | 120.85 | -2.31 | No | No | No | No | No | 0 | 2 | 0 | 0 | 2 | 0.56 |
| CHEMBL517085 | O=C1O[C@@H]2C[C@]([C@@H]3[C@@]42[C@@H](C1)[C@@H](C)[C@H]([C@@]3(OC4)O)O)(C)[C@@H]1OC(=O)C=C1C | 364.39 | 1 | 7 | 2 | 87.9 | 102.29 | 1.06 | No | No | No | No | No | 0 | 0 | 0 | 0 | 0 | 0.55 |
| CHEMBL501233 | CC[C@@H]([C@H](N(C(=O)[C@H](C(C)C)N=C([C@H]([C@H](CC)C)N(C)C)O)C)[C@@H](CC(=O)N1CCC[C@H]1[C@@H]([C@H](C(=N[C@H](c1nccs1)Cc1ccccc1)O)C)OC)OC)C | 799.12 | 24 | 10 | 2 | 233.77 | 168.63 | 6.09 | No | No | No | No | Yes | 2 | 4 | 2 | 2 | 4 | 0.17 |
| CHEMBL223942 | CC([C@@H]1CCc2coc(c2)C[C@]2([C@H](CC[C@](/C=C/1)(C)O)O2)C)C | 318.45 | 1 | 3 | 1 | 93.56 | 45.9 | 3.81 | No | No | No | Yes | No | 0 | 0 | 0 | 0 | 0 | 0.55 |
| CHEMBL2334889 | COc1cc2CCN3[C@@H](c2cc1OC)Cc1c(C3)c(OC)c(cc1)OC | 355.43 | 4 | 5 | 0 | 103.99 | 40.16 | 3.09 | No | No | No | Yes | Yes | 0 | 0 | 0 | 0 | 0 | 0.55 |
| CHEMBL2386503 | O=c1cc(C)c2c(cc1C(O)(C)C)[C@@](CC2)(C)O | 248.32 | 1 | 3 | 2 | 71.78 | 57.53 | 1.93 | No | No | No | No | No | 0 | 0 | 0 | 0 | 0 | 0.55 |
| CHEMBL3181980 | COCc1ccc(cc1)COC | 166.22 | 4 | 2 | 0 | 48.16 | 18.46 | 1.87 | Yes | No | No | No | No | 0 | 0 | 0 | 0 | 1 | 0.55 |
| CHEMBL560374 | Oc1cc(/C=C/c2ccccc2)c2c(c1)O[C@@H](CC2=O)c1ccccc1 | 342.39 | 3 | 3 | 1 | 102.89 | 46.53 | 4.23 | Yes | Yes | Yes | No | No | 0 | 0 | 0 | 0 | 0 | 0.55 |
| CHEMBL589343 | COC(=O)/C=C/1\CC[C@@]2(O1)[C@H](C)CC[C@@H]1[C@]2(C)CC[C@@H](C1(C)C)OC(=O)C | 378.5 | 4 | 5 | 0 | 104.23 | 61.83 | 4.03 | No | No | No | No | No | 0 | 0 | 0 | 0 | 0 | 0.85 |
| CHEMBL469424 | COc1cc(O)cc(c1Oc1cc(C)cc(c1C(=O)O)O)C(=O)OC | 348.3 | 6 | 8 | 3 | 86.7 | 122.52 | 2.18 | No | No | No | No | No | 0 | 0 | 0 | 0 | 0 | 0.56 |
| CHEMBL486961 | C=C1CCC[C@]2([C@H]1CC1=C(C)C(=O)O[C@]1(C2)O)C | 248.32 | 0 | 3 | 1 | 69.15 | 46.53 | 2.65 | No | No | Yes | No | No | 0 | 0 | 0 | 0 | 0 | 0.55 |
| CHEMBL452242 | O[C@H]1CC[C@@H]2C(=CC[C@H]3[C@@]2(C)CC[C@@]2([C@]3(C)CC[C@@]3([C@H]2CC(C)(C)CC3)C)C)C1(C)C | 426.72 | 0 | 1 | 1 | 134.88 | 20.23 | 7.19 | No | No | No | No | No | 1 | 3 | 0 | 1 | 2 | 0.55 |
| CHEMBL1807146 | COc1cc(O)c(c(c1)c1oc2c(c1)c(O)cc(c2)OC)C | 300.31 | 3 | 5 | 2 | 83.65 | 72.06 | 3.02 | Yes | Yes | No | Yes | Yes | 0 | 0 | 0 | 0 | 0 | 0.55 |
| CHEMBL487081 | CCCCC/C=C/C=C/C(=O)O[C@H]1C(=C[C@]23[C@]1(O)[C@H](O)C(=C[C@H](C3=O)[C@H]1[C@@H](C[C@H]2C)C1(C)C)CO)C | 498.65 | 9 | 6 | 3 | 140.46 | 104.06 | 3.02 | Yes | Yes | No | Yes | Yes | 0 | 0 | 0 | 0 | 0 | 0.55 |
| CHEMBL1795551 | CCCCCCC=CCCCCCCCCCCCc1cc(O)cc(c1)O | 374.6 | 17 | 2 | 2 | 121.51 | 40.46 | 7.51 | Yes | Yes | No | No | No | 1 | 1 | 1 | 1 | 2 | 0.55 |
| CHEMBL478941 | COC(=O)C[C@@H](c1ccc(c(c1)O)O)OC(=O)/C=C/c1ccc(c(c1)O)O | 374.34 | 8 | 8 | 4 | 95.72 | 133.52 | 1.93 | No | No | No | No | No | 0 | 0 | 0 | 1 | 0 | 0.55 |
| CHEMBL497243 | OC[C@@]1(C)CCC[C@]2([C@H]1CCC(=C)[C@@H]2CCC1=CCOC1=O)C | 318.45 | 4 | 3 | 1 | 92.89 | 46.53 | 3.96 | No | Yes | Yes | No | No | 0 | 0 | 0 | 0 | 0 | 0.55 |
| CHEMBL498119 | O=C/C=C/C(=C/C=C/C(=C\1/C(=O)C[C@H]2[C@]1(C)CC[C@@H]1[C@]2(C)CC[C@H]([C@]1(C)C(=O)OC)OC(=O)C)/C)/C | 496.64 | 8 | 6 | 0 | 140.28 | 86.74 | 5.11 | No | No | Yes | No | No | 0 | 3 | 0 | 0 | 1 | 0.55 |
| CHEMBL512552 | C=CC1=C(C)[C@H]2[C@H](CC1)[C@@]1(C)CCC[C@@]([C@@H]1[C@@H](C2)O)(C)CO | 304.47 | 2 | 2 | 2 | 92.77 | 40.46 | 3.83 | No | No | No | No | No | 0 | 0 | 0 | 0 | 0 | 0.55 |
| CHEMBL480072 | OC[C@H]1O[C@@H](OC[C@@]2(O)[C@H]3C[C@H]3[C@]3([C@H]2CC2=C(C)C(=O)OC2=C3)C)[C@@H]([C@H]([C@@H]1O)O)O | 424.44 | 4 | 9 | 5 | 100.59 | 145.91 | -0.23 | No | No | No | No | No | 0 | 1 | 1 | 1 | 0 | 0.55 |
| CHEMBL182992 | COc1cc2oc(cc(=O)c2c(c1OC)OC)c1ccccc1 | 312.32 | 4 | 5 | 0 | 87.4 | 57.9 | 3.02 | Yes | Yes | Yes | Yes | Yes | 0 | 0 | 0 | 0 | 0 | 0.55 |
| CHEMBL1668468 | CC(=O)O[C@H]1CC[C@@]2([C@@H]([C@]1(C)CO[C@@H]1CCC(=N1)O)CC[C@]13[C@H]2[C@@H](O)C[C@H](C1)C(=C)C3=O)C | 459.58 | 5 | 7 | 2 | 127.59 | 105.42 | 3.1 | No | No | No | No | Yes | 0 | 0 | 0 | 0 | 0 | 0.55 |
| CHEMBL445203 | COC(=O)Cc1ccc(cc1)OC | 180.2 | 4 | 3 | 0 | 48.8 | 35.53 | 1.86 | Yes | No | No | No | No | 0 | 0 | 0 | 0 | 1 | 0.55 |
| CHEMBL508537 | CC(=O)CC[C@H]1C(=C)C(=O)O[C@@H]1C1=C(C)[C@H](CC1=O)O | 278.3 | 4 | 5 | 1 | 71.89 | 80.67 | 1.15 | No | No | No | No | No | 0 | 0 | 0 | 0 | 0 | 0.55 |
| CHEMBL2152481 | COCC1=CCc2c(c3c1cc(OC)c(c3)O)ccc(c2)OC | 312.36 | 4 | 4 | 1 | 90.53 | 47.92 | 3.26 | Yes | Yes | No | Yes | Yes | 0 | 0 | 0 | 0 | 0 | 0.55 |
| CHEMBL1950971 | O[C@H]1C[C@@H](O)c2c3C1=C1c4cccc(c4C(=O)C[C@@H]1c3ccc2O)O | 336.34 | 0 | 5 | 4 | 91.58 | 97.99 | 1.64 | Yes | No | No | Yes | No | 0 | 0 | 0 | 0 | 0 | 0.55 |
| CHEMBL1950969 | CCO[C@@H]1C[C@H](O)C2=C3[C@@H](c4c2c1c(O)cc4)CC(=O)c1c3cccc1O | 364.39 | 2 | 5 | 3 | 101.12 | 86.99 | 2.65 | No | No | No | Yes | No | 0 | 0 | 0 | 0 | 0 | 0.55 |
| CHEMBL2164941 | COc1cc2c(cc1OC)N1[C@@H]3[C@@]42CCN2[C@@]4(O)C[C@@H]4[C@H]3[C@H](CC1=O)OCC=C4C2 | 410.46 | 2 | 6 | 1 | 115.24 | 71.47 | 1.68 | No | No | No | Yes | No | 0 | 0 | 0 | 0 | 0 | 0.55 |
| CHEMBL1092510 | CCCCCC/C=C/C=C/C#C/C=C/C=C/COC(=O)C | 286.41 | 10 | 2 | 0 | 91 | 26.3 | 5.15 | Yes | No | Yes | No | No | 1 | 0 | 0 | 0 | 1 | 0.55 |
| CHEMBL465063 | COC1=CC(=O)C(=CC1=O)OC | 168.15 | 2 | 4 | 0 | 40.08 | 52.6 | 0.24 | No | No | No | No | No | 0 | 0 | 0 | 0 | 1 | 0.85 |
| CHEMBL925 | OC(=O)[C@H](Cc1ccc(cc1)O)N | 181.19 | 3 | 4 | 3 | 47.52 | 83.55 | -0.48 | No | No | No | No | No | 0 | 0 | 0 | 0 | 2 | 0.55 |
| CHEMBL1087439 | COC(=O)C(CC(=O)C[C@H]([C@H]1CC(=O)[C@@]2([C@]1(C)CC(=O)C1=C2[C@@H](O)C[C@@H]2[C@]1(C)CCC(=O)C2(C)C)C)C)C | 528.68 | 7 | 7 | 1 | 144.41 | 114.81 | 3.74 | No | No | No | No | No | 1 | 3 | 0 | 0 | 0 | 0.55 |
| CHEMBL463263 | O=C1CC[C@]2([C@@H](C1)OCC2)O | 156.18 | 0 | 3 | 1 | 38.83 | 46.53 | 0.43 | No | No | No | No | No | 0 | 2 | 0 | 0 | 1 | 0.55 |
| CHEMBL454965 | COC(=O)CCC(=Nc1ccccc1C(=O)OC[C@]12CC[C@@H]([C@@]34[C@@H]2[C@H](OC)[C@@]([C@H]3N(C1)CC)(O)[C@@]1([C@@H]2[C@H]4C[C@@H]([C@@H]2OC)[C@H](C1)OC)O)OC)O | 700.82 | 14 | 13 | 3 | 184.73 | 165.81 | 0.43 | No | No | No | No | No | 0 | 2 | 0 | 0 | 1 | 0.55 |
| CHEMBL479018 | CC(C[C@H]1C(=O)N(C(=O)[C@@H]1c1ccc(cc1)OCC=C(C)C)O)C | 331.41 | 6 | 4 | 1 | 96.13 | 66.84 | 3.16 | No | Yes | Yes | No | Yes | 0 | 0 | 0 | 0 | 0 | 0.55 |
| CHEMBL1773909 | O[C@H]1C=CC(=O)C(C1)(CC=C(C)C)CC=C(C)C | 248.36 | 4 | 2 | 1 | 76.59 | 37.3 | 3.27 | No | No | Yes | No | No | 0 | 0 | 0 | 0 | 0 | 0.55 |
| CHEMBL490513 | OC[C@H]1O[C@@H](Oc2cc(OC)cc(c2C(=O)CCc2ccc(cc2)O)O)[C@@H]([C@H]([C@@H]1O)O)O | 450.44 | 8 | 10 | 6 | 110.61 | 166.14 | 0.6 | No | No | No | No | No | 1 | 0 | 1 | 1 | 2 | 0.55 |
| CHEMBL2270648 | O=C1O[C@@H]2[C@@]3([C@H]1[C@]1(CO3)C(=O)C=C3[C@]([C@H]1[C@H]2O)(C)C(=O)C=C3C)C | 330.33 | 0 | 6 | 1 | 80.57 | 89.9 | 0.68 | No | No | No | No | No | 0 | 0 | 0 | 0 | 0 | 0.55 |
| CHEMBL103410 | COc1cc(/C=C/C(=O)CC(=O)/C=C/c2ccc(c(c2)O)O)ccc1O | 354.35 | 7 | 6 | 3 | 98.33 | 104.06 | 2.62 | No | No | Yes | No | Yes | 0 | 0 | 0 | 0 | 0 | 0.55 |
| CHEMBL1080178 | OC[C@H]1O[C@@H](O[C@@H]2OC=C([C@@H]3[C@H]2[C@@H](C)CC3=O)C(=O)OC)[C@@H]([C@H]([C@@H]1O)O)O | 388.37 | 5 | 10 | 4 | 86.4 | 151.98 | -0.98 | No | No | No | No | No | 0 | 1 | 1 | 1 | 1 | 0.11 |
| CHEMBL503787 | OC[C@H]1O[C@@H](O[C@H]2CC[C@]3([C@H](C2(C)C)CCc2c3c(O)c(c(c2)[C@@H](CO)C)O[C@@H]2O[C@H](CO)[C@H]([C@@H]([C@H]2O)O)O)C)[C@@H]([C@H]([C@@H]1O)O)O | 658.73 | 8 | 14 | 10 | 160.49 | 239.22 | -0.76 | No | No | No | No | No | 3 | 4 | 1 | 1 | 4 | 0.17 |
| CHEMBL449171 | OC[C@H]1O[C@@H](OC/C=C(/CC=CC(O)(C)C)\C)[C@@H]([C@H]([C@@H]1OC(=O)/C=C/c1ccc(cc1)O)O[C@@H]1O[C@@H](C)[C@@H]([C@H]([C@H]1O)O)O)O | 624.67 | 13 | 13 | 7 | 156.57 | 204.83 | 0.67 | No | No | No | No | No | 3 | 3 | 2 | 1 | 4 | 0.17 |
| CHEMBL448815 | OC(=O)C(=O)C1=C(O)/C(=C/c2ccc(c(c2)Cl)O)/N=C1O | 309.66 | 3 | 7 | 4 | 77.14 | 127.42 | 1.1 | No | No | No | No | No | 0 | 0 | 0 | 0 | 0 | 0.56 |
| CHEMBL558750 | CCCCCCCCCC[C@@H]1c2c(OO[C@]31C(=O)O[C@@H](C3=O)C)cc(c1c2oc(cc1=O)c1ccc(cc1)O[C@@H]1O[C@H](CO)[C@H]([C@@H]([C@H]1O)O)O)O | 712.74 | 13 | 14 | 5 | 181.22 | 211.65 | 3.55 | No | No | No | No | Yes | 2 | 3 | 2 | 1 | 4 | 0.17 |
| CHEMBL253433 | COc1cc(ccc1O)[C@H]([C@@H]([C@@H](Cc1ccc2c(c1)OCO2)C)C)O | 344.4 | 6 | 5 | 2 | 95.67 | 68.15 | 3.47 | No | Yes | No | Yes | No | 0 | 0 | 0 | 0 | 0 | 0.55 |
| CHEMBL516799 | CSC1=CC(=O)C(=C[C@@]21C=CNc1c2c2nccc3c2c(c1=O)n(c3)C)Br | 440.31 | 1 | 3 | 1 | 116.71 | 89.29 | 2.82 | Yes | Yes | Yes | No | Yes | 0 | 0 | 0 | 0 | 0 | 0.55 |
| CHEMBL2063160 | OC[C@H]1O[C@@H](OC(=O)[C@]23CC[C@@]4([C@@H]([C@H]3[C@@H](C[C@@H]2O)C(=C)C)CC[C@H]2[C@@]4(C)CC[C@@H]3[C@]2(C)[C@@H](CC(=O)O)OC3(C)C)C)[C@@H]([C@H]([C@@H]1O)O)O | 664.82 | 7 | 11 | 6 | 172.04 | 183.21 | 2.91 | No | No | No | No | No | 3 | 3 | 1 | 1 | 4 | 0.11 |
| CHEMBL376042 | Brc1ccc2c(c1)[nH]cc2[C@@H]1CN=C([C@@H](N1)c1c[nH]c2c1cccc2)O | 409.28 | 2 | 3 | 4 | 114.91 | 76.2 | 3.2 | No | Yes | No | Yes | Yes | 0 | 0 | 0 | 0 | 0 | 0.55 |
| CHEMBL353955 | OC[C@H]1O[C@H](C[C@@H]1O)n1ccc(nc1=O)O | 228.2 | 2 | 6 | 3 | 52.3 | 104.81 | -0.94 | No | No | No | No | No | 0 | 1 | 0 | 0 | 0 | 0.55 |
| CHEMBL491544 | COC1C(=O)CCC2C1CN1CCc3c(C1C2)cc1c(c3)OCO1.Cl | 365.85 | 1 | 5 | 0 | 99.23 | 48 | 1.95 | No | No | No | Yes | Yes | 0 | 0 | 0 | 0 | 0 | 0.55 |
| CHEMBL496894 | C/C=C\1/C[C@@H](C)[C@](O)(CO)C(=O)OCC2=CCN3[C@H]2[C@H](OC1=O)CC3 | 351.39 | 1 | 7 | 2 | 93.09 | 96.3 | 0.88 | No | No | No | No | No | 0 | 0 | 0 | 0 | 0 | 0.55 |
| CHEMBL540151 | O[C@@H]1CC[C@@](CC1)(O)CC(=O)OC[C@H]1O[C@@H](OCCc2ccc(c(c2)O)O)[C@@H]([C@H]([C@@H]1OC(=O)/C=C/c1ccc(c(c1)O)O)O[C@@H]1O[C@@H](C)[C@@H]([C@H]([C@H]1O)O)O)O | 780.77 | 15 | 18 | 10 | 187.25 | 291.82 | 0.11 | No | No | No | No | No | 3 | 4 | 2 | 1 | 4 | 0.17 |
| CHEMBL3184983 | O=CC(CCC=C(C)C)C | 140.22 | 4 | 1 | 0 | 45.1 | 17.07 | 2.42 | No | No | No | No | No | 0 | 1 | 0 | 0 | 2 | 0.55 |
| CHEMBL2337110 | COC1=Cc2ccnc3c2c(C1=O)ncc3N | 227.22 | 1 | 4 | 1 | 63.2 | 78.1 | 0.83 | Yes | No | No | No | Yes | 0 | 0 | 0 | 0 | 0 | 0.55 |
| CHEMBL1080115 | CCCCCCCCC=CCCCCCCCc1c(C)c(O)cc2c1[nH]c1c2cccc1 | 433.67 | 15 | 1 | 2 | 144.2 | 36.02 | 8.58 | No | No | No | No | No | 1 | 3 | 1 | 1 | 1 | 0.55 |
| CHEMBL2227789 | COC[C@@]12CN(CC)C3[C@]4([C@@H]2[C@@H](OC)[C@@H]3[C@@]2([C@@H]3[C@H]4C[C@@]([C@@H]3OC(=O)c3ccc(cc3)OC)([C@H](C2)OC)O)OC(=O)C)[C@H](C[C@H]1O)OC | 659.76 | 12 | 12 | 2 | 170.83 | 142.45 | 1.77 | No | No | No | No | No | 2 | 3 | 2 | 1 | 2 | 0.17 |
| CHEMBL256146 | CCCCCCC/C=C/C(=O)CCCCCCCC(=O)O | 296.44 | 15 | 3 | 1 | 90.14 | 54.37 | 4.78 | Yes | No | Yes | No | No | 0 | 0 | 1 | 0 | 1 | 0.85 |
| CHEMBL2139695 | CCCCCCC(CCCCCCCCCCC(=O)OC)O | 314.5 | 17 | 3 | 1 | 95.89 | 46.53 | 5.43 | Yes | No | No | No | No | 0 | 0 | 1 | 0 | 2 | 0.55 |
| CHEMBL363942 | CC(=O)OC[C@]12[C@@H](OC(=O)c3ccccc3)[C@@H](OC(=O)C)C[C@]([C@@]32OC([C@H](C(=O)[C@H]1OC(=O)c1ccccc1)[C@H]3O)(C)C)(C)O | 624.63 | 11 | 12 | 2 | 154.74 | 171.96 | 2.43 | No | No | No | No | Yes | 2 | 3 | 2 | 1 | 3 | 0.17 |
| CHEMBL403750 | CCCCCCCCCCCCC[C@@H]1C[C@@H](O)C[C@]2(O1)CCC1(O2)C=CC(=O)C=C1 | 418.61 | 12 | 4 | 1 | 123.41 | 55.76 | 5.58 | No | No | No | Yes | No | 0 | 2 | 1 | 1 | 1 | 0.55 |
| CHEMBL251978 | Oc1ccc(cc1)[C@@H]1CCc2c(O1)c(C)c(cc2)O | 256.3 | 1 | 3 | 2 | 74.09 | 49.69 | 2.96 | Yes | No | No | Yes | No | 0 | 0 | 0 | 0 | 0 | 0.55 |
| CHEMBL3578258 | COc1cc(C[C@H]2COC(=O)[C@@H]2Cc2ccc(c(c2)OC)O)cc(c1OC)OC | 402.44 | 8 | 7 | 1 | 107.09 | 83.45 | 3.04 | No | No | Yes | Yes | Yes | 0 | 0 | 0 | 0 | 0 | 0.55 |
| CHEMBL194378 | CN(CCc1c[nH]c2c1c(ccc2)OP(=O)(O)O)C | 284.25 | 5 | 5 | 3 | 73.25 | 95.6 | 0.47 | No | No | No | No | No | 0 | 0 | 0 | 0 | 0 | 0.56 |
| CHEMBL2159606 | OC[C@H]1O[C@@H](Oc2ccc(cc2O)CCCC[C@@H](CCc2ccc(c(c2)O)O[C@@H]2O[C@H](CO)[C@H]([C@@H]([C@H]2O)O)O)O)[C@@H]([C@H]([C@@H]1O)O)O | 656.67 | 14 | 15 | 11 | 158.24 | 259.45 | -0.44 | No | No | No | No | No | 3 | 4 | 2 | 1 | 4 | 0.17 |
| CHEMBL229454 | CC(=CCc1cc(cc(c1O)O)[C@@H]1CC(=O)c2c(O1)cc(cc2O)O)C | 356.37 | 3 | 6 | 4 | 97.31 | 107.22 | 2.9 | Yes | No | Yes | Yes | Yes | 0 | 0 | 0 | 0 | 0 | 0.55 |
| CHEMBL507293 | COc1ccc(c2c1CCC(O2)(C)C)C(=O)/C=C/c1ccc(cc1)O | 338.4 | 4 | 4 | 1 | 98.57 | 55.76 | 3.85 | Yes | Yes | Yes | Yes | Yes | 0 | 0 | 0 | 0 | 0 | 0.55 |
| CHEMBL452646 | CC1=CCCC(=CC[C@@H](CC1)C(N)(C)C)C | 221.38 | 1 | 1 | 1 | 73.9 | 26.02 | 3.38 | No | No | No | No | No | 0 | 0 | 0 | 0 | 1 | 0.55 |
| CHEMBL1366408 | OC[C@H]1O[C@@H]([C@@H]([C@H]([C@@H]1O)O)O)c1c(O)c2c(c(c1O)O)C(=O)c1c(C2=O)c(C)c(c(c1)O)C(=O)O | 492.39 | 3 | 13 | 9 | 112.39 | 242.51 | -0.85 | No | No | No | No | No | 2 | 2 | 1 | 1 | 3 | 0.11 |
| CHEMBL3289101 | OC(=O)CC[C@@]1(C)[C@@H](CC[C@@]2([C@@H]1CC=C1[C@@]2(C)CC[C@@]2([C@H]1[C@@H](C)[C@H](C)CC2)C)C)C(=C)C | 440.7 | 4 | 2 | 1 | 137.65 | 37.3 | 7.01 | No | No | Yes | No | No | 1 | 3 | 0 | 1 | 1 | 0.85 |
| CHEMBL171804 | O[C@H]1CC[C@]2(C(=CC[C@@H]3[C@@H]2CC[C@]2([C@H]3CC[C@@H]2[C@@H](CC[C@@H](C(C)C)O)C)C)C1)C | 402.65 | 5 | 2 | 2 | 124.78 | 40.46 | 5.78 | No | No | No | No | No | 1 | 2 | 0 | 1 | 1 | 0.55 |
| CHEMBL525937 | CC(C[C@@H]1N=C(O)[C@@H]2CCCN2C(=O)[C@@H](N=C(O)[C@@H](N=C(O)[C@H]2N(C(=O)[C@@H](N=C([C@H]3N(C(=O)[C@@H](N=C1O)[C@H](O)C)CCC3)O)CC(C)C)CCC2)Cc1c[nH]c2c1cccc2)C(C)C)C | 904.11 | 8 | 14 | 7 | 282.58 | 259.9 | 3.47 | No | No | No | No | No | 3 | 3 | 1 | 1 | 5 | 0.17 |
| CHEMBL493737 | CO[C@@H]1[C@@H](O)[C@@H](O)[C@@H]([C@@H]([C@H]1O)O)O | 194.18 | 1 | 6 | 5 | 40.54 | 110.38 | -2.22 | No | No | No | No | No | 0 | 1 | 0 | 0 | 2 | 0.55 |
| CHEMBL501246 | CC(=O)Oc1ccc(cc1)C | 150.17 | 2 | 2 | 0 | 42.91 | 26.3 | 2.11 | Yes | No | No | No | No | 0 | 1 | 0 | 0 | 1 | 0.55 |
| CHEMBL556684 | COc1cc(O)c2c(c1)C(=O)c1c(C2=O)c(O)c(c(c1)C)O | 300.26 | 1 | 6 | 3 | 77.28 | 104.06 | 1.92 | Yes | No | Yes | No | Yes | 0 | 0 | 0 | 0 | 0 | 0.55 |
| CHEMBL479892 | OC[C@H]1O[C@H]2Oc3c4[C@@H](O[C@@H]2[C@H]([C@@H]1O)OC(=O)C)C[C@H](Oc4c(c(c3C)O)C)c1ccc(cc1)OC | 502.51 | 5 | 10 | 3 | 125.22 | 133.14 | 2.14 | No | No | No | Yes | No | 1 | 1 | 0 | 1 | 0 | 0.55 |
| CHEMBL2419350 | COc1c(O)cc2c(c1C(=O)O)c(=O)c1c(o2)cc(nc1)C | 301.25 | 2 | 7 | 2 | 78.23 | 109.86 | 1.52 | Yes | No | No | No | No | 0 | 0 | 0 | 0 | 0 | 0.56 |
| CHEMBL464767 | CCCCC[C@]1(CC)OO[C@@H](C(=C1)CC)CC(=O)OC | 284.39 | 9 | 4 | 0 | 79.93 | 44.76 | 3.51 | No | No | Yes | No | No | 0 | 0 | 0 | 0 | 0 | 0.55 |
| CHEMBL1566888 | Nc1ccc(cc1)S(=O)(=O)O | 173.19 | 1 | 3 | 2 | 40.71 | 88.77 | -0.09 | No | No | No | No | No | 0 | 1 | 0 | 0 | 2 | 0.56 |
| CHEMBL455737 | CC([C@H]1OC(=O)[C@H](C(C)C)N(C)C(=O)[C@H](OC(=O)[C@@H](N(C(=O)[C@H](OC(=O)[C@@H](N(C1=O)C)C(C)C)C(C)C)C)C)C(C)C)C | 611.77 | 5 | 9 | 0 | 173.91 | 139.83 | 3.08 | No | No | No | No | No | 2 | 3 | 0 | 1 | 2 | 0.17 |
| CHEMBL295069 | O[C@@H]1[C@H](O)[C@H](O[C@H]1[n+]1cccc(c1)C(=N)O)COP(=O)(OP(=O)(OC[C@H]1O[C@H]([C@@H]([C@@H]1O)OP(=O)(O)O)n1cnc2c1ncnc2N)[O-])O | 743.41 | 13 | 21 | 9 | 152.37 | 398.04 | -5.5 | No | No | No | No | No | 3 | 4 | 2 | 1 | 5 | 0.11 |
| CHEMBL1215502 | COc1c2O[C@@H]3[C@H](c2cc(c1OC)O)COc1c3ccc(c1)O | 316.31 | 2 | 6 | 2 | 81.68 | 77.38 | 2.1 | Yes | No | No | Yes | Yes | 0 | 0 | 0 | 0 | 0 | 0.55 |
| CHEMBL453642 | COc1cc(O)ccc1[C@@H]1CC(=O)c2c(O1)c(C[C@H](C(=C)C)CC=C(C)C)c(cc2O)O | 438.51 | 7 | 6 | 3 | 125.34 | 96.22 | 4.55 | No | No | Yes | No | Yes | 0 | 0 | 0 | 0 | 1 | 0.55 |
| CHEMBL2063150 | OC[C@]12CC[C@@]3([C@]([C@H]2[C@@H]([C@H]([C@H]1C(C)C)O)O)(C)CC=C1[C@H]3[C@@H](O)CC2=C1CC[C@@H](C2(C)C)O)C | 474.67 | 2 | 5 | 5 | 134.77 | 101.15 | 3.39 | No | No | No | No | No | 0 | 2 | 0 | 0 | 0 | 0.55 |
| CHEMBL1928406 | OC[C@H]1O[C@@H](OC[C@H]2O[C@@H](Oc3c(CCC(=O)O)ccc4c3C=CC(O4)(C)C)[C@@H]([C@H]([C@@H]2O)O)O)[C@@H]([C@H]([C@@H]1O)O)O | 572.56 | 9 | 14 | 8 | 133.44 | 225.06 | -1.51 | No | No | No | No | No | 3 | 4 | 1 | 1 | 3 | 0.11 |
| CHEMBL2375790 | CCCCCCCCCC(=O)O[C@@]12[C@H](OC(=O)/C(=C/C)/C)[C@@H](C)[C@]3([C@H]([C@@H]1C2(C)C)C=C(CO)C[C@@H]1[C@H]3C=C(C1=O)C)O | 584.78 | 14 | 7 | 2 | 165.39 | 110.13 | 5.53 | No | No | No | No | Yes | 1 | 4 | 1 | 1 | 1 | 0.55 |
| CHEMBL504184 | O[C@H]1CCN[C@H](C1)C(=O)O | 145.16 | 1 | 4 | 3 | 38.49 | 69.56 | -1.35 | No | No | No | No | No | 0 | 3 | 0 | 0 | 2 | 0.55 |
| CHEMBL471086 | CC(=CCc1c(O)cc(c(c1/C=C/c1ccccc1)CC=C(C)C)O)C | 348.48 | 6 | 2 | 2 | 113.3 | 40.46 | 5.82 | Yes | Yes | Yes | No | Yes | 1 | 1 | 0 | 1 | 1 | 0.55 |
| CHEMBL465434 | COC(=O)[C@@H]([C@@H]1[C@@H](O)C[C@]2([C@@]1(C)CC[C@H]1C2=CC[C@@H]2[C@]1(C)CCC(=O)C2(C)C)C)C/C=C/C(O)(C)C | 500.71 | 6 | 5 | 2 | 144.53 | 83.83 | 4.95 | No | No | No | No | Yes | 1 | 4 | 0 | 0 | 0 | 0.55 |
| CHEMBL1699927 | COc1c2ccoc2c(c2c1ccc(=O)o2)OCC(C(=C)C)O | 316.31 | 5 | 6 | 1 | 85.16 | 82.04 | 2.64 | Yes | Yes | Yes | No | No | 0 | 0 | 0 | 0 | 0 | 0.55 |
| CHEMBL452243 | C=C([C@@H]1CC[C@@]2([C@@](C1)(O)[C@](C)(O)CCC2)C)C(=O)O | 268.35 | 2 | 4 | 3 | 73.43 | 77.76 | 2.01 | No | No | No | No | No | 0 | 0 | 0 | 0 | 0 | 0.56 |
| CHEMBL102201 | CC(=CC(=O)O)CC[C@@H]1C(=C)CC[C@H]2[C@@]1(C)CCCC2(C)C | 304.47 | 4 | 2 | 1 | 94.33 | 37.3 | 4.97 | No | Yes | Yes | No | No | 1 | 0 | 0 | 0 | 1 | 0.85 |
| CHEMBL1956348 | COC1=CC(=O)C[C@H]([C@@]21Oc1c(C2=O)c(OC)cc(c1Cl)O)C | 338.74 | 2 | 6 | 1 | 82.12 | 82.06 | 2.06 | Yes | No | Yes | No | Yes | 0 | 0 | 0 | 0 | 0 | 0.56 |
| CHEMBL2251611 | CCOC(=O)c1cccnc1 | 151.16 | 3 | 3 | 0 | 40.32 | 39.19 | 1.32 | No | No | No | No | No | 0 | 1 | 0 | 0 | 1 | 0.55 |
| CHEMBL446639 | COc1ccc(cc1)[C@]1(O)[C@@H](O)C(=Nc2c1cccc2)O | 285.29 | 2 | 5 | 3 | 81.39 | 82.28 | 1.66 | No | No | No | No | No | 0 | 0 | 0 | 0 | 0 | 0.55 |
| CHEMBL2164627 | COc1cc2c(cc1OC)N1[C@@H]3[C@@]42CCN(C)CC2=CCO[C@H]([C@@H]3[C@H]2CC4=O)CC1=O | 424.49 | 2 | 6 | 0 | 121.16 | 68.31 | 1.59 | No | No | No | Yes | No | 0 | 0 | 0 | 0 | 0 | 0.55 |
| CHEMBL554759 | C/C=C(/C(=O)O[C@H]1[C@H]2OC(=O)[C@@H]([C@@H]3[C@@]42CO[C@]([C@@H]4[C@@]2([C@@H]1C(=CC(=O)[C@H]2O)C)C)([C@@H]([C@@H]3C)O)O)OC(=O)C)\C | 534.55 | 5 | 11 | 3 | 128.53 | 165.89 | 0.99 | No | No | No | No | No | 2 | 2 | 1 | 1 | 2 | 0.17 |
| CHEMBL406378 | OC/C=C/c1cc2c(c(c1)OC)O[C@@H]([C@H]2CO)c1ccc(c(c1)OC)O | 358.39 | 6 | 6 | 3 | 97.31 | 88.38 | 2.26 | No | No | No | Yes | No | 0 | 0 | 0 | 0 | 0 | 0.55 |
| CHEMBL2051971 | OC[C@H]1O[C@H]([C@H]([C@H]1O)O)n1cnc2c1ncnc2N | 267.24 | 2 | 7 | 4 | 62.67 | 139.54 | -1.45 | No | No | No | No | No | 0 | 1 | 0 | 1 | 0 | 0.55 |
| CHEMBL1802168 | COC[C@@H]1[C@@H]2CC[C@@H]3[C@]4(C1=O)[C@H]2O[C@@H]1[C@@]23[C@@H](O)CCC([C@H]2[C@@H]([C@@]4(O)O1)O)(C)C | 394.46 | 2 | 7 | 3 | 96.58 | 105.45 | 0.95 | No | No | No | No | No | 0 | 0 | 0 | 0 | 0 | 0.55 |
| CHEMBL490699 | CC(CCCCCCCCCCCCC(=O)O[C@H]([C@H]([C@H](C[C@H](c1coc(n1)Cc1cnco1)O)O)O)C)C | 536.7 | 22 | 9 | 3 | 146.7 | 139.05 | 5.04 | No | No | No | No | Yes | 1 | 3 | 1 | 1 | 2 | 0.55 |
| CHEMBL463464 | OC[C@]1(/C=C/C=C(C)C)OC(=O)[C@@H]2[C@@H]1CC[C@]1(C)O[C@H]1CCC2=C | 332.43 | 3 | 4 | 1 | 94.1 | 59.06 | 3.25 | No | No | Yes | No | No | 0 | 0 | 0 | 0 | 0 | 0.55 |
| CHEMBL466928 | OC[C@@H]([C@H](c1cc(OC)c(c(c1)OC)O)O)O[C@@H]1O[C@H](CO)[C@H]([C@@H]([C@H]1O)O)O | 406.38 | 8 | 11 | 7 | 91.9 | 178.53 | -1.58 | No | No | No | No | No | 2 | 1 | 1 | 1 | 4 | 0.17 |
| CHEMBL185885 | OCc1ccc(o1)C=O | 126.11 | 2 | 3 | 1 | 30.22 | 50.44 | 0.19 | No | No | No | No | No | 0 | 3 | 0 | 0 | 1 | 0.55 |
| CHEMBL2333674 | OCC1=C(C)C[C@@H](OC1=O)[C@@]([C@]1(O)CC[C@@]2([C@]1(C)CC[C@H]1[C@H]2CC=C2[C@]1(C)C(=O)C=CC2)O)(O)C | 486.6 | 3 | 7 | 4 | 130.44 | 124.29 | 2.67 | No | No | No | No | No | 0 | 3 | 0 | 0 | 0 | 0.55 |
| CHEMBL3087312 | CCC(=O)[C@@H](c1oc([C@@H]([C@@H]2O[C@@](O)([C@H]([C@H]([C@@H]2C)O)C)[C@H](c2oc(CC)c(c(=O)c2C)C)C)C)c(c(=O)c1C)C)C | 558.7 | 8 | 8 | 2 | 156.4 | 127.18 | 4.33 | No | No | No | No | Yes | 1 | 3 | 0 | 0 | 0 | 0.55 |
| CHEMBL1320034 | OC(=O)C=Cc1ccc(c(c1)O)O | 180.16 | 2 | 4 | 3 | 47.16 | 77.76 | 0.93 | No | No | No | No | No | 0 | 0 | 0 | 0 | 1 | 0.56 |
| CHEMBL158766 | Cc1ccc(c(c1)O)O | 124.14 | 0 | 2 | 2 | 35.45 | 40.46 | 1.33 | No | No | No | No | Yes | 0 | 3 | 0 | 0 | 1 | 0.55 |
| CHEMBL388661 | COc1cc(O)c(c2c1C(=O)[C@@H](O)[C@H](O2)c1ccc(cc1O)O)C[C@H](C(=C)C)CCC(O)(C)C | 472.53 | 8 | 8 | 5 | 128.18 | 136.68 | 2.98 | No | No | No | No | Yes | 0 | 0 | 0 | 1 | 0 | 0.55 |
| CHEMBL443408 | OCC1=CCC2CC1C2(C)C | 152.23 | 1 | 1 | 1 | 46.38 | 20.23 | 2.4 | No | No | No | No | No | 0 | 1 | 0 | 0 | 2 | 0.55 |
| CHEMBL2409057 | COc1cc2O[C@@H](CCc2cc1O)c1ccc(c(c1)O)O | 288.3 | 2 | 5 | 3 | 77.64 | 79.15 | 2.29 | Yes | No | No | Yes | Yes | 0 | 0 | 0 | 0 | 0 | 0.55 |
| CHEMBL480278 | CC(=C)[C@@H]1[C@@H]2OC(=O)C(=C2)CC[C@H]([C@@H](c2oc1cc2C)O)C(=C)C | 328.4 | 2 | 4 | 1 | 92.89 | 59.67 | 3.36 | No | Yes | Yes | No | Yes | 0 | 0 | 0 | 0 | 0 | 0.55 |
| CHEMBL460647 | CC(=CCc1c(O)cc(c2c1OC(CC2=O)c1ccc(cc1)O)O)C | 340.37 | 3 | 5 | 3 | 95.29 | 86.99 | 3.26 | Yes | No | Yes | Yes | Yes | 0 | 0 | 0 | 0 | 0 | 0.55 |
| CHEMBL512755 | C[C@H]1CCC(=O)C(=C)CC[C@H](C(C=C1)(C)C)O | 236.35 | 0 | 2 | 1 | 72.26 | 37.3 | 2.96 | No | No | No | No | No | 0 | 0 | 0 | 0 | 0 | 0.55 |
| CHEMBL1083820 | Oc1cc2O[C@@H](CC(=O)c2c(c1)O)c1ccccc1O | 272.25 | 1 | 5 | 3 | 71.57 | 86.99 | 1.86 | Yes | No | No | No | Yes | 0 | 0 | 0 | 0 | 0 | 0.55 |
| CHEMBL2287145 | COC(=O)C=Cc1ccc(c(c1)OC)O | 208.21 | 4 | 4 | 1 | 55.95 | 55.76 | 1.76 | No | No | No | No | No | 0 | 0 | 0 | 0 | 0 | 0.55 |
| CHEMBL2337120 | COc1cc(/C=C(\c2cc(O)c(cc2CCC(=NCCc2ccc(cc2)O)O)O)/C(=NCCc2ccc(cc2)O)O)cc(c1O)OC | 642.69 | 14 | 11 | 7 | 183.01 | 184.79 | 4.83 | No | No | No | No | No | 3 | 4 | 2 | 2 | 5 | 0.17 |
| CHEMBL2205288 | OC[C@H]1O[C@@H](O[C@@]23C[C@@H]4[C@@]3(CO)[C@@H]3O[C@@]2(C)C[C@]4(O3)O)[C@@H]([C@H]([C@@H]1O)O)O | 376.36 | 4 | 10 | 6 | 79.62 | 158.3 | -1.77 | No | No | No | No | No | 1 | 1 | 1 | 1 | 3 | 0.55 |
| CHEMBL228831 | CO[C@@H]1C[C@@H](C)O[C@@H]2[C@@]1(O)O[C@@H]1C[C@@]3(C)C(=C[C@H]1O2)CC[C@@H]1[C@@H]3CC[C@]2([C@]1(O)C[C@@H]([C@@H]2C1=CC(=O)OC1)OC(=O)C)C | 588.69 | 4 | 10 | 2 | 148.98 | 129.98 | 2.58 | No | No | No | No | No | 1 | 3 | 0 | 0 | 0 | 0.55 |
| CHEMBL447310 | OC(=N)Cc1ccc(cc1)O | 151.16 | 2 | 3 | 3 | 43.4 | 64.31 | 1.12 | No | No | No | No | No | 0 | 1 | 0 | 0 | 1 | 0.55 |
| CHEMBL471473 | COc1cc2[C@H]3O[C@@H]3CCCC(=O)CCC[C@@H](OC(=O)c2c(c1)O)C | 348.39 | 1 | 6 | 1 | 91.52 | 85.36 | 2.54 | No | No | No | No | No | 0 | 0 | 0 | 0 | 0 | 0.55 |
| CHEMBL504190 | Oc1ccc(cc1)[C@@H]1Oc2cc(O)cc(c2C(=O)[C@H]1c1c(O)cc(c2c1oc(c1ccc(c(c1)O)O)c(c2=O)O)O)O | 572.47 | 3 | 12 | 8 | 147.65 | 218.35 | 2.59 | No | No | Yes | No | No | 3 | 2 | 1 | 1 | 3 | 0.17 |
| CHEMBL495849 | COC(=O)Cc1c(cc(cc1O)O)OC(=O)c1ccc(c(c1)O)O | 334.28 | 6 | 8 | 4 | 81.8 | 133.52 | 1.39 | No | No | No | No | No | 0 | 0 | 0 | 1 | 0 | 0.55 |
| CHEMBL1765415 | Cc1cc(O)c(c(c1c1c(C)cc(c(c1O)C(=O)c1c(O)ccc(c1C)O)O)O)C(=O)c1c(O)ccc(c1C)O | 546.52 | 5 | 10 | 8 | 147.67 | 195.98 | 3.76 | No | No | Yes | No | No | 2 | 2 | 1 | 1 | 3 | 0.17 |
| CHEMBL3099359 | OCC(C(=O)O)(CO)N | 135.12 | 3 | 5 | 4 | 28.18 | 103.78 | -2.59 | No | No | No | No | No | 0 | 4 | 0 | 0 | 3 | 0.55 |
| CHEMBL462902 | CC(=O)O[C@@H]1C[C@H]2C(C)(C)C(=O)CC[C@@]2([C@@H]2[C@]1(C)[C@@]13O[C@@H]1C(=O)O[C@H]([C@@]3(CC2)C)c1ccoc1)C | 484.58 | 3 | 7 | 0 | 126.52 | 95.34 | 3.89 | No | No | No | No | No | 0 | 2 | 0 | 0 | 0 | 0.55 |
| CHEMBL1910875 | CC/C=C/[C@H]1C/C=C/C=C[C@H](OC)[C@@H](O)[C@@H](O)[C@@H](C)/C=C/C[C@H](/C=C/C=C(/C=C/C(=O)O1)\C)C | 470.64 | 3 | 5 | 2 | 140.78 | 75.99 | 4.44 | No | No | No | No | Yes | 0 | 2 | 0 | 0 | 1 | 0.55 |
| CHEMBL478951 | CC1=CCCC(=C)[C@H]2[C@H](CC1)[C@H](C/C=C/C(O)(C)C)C(=O)OC2 | 318.45 | 3 | 3 | 1 | 95.09 | 46.53 | 3.6 | No | Yes | Yes | No | No | 0 | 0 | 0 | 0 | 0 | 0.55 |
| CHEMBL1256379 | Oc1cccc(c1)C(=O)c1cc(O)c(c(c1)O)O | 246.22 | 2 | 5 | 4 | 64.41 | 97.99 | 1.35 | Yes | No | No | No | Yes | 0 | 0 | 0 | 0 | 0 | 0.55 |
| CHEMBL513879 | COC(=O)[C@@H](CC(=O)C[C@H](C1=CC[C@@]2([C@]1(C)CC=C1[C@H]2CC[C@@H]2[C@]1(C)CC[C@@H](C2(C)C)O)C)C)C | 484.71 | 7 | 4 | 1 | 143.33 | 63.6 | 5.75 | No | No | No | No | Yes | 1 | 4 | 0 | 1 | 1 | 0.55 |
| CHEMBL1922169 | O=C(CC(C(=O)O)C)C[C@H]([C@H]1CC(=O)[C@@]2([C@]1(C)C[C@H](O)C1=C2[C@@H](O)C[C@@H]2[C@]1(C)CCC(=O)C2(C)C)C)C | 516.67 | 6 | 7 | 3 | 141.05 | 128.97 | 3.07 | No | No | No | No | Yes | 1 | 3 | 0 | 0 | 0 | 0.56 |
| CHEMBL3186302 | CC1OC(=C(C1=O)O)C | 128.13 | 0 | 3 | 1 | 31.22 | 46.53 | 0.56 | No | No | No | No | No | 0 | 3 | 0 | 0 | 1 | 0.85 |
| CHEMBL376835 | C=C(C(C)C)CC[C@H]([C@H]1CC[C@@]2([C@]1(C)CC[C@@]13[C@H]2CC[C@@H]2[C@]3(C1)CCC(=O)C2(C)C)C)C | 438.73 | 5 | 1 | 0 | 138.99 | 17.07 | 7.84 | No | No | No | No | No | 1 | 3 | 0 | 1 | 2 | 0.55 |
| CHEMBL450107 | CCCCCCCCCCCCC(C(CCC(C1CCC(O1)CCCCCCC[C@H](CC1=C[C@@H](OC1=O)C)O)O)O)O | 596.88 | 26 | 7 | 4 | 172.67 | 116.45 | 7.03 | No | No | No | No | Yes | 1 | 4 | 1 | 1 | 2 | 0.55 |
| CHEMBL2332222 | COC(=O)[C@@H]1[C@@H](c2ccccc2)[C@]2([C@]([C@@H]1O)(OC)c1c(O2)cc(cc1OC)OC)c1ccc2c(c1)OCO2 | 520.53 | 7 | 9 | 1 | 133.83 | 101.91 | 3.4 | No | No | Yes | Yes | Yes | 1 | 2 | 0 | 0 | 0 | 0.55 |
| CHEMBL1689080 | CC1=CC[C@@]2(C)C(=O)C[C@@H]([C@@H]2CC[C@]2([C@H](CC1)O2)C)C(=C)C | 302.45 | 1 | 2 | 0 | 92.03 | 29.6 | 4.12 | No | Yes | Yes | No | No | 0 | 0 | 0 | 0 | 0 | 0.55 |
| CHEMBL2087217 | C/C=C(/C(=O)O[C@H]1CC(=C2[C@@H]([C@@H]3[C@@H]1[C@H](C)C(=O)O3)C(=CC2=O)C)C)\C | 344.4 | 3 | 5 | 0 | 93.26 | 69.67 | 2.8 | No | No | No | No | No | 0 | 0 | 0 | 0 | 0 | 0.55 |
| CHEMBL2269698 | CC[C@@H](C(=O)O[C@H](C1=CC(=O)OC1)C[C@@]1(C)[C@H](C)C[C@@H]([C@@]2([C@@H]1[C@@H](CC[C@@]12CO1)OC(=O)/C(=C/C)/C)COC(=O)C)OC(=O)C)C | 632.74 | 15 | 11 | 0 | 163.18 | 144.03 | 4.4 | No | Yes | No | No | No | 2 | 3 | 2 | 1 | 2 | 0.17 |
| CHEMBL301523 | N[C@H](C(=O)O)Cc1ccccc1 | 165.19 | 3 | 3 | 2 | 45.5 | 63.32 | -0.01 | No | No | No | No | No | 0 | 0 | 0 | 0 | 1 | 0.55 |
| CHEMBL496712 | CNc1ccccc1C(=O)O[C@@H]1[C@@H](C)[C@]2(O)[C@@H]3C=C(C(=O)[C@@H]3[C@@H](C(=C[C@H]2[C@H]2[C@]1(OC(=O)C)C2(C)C)C)O)C | 523.62 | 6 | 7 | 3 | 142.19 | 122.16 | 3.06 | No | No | No | No | Yes | 1 | 3 | 0 | 0 | 0 | 0.55 |
| CHEMBL3589062 | OC[C@H]1O[C@H]([C@@H]([C@H]([C@@H]1O)O)O)n1c2c(c3c1cccc3)c1C(=O)N=C(c1c1c2[nH]c2c1cccc2)O | 487.46 | 2 | 8 | 6 | 134.54 | 160.53 | 1.48 | No | No | Yes | No | No | 1 | 2 | 1 | 1 | 2 | 0.55 |
| CHEMBL6355 | O=c1oc2ccccc2c2c1cccc2 | 196.2 | 0 | 2 | 0 | 59.99 | 30.21 | 2.9 | Yes | No | No | No | No | 0 | 0 | 0 | 0 | 1 | 0.55 |
| CHEMBL442852 | OCC[C@]1(O)C[C@@H](O)C=CC1=O | 172.18 | 2 | 4 | 3 | 41.71 | 77.76 | -0.37 | No | No | No | No | No | 0 | 1 | 0 | 0 | 1 | 0.55 |
| CHEMBL343316 | BrC[C@@](C(=C)Cl)(CCC(=C(C)C)Br)Cl | 364.93 | 5 | 0 | 0 | 74.61 | 0 | 4.85 | No | No | Yes | No | No | 1 | 0 | 0 | 0 | 2 | 0.55 |
| CHEMBL601291 | Oc1ccc(cc1)[C@H]1c2c(O)cc(cc2[C@H]2c3c4[C@H]1[C@@H](c1ccc(cc1)O)[C@@H](c4c(cc3O[C@@H]2c1ccc(cc1)O)O)c1cc(O)cc(c1)O)O | 680.7 | 4 | 9 | 8 | 189.88 | 171.07 | 5.12 | No | No | No | No | No | 2 | 4 | 1 | 2 | 5 | 0.17 |
| CHEMBL1922185 | OC(=O)/C=C/C=C/C(=O)O | 142.11 | 3 | 4 | 2 | 33.55 | 74.6 | 0.19 | No | No | No | No | No | 0 | 3 | 0 | 0 | 1 | 0.85 |
| CHEMBL1236395 | OC[C@H]1O[C@H](O[C@H]2O[C@H](CO)[C@H]([C@@H]([C@H]2O)O)O)[C@@H]([C@H]([C@@H]1O)O)O | 342.3 | 4 | 11 | 8 | 68.12 | 189.53 | -3.47 | No | No | No | No | No | 2 | 1 | 1 | 1 | 4 | 0.17 |
| CHEMBL29711 | OC[C@@]1(O)CC[C@]23C[C@H]1C[C@@H]2CC[C@@H]1[C@]3(C)CC[C@H]([C@@]1(C)CO)O | 338.48 | 2 | 4 | 4 | 93.7 | 80.92 | 2.37 | No | No | No | No | No | 0 | 0 | 0 | 0 | 0 | 0.55 |
| CHEMBL553673 | CCCCCC(=N[C@H](C(=N[C@H]1[C@@H](C)OC(=O)[C@@H](N=C(O)[C@H](Cc2ccc(cc2)O)N(C)C(=O)[C@@H](N2C(=O)[C@@H](N=C([C@@H](N=C1O)Cc1ccc(cc1)O)O)CC[C@H]2O)CC(C)C)C(C)C)O)CCC(=O)O)O | 994.14 | 17 | 19 | 9 | 288.07 | 327.86 | 3.99 | No | No | No | No | No | 3 | 3 | 2 | 1 | 6 | 0.11 |
| CHEMBL2431871 | O[C@H]1OC[C@@]2(C1)CC[C@@]1(O2)[C@H](C)CC[C@@H]2[C@]1(C)CCCC2(C)C | 322.48 | 0 | 3 | 1 | 92.69 | 38.69 | 3.9 | No | No | No | No | No | 0 | 0 | 0 | 0 | 0 | 0.55 |
| CHEMBL507185 | CCCCCCCCCC[C@H]([C@H]1CC[C@@H](O1)[C@@H](CCCC[C@@H](CCCCCCC[C@H](CC1=C[C@@H](OC1=O)C)O)O)O)O | 596.88 | 26 | 7 | 4 | 172.67 | 116.45 | 6.97 | No | No | No | No | Yes | 1 | 4 | 1 | 1 | 2 | 0.55 |
| CHEMBL466583 | OC(=O)[C@H]1O[C@@H](Oc2c(O)cc(c3c2oc(cc3=O)c2ccc(c(c2)O)O)O)[C@@H]([C@H]([C@@H]1O)OS(=O)(=O)O)O | 558.42 | 6 | 16 | 8 | 121.23 | 279.33 | -0.68 | No | No | No | No | No | 3 | 1 | 1 | 1 | 3 | 0.11 |
| CHEMBL1821996 | NC(=N)NCCCCc1nccc2c1n(O)c1c2ccc(c1)Br | 376.25 | 6 | 3 | 4 | 95.78 | 99.95 | 2.51 | Yes | No | No | No | No | 0 | 0 | 0 | 0 | 0 | 0.55 |
| CHEMBL481041 | O=C[C@@]12C[C@@H](OC(=O)C)[C@H](C(C)C)[C@H](OC(=O)C)[C@H]3[C@](CC[C@H]2O1)(C)O3 | 368.42 | 6 | 7 | 0 | 92.12 | 94.73 | 1.99 | No | No | No | No | No | 0 | 0 | 0 | 0 | 0 | 0.55 |
| CHEMBL464742 | COc1oc(C/C=C(/CC/C=C(\C/C(=C/Cc2ccccc2)/C)/C)\C)c(c(=O)c1OC)C | 436.58 | 11 | 4 | 0 | 133.45 | 48.67 | 1.99 | No | No | No | No | No | 0 | 0 | 0 | 0 | 0 | 0.55 |
| CHEMBL1095333 | CC(=O)O[C@H]1CC(=CC[C@]2([C@H](CC[C@]3([C@H]1O3)C)C(=C(C)C)C(=O)C2)C)C | 360.49 | 2 | 4 | 0 | 102.93 | 55.9 | 3.89 | No | No | No | No | No | 0 | 0 | 0 | 0 | 0 | 0.55 |
| CHEMBL174243 | OCC1OC(C(C(C1O)O)O)c1c(OC)cc2c(c1O)c(=O)cc(o2)c1ccc(cc1)O | 446.4 | 4 | 10 | 6 | 111.08 | 170.05 | 0.35 | No | No | No | No | No | 1 | 0 | 1 | 1 | 2 | 0.55 |
| CHEMBL1915271 | OC(=O)CC[C@H]([C@H]1CC[C@@H]2[C@]1(C)[C@@H](O)C[C@H]1[C@H]2[C@H](O)CC2=CC(=O)C=C[C@]12C)C | 402.52 | 4 | 5 | 3 | 111.85 | 94.83 | 2.91 | No | No | No | No | Yes | 0 | 0 | 0 | 0 | 0 | 0.56 |
| CHEMBL3039487 | Cc1oc2cc(O)c(c(c2c(=O)c1)O)C | 206.19 | 0 | 4 | 2 | 56.46 | 70.67 | 1.74 | Yes | No | No | No | Yes | 0 | 0 | 0 | 0 | 0 | 0.55 |
| CHEMBL1651024 | O=C1C[C@H]2C(C)(C)CCC[C@@]2(c2c1cc(C(C)C)c(c2)O)O | 302.41 | 1 | 3 | 2 | 88.41 | 57.53 | 3.51 | No | No | No | Yes | No | 0 | 0 | 0 | 0 | 0 | 0.55 |
| CHEMBL3109385 | OC(=O)[C@H]([C@@H]1CC[C@]2([C@H]1[C@H]1CC[C@H]3[C@@]([C@]1(C)CC2)(C)CC[C@@H]1[C@]3(C)CCC(=O)C1(C)C)C)C | 456.7 | 2 | 3 | 1 | 136.43 | 54.37 | 6.33 | No | No | Yes | No | No | 1 | 3 | 0 | 1 | 1 | 0.85 |
| CHEMBL3400306 | OC[C@H]1O[C@@H](O[C@H](CC[C@H]2C(=CC(=O)CC2(C)C)C)C)[C@@H]([C@H]([C@@H]1O)O)O | 372.45 | 6 | 7 | 4 | 95.5 | 116.45 | 0.81 | No | No | No | No | No | 0 | 0 | 0 | 0 | 0 | 0.55 |
| CHEMBL1651071 | CCO[C@@H]1OC[C@@]2([C@H]3[C@]1(CCC2)c1cc(O)c(cc1CC3)C(C)C)C | 344.49 | 3 | 3 | 1 | 101.31 | 38.69 | 4.61 | No | No | Yes | Yes | No | 0 | 0 | 0 | 0 | 1 | 0.55 |
| CHEMBL17976 | COc1cc(ccc1O)CC(=O)OCC1=C[C@H]2[C@H]3O[C@]4(O[C@]2([C@H]2[C@@](C1)(O)C(=O)C(=C2)C)[C@@H](C[C@@]3(O4)C(=C)C)C)Cc1ccccc1 | 628.71 | 9 | 9 | 2 | 169.03 | 120.75 | 4.42 | No | No | Yes | No | Yes | 1 | 3 | 0 | 0 | 1 | 0.55 |
| CHEMBL337824 | CCCCCCCC=CCC#CC#CCCCO | 246.39 | 9 | 1 | 1 | 80.84 | 20.23 | 4.65 | Yes | No | No | No | No | 1 | 0 | 0 | 0 | 2 | 0.55 |
| CHEMBL253982 | COc1c2c(CCc3c2ccc(c3)O)cc(c1OC)O | 272.3 | 2 | 4 | 2 | 76.73 | 58.92 | 2.73 | Yes | No | No | Yes | Yes | 0 | 0 | 0 | 0 | 0 | 0.55 |
| CHEMBL402794 | CCCC[C@H](COC(=O)c1ccccc1C(=O)OC[C@H](CCCC)CC)CC | 390.56 | 16 | 4 | 0 | 116.3 | 52.6 | 6.1 | No | No | Yes | No | Yes | 1 | 1 | 1 | 1 | 2 | 0.55 |
| CHEMBL477898 | CO[C@@H]1Oc2c(O)cccc2[C@]2([C@H]1Oc1cc(OC)c(c(c1C2=O)O)C)O | 374.34 | 2 | 8 | 3 | 92.32 | 114.68 | 1.68 | No | No | No | No | Yes | 0 | 0 | 0 | 0 | 0 | 0.55 |
| CHEMBL448500 | OCCc1ccccc1 | 122.16 | 2 | 1 | 1 | 37.38 | 20.23 | 1.64 | Yes | No | No | No | No | 0 | 3 | 0 | 0 | 2 | 0.55 |
| CHEMBL1172581 | CO[C@H]1[C@@H](O[C@H]2CC[C@]3([C@](C2)(O)CC[C@@H]2[C@@H]3CC[C@]3([C@]2(O)CC[C@@H]3C2=CC(=O)OC2)C)C=O)O[C@@H]([C@H]([C@H]1O)O)C | 564.66 | 5 | 10 | 4 | 142.11 | 151.98 | 1.32 | No | No | No | No | No | 1 | 3 | 1 | 1 | 1 | 0.55 |
| CHEMBL369243 | CCOC(=O)CCC(=O)OCC | 174.19 | 7 | 4 | 0 | 43.14 | 52.6 | 1.26 | No | No | No | No | No | 0 | 0 | 0 | 0 | 1 | 0.55 |
| CHEMBL1979562 | COC[C@@]12CN(CC)C3[C@]4([C@@H]2[C@@H](OC)[C@@H]3[C@@]2([C@@H]3[C@H]4C[C@@]([C@@H]3OC(=O)c3ccccc3)([C@H]([C@@H]2O)OC)O)OC(=O)C)[C@H](C[C@H]1O)OC | 645.74 | 11 | 12 | 3 | 165.5 | 153.45 | 1.05 | No | No | No | No | No | 2 | 3 | 2 | 1 | 3 | 0.17 |
| CHEMBL1081411 | COc1cc2OC(=O)c3c(Oc2c(c1Cl)C)cc(c(c3C)Cl)OC | 369.2 | 2 | 5 | 0 | 90.85 | 53.99 | 4.44 | No | Yes | Yes | No | No | 0 | 0 | 0 | 0 | 0 | 0.55 |
| CHEMBL2426312 | CCCC(=O)O[C@]1(C)CC[C@H](OC(=O)Cc2ccccc2)[C@@](C[C@H]2O[C@@H]1[C@@H]1[C@H](C[C@@H](C(=C)[C@H]21)O)C(C)C)(C)O | 542.7 | 9 | 7 | 2 | 150.82 | 102.29 | 4.47 | No | No | No | No | Yes | 1 | 3 | 0 | 0 | 0 | 0.55 |
| CHEMBL43612 | O=C1C=CC(=O)c2c1c(O)ccc2 | 174.15 | 0 | 3 | 1 | 46.27 | 54.37 | 1.36 | No | No | No | No | No | 0 | 1 | 0 | 0 | 1 | 0.55 |
| CHEMBL110442 | CCCCCCCCCCCC[C@H]([C@H]1CC[C@H](O1)[C@H](CCCCCCC(CCCCC[C@H](CC1=C[C@@H](OC1=O)C)O)O)O)O | 624.93 | 28 | 7 | 4 | 182.29 | 116.45 | 7.67 | No | No | No | No | Yes | 1 | 4 | 1 | 1 | 3 | 0.55 |
| CHEMBL504463 | OC(=NCc1ccccc1)NCc1ccccc1 | 240.3 | 5 | 2 | 2 | 73.57 | 44.62 | 2.81 | No | No | No | Yes | No | 0 | 0 | 0 | 0 | 0 | 0.55 |
| CHEMBL471281 | COc1ccc2c(c1)c1c3OCOc3cc3c1c(C2=O)ncc3 | 305.28 | 1 | 5 | 0 | 83.17 | 57.65 | 2.87 | Yes | No | Yes | Yes | Yes | 0 | 0 | 0 | 0 | 0 | 0.55 |
| CHEMBL490622 | COC(=O)/C(=C/C=C/C(=C/C=C/C(=C/1\C(=O)C[C@@H]2[C@]1(C)CC[C@@H]1[C@]2(C)CC[C@@H](C1(C)C)O)/C)/C)/C | 480.68 | 6 | 4 | 1 | 144.29 | 63.6 | 6.21 | No | No | Yes | No | Yes | 1 | 4 | 0 | 1 | 1 | 0.55 |
| CHEMBL2063164 | OC(=O)C[C@H]1OC([C@H]2[C@@]1(C)[C@H]1[C@H](O)C[C@H]3[C@@]([C@@]1(CC2)C)(C)CC[C@@]1([C@@H]3[C@@H](C[C@@H]1O)C(=C)C)C(=O)O)(C)C | 518.68 | 4 | 7 | 4 | 141.23 | 124.29 | 3.79 | No | No | No | No | No | 1 | 3 | 0 | 0 | 0 | 0.56 |
| CHEMBL278958 | c1cccs1 | 84.14 | 0 | 0 | 0 | 24.32 | 28.24 | 1.51 | No | No | No | No | No | 0 | 3 | 0 | 0 | 3 | 0.55 |
| CHEMBL183745 | COc1cc(O)c2c(c1)oc(cc2=O)c1ccc(c(c1)O)O | 300.26 | 2 | 6 | 3 | 80.48 | 100.13 | 2.13 | Yes | No | Yes | Yes | Yes | 0 | 0 | 0 | 0 | 0 | 0.55 |
| CHEMBL465251 | O=C1C=C2[C@@](c3c1c(C(=O)O)c(O)c(c3)O)(C)CC[C@@]1([C@]2(C)CC[C@@]2([C@H]1C[C@](CC2)(C)C(=O)O)C)C | 496.59 | 2 | 7 | 4 | 135.63 | 132.13 | 4.48 | No | No | Yes | No | Yes | 0 | 4 | 0 | 1 | 1 | 0.56 |
| CHEMBL369474 | COc1cc2O[C@H](Cc2c2c1c(=O)c1c(o2)c(O)ccc1)[C@@]1(C)CO1 | 340.33 | 2 | 6 | 1 | 91.28 | 81.43 | 2.64 | Yes | Yes | Yes | Yes | Yes | 0 | 0 | 0 | 0 | 0 | 0.55 |
| CHEMBL1375576 | COc1cc2CCN[C@H](c2cc1O)Cc1ccc(cc1)Oc1cc(ccc1O)C[C@@H]1NCCc2c1cc(O)c(c2)OC | 568.66 | 8 | 8 | 5 | 169.28 | 112.44 | 4.44 | No | No | No | No | No | 1 | 3 | 0 | 0 | 1 | 0.55 |
| CHEMBL1513871 | C[C@]1(O)CC[C@H](CC1)C(O)(C)C | 172.26 | 1 | 2 | 2 | 50.47 | 40.46 | 1.68 | No | No | No | No | No | 0 | 0 | 0 | 0 | 1 | 0.55 |
| CHEMBL480283 | OC[C@H]1O[C@@H](OC(=O)c2cc(O)c(c(c2)O)O)[C@@H]([C@H]([C@@H]1O)O)O | 332.26 | 4 | 10 | 7 | 71.44 | 177.14 | -1.54 | No | No | No | No | No | 1 | 1 | 1 | 1 | 2 | 0.55 |
| CHEMBL490162 | COc1cc(ccc1OC(=O)C(C)C)[C@@H]1CC(=O)c2c(O1)cc(cc2O)O | 372.37 | 5 | 7 | 2 | 97.15 | 102.29 | 2.85 | Yes | No | Yes | No | Yes | 0 | 0 | 0 | 0 | 0 | 0.55 |
| CHEMBL563622 | C[C@@H]1C[C@H](C)[C@H]([C@@H]([C@@](C1)(C)O)C)c1c(O)ccnc1O | 279.37 | 1 | 4 | 3 | 80.4 | 73.58 | 2.49 | No | No | No | Yes | No | 0 | 0 | 0 | 0 | 0 | 0.55 |
| CHEMBL225011 | CC(=CC(=O)OC[C@H]1CC[C@@H]2[C@H]([C@]3([C@@]1(O)CCC3=O)C)OC(=O)C2=C)C | 362.42 | 4 | 6 | 1 | 94.67 | 89.9 | 2.26 | No | No | No | No | No | 0 | 0 | 0 | 0 | 0 | 0.55 |
| CHEMBL487567 | CC(=O)O[C@H]1CC[C@@H]2C(=CC[C@H]3[C@@]2(C)CC[C@@]2([C@]3(C)CC[C@@]3([C@H]2CC(C)(C)CC3)C)C)C1(C)C | 468.75 | 2 | 2 | 0 | 144.62 | 26.3 | 7.58 | No | No | No | No | No | 1 | 3 | 0 | 1 | 1 | 0.55 |
| CHEMBL2071396 | C[C@@H]1O[C@H](CC[C@@H]1O[C@H]1C=CC(=O)[C@@H](O1)C)O[C@@]1(C)CC(=O)[C@]2([C@](C1)(O)C=CC1=C2C(=O)c2c(C1=O)c(O)c(cc2)[C@H]1C[C@@H](O)[C@@H]([C@H](O1)C)O[C@@H]1O[C@@H](C)C(=O)C=C1N)O | 835.85 | 7 | 16 | 5 | 205.45 | 256.9 | 0.95 | No | No | No | No | No | 2 | 3 | 1 | 1 | 4 | 0.17 |
| CHEMBL3185053 | C=CC(CC/C=C(\CCC=C(C)C)/C)(O)C | 222.37 | 7 | 1 | 1 | 74 | 20.23 | 4.18 | No | No | Yes | No | No | 0 | 0 | 0 | 0 | 1 | 0.55 |
| CHEMBL1773905 | CC(=CCc1cc(O)cc(c1O)C=CC(O)(C)C)C | 262.34 | 4 | 3 | 3 | 79.92 | 60.69 | 3.13 | Yes | No | No | Yes | Yes | 0 | 0 | 0 | 0 | 0 | 0.55 |
| CHEMBL489353 | CO[C@@]1(CC=C(C)C)C(=C(CC=C(C)C)C(=O)[C@](C1=O)(O)C(=O)/C=C/c1ccccc1)O | 438.51 | 8 | 6 | 2 | 124.02 | 100.9 | 3.52 | Yes | Yes | Yes | No | Yes | 0 | 0 | 0 | 0 | 0 | 0.56 |
| CHEMBL464705 | O=C/C=C/c1cc(OC)c(c(c1)OC)OC/C=C(/CCC=C(C)C)\C | 344.44 | 10 | 4 | 0 | 103.33 | 44.76 | 4.54 | Yes | No | Yes | Yes | Yes | 0 | 0 | 0 | 0 | 1 | 0.55 |
| CHEMBL1096631 | Oc1ccc(cc1)/C=C/C(=O)O[C@@H]([C@@](C(=O)O)(Cc1ccc(cc1)O)O)C(=O)O | 402.35 | 9 | 9 | 5 | 99.94 | 161.59 | 1.23 | No | No | No | No | No | 0 | 0 | 1 | 1 | 1 | 0.11 |
| CHEMBL487996 | O=c1oc(C(C)C)c2c(c1)[C@@]1(C)[C@@H]3O[C@@H]3[C@@H]([C@]3([C@@H]1[C@@H]([C@@H]2O)OC3=O)C)O | 362.37 | 1 | 7 | 2 | 88.61 | 109.5 | 1.12 | No | No | No | No | No | 0 | 0 | 0 | 0 | 0 | 0.55 |
| CHEMBL1412041 | O[C@@H](c1ccccc1)[C@@H](N)C | 151.21 | 2 | 2 | 2 | 44.89 | 46.25 | 1.16 | No | No | No | No | No | 0 | 1 | 0 | 0 | 1 | 0.55 |
| CHEMBL2334482 | CC(=O)O[C@H]1C[C@@H]2C[C@]3([C@@H]1[C@]1(C)[C@@H](O)C[C@@H](C([C@H]1C(=O)C3)(C)C)O)C(=O)C2=C | 390.47 | 2 | 6 | 2 | 102.17 | 100.9 | 2.06 | No | No | No | No | No | 0 | 0 | 0 | 0 | 0 | 0.55 |
| CHEMBL458900 | OC[C@H]1O[C@@H](O[C@@H](C[C@@H](CCc2ccc(c(c2)O)O)O)CCc2ccc(cc2)O)[C@@H]([C@H]([C@@H]1O)O)O | 494.53 | 11 | 10 | 8 | 125.51 | 180.3 | 0.85 | No | No | No | No | No | 1 | 1 | 2 | 1 | 2 | 0.55 |
| CHEMBL517330 | OC[C@H]1O[C@@H](Oc2ccc3c(c2)OC[C@@H]2[C@H]3Oc3c2ccc(c3)OC)[C@@H]([C@H]([C@@H]1O)O)O | 432.42 | 4 | 9 | 4 | 105.29 | 127.07 | 0.81 | No | No | No | No | No | 0 | 0 | 0 | 0 | 0 | 0.55 |
| CHEMBL447144 | OCC1=C[C@@H]2[C@@]3(CC1)COC(=O)/C=C(\C)/CCO[C@H](/C=C/C=CC(=O)O[C@H]1[C@@]3(C)[C@@]3([C@H](O2)C1)CO3)[C@H](O)C | 530.61 | 2 | 9 | 2 | 136.72 | 124.05 | 2.17 | No | No | No | No | No | 1 | 3 | 0 | 0 | 0 | 0.55 |
| CHEMBL2087919 | OCC(=CCc1c(O[C@@H]2O[C@H](CO)[C@H]([C@@H]([C@H]2O)O)O)cc2c(c1O)c(=O)cc(o2)C)C | 438.43 | 6 | 10 | 6 | 108.5 | 170.05 | 0.37 | No | No | No | No | No | 1 | 1 | 1 | 1 | 2 | 0.55 |
| CHEMBL500352 | OC[C@H]1O[C@@H](OC(=O)[C@@]23CC[C@H]([C@@H]([C@H]3C3=CC[C@H]4[C@@]([C@@]3(CC2)C)(C)CC[C@@H]2[C@]4(C)C[C@H]([C@@H]([C@@]2(C)CO)O)O)C)C)[C@@H]([C@H]([C@@H]1O)O)O | 650.84 | 5 | 10 | 7 | 171.21 | 177.14 | 2.7 | No | No | No | No | No | 2 | 3 | 1 | 1 | 3 | 0.17 |
| CHEMBL510782 | COc1c(OC)cc2c3c1Oc1cc4c(cc1OC)CCN(=O)([C@H]4Cc1ccc(Oc4cc(C[C@@H]3N(CC2)C)ccc4OC)cc1)C | 638.75 | 4 | 9 | 0 | 189.44 | 88.05 | 4.71 | No | No | No | No | No | 1 | 4 | 0 | 1 | 2 | 0.55 |
| CHEMBL502028 | O[C@H]1C[C@]2(C)[C@H]3CC[C@]4([C@@](C3=CC(=O)[C@@]2(C[C@H]1O)O)(O)CC[C@@H]4[C@]([C@@H](CCC(O)(C)C)O)(O)C)C | 496.63 | 5 | 8 | 7 | 130.94 | 158.68 | 1.18 | No | No | No | No | No | 1 | 3 | 1 | 1 | 2 | 0.55 |
| CHEMBL1078144 | O=CC1=C[C@]2(O)CC([C@@H]([C@@H]2[C@@]2([C@@]1(O)[C@H](OC(=O)c1c(O)cc(c(c1C)Cl)OC)C2)C)O)(C)C | 480.94 | 5 | 8 | 4 | 119.94 | 133.52 | 2.59 | No | No | No | No | Yes | 0 | 1 | 0 | 1 | 0 | 0.55 |
| CHEMBL505504 | OC[C@H]1O[C@@H](OC(=O)[C@@]23CC[C@@]4([C@H](C2=CC[C@H]2[C@@]3(C)CC[C@@H]3[C@]2(C)CC[C@@H](C3(C)C)O[C@@H]2O[C@H](C)[C@H]([C@@H]([C@H]2O)O)O)[C@@H](C)[C@@H](CC4)C)C(=O)O)[C@@H]([C@H]([C@@H]1O)O)O | 794.97 | 7 | 14 | 8 | 201.88 | 232.9 | 1.95 | No | No | No | No | No | 3 | 3 | 1 | 1 | 4 | 0.11 |
| CHEMBL465969 | COc1ccc(cc1)[C@]1(O)[C@H](O)C(=Nc2c1cccc2)O | 285.29 | 2 | 5 | 3 | 81.39 | 82.28 | 1.67 | No | No | No | No | No | 0 | 0 | 0 | 0 | 0 | 0.55 |
| CHEMBL2047326 | COc1cc(OC(=O)C)c2c(c1OC(=O)C)C(=O)c1c(C2=O)cc(c(c1)C)OC(=O)C | 426.37 | 7 | 9 | 0 | 105.7 | 122.27 | 2.74 | Yes | No | Yes | No | Yes | 0 | 0 | 0 | 0 | 0 | 0.55 |
| CHEMBL57394 | Nc1cccc2c1cccc2 | 143.19 | 0 | 0 | 1 | 48.35 | 26.02 | 2.23 | Yes | No | No | No | No | 0 | 1 | 0 | 0 | 2 | 0.55 |
| CHEMBL552708 | CC(=O)OCC1=C[C@H]2[C@H]3[C@H](C3(C)C)C[C@H]([C@]3([C@@]([C@@H]1O)(O)[C@@H](OC(=O)c1ccccc1N=C(c1ccccc1N)O)C(=C3)C)C2=O)C | 628.71 | 8 | 9 | 4 | 172.26 | 168.74 | 3.69 | No | No | No | No | Yes | 1 | 3 | 1 | 1 | 2 | 0.55 |
| CHEMBL504937 | Oc1cc(O)c2c(c1)O[C@@H]([C@@H]([C@H]2c1c(O)cc(c2c1O[C@@H]([C@H](C2)O)c1ccc(c(c1)O)O)O)O)c1ccc(c(c1)O)O | 578.52 | 3 | 12 | 10 | 146.71 | 220.76 | 1.37 | No | No | No | No | Yes | 3 | 2 | 1 | 1 | 3 | 0.17 |
| CHEMBL498039 | CC1=CCCC(=CCC/C(=C/[C@H]2[C@@H](CC1)C(=C)C(=O)O2)/C)C(=O)O | 330.42 | 1 | 4 | 1 | 95.19 | 63.6 | 3.27 | No | Yes | Yes | No | No | 0 | 0 | 0 | 0 | 0 | 0.85 |
| CHEMBL1782230 | COC/C=C/1\CN2[C@@H]3[C@@H]4[C@H]1C[C@H]2[C@]1(C3)C(=Nc2c1c(O[C@@H]1O[C@H](CO)[C@H]([C@@H]([C@H]1O)O)O)c1c(c2)OCO1)OC4 | 544.55 | 5 | 12 | 4 | 139.66 | 151.9 | 0.23 | No | No | No | No | No | 2 | 4 | 1 | 1 | 3 | 0.17 |
| CHEMBL1289337 | O[C@H]1[C@@H](O[C@H]([C@@H]([C@H]1O)O)C)Oc1cc(O)c2c(c1)oc(c(c2=O)O)c1ccc(cc1)O | 432.38 | 3 | 10 | 6 | 106.97 | 170.05 | 0.61 | No | No | No | No | No | 1 | 0 | 1 | 1 | 2 | 0.55 |
| CHEMBL490988 | O=C1CC[C@@H]2[C@]([C@H]1C)(C)[C@H](O)C[C@H]1[C@@]2(C)CC[C@@]2([C@]1(C)CC(=O)[C@@]1([C@H]2CC(C)(C)C(=O)C1)C)C | 470.68 | 0 | 4 | 1 | 135.96 | 71.44 | 4.81 | No | No | No | No | No | 0 | 3 | 0 | 0 | 0 | 0.55 |
| CHEMBL1224597 | CC(=N[C@H](C(=O)O)CSc1c2nc3cc(ccc3oc2cc(=O)c1N)C(=O)O)O | 417.39 | 6 | 9 | 4 | 106.24 | 201.61 | 1.11 | No | No | No | No | No | 0 | 0 | 1 | 1 | 1 | 0.11 |
| CHEMBL498455 | C[C@H]1CC=C[C@H]2[C@H](O)C(=C([C@@H]3[C@@]2(OC(=O)C=C[C@@](C1)(C)O)C(=N[C@H]3Cc1ccccc1)O)C)C | 465.58 | 2 | 6 | 3 | 136.86 | 99.35 | 3.5 | No | No | No | No | Yes | 0 | 1 | 0 | 0 | 0 | 0.55 |
| CHEMBL2022669 | OCCCc1ccc(cc1)OCC=C(C)C | 220.31 | 6 | 2 | 1 | 67.43 | 29.46 | 3.15 | Yes | No | No | Yes | No | 0 | 0 | 0 | 0 | 0 | 0.55 |
| CHEMBL2165236 | CC(=CCc1c(O)cc2c(c1O)C(=O)C[C@H](O2)c1ccccc1)C | 324.37 | 3 | 4 | 2 | 93.27 | 66.76 | 3.73 | Yes | Yes | Yes | Yes | Yes | 0 | 0 | 0 | 0 | 0 | 0.55 |
| CHEMBL478710 | CCCC(=O)c1c(O)c2c(cc(=O)oc2c2c1O[C@@H](C2)C(O)(C)C)c1ccccc1 | 408.44 | 5 | 6 | 2 | 114.72 | 96.97 | 3.75 | No | No | Yes | No | Yes | 0 | 0 | 0 | 0 | 0 | 0.55 |
| CHEMBL3104877 | Oc1cc2c(c(c1C)O)C(=O)[C@@]1([C@@](C2=O)(C[C@@H]2[C@@](C)(O)CC[C@@H](C2(C)C)Cl)OC([C@@H](C1)Cl)(C)C)Cl | 547.9 | 2 | 6 | 3 | 138.13 | 104.06 | 4.67 | No | No | No | No | Yes | 1 | 2 | 0 | 0 | 1 | 0.55 |
| CHEMBL517338 | O=C1C=C(O)C(=O)C(=C1)C[C@]1(C)[C@@H](C)CC[C@]2([C@H]1CCC=C2C)C | 328.45 | 2 | 3 | 1 | 96.75 | 54.37 | 3.91 | No | Yes | Yes | No | Yes | 0 | 0 | 0 | 0 | 0 | 0.85 |
| CHEMBL330320 | COc1c(OC)cc2c(c1OC)c1ccc(c(=O)cc1[C@@H](CC2)N=C(O)C)OC | 399.44 | 5 | 7 | 1 | 111.61 | 86.58 | 2.91 | No | No | No | No | Yes | 0 | 0 | 0 | 0 | 0 | 0.55 |
| CHEMBL2088425 | NCCC[C@@H](CC(=NCCC[C@@H](CC(=NCCC[C@@H](CC(=N[C@H]1[C@H](NC2=N[C@H]3[C@H](N2)C(=NC[C@H]3O)O)O[C@@H]([C@@H]([C@H]1O)O)COC(=N)O)O)N=C(O)C)O)N)O)N | 800.9 | 24 | 21 | 15 | 219.48 | 400.66 | -1.87 | No | No | No | No | No | 3 | 4 | 2 | 1 | 6 | 0.17 |
| CHEMBL129992 | C[C@H]1Oc2c3c(O[C@@H]4[C@H]3C4(C)C)c3c(c2[C@H]([C@H]1C)O)oc(=O)cc3c1ccccc1 | 404.46 | 1 | 5 | 1 | 114.24 | 68.9 | 4.11 | No | Yes | No | Yes | No | 0 | 0 | 0 | 0 | 0 | 0.55 |
| CHEMBL400327 | COc1cc(OC)c2c(c1)C(=O)C1=C(C2=O)COC(=C1)C | 286.28 | 2 | 5 | 0 | 74.95 | 61.83 | 2.05 | Yes | Yes | Yes | No | Yes | 0 | 0 | 0 | 0 | 0 | 0.55 |
| CHEMBL3247426 | CCCCC(=O)C(=O)O | 130.14 | 4 | 3 | 1 | 32.93 | 54.37 | 0.74 | No | No | No | No | No | 0 | 3 | 0 | 0 | 1 | 0.85 |
| CHEMBL53493 | C/C=C(/C=O)\C | 84.12 | 1 | 1 | 0 | 25.88 | 17.07 | 1.05 | No | No | No | No | No | 0 | 3 | 0 | 0 | 2 | 0.55 |
| CHEMBL2335697 | CC(=O)O[C@@H]1[C@H]2[C@@H](OC(=O)c3cccnc3)[C@H]([C@]3([C@@]1(OC2(C)C)[C@@](C)(O)CC[C@@H]3OC(=O)c1ccoc1)COC(=O)C)OC(=O)c1ccoc1 | 695.67 | 14 | 15 | 1 | 166.5 | 200.13 | 2.77 | No | Yes | No | No | Yes | 2 | 3 | 2 | 1 | 3 | 0.17 |
| CHEMBL516668 | C=C1[C@@H]2CC[C@@H]3[C@](C1=O)(C2)C(=O)[C@@H](O)[C@H]1[C@@]23CO[C@@H](C1(C)C)CC2=O | 344.4 | 0 | 5 | 1 | 89.28 | 80.67 | 1.7 | No | No | No | No | No | 0 | 0 | 0 | 0 | 0 | 0.55 |
| CHEMBL1651277 | COc1c(O)cc(cc1O)[C@H]1Oc2c(C[C@@H]1O)c(O)cc1c2[C@H](CC(=O)O1)c1ccc(c(c1)OC)O | 496.46 | 4 | 10 | 5 | 126.16 | 155.14 | 2.21 | No | No | No | No | Yes | 0 | 1 | 1 | 1 | 1 | 0.55 |
| CHEMBL504757 | COc1c(OC)cc2c3c1Oc1cc4c(cc1OC)CCN([C@@H]4Cc1ccc(Oc4cc(C[C@@H]3N(CC2)C)ccc4OC)cc1)C | 622.75 | 4 | 8 | 0 | 186.07 | 61.86 | 5.47 | No | No | No | No | No | 1 | 4 | 0 | 0 | 2 | 0.55 |
| CHEMBL116438 | COc1cc(/C=C/C(=C/C(=O)/C=C/c2ccc(c(c2)OC)O)/O)ccc1O | 368.38 | 7 | 6 | 3 | 103.7 | 96.22 | 3.17 | No | No | Yes | No | Yes | 0 | 0 | 0 | 0 | 0 | 0.56 |
| CHEMBL2431351 | CC(CCC(=O)c1c(O)cc(cc1O)CC(=O)c1ccccc1)C | 326.39 | 7 | 4 | 2 | 94.59 | 74.6 | 3.71 | Yes | Yes | Yes | Yes | Yes | 0 | 0 | 0 | 0 | 0 | 0.55 |
| CHEMBL1330613 | OC(=N)NCC(C(=O)O)N | 147.13 | 4 | 5 | 5 | 33.79 | 119.43 | -2.29 | No | No | No | No | No | 0 | 4 | 0 | 0 | 3 | 0.55 |
| CHEMBL136933 | CN([C@H]([C@H]1CC[C@@H]2[C@]1(C)CC[C@H]1[C@H]2CC[C@@H]2[C@]1(C)CC[C@@H](C2)N=C(c1ccccc1)O)C)C | 450.7 | 4 | 3 | 1 | 141.15 | 35.83 | 6.26 | No | No | No | No | No | 1 | 3 | 0 | 1 | 1 | 0.55 |
| CHEMBL598918 | Brc1c(Br)cc(n1C)C(=O)O | 282.92 | 1 | 2 | 1 | 48.05 | 42.23 | 1.81 | Yes | No | No | No | No | 0 | 1 | 0 | 0 | 0 | 0.85 |
| CHEMBL267742 | Oc1ccc2c(c1)OC[C@@H]1[C@H]2Oc2c1ccc1c2cc(o1)C(=O)C | 322.31 | 1 | 5 | 1 | 86.64 | 68.9 | 2.89 | Yes | Yes | No | Yes | Yes | 0 | 0 | 0 | 0 | 0 | 0.55 |
| CHEMBL1835967 | COc1cc(O)c2c(c1Cc1ccccc1O)O[C@@H](CC2=O)c1ccccc1 | 376.4 | 4 | 5 | 2 | 105.49 | 75.99 | 3.76 | No | Yes | Yes | Yes | Yes | 0 | 0 | 0 | 0 | 0 | 0.55 |
| CHEMBL515914 | CC(=O)O[C@@H]1[C@H](OC(=O)C)[C@H](O[C@H]1n1ncc(nc1=O)O)COC(=O)C | 371.3 | 8 | 11 | 1 | 80.47 | 156.14 | -0.45 | No | No | No | No | No | 1 | 1 | 1 | 1 | 2 | 0.55 |
| CHEMBL1813866 | C/C=C(/C(=O)O[C@@H]1CC[C@@]2([C@]3([C@H]1[C@@](C)(C[C@@H](C1=CC(=O)OC1)O)[C@H](C)C[C@@H]3OC(=O)C)COC(=O)C)CO2)\C | 548.62 | 11 | 10 | 1 | 139.02 | 137.96 | 2.95 | No | No | No | No | No | 1 | 3 | 1 | 1 | 0 | 0.55 |
| CHEMBL2431352 | COc1ccc(cc1)C(=O)Cc1cc(O)c(c(c1)O)C(=O)CCC(C)C | 356.41 | 8 | 5 | 2 | 101.08 | 83.83 | 3.6 | Yes | No | Yes | Yes | Yes | 0 | 0 | 0 | 0 | 0 | 0.55 |
| CHEMBL571616 | COc1cc(C=O)cc2c1oc(c2C)c1ccc2c(c1)OCO2 | 310.3 | 3 | 5 | 0 | 84.56 | 57.9 | 3.36 | Yes | Yes | Yes | No | Yes | 0 | 0 | 0 | 0 | 0 | 0.55 |
| CHEMBL2207726 | COC1(OC)COc2cc(O)ccc2c2c(C1)cc(O)c(c2)O | 318.32 | 2 | 6 | 3 | 83.92 | 88.38 | 1.93 | Yes | No | No | No | Yes | 0 | 0 | 0 | 0 | 0 | 0.55 |
| CHEMBL370087 | COc1ccc(cc1)C(C(=O)O)C | 180.2 | 3 | 3 | 1 | 49.29 | 46.53 | 1.74 | No | No | No | No | No | 0 | 0 | 0 | 0 | 1 | 0.85 |
| CHEMBL1256380 | Oc1cc(O)c(c(c1)O)C(=O)c1cc(O)cc(c1)O | 262.21 | 2 | 6 | 5 | 66.43 | 118.22 | 1.03 | Yes | No | Yes | No | Yes | 0 | 0 | 0 | 0 | 0 | 0.55 |
| CHEMBL3580736 | CCOC(=O)/C=C/c1cc(OC)c(c(c1)OC)O | 252.26 | 6 | 5 | 1 | 67.25 | 64.99 | 2.08 | Yes | No | No | No | No | 0 | 0 | 0 | 0 | 0 | 0.55 |
| CHEMBL233040 | C/C=C\1/C(=O)C[C@@H]2[C@]1(C)CC[C@H]1[C@H]2CC=C2[C@]1(C)CC[C@@H]([C@@H]2O)O | 330.46 | 0 | 3 | 2 | 95.66 | 57.53 | 3.12 | No | No | No | No | No | 0 | 0 | 0 | 0 | 0 | 0.55 |
| CHEMBL110370 | COc1cc(/C=C/c2cc(O)cc(c2)O)ccc1O | 258.27 | 3 | 4 | 3 | 74.37 | 69.92 | 2.63 | Yes | No | Yes | No | Yes | 0 | 0 | 0 | 0 | 0 | 0.55 |
| CHEMBL699 | OCCn1cnc2c1c(=O)n(C)c(=O)n2C | 224.22 | 2 | 4 | 1 | 58.01 | 82.05 | -0.42 | No | No | No | No | No | 0 | 1 | 0 | 0 | 0 | 0.55 |
| CHEMBL482585 | COCc1c(Cc2cc(O)c(c(c2Br)Br)O)c(O)c(c(c1Br)Br)O | 591.87 | 4 | 5 | 4 | 105.64 | 90.15 | 4.31 | No | No | Yes | Yes | No | 1 | 1 | 0 | 0 | 0 | 0.55 |
| CHEMBL457680 | CC(=CCc1cc(ccc1O)C1CC(=O)c2c(O1)cc(cc2O)O)C | 340.37 | 3 | 5 | 3 | 95.29 | 86.99 | 3.33 | Yes | No | Yes | Yes | Yes | 0 | 0 | 0 | 0 | 0 | 0.55 |
| CHEMBL1824574 | O[C@@H](C[C@H](OC(=O)C)CCc1ccc(c(c1)O)O)CCc1ccc(c(c1)O)O | 390.43 | 10 | 7 | 5 | 104.89 | 127.45 | 2.6 | No | No | No | Yes | No | 0 | 0 | 0 | 0 | 0 | 0.55 |
| CHEMBL1651112 | CCCCC/C=C/C=CC(=O)O[C@H]1C=C2[C@@H](OC(=O)C)O[C@H]([C@]32[C@@H](C1)[C@](C)(C/C=C(/C=C)\C)[C@@H](C[C@H]3O)C)OC(=O)C | 584.74 | 15 | 8 | 1 | 162.42 | 108.36 | 5.85 | No | No | No | No | Yes | 1 | 4 | 1 | 1 | 1 | 0.55 |
| CHEMBL178 | COc1cccc2c1C(=O)c1c(C2=O)c(O)c2c(c1O)[C@@H](O[C@H]1C[C@H](N)[C@@H]([C@@H](O1)C)O)C[C@](C2)(O)C(=O)C | 527.52 | 4 | 11 | 5 | 131.5 | 185.84 | 1.18 | No | No | No | No | No | 2 | 2 | 1 | 1 | 2 | 0.17 |
| CHEMBL462737 | O=C(C(C)C)O[C@@H]1C[C@H](C[C@]21C(=CC(=O)[C@@H]([C@H]2C)O)C)C(=C)C | 320.42 | 4 | 4 | 1 | 90.66 | 63.6 | 3.08 | No | No | No | No | Yes | 0 | 0 | 0 | 0 | 0 | 0.55 |
| CHEMBL511199 | CN[C@H](C(=NCCc1c(Br)[nH]c2c1cc(Br)cc2)O)CC(C)C | 445.19 | 7 | 3 | 3 | 105.18 | 60.41 | 4.38 | Yes | Yes | Yes | No | Yes | 0 | 0 | 0 | 0 | 1 | 0.55 |
| CHEMBL2074802 | OC(=O)[C@H]1O[C@@H](O[C@@H]2C[C@@H]3[C@]([C@H]2O)(C)CC[C@H]2[C@H]3CCc3c2ccc(c3)O)[C@@H]([C@H]([C@@H]1O)O)O | 464.51 | 3 | 9 | 6 | 115.18 | 156.91 | 0.66 | No | No | No | No | No | 1 | 0 | 1 | 1 | 2 | 0.11 |
| CHEMBL3391893 | O[C@@H]1C[C@]2(C)[C@@H]([C@@]3([C@@H]1C(C)(C)[C@@H](O)CC3)C)C[C@H]([C@H]1[C@@]2(C)CC[C@@H]1[C@@]1(C)CCCC(O1)(C)C)O | 476.73 | 1 | 4 | 3 | 139.36 | 69.92 | 5.02 | No | No | No | No | No | 1 | 3 | 0 | 0 | 1 | 0.55 |
| CHEMBL517713 | O=C1O[C@@H](CC=CC=CC[C@@H]([C@@H]([C@H](C(=O)C([C@H](C1)O)(C)C)C)O)C)/C(=C/c1csc(n1)C)/C | 475.64 | 2 | 6 | 2 | 133.8 | 124.96 | 4 | No | No | No | No | Yes | 0 | 1 | 0 | 0 | 0 | 0.55 |
| CHEMBL464481 | CC(=O)O[C@@H]1C[C@@H](C)[C@@]2([C@@]3([C@@H]1C(C)(C)CCC3)C)CCC(=O)O2 | 322.44 | 2 | 4 | 0 | 89.19 | 52.6 | 3.65 | No | No | Yes | No | No | 0 | 0 | 0 | 0 | 0 | 0.55 |
| CHEMBL454687 | C[C@H]1CC[C@H](OC1=O)[C@H]([C@H]1CC[C@@]2([C@]1(C)CCC1=C2CC[C@@H]2[C@]1(C)CCC(=O)C2(C)C)C)C | 454.68 | 2 | 3 | 0 | 135.73 | 43.37 | 6.29 | No | No | No | No | No | 1 | 3 | 0 | 1 | 1 | 0.55 |
| CHEMBL485479 | CC(C1=Cc2ccc3c(c2C(=O)C1=O)C(=O)CCC3(C)C)C | 296.36 | 1 | 3 | 0 | 86.58 | 51.21 | 3.28 | Yes | Yes | Yes | No | No | 0 | 0 | 0 | 0 | 0 | 0.55 |
| CHEMBL560841 | C/C=C/[C@H]1C(=C[C@@H]2[C@H]([C@]1(C)/C=C(/C=C/C(=O)O)\C)C[C@H]([C@H](C2)C)O)C | 344.49 | 4 | 3 | 2 | 104.42 | 57.53 | 4.15 | No | No | Yes | No | Yes | 0 | 0 | 0 | 0 | 0 | 0.85 |
| CHEMBL2087212 | O[C@H]1CC[C@]2([C@H]([C@@H]1O)CC[C@@H]1[C@@H]2CC[C@]2([C@H]1CC[C@@H]2[C@@H](N(C)C)C)C)C | 363.58 | 2 | 3 | 2 | 108.92 | 43.7 | 3.87 | No | No | No | No | No | 0 | 0 | 0 | 0 | 0 | 0.55 |
| CHEMBL497207 | CC(CCC[C@H]([C@H]1CC[C@@H]2[C@]1(C)CC[C@H]1[C@H]2[C@H](O)C=C2[C@]1(C)CC[C@@H](C2)O)C)C | 402.65 | 5 | 2 | 2 | 124.78 | 40.46 | 5.89 | No | No | No | No | No | 1 | 2 | 0 | 1 | 1 | 0.55 |
| CHEMBL2419335 | CC1=C[C@@]23O[C@]3(CC(C2=O)(C)C)[C@@H](CC[C@@H]1O)C | 250.33 | 0 | 3 | 1 | 69.67 | 49.83 | 2.19 | No | No | No | No | No | 0 | 0 | 0 | 0 | 0 | 0.55 |
| CHEMBL496046 | CC(=CC(=O)O)CC[C@]1(C)[C@@H](C)CC[C@]2([C@H]1CC(=O)C=C2C)C | 318.45 | 4 | 3 | 1 | 94.53 | 54.37 | 4.04 | No | Yes | Yes | No | Yes | 0 | 0 | 0 | 0 | 0 | 0.85 |
| CHEMBL1076002 | Oc1ccc(cc1)C[C@@H]1N(C)C(=O)[C@@](C1=O)(C)O | 249.26 | 2 | 4 | 2 | 68.57 | 77.84 | 0.57 | No | No | No | No | No | 0 | 0 | 0 | 0 | 0 | 0.55 |
| CHEMBL2437365 | OC[C@H]1O[C@H]([C@@H]([C@H]([C@@H]1O)O)O)c1c(O)c([C@@H]2OC[C@@H]([C@@H]([C@H]2O)O)O)c(c2c1oc(cc2=O)c1ccc(cc1)O)O | 564.49 | 4 | 14 | 10 | 133.26 | 250.97 | -1.64 | No | No | No | No | No | 3 | 3 | 1 | 1 | 4 | 0.17 |
| CHEMBL2006033 | C/C=C(/C(=O)OC1C2OC(=O)C(C3C42COC(C4C2(C1C(=CC(=O)C2O)C)C)(C(C3C)O)O)OC(=O)C)\C | 534.55 | 5 | 11 | 3 | 128.53 | 165.89 | 0.69 | No | No | No | No | No | 2 | 2 | 1 | 1 | 2 | 0.17 |
| CHEMBL1098658 | COc1ccc(cc1)/C=C/c1cc(OC)cc(=O)o1 | 258.27 | 4 | 4 | 0 | 73.33 | 48.67 | 2.72 | Yes | Yes | Yes | No | No | 0 | 0 | 0 | 0 | 0 | 0.55 |
| CHEMBL3426666 | COc1cc(OC)c2c(c1C=CC(=C)C)oc(=O)cc2 | 272.3 | 4 | 4 | 0 | 79.51 | 48.67 | 3.35 | Yes | Yes | Yes | No | No | 0 | 0 | 0 | 0 | 0 | 0.55 |
| CHEMBL455349 | Oc1cc(CCCCCCC=CCCCCCCc2cc(O)cc(c2)O)cc(c1)O | 412.56 | 14 | 4 | 4 | 126 | 80.92 | 6.09 | Yes | Yes | No | Yes | No | 1 | 1 | 1 | 1 | 1 | 0.55 |
| CHEMBL2003972 | CCCCCCCCCc1cc(=O)c2c(o1)cc(cc2O)O | 304.38 | 8 | 4 | 2 | 89.95 | 70.67 | 4.29 | Yes | Yes | Yes | Yes | No | 0 | 0 | 0 | 0 | 1 | 0.55 |
| CHEMBL463509 | OC[C@H]([C@@H](/C=C/[C@H]([C@H]1C[C@H]([C@@H]2[C@]1(C)CC[C@H]1[C@@]2(O)C[C@@H]([C@@H]2[C@]1(C)CC[C@@H]([C@@H]2O)O)O)O)C)C)C | 480.68 | 5 | 6 | 6 | 134.27 | 121.38 | 2.72 | No | No | No | No | No | 1 | 3 | 0 | 0 | 1 | 0.55 |
| CHEMBL486801 | COc1cccc2c1ccc1c2c2OCOc2cc1C(=O)O | 296.27 | 2 | 5 | 1 | 80.97 | 64.99 | 3.02 | Yes | Yes | Yes | No | No | 0 | 0 | 0 | 0 | 0 | 0.85 |
| CHEMBL61004 | OC(=O)c1ccc2c(c1)CCC(O2)(C)C | 206.24 | 1 | 3 | 1 | 57.2 | 46.53 | 2.35 | Yes | No | No | No | No | 0 | 0 | 0 | 0 | 0 | 0.85 |
| CHEMBL2269099 | CC(=C[C@@H]1c2cccc3c2[C@]24CCN1[C@H]1N(CC[C@@]21c1ccccc1N[C@@H]4N3C)C(=O)C)C | 440.58 | 2 | 2 | 1 | 145.66 | 38.82 | 3.59 | No | No | No | No | Yes | 1 | 1 | 0 | 0 | 0 | 0.55 |
| CHEMBL2144305 | OC[C@H]1O[C@@H](O[C@@H]2[C@@H](O)[C@@H](O[C@@H]([C@@H]2O)C)O[C@H]2CC[C@]3(C([C@]2(C)CO)CC[C@@]2(C3C=CC3=C4CC(C)(C)CC[C@@]4([C@@H](C[C@@]23C)O)CO)C)C)[C@@H]([C@H]([C@@H]1O)O)O | 780.98 | 7 | 13 | 9 | 201.5 | 218.99 | 1.86 | No | No | No | No | No | 3 | 3 | 1 | 1 | 4 | 0.17 |
| CHEMBL456951 | CC(=CC(=O)C=C(C)C)CCC[C@]1(C)Oc2c([C@H]1C)c(=O)c1c(o2)cc(cc1)O | 396.48 | 6 | 5 | 1 | 115.63 | 76.74 | 4.6 | No | Yes | Yes | No | Yes | 0 | 0 | 0 | 0 | 1 | 0.55 |
| CHEMBL445740 | CC1=CCCC(=C)[C@@H]2[C@@H](CC1)C(C2)(C)C | 204.35 | 0 | 0 | 0 | 68.78 | 0 | 4.24 | No | Yes | Yes | No | No | 1 | 0 | 0 | 0 | 1 | 0.55 |
| CHEMBL464725 | C/C=C/c1ccc(c(c1)OC)O[C@H]([C@H](c1ccc(c(c1)OC)OC)O)C | 358.43 | 8 | 5 | 1 | 102.57 | 57.15 | 3.76 | Yes | No | No | Yes | Yes | 0 | 0 | 0 | 0 | 0 | 0.55 |
| CHEMBL516835 | COc1cc(O)c(c(c1)C(=O)OC)OC(=O)c1c(O)cc2c(c1O)c(=O)oc(c2)C | 416.34 | 6 | 10 | 3 | 102.69 | 152.73 | 2.43 | Yes | No | Yes | No | Yes | 0 | 0 | 1 | 1 | 1 | 0.55 |
| CHEMBL3085398 | C=CC(c1cc([C@@H]2CC(=O)c3c(O2)c(CC=C(C)C)c(cc3O)O)c(cc1O)O)(C)C | 424.49 | 5 | 6 | 4 | 120.92 | 107.22 | 4.35 | No | No | Yes | No | Yes | 0 | 0 | 0 | 0 | 1 | 0.55 |
| CHEMBL445274 | O=C1CC[C@]2(C=C1)C(=C)C[C@H]([C@H](C2(C)C)Br)O | 299.2 | 0 | 2 | 1 | 72.95 | 37.3 | 2.72 | No | No | Yes | No | No | 0 | 0 | 0 | 0 | 0 | 0.55 |
| CHEMBL491882 | CC(=O)OCc1nccc2c1C(=O)C(=C(C2=O)C)O | 261.23 | 3 | 6 | 1 | 64.28 | 93.56 | 0.83 | Yes | No | No | No | No | 0 | 0 | 0 | 0 | 0 | 0.56 |
| CHEMBL486004 | CC[C@H]1CN2CCc3c([C@@H]2C[C@@H]1C[C@@]1(O)NCCc2c1cc(OC)c(c2)O)cc(c(c3)O)OC | 468.59 | 5 | 7 | 4 | 139.16 | 94.42 | 3.15 | No | No | No | Yes | No | 0 | 1 | 0 | 0 | 0 | 0.55 |
| CHEMBL455764 | CO[C@@H]1O[C@@H]2[C@H]([C@@H]1C)[C@]1(C)[C@@H](C)C[C@@H](C=C1CC2)O | 266.38 | 1 | 3 | 1 | 75.28 | 38.69 | 2.56 | No | No | No | No | No | 0 | 0 | 0 | 0 | 0 | 0.55 |
| CHEMBL111134 | C=CCc1ccc(c(c1)O)O | 150.17 | 2 | 2 | 2 | 44.59 | 40.46 | 1.53 | No | No | No | No | No | 0 | 1 | 0 | 0 | 1 | 0.55 |
| CHEMBL478084 | CC(CC(=O)c1c(O)c2C[C@H](Oc2c2c1oc(=O)cc2c1ccccc1)C(O)(C)C)C | 422.47 | 5 | 6 | 2 | 119.52 | 96.97 | 3.94 | No | No | Yes | No | Yes | 0 | 0 | 0 | 0 | 0 | 0.55 |
| CHEMBL464284 | O/N=C(/C(=NCCc1cc(Br)c(c(c1)Br)OCCCN(=O)(C)C)O)\Cc1ccc(c(c1)Br)OC | 666.2 | 12 | 8 | 2 | 146.37 | 113.07 | 4.78 | No | Yes | No | No | No | 1 | 3 | 1 | 1 | 2 | 0.55 |
| CHEMBL494054 | O[C@H]1C[C@@]2(C)[C@@H](C[C@H]1O)C(=O)C=C1[C@@H]2CC[C@]2([C@@]1(O)CC[C@@H]2[C@](C(=O)C[C@@H]1[C@@H](C)OC(=O)[C@H]1C)(O)C)C | 518.64 | 4 | 8 | 4 | 136.36 | 141.36 | 1.97 | No | No | No | No | No | 1 | 3 | 1 | 1 | 0 | 0.55 |
| CHEMBL464100 | O=C(/C=C/c1ccc(c(c1)O)O)OC[C@H]1O[C@@H](O[C@@H]2OC=C([C@@H]3[C@H]2[C@@](C)(O)CC3)C(=O)O)[C@@H]([C@H]([C@@H]1O)O)OC(=O)c1ccc(cc1)O | 658.6 | 11 | 15 | 7 | 158.14 | 238.97 | 0.88 | No | No | No | No | No | 3 | 3 | 2 | 1 | 4 | 0.11 |
| CHEMBL1099251 | CC1=C2C[C@]3(C)[C@@H](C)CC[C@H]([C@]3(C[C@H]2OC1=O)O)O | 266.33 | 0 | 4 | 2 | 70.79 | 66.76 | 1.75 | No | No | No | No | No | 0 | 0 | 0 | 0 | 0 | 0.55 |
| CHEMBL53343 | COc1c(C)c2O[C@@H](CC(=O)c2c(c1C)O)c1ccccc1 | 298.33 | 2 | 4 | 1 | 83.95 | 55.76 | 3.27 | Yes | Yes | Yes | Yes | Yes | 0 | 0 | 0 | 0 | 0 | 0.55 |
| CHEMBL1077015 | OC[C@H]1O[C@@H](OC/C=C/c2ccc(c(c2)O)OC)[C@@H]([C@H]([C@@H]1O)O[C@@H]1OC[C@]([C@H]1O)(O)COC(=O)/C=C/c1ccccc1)O | 604.6 | 13 | 13 | 6 | 149.2 | 193.83 | 0.7 | No | No | No | No | No | 3 | 3 | 2 | 1 | 4 | 0.17 |
| CHEMBL1568181 | COc1c(OC)c(O)cc2c1oc(=O)cc2c1ccccc1 | 298.29 | 3 | 5 | 1 | 82.93 | 68.9 | 2.8 | Yes | No | Yes | Yes | Yes | 0 | 0 | 0 | 0 | 0 | 0.55 |
| CHEMBL521934 | COc1ccc(cc1)c1oc2cc(OC)c(c(c2c(=O)c1OC)O)OC | 358.34 | 5 | 7 | 1 | 95.91 | 87.36 | 2.83 | Yes | No | Yes | Yes | Yes | 0 | 0 | 0 | 0 | 0 | 0.55 |
| CHEMBL517189 | OC(=O)CC[C@@]1(C)[C@@H](CC[C@@]2([C@@H]1CC=C1[C@@]2(C)CC[C@@]2([C@H]1C[C@@](C)(CC2)C(=O)O)C)C)C(=C)C | 470.68 | 5 | 4 | 2 | 139.16 | 74.6 | 6.06 | No | No | Yes | No | No | 1 | 3 | 0 | 1 | 1 | 0.85 |
| CHEMBL44857 | CCCOC(=O)C | 102.13 | 3 | 2 | 0 | 27.43 | 26.3 | 1.14 | No | No | No | No | No | 0 | 3 | 0 | 0 | 1 | 0.55 |
| CHEMBL254348 | CN(Cc1c[nH]c2c1cccc2)C | 174.24 | 2 | 1 | 1 | 55.77 | 19.03 | 1.94 | Yes | No | No | No | No | 0 | 0 | 0 | 0 | 1 | 0.55 |
| CHEMBL2002878 | C[C@@H]1N=C(O)c2csc(n2)[C@H](N=C(O)c2nc(CN=C(c3nc1oc3C)O)sc2)C(C)C | 474.56 | 1 | 10 | 3 | 134.13 | 206.06 | 2.77 | No | No | No | No | No | 0 | 1 | 1 | 1 | 1 | 0.55 |
| CHEMBL380697 | CN(CCCOc1c(Br)cc(cc1Br)CCN(C)C)C | 408.17 | 8 | 3 | 0 | 92.74 | 15.71 | 3.83 | Yes | No | No | Yes | No | 0 | 0 | 0 | 0 | 0 | 0.55 |
| CHEMBL1458 | OC(=NC1CCCCC1)NC1CCCCC1 | 224.34 | 3 | 2 | 2 | 68.44 | 44.62 | 2.9 | No | No | No | No | No | 0 | 0 | 0 | 0 | 0 | 0.55 |
| CHEMBL1956941 | OC[C@H]1O[C@@H](OC(=O)[C@@]23CC[C@H]([C@@H]([C@H]3C3=CC[C@H]4[C@@]([C@@]3(CC2)C)(C)CC[C@@H]2[C@]4(C)C[C@H]([C@H]([C@@]2(C)CO)O)O)C)C)[C@@H]([C@H]([C@@H]1O)O)O | 650.84 | 5 | 10 | 7 | 171.21 | 177.14 | 2.83 | No | No | No | No | No | 2 | 3 | 1 | 1 | 3 | 0.17 |
| CHEMBL484847 | CC(=O)[C@H]1[C@H](O)C[C@@]2([C@]1(C)CC(=O)[C@@]1([C@H]2CC=C2[C@H]1C[C@H](O)[C@H](C2(C)C)O)C)C | 404.54 | 1 | 5 | 3 | 111.4 | 94.83 | 2.31 | No | No | No | No | No | 0 | 0 | 0 | 0 | 0 | 0.55 |
| CHEMBL304564 | CC([C@@H]1CCC(=O)C2=C1[C@H](O)[C@](CC2)(C)O)C | 238.32 | 1 | 3 | 2 | 67.27 | 57.53 | 1.7 | No | No | No | No | No | 0 | 0 | 0 | 0 | 0 | 0.55 |
| CHEMBL3126956 | OC[C@]1(O)CC[C@H]([C@@H]1c1nccc2c1[nH]c1c2c(OC)ccc1)C(O)(C)C | 370.44 | 4 | 5 | 4 | 104.96 | 98.6 | 2.23 | No | No | No | Yes | No | 0 | 0 | 0 | 0 | 0 | 0.55 |
| CHEMBL3358229 | O=Cc1c[nH]c2c1ccc(c2)Br | 224.05 | 1 | 1 | 1 | 51.39 | 32.86 | 2.31 | Yes | No | No | No | No | 0 | 1 | 0 | 0 | 0 | 0.55 |
| CHEMBL524719 | Cc1cc2c3ccccc3n3c2c2c1O[C@@]1(C)CC[C@H]([C@@H]2C1)C3(C)C | 331.45 | 0 | 1 | 0 | 104.36 | 14.16 | 4.94 | No | No | No | Yes | No | 1 | 1 | 0 | 1 | 1 | 0.55 |
| CHEMBL1162275 | CNCCc1c[nH]c2c1c(=O)c1c(c[nH]c1c2=O)c1ccc(cc1)O | 335.36 | 4 | 4 | 4 | 98.72 | 97.98 | 2.14 | Yes | No | No | Yes | Yes | 0 | 0 | 0 | 0 | 0 | 0.55 |
| CHEMBL1094939 | CC1=CC[C@@H](OC(=O)c2ccc(cc2)O)C(=C[C@@H](C(CCC1)(C)C)O)C | 358.47 | 3 | 4 | 2 | 104.88 | 66.76 | 3.92 | No | No | No | No | Yes | 0 | 0 | 0 | 0 | 0 | 0.55 |
| CHEMBL521753 | C=C1CC[C@H](C([C@@]21CC[C@]([C@H](C2)Cl)(C)Br)(C)C)Br | 398.6 | 0 | 0 | 0 | 89.57 | 0 | 5.31 | No | Yes | Yes | No | No | 1 | 1 | 0 | 1 | 2 | 0.55 |
| CHEMBL537954 | COc1ccc(cc1)[C@@H]1CC(=O)c2c(O1)cc(cc2OC)O | 300.31 | 3 | 5 | 1 | 80.51 | 64.99 | 2.43 | Yes | Yes | No | Yes | Yes | 0 | 0 | 0 | 0 | 0 | 0.55 |
| CHEMBL1812584 | CCCCCCCCC=CCCCCCCC=O | 252.44 | 14 | 1 | 0 | 83.56 | 17.07 | 5.56 | Yes | No | No | No | No | 1 | 1 | 1 | 0 | 2 | 0.55 |
| CHEMBL480866 | OC/C(=C/C)/C1=C(C=C)C(C[C@]1(C)CO)(C)C | 236.35 | 4 | 2 | 2 | 72.49 | 40.46 | 2.82 | No | No | No | No | No | 0 | 0 | 0 | 0 | 0 | 0.55 |
| CHEMBL1094579 | O=C1C=CC(C=C1)(O)CC(=O)OC[C@H]1O[C@@H](OC(=O)CC2(O)C=CC(=O)C=C2)[C@@H]([C@H]([C@@H]1O)OC(=O)Cc1ccccc1)OC(=O)Cc1ccccc1 | 716.68 | 17 | 14 | 3 | 178.02 | 209.26 | 1.71 | No | No | No | No | No | 2 | 3 | 2 | 1 | 4 | 0.17 |
| CHEMBL524490 | Oc1ccc(c(c1)O)c1oc2c(c1)cc1c(c2)OCO1 | 270.24 | 1 | 5 | 2 | 71.76 | 72.06 | 2.6 | Yes | Yes | No | Yes | Yes | 0 | 0 | 0 | 0 | 0 | 0.55 |
| CHEMBL3248214 | N#Cc1ccc(c(c1)C)C | 131.17 | 0 | 1 | 0 | 41.09 | 23.79 | 2.26 | Yes | No | No | No | No | 0 | 2 | 0 | 0 | 2 | 0.55 |
| CHEMBL3093406 | C/C=C/C=C/C(=C\1/C(=O)C(=C([C@@]2([C@H]1[C@]1(C)C(=O)C(=C([C@]([C@@]1(O2)O)(C)O)N)C(=O)/C=C/C=C/C)C)O)C)/O | 511.56 | 5 | 8 | 5 | 136.76 | 167.38 | 2.15 | No | No | Yes | No | Yes | 1 | 2 | 1 | 1 | 1 | 0.11 |
| CHEMBL454077 | CC(=CCc1cc(cc2c1OC(C)(C)C=C2)[C@@H]1CC(=O)c2c(O1)cc(cc2)O)C | 390.47 | 3 | 4 | 1 | 115.37 | 55.76 | 4.69 | No | Yes | Yes | No | Yes | 0 | 0 | 0 | 0 | 1 | 0.55 |
| CHEMBL479311 | COC(=O)C(=C)[C@@H]1C[C@@H](OC(=O)C)C2=C[C@@H](OC2=O)C[C@@]2([C@@H](c3oc([C@H]1OC(=O)C)c(C)c3)O2)C | 488.48 | 7 | 10 | 0 | 119.11 | 130.87 | 2.26 | No | No | No | No | No | 0 | 1 | 0 | 0 | 0 | 0.55 |
| CHEMBL86075 | COc1cc(/C=C/C(=O)OCC(=O)/C=C/c2ccc(c(c2)OC)O)ccc1O | 384.38 | 9 | 7 | 2 | 103.89 | 102.29 | 2.88 | No | No | Yes | No | Yes | 0 | 0 | 0 | 0 | 0 | 0.55 |
| CHEMBL1414826 | COc1cc(cc(c1OC)OC)C(=S)N1CCOCC1 | 297.37 | 5 | 4 | 0 | 83.69 | 72.25 | 1.99 | No | Yes | No | No | No | 0 | 0 | 0 | 0 | 0 | 0.55 |
| CHEMBL458630 | OCC(C)(C)C | 88.15 | 1 | 1 | 1 | 27.05 | 20.23 | 1.16 | No | No | No | No | No | 0 | 3 | 0 | 0 | 2 | 0.55 |
| CHEMBL454823 | OC[C@H]1O[C@@H](Oc2cc(OC)cc3c2c(=O)cc(o3)c2cc(OC)c(c(c2)OC)OC)[C@@H]([C@H]([C@@H]1O)O)O | 520.48 | 8 | 12 | 4 | 128.03 | 166.51 | 1.06 | No | No | No | No | Yes | 2 | 1 | 1 | 1 | 2 | 0.17 |
| CHEMBL1269323 | OC[C@H]1O[C@@H](OC[C@H]2O[C@@H](OC(=O)[C@@]34CC[C@H]([C@@H]4[C@@H]4[C@](CC3)(C)[C@]3(C)CC[C@@H]5[C@]([C@H]3CC4)(C)CC[C@H]([C@@]5(C)C(=O)O)O)C(=C)C)[C@@H]([C@H]([C@@H]2O)O)O)[C@@H]([C@H]([C@@H]1O)O)O | 810.96 | 9 | 15 | 9 | 203.04 | 253.13 | 1.62 | No | No | No | No | No | 3 | 3 | 1 | 1 | 4 | 0.11 |
| CHEMBL3234602 | CC(=O)O[C@@H]1[C@H](O)[C@@H](C)[C@@]2([C@@]3([C@@H]1C(C)(C)CCC3)C)CC[C@]1(O2)CO[C@H](C1)O | 396.52 | 2 | 6 | 2 | 104.75 | 85.22 | 2.71 | No | No | No | No | No | 0 | 0 | 0 | 0 | 0 | 0.55 |
| CHEMBL446471 | OC1C[C@@]2(C)OC(=O)C=C2C(C1)(C)C | 196.24 | 0 | 3 | 1 | 52.51 | 46.53 | 1.53 | No | No | No | No | No | 0 | 0 | 0 | 0 | 1 | 0.55 |
| CHEMBL463048 | O[C@H]1C[C@@H]2[C@H]([C@@H]([C@@H]1O)O)N=C(c1c2cc2OCOc2c1O)O | 309.27 | 0 | 8 | 5 | 76.34 | 131.97 | -0.23 | No | No | No | No | No | 0 | 1 | 0 | 1 | 0 | 0.55 |
| CHEMBL448515 | COC1=CC(=O)C=C(C1=O)OC | 168.15 | 2 | 4 | 0 | 40.08 | 52.6 | 0.22 | No | No | No | No | No | 0 | 0 | 0 | 0 | 1 | 0.85 |
| CHEMBL2380793 | O=C1C[C@H](C)C2=C[C@H]3[C@@H](C[C@]2(C1)C)OC(=O)C3=C | 246.3 | 0 | 3 | 0 | 68.15 | 43.37 | 2.27 | No | No | No | No | No | 0 | 0 | 0 | 0 | 0 | 0.55 |
| CHEMBL595489 | COc1cc2CCN3C(c2cc1O)Cc1c(C3)c(OC)c(cc1)O | 327.37 | 2 | 5 | 2 | 95.05 | 62.16 | 2.38 | No | No | No | Yes | Yes | 0 | 0 | 0 | 0 | 0 | 0.55 |
| CHEMBL2437372 | COc1c(/C=C/C(=O)c2ccc(cc2)O)ccc(c1O)O | 286.28 | 4 | 5 | 3 | 78.81 | 86.99 | 2.14 | Yes | No | Yes | No | Yes | 0 | 0 | 0 | 0 | 0 | 0.55 |
| CHEMBL255297 | COC1=CC(=O)C(=CC1=O)[C@H](c1ccccc1)C=C | 254.28 | 4 | 3 | 0 | 72.62 | 43.37 | 2.53 | Yes | Yes | Yes | No | No | 0 | 0 | 0 | 0 | 0 | 0.85 |
| CHEMBL519725 | O=C1C[C@H](C/C=C/2\C(=O)O[C@H](C[C@]3(O[C@@H](C1)C(=O)C3)C)[C@H]2O)C(=C)C | 348.39 | 1 | 6 | 1 | 90.13 | 89.9 | 1.54 | No | No | No | No | No | 0 | 0 | 0 | 0 | 0 | 0.55 |
| CHEMBL506286 | O/N=C/1\Cc2cc(Br)c(c(c2)Oc2c(Br)cc(cc2Br)CC(=NO)C(=NCCc2ccc(Oc3cc(CCN=C1O)cc(Br)c3O)c(Br)c2)O)O | 1019.12 | 0 | 12 | 6 | 218.93 | 189.28 | 7.3 | No | No | No | No | No | 4 | 4 | 1 | 2 | 5 | 0.17 |
| CHEMBL478952 | OC/C=C/CC[C@@H]1C(=O)OC[C@@H]2[C@@H]1CCC(=CCCC2=C)C | 304.42 | 4 | 3 | 1 | 90.24 | 46.53 | 3.43 | No | Yes | Yes | No | No | 0 | 0 | 0 | 0 | 0 | 0.55 |
| CHEMBL250263 | O=C1OC[C@H]2C1=CC[C@@H]1[C@]2(C)[C@H](O)C[C@H]2[C@@]1(C)CC[C@@H]1[C@]2(C)CCCC1(C)C | 386.57 | 0 | 3 | 1 | 112.65 | 46.53 | 4.85 | No | No | Yes | No | No | 1 | 0 | 0 | 0 | 1 | 0.55 |
| CHEMBL484901 | OC(=O)C(Cc1c[nH]c2c1cccc2)N | 204.23 | 3 | 3 | 3 | 57.36 | 79.11 | 0.17 | No | No | No | No | No | 0 | 0 | 0 | 0 | 0 | 0.55 |
| CHEMBL171281 | N[C@@H](C(=O)O)CS | 121.16 | 2 | 3 | 2 | 28.94 | 102.12 | -1.31 | No | No | No | No | No | 0 | 4 | 0 | 0 | 3 | 0.55 |
| CHEMBL1644931 | C=CC(c1cc(/C=C/C(=O)c2ccc(cc2O)O)c(cc1O)OC)(C)C | 354.4 | 6 | 5 | 3 | 102.41 | 86.99 | 3.77 | Yes | No | Yes | No | Yes | 0 | 0 | 0 | 0 | 1 | 0.55 |
| CHEMBL2409058 | COc1cc2O[C@@H](CCc2c(c1)OC)c1ccc(c(c1)O)O | 302.32 | 3 | 5 | 2 | 82.11 | 68.15 | 2.56 | Yes | No | No | Yes | Yes | 0 | 0 | 0 | 0 | 0 | 0.55 |
| CHEMBL3787164 | CC[C@@H]([C@@H]1N=C(O)[C@@H](N=C(O)[C@H](Cc2ccccc2)N=C(O)[C@H](Cc2ccccc2)N=C(O)[C@H]2N(C(=O)[C@@H](N=C([C@@H](N=C([C@@H](N=C1O)CCS(=O)C)O)CC(C)C)O)CCS(=O)C)CCC2)Cc1c[nH]c2c1cccc2)C | 1098.38 | 16 | 17 | 8 | 339.9 | 336.79 | 5.46 | No | No | No | No | No | 3 | 4 | 2 | 2 | 6 | 0.17 |
| CHEMBL28457 | CC(=CC[C@@H](C1=CC(=O)c2c(C1=O)c(O)ccc2O)O)C | 288.3 | 3 | 5 | 3 | 77.82 | 94.83 | 2.08 | Yes | No | Yes | No | No | 0 | 0 | 0 | 0 | 0 | 0.55 |
| CHEMBL2322569 | N[C@@H]1CC[C@]2([C@H](C1)CC[C@@H]1[C@@H]2CC[C@]2([C@H]1CC[C@@H]2C(=O)C)C)C | 317.51 | 1 | 2 | 1 | 96.99 | 43.09 | 4.07 | No | No | No | No | No | 1 | 0 | 0 | 0 | 0 | 0.55 |
| CHEMBL3581419 | CO[C@@H]1O[C@H]2C[C@H](C(=C2[C@]2([C@H](C1)[C@@]1(C)[C@H](C[C@H]([C@@]3([C@@H]1[C@H]([C@H]2O)OC3)C)OC(=O)C)OC(=O)c1ccccc1)C)C)c1cocc1 | 620.73 | 7 | 9 | 1 | 163.72 | 113.66 | 4.15 | No | Yes | No | No | Yes | 1 | 3 | 0 | 0 | 1 | 0.55 |
| CHEMBL451707 | COc1cc(ccc1OC)c1cc(=O)c2c(o1)c(OC)c(cc2OC)OC | 372.37 | 6 | 7 | 0 | 100.38 | 76.36 | 2.98 | No | No | Yes | No | Yes | 0 | 0 | 0 | 0 | 0 | 0.55 |
| CHEMBL3794000 | CO[C@@H]1C=C2[C@@H]3CC(C)(C)CC[C@]3(CO)[C@H](C[C@]2([C@]2([C@H]1[C@@]1(C)CC[C@@H]([C@@]([C@@H]1CC2)(C)CO)O[C@@H]1O[C@H](C)[C@@H]([C@@H]([C@H]1O)O[C@@H]1O[C@H](CO)[C@H]([C@@H]([C@H]1O)O)O)O)C)C)O | 813.02 | 8 | 14 | 9 | 207.86 | 228.22 | 1.51 | No | No | No | No | No | 3 | 3 | 1 | 1 | 4 | 0.17 |
| CHEMBL469624 | O=C(C[C@H](C1=CC[C@@]2([C@]1(C)CC=C1[C@H]2CC[C@@H]2[C@]1(C)CC[C@@H](C2(C)C)O)C)C)C[C@H](C(=O)O)C | 470.68 | 6 | 4 | 2 | 139.01 | 74.6 | 5.31 | No | No | No | No | Yes | 1 | 3 | 0 | 1 | 1 | 0.85 |
| CHEMBL508791 | Oc1cc(O)cc(c1)Oc1c(O)cc(c2c1Oc1c(O)cc(cc1O2)Oc1c(O)cc(cc1O)Oc1c(O)cc(c2c1Oc1c(O)cc(cc1O2)O)O)O | 742.55 | 6 | 18 | 11 | 181.42 | 287.14 | 3.39 | No | No | Yes | No | No | 3 | 4 | 1 | 2 | 5 | 0.17 |
| CHEMBL447988 | COc1cc(ccc1O)C[C@H]([C@@H](Cc1ccc(c(c1)OC)O)COC(=O)/C=C/c1ccc(c(c1)OC)O)COC(=O)/C=C/c1ccc(c(c1)OC)O | 714.75 | 19 | 12 | 4 | 195.01 | 170.44 | 5.5 | No | Yes | Yes | No | No | 2 | 4 | 2 | 1 | 5 | 0.17 |
| CHEMBL3622812 | OC[C@H]1O[C@@H](O[C@@H]2OC=C([C@@]3([C@H]2[C@H](C)C[C@H]3O)O)C(=O)OC)[C@@H]([C@H]([C@@H]1O)O)O | 406.38 | 5 | 11 | 6 | 88.57 | 175.37 | -1.72 | No | No | No | No | No | 2 | 1 | 1 | 1 | 3 | 0.11 |
| CHEMBL950 | O=C(c1ccccc1)C(=O)O | 150.13 | 2 | 3 | 1 | 38.41 | 54.37 | 0.99 | No | No | No | No | No | 0 | 3 | 0 | 0 | 1 | 0.85 |
| CHEMBL67945 | CCCCCCCCCCCC[C@H]([C@H]1CC[C@@H](O1)[C@@H](CCCC[C@@H](CCCCC[C@H](CC1=C[C@@H](OC1=O)C)O)O)O)O | 596.88 | 26 | 7 | 4 | 172.67 | 116.45 | 6.93 | No | No | No | No | Yes | 1 | 4 | 1 | 1 | 2 | 0.55 |
| CHEMBL1807753 | O=C1CC(C)(C)C2=CC=C3C(=C(O)C(=O)C(=C3)C(C)C)[C@@]2(C1)C | 312.4 | 1 | 3 | 1 | 91.47 | 54.37 | 3.4 | No | Yes | Yes | No | Yes | 0 | 0 | 0 | 0 | 0 | 0.55 |
| CHEMBL423178 | CCCCC[C@@H](/C=C/C=C/C#CC#CCCCO)O | 260.37 | 8 | 2 | 2 | 81.53 | 40.46 | 3.7 | Yes | No | Yes | No | No | 0 | 0 | 0 | 0 | 0 | 0.55 |
| CHEMBL3092700 | CC(=CCOc1cccc2c1nc1cccc(c1n2)O)CCC=C(C)C | 348.44 | 6 | 4 | 1 | 107.87 | 55.24 | 4.81 | No | Yes | Yes | Yes | No | 0 | 0 | 0 | 0 | 1 | 0.55 |
| CHEMBL2334863 | COc1c2c(cc(c1OC)O)[C@H](O)[C@@H](C)[C@H](Cc1c2c(OC)c2c(c1)OCO2)C | 402.44 | 3 | 7 | 2 | 107.65 | 86.61 | 3.18 | No | No | No | Yes | No | 0 | 0 | 0 | 0 | 0 | 0.55 |
| CHEMBL485747 | OCCCc1ccc(c(c1)O)O | 168.19 | 3 | 3 | 3 | 46.23 | 60.69 | 0.81 | No | No | No | No | No | 0 | 0 | 0 | 0 | 1 | 0.55 |
| CHEMBL53757 | O=Cc1c(O)c(C)c2c(c1O)C(=O)C[C@H](O2)c1ccccc1 | 298.29 | 2 | 5 | 2 | 79.9 | 83.83 | 2.47 | Yes | No | Yes | No | Yes | 0 | 0 | 0 | 0 | 0 | 0.55 |
| CHEMBL474074 | OC[C@@H]([C@H](/C(=C/[C@H]([C@H]1C[C@H]([C@@H]2[C@]1(C)CC[C@H]1[C@H]2C[C@@H]([C@@H]2[C@]1(C)CC[C@@H](C2)O)O)O)C)/C)C)C | 462.7 | 5 | 4 | 4 | 136.71 | 80.92 | 4.54 | No | No | No | No | No | 0 | 2 | 0 | 0 | 1 | 0.55 |
| CHEMBL399742 | C=C1C[C@]23[C@H]([C@@H]1CC[C@@H]3[C@@]1([C@@H](CC2=O)C(C)(C)CCC1)C)O | 302.45 | 0 | 2 | 1 | 89.91 | 37.3 | 3.81 | No | No | Yes | No | No | 0 | 0 | 0 | 0 | 0 | 0.55 |
| CHEMBL254635 | C=C[C@@]1(C)CCC2=C(C1)CC[C@@H]1[C@]2(C)CC[C@@H](C1(C)C)O | 288.47 | 1 | 1 | 1 | 91.35 | 20.23 | 4.74 | No | Yes | Yes | No | No | 1 | 0 | 0 | 0 | 1 | 0.55 |
| CHEMBL2047346 | CCC/C=C/1\OC(=O)C2=C1CC[C@H]1[C@@H]2[C@]2([C@H]1CCC)OC(=O)C1=C2CCC=C1 | 380.48 | 4 | 4 | 0 | 107.62 | 52.6 | 4.51 | No | Yes | Yes | No | Yes | 1 | 0 | 0 | 0 | 0 | 0.55 |
| CHEMBL374606 | O=Cc1c(Br)c(O)c(c(c1Br)Br)O | 374.81 | 1 | 3 | 2 | 58.98 | 57.53 | 2.63 | No | No | Yes | No | No | 0 | 1 | 0 | 0 | 0 | 0.55 |
| CHEMBL3596258 | COC1=C(O)c2c(O)cc(c3c2c(C1=O)c(O)cc3O)C | 288.25 | 1 | 6 | 4 | 75.81 | 107.22 | 1.78 | Yes | No | Yes | No | Yes | 0 | 0 | 0 | 0 | 0 | 0.56 |
| CHEMBL452677 | CCCC=CCC=CCC=CCCCCC(=O)O[C@@H](CO[C@@H]1O[C@H](CO)[C@@H]([C@@H]([C@H]1O)O)O)COC(=O)CCCC=CCC=CCC=CCC=CCC=CCC | 771.03 | 33 | 10 | 4 | 221.91 | 151.98 | 7.4 | No | No | No | No | No | 1 | 4 | 2 | 2 | 4 | 0.55 |
| CHEMBL507346 | CC(=CC(=O)O[C@@H]1[C@@H](O)C(C)(C)C[C@@H]2[C@]1(CC[C@@]1(C2=CC[C@H]2[C@@]1(C)CC[C@@H]1[C@@]32CC[C@@](C1(C)C)(OC3)O)C)C(=O)O)C | 584.78 | 4 | 7 | 3 | 161.67 | 113.29 | 5.23 | No | No | No | No | Yes | 2 | 4 | 0 | 1 | 1 | 0.56 |
| CHEMBL303062 | O=C1C=C2[C@@]3(O1)C[C@@H](C=C2)N1[C@@H]3CCCC1 | 217.26 | 0 | 3 | 0 | 63.33 | 29.54 | 1.55 | No | No | No | No | No | 0 | 0 | 0 | 0 | 0 | 0.55 |
| CHEMBL570475 | CC(=CCc1c(O)cc(cc1O)C(=O)[C@H]1[C@H](C=C(C[C@@H]1c1ccc(cc1O)O)C)c1c(O)ccc(c1O)C(=O)/C=C/c1ccc(cc1O)O)C | 678.72 | 9 | 10 | 8 | 191.57 | 195.98 | 5.25 | No | No | No | No | No | 2 | 4 | 1 | 2 | 4 | 0.17 |
| CHEMBL449522 | CCCCCC(O)C | 116.2 | 4 | 1 | 1 | 36.92 | 20.23 | 1.99 | No | No | No | No | No | 0 | 2 | 0 | 0 | 2 | 0.55 |
| CHEMBL522898 | COc1cc2C[C@@H](Oc2c2c1C=COC=C2)C(O)(C)C | 274.31 | 2 | 4 | 1 | 77.05 | 47.92 | 2.37 | Yes | Yes | No | No | No | 0 | 0 | 0 | 0 | 0 | 0.55 |
| CHEMBL274510 | CC(=O)O[C@@H]1[C@@H](Cl)C2=C([C@@]3([C@@H]1C(C)(C)CCC3)C)COC2=O | 326.82 | 2 | 4 | 0 | 83.86 | 52.6 | 3.19 | No | No | Yes | No | No | 0 | 0 | 0 | 0 | 0 | 0.55 |
| CHEMBL1414 | COc1cc(cc(c1O)OC)C(=O)O | 198.17 | 3 | 5 | 2 | 48.41 | 75.99 | 0.99 | No | No | No | No | No | 0 | 0 | 0 | 0 | 1 | 0.56 |
| CHEMBL30909 | CCCC(C)C | 86.18 | 2 | 0 | 0 | 30.96 | 0 | 2.61 | No | No | No | No | No | 0 | 2 | 0 | 0 | 2 | 0.55 |
| CHEMBL558710 | C[C@@H]([C@H]1CC[C@@]2([C@]1(C)CC[C@@]13[C@H]2CC[C@@H]2[C@]3(C1)CC[C@H](C2(C)C)O)C)CC/C=C(/C(=O)O)\C | 456.7 | 5 | 3 | 2 | 136.91 | 57.53 | 6.47 | No | No | No | No | No | 1 | 3 | 0 | 1 | 1 | 0.85 |
| CHEMBL449242 | CC(=O)OC[C@@]1(C)[C@@H](CC[C@]2([C@H]1CC[C@]1([C@H]2CC(=O)/C/1=C(\C=CC=C([C@@H](OC(=O)C)CC=C(C)C)C)/C)C)C)OC(=O)C | 596.79 | 12 | 7 | 0 | 170.2 | 95.97 | 6.63 | No | No | Yes | No | No | 2 | 4 | 1 | 1 | 1 | 0.17 |
| CHEMBL2037047 | COc1c(O)cc2c(c1O)c(=O)c(c(o2)c1ccc(cc1)O)O[C@@H]1O[C@H](CO[C@@H]2O[C@@H](C)[C@@H]([C@H]([C@H]2O)O)O)[C@H]([C@@H]([C@H]1O)O)O | 624.54 | 7 | 16 | 9 | 145.85 | 258.43 | -1.13 | No | No | No | No | No | 3 | 4 | 1 | 1 | 4 | 0.17 |
| CHEMBL180970 | O=C1OC[C@H]([C@@H]1Cc1ccc2c(c1)OCO2)Cc1ccc2c(c1)OCO2 | 354.35 | 4 | 6 | 0 | 91.23 | 63.22 | 3.16 | Yes | Yes | Yes | Yes | Yes | 0 | 0 | 0 | 0 | 0 | 0.55 |
| CHEMBL458053 | COc1cc2oc(cc(=O)c2cc1CC=C(C)C)c1ccc(cc1)O | 336.38 | 4 | 4 | 1 | 100.15 | 59.67 | 4.19 | Yes | Yes | Yes | No | Yes | 0 | 0 | 0 | 0 | 1 | 0.55 |
| CHEMBL1213003 | CC(=CC(=O)O[C@H]1[C@@H]2[C@H](C)C(=O)O[C@@H]2[C@@H]([C@H]([C@H]2[C@@]1(C)C(=O)C=C2)C)O)C | 362.42 | 3 | 6 | 1 | 94.64 | 89.9 | 2.18 | No | No | No | No | No | 0 | 0 | 0 | 0 | 0 | 0.55 |
| CHEMBL2337720 | OC[C@@H](CC[C@@H]([C@H]([C@H]1[C@@H](O)C[C@@H]2[C@]1(C)[C@H](O)C[C@H]1[C@H]2CCC2=CC(=O)CC[C@]12C)C)O)C | 448.64 | 6 | 5 | 4 | 127.3 | 97.99 | 3.35 | No | No | No | No | No | 0 | 1 | 0 | 0 | 0 | 0.55 |
| CHEMBL154 | COc1ccc2c(c1)ccc(c2)[C@@H](C(=O)O)C | 230.26 | 3 | 3 | 1 | 66.79 | 46.53 | 2.76 | No | No | No | No | No | 0 | 0 | 0 | 0 | 0 | 0.85 |
| CHEMBL326421 | CC[C@H]1OC(=O)[C@H](C)[C@@H](O[C@@H]2O[C@H](C)C[C@@H]([C@H]2O)N(C)C)[C@@H](C)C[C@H](C(=O)C=C[C@H]1C)C | 453.61 | 4 | 7 | 1 | 125.3 | 85.3 | 2.81 | No | No | No | No | No | 0 | 1 | 0 | 0 | 0 | 0.55 |
| CHEMBL2063124 | O[C@H]1c2c(ccc(c2[C@@H](C[C@@]1(C)O)O)C)C(C)C | 250.33 | 1 | 3 | 3 | 71.75 | 60.69 | 2.06 | No | No | No | Yes | No | 0 | 0 | 0 | 0 | 0 | 0.55 |
| CHEMBL552055 | OC[C@@]1(C)[C@@H](O)CC[C@]2([C@H]1CC[C@@]1([C@@H]2CC=C2[C@]1(CC[C@@]1([C@H]2CC(CC1)(C)C)C)C(=O)O)C)C | 472.7 | 2 | 4 | 3 | 137.82 | 77.76 | 5.43 | No | No | No | No | No | 1 | 3 | 0 | 1 | 1 | 0.56 |
| CHEMBL63677 | Oc1ccc(cc1)c1cc(=O)c2c(o1)cc(c(c2O)c1cc(ccc1O)c1cc(=O)c2c(o1)cc(cc2O)O)O | 538.46 | 3 | 10 | 6 | 146.97 | 181.8 | 3.62 | No | No | No | No | No | 2 | 2 | 1 | 1 | 3 | 0.17 |
| CHEMBL467620 | CC(=O)CC[C@H]1C(=C)C(=O)O[C@@H]1[C@H]1C(=O)C=C[C@@]1(C)O | 278.3 | 4 | 5 | 1 | 71.93 | 80.67 | 1.09 | No | No | No | No | No | 0 | 0 | 0 | 0 | 0 | 0.55 |
| CHEMBL90 | NCCc1cnc[nH]1 | 111.15 | 2 | 2 | 2 | 31.07 | 54.7 | -0.02 | No | No | No | No | No | 0 | 3 | 0 | 0 | 1 | 0.55 |
| CHEMBL507409 | Oc1ccc(cc1)[C@@H]1[C@@H](c2cc(ccc2O)/C=C/c2cc(O)cc3c2[C@H](c2cc(O)cc(c2)O)[C@H](O3)c2ccc(cc2)O)c2cc(O)cc3c2[C@H](c2c1c(O)cc(c2)O)[C@H](O3)c1ccc(cc1)O | 906.93 | 7 | 12 | 10 | 255.47 | 220.76 | 6.86 | No | No | No | No | No | 3 | 4 | 1 | 2 | 6 | 0.17 |
| CHEMBL491987 | CC[C@@H](C[C@]12[C@@H]3C[C@@H]4N1CC[C@@H]2[C@]1(O3)[C@@H]4[C@@H](/C(=C\2/OC(=O)C(=C2OC)C)/O1)C)O | 403.47 | 4 | 7 | 1 | 106.82 | 77.46 | 1.38 | No | No | No | No | No | 0 | 0 | 0 | 0 | 0 | 0.56 |
| CHEMBL2041869 | OCC[C@]1(C)[C@H](CC[C@H]1[C@@H]1C[C@H]2O[C@]32[C@](C1=O)(C)CC[C@@H](C3)O)[C@@H](CCC(=C)C(C)C)C | 446.66 | 8 | 4 | 2 | 130.91 | 70.06 | 5.08 | No | No | No | No | No | 0 | 2 | 0 | 0 | 1 | 0.55 |
| CHEMBL1375951 | CC(=O)C1C(=O)C=C2C(C1=O)(C)c1c(O)c(C)c(c(c1O2)C(=O)C)O | 344.32 | 2 | 7 | 2 | 86.54 | 117.97 | 1.5 | No | No | No | No | No | 0 | 0 | 0 | 0 | 0 | 0.56 |
| CHEMBL551967 | O[C@@H]1C[C@@]2(C)[C@H]([C@]([C@H]1O)(C)C(=O)O)CC[C@@]1([C@@H]2CC=C2[C@@]1(C)CC[C@@]1([C@H]2[C@H](O)C(C)(C)CC1)C(=O)O)C | 518.68 | 2 | 7 | 5 | 140.75 | 135.29 | 3.44 | No | No | No | No | No | 1 | 3 | 0 | 1 | 0 | 0.56 |
| CHEMBL2299415 | O=C/C=C/c1ccc2c(c1)OCO2 | 176.17 | 2 | 3 | 0 | 47.6 | 35.53 | 1.91 | Yes | No | No | No | No | 0 | 0 | 0 | 0 | 1 | 0.55 |
| CHEMBL1490826 | Cc1ccc(c(c1)C)N | 121.18 | 0 | 0 | 1 | 40.78 | 26.02 | 1.9 | Yes | No | No | No | No | 0 | 1 | 0 | 0 | 2 | 0.55 |
| CHEMBL2322192 | C=C(C(C)C)CC[C@H]([C@H]1CC[C@@H]2[C@]1(C)CC[C@H]1[C@H]2CC[C@@H]2[C@]1(C)CC[C@@H]([C@H]2C)O)C | 414.71 | 5 | 1 | 1 | 133.23 | 20.23 | 7.29 | No | No | No | No | No | 1 | 3 | 0 | 1 | 2 | 0.55 |
| CHEMBL1760591 | OCCCc1ccc(c(c1)OC)O[C@@H]([C@@H](c1ccc(c(c1)OC)O)O)CO | 378.42 | 10 | 7 | 4 | 100.11 | 108.61 | 2 | No | No | No | Yes | No | 0 | 0 | 0 | 0 | 0 | 0.55 |
| CHEMBL2207535 | OC1=N[C@@H](Cc2c[nH]c3c2cccc3)C(=O)N([C@H]1C)C | 271.31 | 2 | 3 | 2 | 85.59 | 68.69 | 1.67 | No | No | No | No | No | 0 | 0 | 0 | 0 | 0 | 0.55 |
| CHEMBL1095257 | OCC1=C2C[C@]3(C)[C@@H](C)CC[C@H]4[C@]3(C[C@H]2OC1=O)O4 | 264.32 | 1 | 4 | 1 | 68.6 | 59.06 | 1.9 | No | No | No | No | No | 0 | 0 | 0 | 0 | 0 | 0.55 |
| CHEMBL82411 | OC(=O)Cc1c[nH]c2c1cccc2 | 175.18 | 2 | 2 | 2 | 49.84 | 53.09 | 1.51 | No | No | No | No | No | 0 | 0 | 0 | 0 | 1 | 0.85 |
| CHEMBL1172876 | COC(=O)C(=C)[C@@H]1[C@@H](OC(=O)C(=C)CO)C[C@]2([C@@H]([C@H]1O)C(=C)C(=O)OC2)C=C | 392.4 | 8 | 8 | 2 | 98.05 | 119.36 | 1.37 | No | No | No | No | No | 0 | 0 | 0 | 0 | 0 | 0.55 |
| CHEMBL482226 | OC/C=C(/CCCC(CC[C@H]1[C@@H](C)CCCC1(C)C)C)\C | 294.52 | 8 | 1 | 1 | 96.57 | 20.23 | 5.69 | No | No | Yes | Yes | No | 1 | 1 | 0 | 1 | 2 | 0.55 |
| CHEMBL387737 | OC1=C[C@@H]2C(=CC[C@@H]3[C@@]2(C)C(=O)C[C@]2([C@@]3(C)C[C@H]([C@@H]2[C@](C(=O)/C=C/C(O)(C)C)(O)C)O)C)C(C1=O)(C)C | 514.65 | 4 | 7 | 4 | 141.14 | 132.13 | 3.01 | No | No | No | No | Yes | 1 | 3 | 0 | 1 | 0 | 0.55 |
| CHEMBL2011652 | COc1cc(O)c2c(c1[C@H]1CC(=O)[C@@H]([C@H](O1)C)O)oc(cc2=O)c1ccc(cc1)O | 412.39 | 3 | 8 | 3 | 107.79 | 126.43 | 1.9 | No | No | No | No | Yes | 0 | 0 | 0 | 0 | 0 | 0.55 |
| CHEMBL1934198 | CC1(C)CCc2c(O1)cc1c(c2)ccc(=O)o1 | 230.26 | 0 | 3 | 0 | 66.29 | 39.44 | 2.92 | Yes | Yes | No | No | No | 0 | 0 | 0 | 0 | 0 | 0.55 |
| CHEMBL1928370 | CCCC(=O)O[C@]1(C)CCC(=O)C(=C)C[C@H]2O[C@@H]1[C@@H]1[C@@H](C(C)C)[C@H](O)C[C@@]([C@H]21)(C)OC(=O)C | 464.59 | 7 | 7 | 1 | 125.37 | 99.13 | 3.38 | No | No | No | No | Yes | 0 | 1 | 0 | 0 | 0 | 0.55 |
| CHEMBL1441727 | C[C@@H]1[C@@H](O)C[C@@H]2[C@H]1[C@H]1OC(=O)[C@H]([C@@H]1[C@H](CC2=C)O)C | 266.33 | 0 | 4 | 2 | 71.01 | 66.76 | 1.45 | No | No | No | No | No | 0 | 0 | 0 | 0 | 0 | 0.55 |
| CHEMBL513640 | CC(=CC[C@@H](C1=CC(=O)c2c(C1=O)c(O)ccc2O)OC(=O)C=C(C)C)C | 370.4 | 6 | 6 | 2 | 101.5 | 100.9 | 3.5 | Yes | Yes | Yes | No | Yes | 0 | 0 | 0 | 0 | 0 | 0.55 |
| CHEMBL509938 | OC(=N)CC[C@@H]1N=C(O)CN=C(O)[C@H](Cc2ccccc2)N(C)C(=O)[C@@H](N=C(CN=C([C@@H](N(C1=O)C)Cc1ccccc1)O)O)Cc1ccccc1 | 711.81 | 9 | 12 | 6 | 223.12 | 215.06 | 2.94 | No | No | No | No | No | 3 | 3 | 1 | 1 | 4 | 0.17 |
| CHEMBL1092163 | COc1ccc(cc1OC)C1=C(C[C@@H]2N(C1)CCC2)c1ccc(c(c1)OC)OC | 395.49 | 6 | 5 | 0 | 119.2 | 40.16 | 3.87 | No | No | No | Yes | Yes | 0 | 0 | 0 | 0 | 0 | 0.55 |
| CHEMBL150457 | CC1=CC(=O)C(=CC1=O)C | 136.15 | 0 | 2 | 0 | 37.91 | 34.14 | 1.22 | No | No | No | No | No | 0 | 3 | 0 | 0 | 1 | 0.55 |
| CHEMBL480663 | COc1cc2C[C@@H]3N([C@H](c2cc1O)Cc1c3cc2OCOc2c1)C | 325.36 | 1 | 5 | 1 | 92.6 | 51.16 | 2.58 | Yes | Yes | No | Yes | Yes | 0 | 0 | 0 | 0 | 0 | 0.55 |
| CHEMBL2262631 | C/C=C\1/CN(C)CC[C@@]23C(=O)C[C@@H]1[C@H]1CO[C@@H](C(=O)N([C@H]21)c1c3cccc1)O | 382.45 | 0 | 5 | 1 | 111.45 | 70.08 | 1.39 | No | No | No | No | No | 0 | 0 | 0 | 0 | 0 | 0.55 |
| CHEMBL448796 | O[C@@H]1[C@H]2COC(=O)c3cc(O)c(c(c3c3c(C(=O)O[C@@H]1[C@H]([C@@H](O2)OC(=O)c1cc(O)c(c(c1)O)O)O)cc(Oc1c(cc(c(c1O)O)O)C(=O)O)c(c3O)O)O)O | 802.56 | 6 | 23 | 14 | 179.37 | 397.65 | -0.66 | No | No | No | No | No | 3 | 3 | 1 | 1 | 4 | 0.11 |
| CHEMBL1173109 | N#Cc1ccc(cc1)Nc1cc(Oc2c(C)cc(cc2C)C)c(cc1N(=O)=O)N(=O)=O | 418.4 | 6 | 6 | 1 | 119.76 | 136.69 | 3.87 | No | Yes | Yes | No | Yes | 0 | 1 | 0 | 2 | 1 | 0.55 |
| CHEMBL277500 | c1ccccc1 | 78.11 | 0 | 0 | 0 | 26.44 | 0 | 2.14 | Yes | No | No | No | No | 0 | 3 | 0 | 0 | 2 | 0.55 |
| CHEMBL562256 | C=C1[C@@H]2CC[C@@H]3[C@](C1=O)([C@@H]2O)[C@H](O)C[C@H]1[C@@]3(C)[C@@H](O)C[C@@H](C1(C)C)O | 350.45 | 0 | 5 | 4 | 93.39 | 97.99 | 1.51 | No | No | No | No | No | 0 | 0 | 0 | 0 | 0 | 0.55 |
| CHEMBL522084 | OC[C@H]1O[C@@H](Oc2cc(O)c3c(c2)C(=O)c2c(C3=O)c(O)cc(c2)C)[C@@H]([C@H]([C@@H]1O)O)O | 432.38 | 3 | 10 | 6 | 102.91 | 173.98 | 0.22 | No | No | No | No | No | 1 | 1 | 1 | 1 | 2 | 0.55 |
| CHEMBL1162470 | N=c1[nH]cc([nH]1)[C@@H](c1[nH]c(=N)[nH]c1CCCNC(=O)c1[nH]c(c(c1)Br)Br)CCNC(=O)c1[nH]c(c(c1)Br)Br | 780.11 | 13 | 4 | 10 | 155.08 | 200.64 | 3.43 | No | No | No | No | Yes | 3 | 2 | 2 | 1 | 3 | 0.17 |
| CHEMBL444426 | CCCCCCCCCCCCCCCCCC[C@H](C(=N[C@H]([C@@H](/C=C/CCC=CCCCCCCCCC)O)CO[C@@H]1O[C@H](CO)[C@H]([C@@H]([C@H]1O)O)O)O)O | 770.13 | 36 | 10 | 7 | 224.96 | 172.43 | 8.56 | No | No | No | No | No | 2 | 4 | 2 | 2 | 5 | 0.17 |
| CHEMBL225562 | CC(=O)O[C@@H]1C[C@@H]2[C@@]3(C)CC[C@H](C([C@@H]3CC[C@]2([C@]2([C@H]1[C@H](CC2)[C@]1(C)CC[C@@H](O1)C(O)(C)C)C)C)(C)C)O | 518.77 | 4 | 5 | 2 | 149.1 | 75.99 | 5.48 | No | No | No | No | No | 2 | 4 | 0 | 1 | 1 | 0.17 |
| CHEMBL497273 | CC(=CCC[C@H]([C@H]1CC[C@@]2([C@]1(C)CCC1=C2CC[C@@H]2[C@]1(C)CC[C@H](C2(C)C)OC(=O)C)C)C(=O)O)C | 498.74 | 7 | 4 | 1 | 148.55 | 63.6 | 6.78 | No | No | Yes | No | No | 1 | 4 | 0 | 1 | 1 | 0.85 |
| CHEMBL2018715 | C[C@@H]1CCC[C@@H](O)/C=C/C(=O)O[C@@]23[C@@H](/C=C/C1)[C@H](O)C(=C([C@H]3[C@@H](N=C2O)Cc1ccccc1)C)C | 479.61 | 2 | 6 | 3 | 141.63 | 99.35 | 3.64 | No | No | No | No | Yes | 0 | 2 | 0 | 0 | 0 | 0.55 |
| CHEMBL327134 | CO/C=C(\[C@H]1C[C@H]2N(C[C@@H]1CC)CCc1c2[nH]c2c1cccc2)/C(=O)OC | 368.47 | 5 | 4 | 1 | 110.39 | 54.56 | 3.22 | No | No | No | Yes | Yes | 0 | 0 | 0 | 0 | 0 | 0.85 |
| CHEMBL1823040 | COc1cc(C#N)nc(c1)c1ccccn1 | 211.22 | 2 | 4 | 0 | 58.68 | 58.8 | 1.62 | Yes | No | No | No | Yes | 0 | 0 | 0 | 0 | 0 | 0.55 |
| CHEMBL252519 | COc1cc(/C=C/C(=O)Oc2cccc(c2)O)cc(c1O)OC | 316.31 | 6 | 6 | 2 | 84.58 | 85.22 | 2.55 | Yes | No | Yes | No | No | 0 | 0 | 0 | 0 | 0 | 0.55 |
| CHEMBL2289484 | COc1ccc(cc1)c1cc(O)c(c(c1OC)OC)c1ccc(cc1)OC | 366.41 | 6 | 5 | 1 | 105.31 | 57.15 | 4.22 | Yes | Yes | Yes | Yes | No | 0 | 0 | 0 | 0 | 0 | 0.55 |
| CHEMBL1782229 | CC(=O)OC[C@]12CC[C@H]3[C@H]([C@@H]1CC[C@@H]2[C@@H]([C@H]1CC[C@@H](C(=O)O1)C)C)CCC1=CC(=O)C=C[C@]31C | 468.62 | 5 | 5 | 0 | 132.25 | 69.67 | 4.96 | No | No | Yes | No | No | 1 | 2 | 0 | 0 | 1 | 0.55 |
| CHEMBL276218 | OC1=NCCCCC1 | 113.16 | 0 | 2 | 1 | 37.42 | 32.59 | 1.23 | No | No | No | No | No | 0 | 3 | 0 | 0 | 1 | 0.55 |
| CHEMBL1224859 | ClCCCC[C@H]1CCCC[C@H](C)[C@@H](O)c2cc(O)c(c(c2)O)[C@H](CCCC[C@@H]([C@H](c2cc(c1c(O)c2)O)O)C)CCCC(Cl)Cl | 688.16 | 8 | 6 | 6 | 189.46 | 121.38 | 7.42 | No | No | No | No | Yes | 3 | 4 | 0 | 1 | 3 | 0.17 |
| CHEMBL596254 | Clc1ccc(cc1)/C=C/1\Oc2c(C1=O)cccc2 | 256.68 | 1 | 2 | 0 | 70.83 | 26.3 | 3.65 | Yes | Yes | Yes | No | No | 0 | 0 | 0 | 0 | 0 | 0.55 |
| CHEMBL3092670 | C=C[C@H]1[C@@H](OC=C([C@H]1/C=C/c1c[n+](cc(c1)C(=O)O)[C@@H](C(=O)[O-])Cc1ccccc1)C(=O)O)O[C@@H]1O[C@H](CO)[C@H]([C@@H]([C@H]1O)O)O | 627.59 | 12 | 13 | 6 | 152.81 | 227.22 | -0.37 | No | No | No | No | No | 3 | 4 | 2 | 1 | 4 | 0.11 |
| CHEMBL2419614 | OCc1ccc(c(c1)OC)O[C@@H]1O[C@H](COC(=O)c2ccccc2)[C@H]([C@@H]([C@H]1O)O)O | 420.41 | 8 | 9 | 4 | 102.85 | 134.91 | 0.53 | No | No | No | No | No | 0 | 0 | 0 | 1 | 0 | 0.55 |
| CHEMBL470655 | CC(=CCc1cc(cc2c1OC(C)(C)C=C2)[C@@H]1CC(=O)c2c(O1)cc(cc2O)O)C | 406.47 | 3 | 5 | 2 | 117.39 | 75.99 | 4.48 | No | Yes | Yes | No | Yes | 0 | 0 | 0 | 0 | 1 | 0.55 |
| CHEMBL467759 | CC(=O)O[C@H]1C[C@H](O)C([C@@H]2[C@]1(C)[C@@H]1[C@@H](O)C[C@@H]3C[C@]1([C@H](O)C3=C)[C@H]([C@@H]2O)OC(=O)C)(C)C | 452.54 | 4 | 8 | 4 | 114.99 | 133.52 | 1.43 | No | No | No | No | No | 0 | 0 | 0 | 1 | 0 | 0.55 |
| CHEMBL2252750 | O=C1C[C@H]2C(C)(C)CCC[C@@]2(c2c1cc(C(C)C)c(c2O)O)C | 316.43 | 1 | 3 | 2 | 94.08 | 57.53 | 4.26 | No | No | Yes | No | Yes | 0 | 0 | 0 | 0 | 1 | 0.55 |
| CHEMBL1950778 | O[C@@H]1C[C@@H](O)[C@@H](C([C@H]1O)O)O | 164.16 | 0 | 5 | 5 | 34.65 | 101.15 | -1.84 | No | No | No | No | No | 0 | 2 | 0 | 0 | 2 | 0.55 |
| CHEMBL491172 | CC1=C(CCC2=C(C)CC[C@H]3[C@@]2(C)CCC(=O)C(O3)(C)C)[C@]2([C@H](CC1)OC([C@@H](CC2)O)(C)C)C | 472.7 | 3 | 4 | 1 | 140.01 | 55.76 | 5.33 | No | No | No | No | No | 0 | 3 | 0 | 1 | 0 | 0.55 |
| CHEMBL120568 | COc1cc(ccc1O)C(=O)O | 168.15 | 2 | 4 | 2 | 41.92 | 66.76 | 1.08 | No | No | No | No | No | 0 | 0 | 0 | 0 | 1 | 0.85 |
| CHEMBL240528 | Oc1ccc(cc1)c1oc2cc(O)cc(c2c(=O)c1O[C@@H]1O[C@@H](C)[C@@H]([C@H]([C@H]1O)O)O)O | 432.38 | 3 | 10 | 6 | 106.97 | 170.05 | 0.67 | No | No | No | No | No | 1 | 0 | 1 | 1 | 2 | 0.55 |
| CHEMBL430628 | CC(=O)O[C@H]1[C@H](OC(=O)C)C2=C(C)C(=O)C[C@H](C2(C)C)C[C@H]2[C@@]1(C)[C@@H](OC(=O)C)C[C@@H](C2=C)O | 476.56 | 6 | 8 | 1 | 124.5 | 116.2 | 2.68 | No | No | No | No | No | 0 | 0 | 0 | 0 | 0 | 0.55 |
| CHEMBL3594097 | COc1cc2c(cc1O)C[C@@]12COc2c(C1=O)c(O)cc(c2)O | 314.29 | 1 | 6 | 3 | 80.8 | 96.22 | 1.75 | Yes | No | Yes | No | Yes | 0 | 0 | 0 | 0 | 0 | 0.55 |
| CHEMBL502885 | OC[C@H]1O[C@@H](OCCc2ccc(cc2)O)[C@@H]([C@H]([C@@H]1OC(=O)/C=C/c1ccc(c(c1)O)O)O[C@@H]1O[C@@H](C)[C@@H]([C@H]([C@H]1O)O)O[C@@H]1O[C@@H](C)[C@@H]([C@H]([C@H]1O)O)O)O | 754.73 | 13 | 18 | 10 | 177.62 | 283.98 | -1.23 | No | No | No | No | No | 3 | 4 | 2 | 1 | 4 | 0.17 |
| CHEMBL2313421 | O=C1O[C@H](C=C1[C@H]1CC[C@@]2([C@@H]1CC[C@H]1[C@@]2(C)CC[C@@H]2[C@]1(C)CC[C@@H](C2(C)C)O)C)CC(O)(C)C | 472.7 | 3 | 4 | 2 | 137.89 | 66.76 | 5.56 | No | No | No | No | No | 1 | 3 | 0 | 1 | 1 | 0.55 |
| CHEMBL1171981 | CC1=CC(=O)[C@H]([C@]2([C@H]1C[C@H]1OC(=O)[C@@H]([C@@]3([C@]41[C@@H]2[C@](O)(OC4)[C@@H](C3=C)O)O)O)C)O | 408.4 | 0 | 9 | 5 | 94.67 | 153.75 | -1.17 | No | No | No | No | No | 0 | 1 | 1 | 1 | 2 | 0.55 |
| CHEMBL1933687 | COC(=O)c1cc(OC)c(c(c1)O)O | 198.17 | 3 | 5 | 2 | 48.26 | 75.99 | 1.07 | No | No | No | No | No | 0 | 0 | 0 | 0 | 1 | 0.55 |
| CHEMBL499359 | COC(=O)c1cc(O)c(c(c1)C(=O)C=C(C)C)O | 250.25 | 4 | 5 | 2 | 65.91 | 83.83 | 2.12 | No | No | No | No | No | 0 | 0 | 0 | 0 | 0 | 0.55 |
| CHEMBL521622 | CC(C/C=C/[C@H]([C@H]1CC[C@@H]2[C@]1(C)CC[C@H]1C2=C[C@@H]([C@@]2([C@]1(C)CC[C@@H](C2)O)O)O)C)C | 416.64 | 4 | 3 | 3 | 125.5 | 60.69 | 4.51 | No | No | No | No | No | 1 | 1 | 0 | 0 | 1 | 0.55 |
| CHEMBL2419873 | O[C@@H]([C@H]([C@H]1CC[C@@]2([C@]1(C)C[C@H]1O[C@]31[C@H]2CCC1=C(C3)C(=O)C=CC1(C)C)C)C)C[C@@H](C(=C)C)C | 466.7 | 5 | 3 | 1 | 140.84 | 49.83 | 6.17 | No | No | Yes | No | No | 1 | 3 | 0 | 1 | 1 | 0.55 |
| CHEMBL253779 | COc1cc(OC)ccc1CCC(=O)c1ccc(cc1)O | 286.32 | 6 | 4 | 1 | 80.94 | 55.76 | 2.98 | Yes | Yes | No | Yes | Yes | 0 | 0 | 0 | 0 | 0 | 0.55 |
| CHEMBL251229 | COc1cc2CC[n+]3c(c2cc1O)cc1c(c3)c(OC)c(cc1)O | 324.35 | 2 | 4 | 2 | 92.86 | 62.8 | 1.96 | Yes | No | No | Yes | Yes | 0 | 0 | 0 | 0 | 0 | 0.55 |
| CHEMBL502992 | C=C[C@@]1(C)CC(=O)[C@H]2[C@@](C1=O)(O)[C@H](OC(=O)C)C[C@@H]1[C@]2(C)[C@@H](OC(=O)c2ccccc2)[C@@H](OC(=O)C)[C@H](C1(C)C)OC(=O)C | 612.66 | 10 | 11 | 1 | 155.76 | 159.57 | 3 | No | No | No | No | Yes | 2 | 3 | 1 | 1 | 3 | 0.17 |
| CHEMBL1231330 | OC(=O)[C@H]([C@@H](C(=O)O)N)O | 149.1 | 3 | 6 | 4 | 28.75 | 120.85 | -2.66 | No | No | No | No | No | 0 | 4 | 0 | 0 | 3 | 0.56 |
| CHEMBL1909923 | Oc1ccc(c(c1)O)[C@H](CCc1ccc2c(c1)OCO2)O | 288.3 | 4 | 5 | 3 | 76.78 | 79.15 | 2.29 | Yes | No | No | Yes | No | 0 | 0 | 0 | 0 | 0 | 0.55 |
| CHEMBL444525 | CCCCCCCCCCCO | 172.31 | 9 | 1 | 1 | 56.15 | 20.23 | 3.59 | Yes | No | No | No | No | 0 | 0 | 0 | 0 | 2 | 0.55 |
| CHEMBL2409059 | COc1ccc2c(c1)O[C@@H](CC2)c1ccc(c(c1)OC)O | 286.32 | 3 | 4 | 1 | 80.09 | 47.92 | 3.02 | Yes | Yes | No | Yes | Yes | 0 | 0 | 0 | 0 | 0 | 0.55 |
| CHEMBL1797766 | OC[C@@H]1[C@H]2COc3c([C@@H]1c1c(C2)cc(c(c1OC)O)OC)c(OC)c(c(c3)OC)O | 418.44 | 5 | 8 | 3 | 108.72 | 106.84 | 2.3 | No | No | No | Yes | Yes | 0 | 0 | 0 | 0 | 0 | 0.55 |
| CHEMBL450607 | O[C@H]1C[C@@H](O[C@@H]2OC[C@@H]([C@@H]([C@H]2O[C@@H]2O[C@@H](C)[C@@H]([C@H]([C@H]2O)O)O)O)O)[C@]2(C(=CC[C@@H]3[C@@H]2CC[C@]2([C@H]3C[C@H]3[C@@H]2[C@H](C)[C@]2(O3)CCC(=C)CO2)C)C1)C | 706.86 | 4 | 12 | 6 | 179.92 | 176.76 | 1.77 | No | No | No | No | No | 3 | 3 | 1 | 1 | 5 | 0.17 |
| CHEMBL268808 | COC(=O)C1=CCCNC1 | 141.17 | 2 | 3 | 1 | 41.18 | 38.33 | 0.56 | No | No | No | No | No | 0 | 1 | 0 | 0 | 1 | 0.55 |
| CHEMBL103552 | Oc1ccc(c(c1)CN(c1ccc(c(c1)C(=O)O)O)Cc1ccccc1O)O | 381.38 | 6 | 6 | 5 | 104.67 | 121.46 | 2.35 | No | No | Yes | Yes | No | 0 | 0 | 0 | 0 | 0 | 0.56 |
| CHEMBL1311 | O=N(=O)O[C@@H]1CO[C@H]2[C@@H]1OC[C@@H]2O | 191.14 | 2 | 6 | 1 | 39.25 | 93.74 | -0.9 | No | No | No | No | No | 0 | 2 | 0 | 0 | 1 | 0.55 |
| CHEMBL1643851 | CCCCCC=CCCCCCCc1cc(=O)c2c(n1C)cccc2 | 339.51 | 11 | 1 | 0 | 111.65 | 22 | 5.87 | No | Yes | No | Yes | Yes | 1 | 1 | 1 | 1 | 1 | 0.55 |
| CHEMBL434864 | Oc1cc2O[C@@H]([C@@H](Cc2c(c1)O)OC(=O)c1cc(O)c(c(c1)O)O)c1cc(O)c(=O)c2c(c1)c(cc(c2O)O)[C@H]1Oc2cc(O)cc(c2C[C@H]1OC(=O)c1cc(O)c(c(c1)O)O)O | 868.7 | 8 | 20 | 13 | 215.39 | 351.12 | 1.48 | No | No | Yes | No | No | 3 | 3 | 1 | 1 | 5 | 0.17 |
| CHEMBL459397 | OC[C@@H]1CCCN2[C@@H]1CCCC2 | 169.26 | 1 | 2 | 1 | 53.93 | 23.47 | 1.46 | No | No | No | No | No | 0 | 0 | 0 | 0 | 1 | 0.55 |
| CHEMBL491542 | COc1cc2O[C@H](c3ccc(c(c3)O)O)[C@H](C(=O)c2c(c1)O)OC(=O)C | 360.31 | 4 | 8 | 3 | 88.96 | 122.52 | 1.46 | No | No | No | No | No | 0 | 0 | 0 | 0 | 0 | 0.55 |
| CHEMBL301982 | O=C1C=C2C(=CC=C3[C@@]2(C)CC[C@@]2([C@]3(C)CC[C@@]3([C@H]2C[C@@](C)(CC3)C(=O)O)C)C)C(=C1O)C | 450.61 | 1 | 4 | 2 | 131.29 | 74.6 | 5.12 | No | No | Yes | No | Yes | 1 | 3 | 0 | 1 | 1 | 0.85 |
| CHEMBL1773758 | CO[C@@H]1OC(=O)C2=CC[C@@H]3[C@]([C@@H]12)(C)[C@H](OC(=O)C)C[C@H]1[C@@]3(C)CC[C@@H]2[C@]1(C)CCCC2(C)C | 458.63 | 3 | 5 | 0 | 128.28 | 61.83 | 5.23 | No | No | Yes | No | No | 1 | 2 | 0 | 0 | 1 | 0.55 |
| CHEMBL460336 | O=C1CC[C@@H]2[C@]([C@H]1C)(C)CC[C@H]1[C@@]2(C)CC[C@@]2([C@]1(C)CC[C@@]1([C@H]2C[C@](C)(O)CC1)C(=O)O)C | 458.67 | 1 | 4 | 2 | 132.82 | 74.6 | 5.15 | No | No | No | No | No | 1 | 3 | 0 | 1 | 1 | 0.85 |
| CHEMBL1096256 | OC1=N[C@@H]2[C@@](N1C)(O)C[C@@H]1[C@H]2NC(=O)c2n1c(Br)cc2 | 341.16 | 0 | 4 | 3 | 84.59 | 90.09 | 0.11 | No | No | No | No | No | 0 | 1 | 0 | 0 | 0 | 0.55 |
| CHEMBL1277662 | O=C[C@]1(C)CCC[C@]2([C@H]1CCc1c2cc(c(c1)C(C)C)O)C | 300.44 | 2 | 2 | 1 | 91.84 | 37.3 | 4.26 | No | No | No | Yes | No | 0 | 0 | 0 | 0 | 0 | 0.55 |
| CHEMBL510560 | O=C1O[C@@H]2C[C@H]3C(=CC(=O)[C@H]([C@@]3([C@@H]3[C@@]42[C@@H]([C@H]1O)[C@@H](C)[C@H]([C@@]3(OC4)O)O)C)O)C | 394.42 | 0 | 8 | 4 | 93.95 | 133.52 | 0.07 | No | No | No | No | No | 0 | 1 | 0 | 1 | 0 | 0.55 |
| CHEMBL505211 | C=C[C@@]1(C)CC(=O)[C@H]2[C@@](C1=O)(O)[C@H](OC(=O)C)C[C@@H]1[C@]2(C)[C@@H](OC(=O)c2ccccc2)[C@@H](O)[C@H](C1(C)C)OC(=O)c1ccccc1 | 632.7 | 9 | 10 | 2 | 165.92 | 153.5 | 3.57 | No | No | No | No | Yes | 1 | 3 | 1 | 1 | 2 | 0.55 |
| CHEMBL563881 | C=C[C@@]1(C)[C@H](Cl)C[C@H]2[C@@]3([C@]1([N+]#[C-])[C@H](O)C(=O)C(c1c3c3c(C2(C)C)cccc3[nH]1)(C)C)O | 452.97 | 1 | 4 | 3 | 124.75 | 73.32 | 3.04 | No | No | No | No | Yes | 0 | 0 | 0 | 0 | 0 | 0.55 |
| CHEMBL106562 | COC1=CC(=O)c2c(C1=O)cccc2 | 188.18 | 1 | 3 | 0 | 50.14 | 43.37 | 1.43 | Yes | No | No | No | No | 0 | 0 | 0 | 0 | 1 | 0.85 |
| CHEMBL1928001 | COc1cc(ccc1OC)[C@H]1O[C@@H]([C@H]([C@H]1C)C)c1cc(OC)c(c(c1)OC)OC | 402.48 | 7 | 6 | 0 | 111.36 | 55.38 | 3.85 | No | No | No | Yes | No | 0 | 0 | 0 | 0 | 0 | 0.55 |
| CHEMBL257745 | COc1c2OCOc2c2c3c1CCN[C@@H]3Cc1c2cccc1 | 295.33 | 1 | 4 | 1 | 86.63 | 39.72 | 2.86 | Yes | Yes | No | Yes | Yes | 0 | 0 | 0 | 0 | 0 | 0.55 |
| CHEMBL513705 | COc1cc(ccc1OC)[C@@H]1O[C@@H]([C@H]([C@@H]1C)C)c1ccc(c(c1)OC)O | 358.43 | 5 | 5 | 1 | 100.4 | 57.15 | 3.48 | No | Yes | No | Yes | No | 0 | 0 | 0 | 0 | 0 | 0.55 |
| CHEMBL463022 | C=CCC=CCC=CCCCCCCCc1c(O)c(OC)cc(c1O)OC | 374.51 | 14 | 4 | 2 | 114.31 | 58.92 | 5.73 | Yes | No | Yes | Yes | Yes | 0 | 1 | 1 | 1 | 1 | 0.55 |
| CHEMBL405 | CC(Cc1ccccc1)N | 135.21 | 2 | 1 | 1 | 43.73 | 26.02 | 1.91 | Yes | No | No | No | No | 0 | 1 | 0 | 0 | 2 | 0.55 |
| CHEMBL1172874 | OCC(=C)[C@]12O[C@@H]2[C@]2(C(=CC1=O)CC[C@H]([C@@H]2C)O)C | 264.32 | 2 | 4 | 2 | 70.32 | 70.06 | 1.3 | No | No | No | No | No | 0 | 0 | 0 | 0 | 0 | 0.55 |
| CHEMBL480480 | CO[C@H]1OC(=O)C=C2C1=C[C@H]1OC(=O)[C@@]3([C@H]1[C@]2(C)CCC3)C | 304.34 | 1 | 5 | 0 | 77.56 | 61.83 | 2.21 | No | No | No | No | No | 0 | 0 | 0 | 0 | 0 | 0.55 |
| CHEMBL453161 | O=C(O[C@H]1CC[C@]2([C@H](C1(C)C)CC[C@@]1([C@@H]2CC[C@H]2[C@@]1(C)C[C@@H]([C@@]1([C@@H]2[C@@H](CC1)C(=C)C)C)O)C)C)/C=C/c1ccc(cc1)O | 588.86 | 5 | 4 | 2 | 177.68 | 66.76 | 7.82 | No | No | No | No | No | 2 | 4 | 0 | 1 | 1 | 0.17 |
| CHEMBL607579 | Oc1ccc2c(c1)c1c3OCOc3cc3c1c(c2)N=C3O | 279.25 | 0 | 5 | 2 | 81.69 | 71.28 | 2.77 | Yes | No | No | Yes | No | 0 | 0 | 0 | 0 | 0 | 0.55 |
| CHEMBL454064 | C=C1CC[C@]2([C@@H](C1)[C@]1(C)CC[C@@]3(C(=CC=C4C3=CC(=O)C(=C4C)O)[C@]1(CC2)C)C)C | 404.58 | 0 | 2 | 1 | 124.5 | 37.3 | 5.78 | No | No | Yes | No | Yes | 1 | 1 | 0 | 1 | 1 | 0.55 |
| CHEMBL461611 | O=C1CC[C@@H](O1)[C@@H](CCCCCCCCCC[C@H](CC1=C[C@@H](OC1=O)C)O)O | 396.52 | 14 | 6 | 2 | 108.06 | 93.06 | 3.87 | No | Yes | Yes | Yes | Yes | 0 | 0 | 1 | 0 | 0 | 0.55 |
| CHEMBL1442950 | OC[C@H]1O[C@H]([C@@H]([C@H]([C@@H]1O)O)O)c1c(O)c([C@@H]2O[C@H](CO)[C@H]([C@@H]([C@H]2O)O)O)c(c2c1oc(cc2=O)c1ccc(cc1)O)O | 594.52 | 5 | 15 | 11 | 139.23 | 271.2 | -1.98 | No | No | No | No | No | 3 | 4 | 1 | 1 | 4 | 0.17 |
| CHEMBL319344 | OC(=O)C1=CCC[C@@H]2[C@]1(C)CC[C@@H]([C@@]2(C)CCc1ccoc1)C | 316.43 | 4 | 3 | 1 | 92.33 | 50.44 | 4.24 | No | Yes | Yes | No | No | 0 | 0 | 0 | 0 | 1 | 0.85 |
| CHEMBL379845 | COC(=O)c1cccnc1 | 137.14 | 2 | 3 | 0 | 35.52 | 39.19 | 0.96 | No | No | No | No | No | 0 | 3 | 0 | 0 | 1 | 0.55 |
| CHEMBL523152 | COc1c(Cc2c3OC(C)(C)C(Cc3c(c(c2O)C(=O)C)O)O)c(O)c(c(c1C)O)C(=O)C | 460.47 | 5 | 9 | 5 | 120.8 | 153.75 | 2.52 | No | No | No | No | Yes | 0 | 0 | 1 | 1 | 1 | 0.55 |
| CHEMBL503534 | OCC1=C[C@H]2[C@@H]3C([C@]3(OC(=O)C)[C@@H]([C@H]([C@@]2([C@H]2[C@H](C1)C(=O)C(=C2)C)O)C)OC(=O)c1ccccc1NC)(C)C | 523.62 | 7 | 7 | 3 | 142.19 | 122.16 | 3.19 | No | No | No | No | Yes | 1 | 3 | 0 | 0 | 0 | 0.55 |
| CHEMBL451031 | COC(=O)C(CC(=O)C[C@H]([C@H]1CC(=O)[C@@]2([C@]1(C)[C@H](OC(=O)C)C(=O)C1=C2C(=O)C[C@@H]2[C@]1(C)CCC(=O)C2(C)C)C)C)C | 584.7 | 9 | 9 | 0 | 154.35 | 137.95 | 3.54 | No | No | No | No | No | 1 | 3 | 0 | 1 | 0 | 0.55 |
| CHEMBL1886292 | OC[C@@H]1O[C@H](Oc2ccc(c3c2c(=O)c2c(o3)cc(cc2O)O)O)[C@H]([C@@H]([C@H]1O)O)O | 422.34 | 3 | 11 | 7 | 100.2 | 190.28 | -0.31 | No | No | No | No | No | 2 | 1 | 1 | 1 | 3 | 0.17 |
| CHEMBL462853 | Cc1c(O)cc(cc1O)c1cc2c(o1)cccc2 | 240.25 | 1 | 3 | 2 | 70.66 | 53.6 | 3.17 | Yes | Yes | No | Yes | Yes | 0 | 0 | 0 | 0 | 0 | 0.55 |
| CHEMBL487419 | OC[C@H]1O[C@@H](O[C@H]2Oc3cc(OC)ccc3N=C2O)[C@@H]([C@H]([C@@H]1O)O)O | 357.31 | 4 | 10 | 5 | 84.96 | 150.43 | -0.76 | No | No | No | No | No | 0 | 1 | 1 | 1 | 1 | 0.55 |
| CHEMBL43192 | OCC1=C2[C@H](OC(=O)C)[C@@H]3CC(=O)[C@H]([C@@](C3(C)C)([C@H]([C@@H]([C@@]2(CC[C@@H]1O)C)OC(=O)C)OC(=O)C)O)C | 510.57 | 7 | 10 | 3 | 127.34 | 156.66 | 1.41 | No | No | No | No | No | 1 | 2 | 1 | 1 | 1 | 0.55 |
| CHEMBL497716 | C/C(=C/Cc1c(O)ccc(c1O)C(=O)/C=C/c1ccc(cc1)O)/COC(=O)/C=C/c1ccc(cc1)O | 486.51 | 10 | 7 | 4 | 138.57 | 124.29 | 4.65 | No | No | Yes | No | No | 0 | 2 | 0 | 0 | 1 | 0.55 |
| CHEMBL511832 | CCC(C(=O)c1c(O)c(CC=C(C)C)c(c2c1oc(=O)cc2c1ccccc1)O)C | 406.47 | 6 | 5 | 2 | 120.3 | 87.74 | 4.82 | No | No | Yes | No | Yes | 0 | 1 | 0 | 0 | 1 | 0.55 |
| CHEMBL1795554 | CCCCCCCCCCCCCCCCCCCCCc1cc(O)cc(c1)O | 404.67 | 20 | 2 | 2 | 131.59 | 40.46 | 8.52 | No | Yes | No | No | No | 1 | 3 | 1 | 1 | 2 | 0.55 |
| CHEMBL1224807 | COC1=CC(=O)c2c(C1=O)c(O)c1c(c2O)[C@H](O)[C@H]([C@@](C1)(C)O)O | 336.29 | 1 | 8 | 5 | 79.94 | 144.52 | -0.05 | No | No | No | No | No | 0 | 1 | 1 | 1 | 0 | 0.56 |
| CHEMBL1082603 | Oc1ccc(cc1)C1Oc2c([C@@H]1c1cc(O)cc3c1[C@H](c1cc(O)cc(c1)O)C(O3)c1ccc(cc1)O)cc(cc2)/C=C/c1cc(O)cc2c1[C@H](c1cc(O)cc(c1)O)[C@H](O2)c1ccc(cc1)O | 906.93 | 8 | 12 | 9 | 254.97 | 209.76 | 6.95 | No | No | No | No | No | 3 | 4 | 1 | 2 | 6 | 0.17 |
| CHEMBL1651090 | OC[C@@H]1CSC(=N1)c1ccccc1O | 209.26 | 2 | 3 | 2 | 61.5 | 78.12 | 1.38 | Yes | No | No | No | No | 0 | 0 | 0 | 0 | 0 | 0.55 |
| CHEMBL2064606 | CCCC(=O)c1c(O)c(C/C=C(/CCC=C(C)C)\C)c(c2c1oc(=O)cc2[C@@H](OC(=O)C)CC)O | 484.58 | 12 | 7 | 2 | 139.1 | 114.04 | 5.34 | No | Yes | Yes | No | Yes | 0 | 4 | 1 | 1 | 1 | 0.55 |
| CHEMBL503540 | COC[C@@H]1N=C(O)[C@@H]2CCC=NN2C(=O)[C@@H](OC(=O)[C@H](N=C([C@H]2N(C(=O)[C@H](N=C1O)[C@H](CC(=O)O)O)[C@@H]1Nc3c([C@@]1(C2)O)ccc(c3Cl)Cl)O)C1(C)CC1)C(C)(C)C | 852.71 | 7 | 16 | 7 | 235.11 | 276.07 | 1.32 | No | No | No | No | No | 3 | 3 | 1 | 1 | 4 | 0.11 |
| CHEMBL211174 | OC[C@H]1O[C@@H](C[C@@H]1O)n1cc(C)c(nc1=O)O | 242.23 | 2 | 6 | 3 | 57.27 | 104.81 | -0.52 | No | No | No | No | No | 0 | 1 | 0 | 0 | 0 | 0.55 |
| CHEMBL479730 | OC[C@H]1O[C@@H](Oc2ccc(cc2OC)[C@H]2OC[C@]3([C@@H]2CO[C@@H]3c2ccc(c(c2)OC)O)O)[C@@H]([C@H]([C@@H]1O)O)O | 536.53 | 7 | 12 | 6 | 128.22 | 176.76 | -0.08 | No | No | No | No | No | 3 | 2 | 1 | 1 | 3 | 0.17 |
| CHEMBL252746 | O=C1O[C@H](C[C@@H]([C@@H](O)C)C)[C@@H](C)C/C=C/[C@@](C)(O)C(=O)/C=C(\C[C@H](C/C=C/[C@@H]([C@@H](C(=C1)C)C)O)O)/C | 506.67 | 3 | 7 | 4 | 143.68 | 124.29 | 3.02 | No | No | No | No | Yes | 1 | 3 | 0 | 0 | 0 | 0.55 |
| CHEMBL8085 | NCCCC[C@@H](C(=O)O)N | 146.19 | 5 | 4 | 3 | 38.14 | 89.34 | -1.19 | No | No | No | No | No | 0 | 3 | 0 | 0 | 2 | 0.55 |
| CHEMBL1165343 | CC(=O)OC[C@]12CCCC([C@H]2C[C@H]([C@]23[C@H]1[C@@H](O)[C@H](O)[C@H]([C@H]2O)C(=C)C3=O)O)(C)C | 408.49 | 3 | 7 | 4 | 104.29 | 124.29 | 1.22 | No | No | No | No | No | 0 | 0 | 0 | 0 | 0 | 0.55 |
| CHEMBL567819 | OCCc1c(C)cc2c(c1C)C(=O)C(C2)(C)C | 232.32 | 2 | 2 | 1 | 69.71 | 37.3 | 2.99 | No | No | No | Yes | No | 0 | 0 | 0 | 0 | 0 | 0.55 |
| CHEMBL465444 | O[C@H]1CC[C@]2([C@](C1)(O)CC[C@@H]1[C@@H]2CC[C@]2([C@]31O[C@@H]3[C@@H]([C@@H]2c1ccc(=O)oc1)O)C)C | 416.51 | 1 | 6 | 3 | 110.03 | 103.43 | 2.27 | No | No | No | Yes | No | 0 | 0 | 0 | 0 | 0 | 0.55 |
| CHEMBL447997 | COc1cccc2c1n(C)c1c(c2=O)cco1 | 229.23 | 1 | 3 | 0 | 65.73 | 44.37 | 2.19 | Yes | No | No | No | Yes | 0 | 0 | 0 | 0 | 0 | 0.55 |
| CHEMBL448821 | COc1cc(cc(c1OC)O)[C@@H]1CCc2c(O1)c(O)c(cc2)OC | 332.35 | 4 | 6 | 2 | 88.6 | 77.38 | 2.64 | Yes | No | No | Yes | Yes | 0 | 0 | 0 | 0 | 0 | 0.55 |
| CHEMBL187504 | Oc1cc(O)c2c(c1)oc(cc2=O)c1ccc(cc1)Oc1cc(ccc1O)c1cc(=O)c2c(o1)cc(cc2O)O | 538.46 | 4 | 10 | 5 | 146.03 | 170.8 | 4.01 | No | No | Yes | No | No | 1 | 2 | 1 | 1 | 2 | 0.55 |
| CHEMBL510612 | COc1cc2CCN[C@H]3c2cc1Oc1c2c(CCN([C@H]2Cc2ccc(Oc4cc(C3)ccc4OC)cc2)C)cc(c1O)OC | 594.7 | 3 | 8 | 2 | 176.7 | 81.65 | 4.94 | No | No | No | No | No | 1 | 3 | 0 | 0 | 1 | 0.55 |
| CHEMBL471886 | C/C=C(/c1cc(O)c(c2c1C(=O)Oc1c(O2)c(cc(c1C)O)/C(=C/C)/C)CO)\C | 396.43 | 3 | 6 | 3 | 112.47 | 96.22 | 4.35 | No | No | Yes | No | No | 0 | 0 | 0 | 0 | 1 | 0.55 |
| CHEMBL498428 | CCC[C@H](C1=C(Br)C(=CBr)OC1=O)OC(=O)C | 368.02 | 5 | 4 | 0 | 70.24 | 52.6 | 2.78 | Yes | Yes | Yes | No | No | 0 | 0 | 0 | 0 | 0 | 0.55 |
| CHEMBL19393 | C[C@H](Cc1ccccc1)N | 135.21 | 2 | 1 | 1 | 43.73 | 26.02 | 1.91 | Yes | No | No | No | No | 0 | 1 | 0 | 0 | 2 | 0.55 |
| CHEMBL484662 | Oc1ccc(cc1)c1cc(=O)c2c(o1)cccc2O | 254.24 | 1 | 4 | 2 | 71.97 | 70.67 | 2.64 | Yes | No | No | Yes | Yes | 0 | 0 | 0 | 0 | 0 | 0.55 |
| CHEMBL478599 | CC(C[C@@H]1N=C([C@@]23[C@H]1[C@H](C)C(=C[C@@H]3C=C(C)CC[C@@H]([C@@H](C=CC(=O)O2)O)O)C)O)C | 417.54 | 2 | 6 | 3 | 121.95 | 99.35 | 2.87 | No | No | No | No | Yes | 0 | 0 | 0 | 0 | 0 | 0.55 |
| CHEMBL448376 | O[C@H]1CC(=C)[C@]2(C([C@@H]1Br)(C)C)CC[C@@](C=C2)(C)O | 315.25 | 0 | 2 | 2 | 78.75 | 40.46 | 2.94 | No | No | No | No | No | 0 | 0 | 0 | 0 | 0 | 0.55 |
| CHEMBL2036080 | OC[C@H]1O[C@@H](O[C@H]2CC[C@]3(C(=CC[C@@H]4[C@@H]3CC[C@]3([C@H]4C[C@H]4[C@@H]3[C@H](C)[C@]3(O4)CC[C@@H](CO3)C)C)C2)C)[C@@H]([C@H]([C@@H]1O)O)O[C@@H]1O[C@@H](C)[C@@H]([C@H]([C@H]1O)O)O | 722.9 | 5 | 12 | 6 | 185.2 | 176.76 | 2.29 | No | No | No | No | No | 3 | 3 | 1 | 1 | 5 | 0.17 |
| CHEMBL3311043 | O=c1c(coc2c1ccc1c2C=CC(O1)(C)C)c1ccc2c(c1)OCO2 | 348.35 | 1 | 5 | 0 | 98.1 | 57.9 | 3.76 | Yes | Yes | Yes | No | Yes | 0 | 0 | 0 | 0 | 0 | 0.55 |
| CHEMBL479432 | O=C(Cn1cnc2c(c1=O)cccc2)C[C@@H]1NCCC[C@H]1O | 301.34 | 4 | 5 | 2 | 86.88 | 84.22 | 0.88 | No | No | No | No | No | 0 | 0 | 0 | 0 | 0 | 0.55 |
| CHEMBL1439332 | O=C1CCCC=C1 | 96.13 | 0 | 1 | 0 | 28.57 | 17.07 | 1.21 | No | No | No | No | No | 0 | 3 | 0 | 0 | 2 | 0.55 |
| CHEMBL13473 | COc1ccc2c(c1)oc(cc2=O)c1ccc(c(c1)OC)OC | 312.32 | 4 | 5 | 0 | 87.4 | 57.9 | 3.19 | Yes | Yes | Yes | Yes | Yes | 0 | 0 | 0 | 0 | 0 | 0.55 |
| CHEMBL2375657 | COc1cc2OC[C@](C(=O)c2c(c1OC)OC)(O)Cc1ccc2c(c1)OCO2 | 388.37 | 5 | 8 | 1 | 97.05 | 92.68 | 2.24 | Yes | No | Yes | Yes | Yes | 0 | 0 | 0 | 0 | 0 | 0.55 |
| CHEMBL1078441 | CC(=CCOc1ccc2c(c1)oc(=O)cc2)CC[C@H](C(O)(C)C)O | 332.39 | 7 | 5 | 2 | 94.13 | 79.9 | 2.92 | No | No | No | No | No | 0 | 0 | 0 | 0 | 0 | 0.55 |
| CHEMBL249669 | Clc1cccc(c1)C(=O)[C@@H](NC(C)(C)C)C | 239.74 | 4 | 2 | 1 | 68.52 | 29.1 | 3.07 | Yes | No | No | Yes | No | 0 | 0 | 0 | 0 | 0 | 0.55 |
| CHEMBL249664 | COc1c2C(=O)C(=O)C=C(c2cc(c1O)C)C(C)C | 260.29 | 2 | 4 | 1 | 72.72 | 63.6 | 2.22 | Yes | No | No | No | No | 0 | 0 | 0 | 0 | 0 | 0.55 |
| CHEMBL89401 | OC[C@H]1Oc2ccc(cc2O[C@@H]1c1ccc(c(c1)OC)O)c1cc(=O)c2c(o1)cc(cc2O)O | 464.42 | 4 | 9 | 4 | 121.81 | 138.82 | 2.74 | No | No | Yes | No | Yes | 0 | 0 | 0 | 1 | 0 | 0.55 |
| CHEMBL465243 | O[C@H]([C@H](N)C)CCCCCCC=CCC=CCC=CCC=CCC=CCC=CC[C@@H]([C@H](N)C)O | 472.75 | 21 | 4 | 4 | 151.22 | 92.5 | 6.2 | Yes | No | Yes | No | Yes | 1 | 3 | 1 | 1 | 2 | 0.55 |
| CHEMBL1456697 | COc1cc(C=CC(=O)N2CCC=CC2=O)cc(c1OC)OC | 317.34 | 6 | 5 | 0 | 89.47 | 65.07 | 1.96 | No | No | No | No | No | 0 | 0 | 0 | 0 | 0 | 0.55 |
| CHEMBL448932 | CCC[C@H]1OC(=O)CCCC=C[C@@H](C1)O | 212.29 | 2 | 3 | 1 | 59.66 | 46.53 | 2.09 | No | No | No | No | No | 0 | 0 | 0 | 0 | 0 | 0.55 |
| CHEMBL2334859 | OCC([C@H](C(c1ccc(c(c1)O)OC)c1ccc(c(c1)O)OC)C(=O)O)CO | 392.4 | 9 | 8 | 5 | 101.06 | 136.68 | 1.48 | No | No | No | No | No | 0 | 0 | 0 | 1 | 0 | 0.56 |
| CHEMBL571655 | O=C1c2ccccc2c2c1cccc2 | 180.2 | 0 | 1 | 0 | 55.31 | 17.07 | 2.96 | Yes | Yes | No | No | No | 0 | 0 | 0 | 0 | 2 | 0.55 |
| CHEMBL320753 | CCCCCCCCCCCC[C@H]([C@H]1CC[C@@H](O1)[C@@H](CCCCCCCCCCCCC1=C[C@@H](OC1=O)C)O)O | 564.88 | 26 | 5 | 2 | 170.35 | 75.99 | 8.83 | No | No | No | No | No | 2 | 4 | 1 | 1 | 2 | 0.17 |
| CHEMBL460271 | C[C@H]([C@H](C(=O)C)O)C/C=C/[C@H]1[C@H](O)C(=C([C@@H]2[C@@]1(O)C(=N[C@H]2Cc1ccccc1)O)C)C | 427.53 | 7 | 6 | 4 | 125.06 | 110.35 | 2.27 | No | No | No | No | Yes | 0 | 0 | 0 | 0 | 0 | 0.55 |
| CHEMBL474037 | CC[C@@H]([C@@H]1N=C(O)[C@@H](N=C(O)[C@@H](N=C([C@@H](N=C1O)[C@@H]([C@H](O)C)C)O)Cc1ccccc1)C(C)C)C | 488.62 | 7 | 9 | 5 | 156.12 | 150.59 | 3.52 | No | No | No | No | Yes | 0 | 3 | 1 | 1 | 1 | 0.55 |
| CHEMBL1223628 | CC(=O)/C=C/C[C@]1(C)CC[C@@H](OO1)[C@H](C(=O)O)C | 256.29 | 5 | 5 | 1 | 66.2 | 72.83 | 1.67 | No | No | No | No | No | 0 | 0 | 0 | 0 | 0 | 0.85 |
| CHEMBL15075 | O/N=C(/C(=NCCc1cnc[nH]1)O)\Cc1ccc(c(c1)Br)OC | 381.22 | 7 | 6 | 3 | 91.13 | 103.09 | 2.39 | Yes | No | No | No | No | 0 | 0 | 0 | 0 | 0 | 0.55 |
| CHEMBL479882 | CC(=CCC1=C2OC(C)(C)C=CC2(C(=O)C(=C1O)C(=O)C(C)C)CC=C(C)C)C | 398.54 | 6 | 4 | 1 | 118.53 | 63.6 | 4.78 | No | Yes | Yes | No | Yes | 0 | 1 | 0 | 1 | 1 | 0.85 |
| CHEMBL1813343 | CCO[C@@H]1C[C@H]2C(C)(C)CCC[C@@]2(C2=C1C(=O)C(=C(C2=O)O)C(C)C)C | 360.49 | 3 | 4 | 1 | 103.11 | 63.6 | 3.95 | No | Yes | Yes | No | Yes | 0 | 0 | 0 | 0 | 0 | 0.85 |
| CHEMBL489133 | COC(=O)c1cc(O)c2c(c1)C=CC(O2)(C)C | 234.25 | 2 | 4 | 1 | 63.87 | 55.76 | 2.24 | Yes | No | No | No | No | 0 | 0 | 0 | 0 | 0 | 0.55 |
| CHEMBL485810 | O[C@H]1CC[C@]2([C@@H](C1)CC[C@@H]1[C@@H]2CC[C@]2([C@]31O[C@@H]3C[C@@H]2[C@]12C=CC(=O)O[C@@H]2O1)C)C | 400.51 | 1 | 5 | 1 | 106.38 | 71.59 | 3.38 | No | No | No | No | No | 0 | 0 | 0 | 0 | 0 | 0.55 |
| CHEMBL1946217 | CC(CC(=O)OC(c1coc(c1)c1cc(O)ccc1O)CC=C(C)C)C | 358.43 | 8 | 5 | 2 | 102.04 | 79.9 | 4.14 | Yes | Yes | Yes | No | Yes | 0 | 0 | 0 | 0 | 0 | 0.55 |
| CHEMBL1927830 | CCC(=O)O[C@@H]1[C@@H]2[C@@H](OC(=O)C)[C@]34CO[C@@]([C@H]([C@]2(C[C@@]1(C)OC(=O)C(C)C)OC(=O)C)OC(=O)C(C)C)([C@@H]4[C@H](C=C[C@H]3OC(=O)C)C(OC(=O)C)(C)C)C | 764.85 | 18 | 15 | 0 | 190.63 | 193.33 | 3.99 | No | Yes | No | No | No | 2 | 3 | 2 | 1 | 4 | 0.17 |
| CHEMBL1080425 | O=Cc1c(O)cc(c2c1Oc1c(OC2=O)c(O)c2c(c1C)C(=O)CC(O2)(C)C)C | 398.36 | 1 | 8 | 2 | 101.51 | 119.36 | 2.91 | No | No | Yes | No | No | 0 | 0 | 0 | 0 | 0 | 0.55 |
| CHEMBL389160 | CC1=C(C)C(=O)O[C@H](C1)[C@H]1CO[C@@]2([C@@]3([C@@]1(O)CC[C@H]3[C@H]1[C@H](C2)[C@@]2(C)C(=O)CCC[C@]32[C@@H](C1)O3)C)O | 486.6 | 1 | 7 | 2 | 127.01 | 105.59 | 2.76 | No | No | No | No | No | 0 | 2 | 0 | 0 | 0 | 0.55 |
| CHEMBL592589 | COC(=O)[C@]1(COC(=O)c2cc(OC)c(c(c2)OC)OC)[C@H]2C[C@H]3[C@@]45[C@@]1(C[C@H](O4)N3CC2=CC)c1ccccc1N5 | 562.61 | 9 | 9 | 1 | 154.21 | 104.79 | 3.26 | No | No | No | Yes | Yes | 1 | 3 | 0 | 0 | 0 | 0.55 |
| CHEMBL1094310 | COc1ccc(cc1)C/C=C/CCCCc1ccc(cc1)O | 296.4 | 8 | 2 | 1 | 92.78 | 29.46 | 4.85 | Yes | Yes | Yes | Yes | No | 1 | 0 | 0 | 0 | 1 | 0.55 |
| CHEMBL470344 | CCCC/C=C/CCc1ccc(cc1)O | 204.31 | 6 | 1 | 1 | 66.61 | 20.23 | 3.94 | Yes | No | No | Yes | No | 0 | 0 | 0 | 0 | 1 | 0.55 |
| CHEMBL182906 | COc1cc(O)cc(c1Cc1ccc(cc1)O)CCc1cccc(c1)O | 350.41 | 6 | 4 | 3 | 102.72 | 69.92 | 3.97 | Yes | Yes | Yes | Yes | No | 0 | 0 | 0 | 0 | 0 | 0.55 |
| CHEMBL500265 | OC[C@H]1O[C@H]([C@@H]([C@H]1O)O[C@@H]1OC[C@H]([C@@H]([C@H]1OC)O)OC)O[C@H](C(C)C)CC[C@H]([C@H]1C[C@H]([C@@H]2[C@]1(C)CC[C@H]1[C@@]2(O)C[C@@H]([C@@H]2[C@]1(C)CC[C@@H]([C@@H]2O)O)O)O)C | 760.95 | 12 | 14 | 8 | 192.23 | 217.22 | 0.76 | No | No | No | No | No | 3 | 3 | 2 | 1 | 4 | 0.17 |
| CHEMBL2204390 | COc1cc(cc2c1OCO2)[C@H]1O[C@@]2([C@]([C@@H]1C)(O2)C)c1cc(OC)c2c(c1)OCO2 | 414.41 | 4 | 8 | 0 | 102.9 | 77.14 | 3.29 | No | No | Yes | Yes | No | 0 | 0 | 0 | 0 | 0 | 0.55 |
| CHEMBL2011665 | COc1cc2[C@H](O)[C@H]3C[C@](C)(O)[C@@H](C[C@@H]3C(=O)c2c(c1)O)O | 308.33 | 1 | 6 | 4 | 78.25 | 107.22 | 0.72 | No | No | No | No | No | 0 | 0 | 0 | 0 | 0 | 0.55 |
| CHEMBL62448 | CC(=O)O[C@H]1CC[C@]2([C@H](C1(C)C)CC[C@]1([C@@H]2Cc2c(O)cc3c(c2O1)COC3=O)C)C | 428.52 | 2 | 6 | 1 | 115.81 | 82.06 | 4.13 | No | No | Yes | No | Yes | 0 | 0 | 0 | 0 | 0 | 0.55 |
| CHEMBL512938 | COc1cc(O)c(c(c1)CO)Oc1cc(C)cc(c1C(=O)O)O | 320.29 | 5 | 7 | 4 | 81.55 | 116.45 | 2 | Yes | No | Yes | No | Yes | 0 | 0 | 0 | 0 | 0 | 0.56 |
| CHEMBL1214423 | O[C@H]1CC[C@@]2(C3=C(OC[C@@H]13)C(=O)c1c2cc2C(=O)CC[C@@H](c2c1)O)C | 340.37 | 0 | 5 | 2 | 89.58 | 83.83 | 1.77 | No | No | No | No | No | 0 | 0 | 0 | 0 | 0 | 0.55 |
| CHEMBL66466 | O=Cc1cc(O)c2c(c1C=O)O[C@@]1([C@H](C2)[C@@]2(C)CC[C@@H](C([C@@H]2CC1)(C)C)OC(=O)C)C | 428.52 | 4 | 6 | 1 | 117.26 | 89.9 | 3.68 | No | No | No | No | Yes | 0 | 0 | 0 | 0 | 0 | 0.55 |
| CHEMBL218105 | COc1cc(/C=C/C(=O)O)ccc1OCC=C(C)C | 262.3 | 6 | 4 | 1 | 74.85 | 55.76 | 2.94 | No | Yes | No | No | No | 0 | 0 | 0 | 0 | 0 | 0.85 |
| CHEMBL505813 | C[C@H]1CCC=C([C@@]21CC[C@H](C2)C(O)(C)C)C | 222.37 | 1 | 1 | 1 | 70.46 | 20.23 | 3.56 | No | No | Yes | No | No | 0 | 0 | 0 | 0 | 1 | 0.55 |
| CHEMBL375563 | CC(=CCC[C@@]([C@H]1CC[C@@]2([C@@H]1[C@H](O)C[C@H]1[C@@]2(C)CC[C@@H]2[C@]1(C)CC[C@@H](C2(C)C)O)C)(O)C)C | 460.73 | 4 | 3 | 3 | 139.88 | 60.69 | 5.82 | No | No | No | No | No | 1 | 3 | 0 | 1 | 1 | 0.55 |
| Conformer3D_CID_115067 | OC[C@H]1O[C@@H](Oc2ccc(cc2)CO)[C@@H]([C@H]([C@@H]1O)O)O | 286.28 | 4 | 7 | 5 | 66.72 | 119.61 | -0.85 | Yes | No | No | No | No | 0 | 1 | 0 | 0 | 0 | 0.55 |
| Conformer3D_CID_1150 | NCCc1c[nH]c2c1cccc2 | 160.22 | 2 | 1 | 2 | 50.78 | 41.81 | 1.53 | Yes | No | No | No | No | 0 | 0 | 0 | 0 | 1 | 0.55 |
| CHEMBL1214365 | O=C1c2cc3c(cc2C(=O)C2=C1NCCS2(=O)=O)C(=O)c1c2[C@@]3(C)CCC(=O)c2co1 | 437.42 | 0 | 7 | 1 | 110.15 | 135.97 | 1.63 | No | No | Yes | No | No | 0 | 0 | 0 | 1 | 0 | 0.55 |
| Conformer3D_CID_1183 | COc1cc(C=O)ccc1O | 152.15 | 2 | 3 | 1 | 40.34 | 46.53 | 1.2 | No | No | No | No | No | 0 | 2 | 0 | 0 | 1 | 0.55 |
| Conformer3D_CID_119093 | OCC(=C)C(=O)O[C@H]1CC(=C)[C@H]2[C@@H]([C@@H]3[C@@H]1C(=C)C(=O)O3)C(=C)[C@H](C2)O | 346.37 | 4 | 6 | 2 | 90.1 | 93.06 | 1.41 | No | No | No | No | No | 0 | 0 | 0 | 0 | 0 | 0.55 |
| Conformer3D_CID_121947 | O[C@H]1[C@H](O)CC(=C[C@H]1OP(=O)(O)O)C(=O)O | 254.13 | 3 | 8 | 5 | 49.34 | 154.33 | -1.89 | No | No | No | No | No | 0 | 1 | 1 | 1 | 2 | 0.11 |
| Conformer3D_CID_126 | O=Cc1ccc(cc1)O | 122.12 | 1 | 2 | 1 | 33.85 | 37.3 | 1.17 | No | No | No | No | No | 0 | 3 | 0 | 0 | 1 | 0.55 |
| CHEMBL201358 | OC[C@H]1O[C@@H](Oc2ccc(cc2)C=O)[C@@H]([C@@H]([C@@H]1O)O)O | 284.26 | 4 | 7 | 4 | 65.97 | 116.45 | -0.51 | Yes | No | No | No | No | 0 | 1 | 0 | 0 | 0 | 0.55 |
| Conformer3D_CID_148195 | Clc1cc(Br)c2c(c1)CCc1c([C@@H]2C2CCN(CC2)C(=O)CC2CCN(CC2)C(=O)N)ncc(c1)Br | 638.82 | 5 | 3 | 1 | 157.3 | 79.53 | 4.8 | No | No | No | Yes | Yes | 2 | 2 | 0 | 0 | 1 | 0.17 |
| Conformer3D_CID_160511 | CN[C@H](C(=O)O)Cc1c[nH]c2c1cccc2 | 218.25 | 4 | 3 | 3 | 62.26 | 65.12 | 0.55 | No | No | No | No | No | 0 | 0 | 0 | 0 | 0 | 0.55 |
| Conformer3D_CID_1663 | NNCc1cccc(c1)O | 138.17 | 2 | 3 | 3 | 38.94 | 58.28 | 0.42 | No | No | No | No | No | 0 | 2 | 0 | 0 | 1 | 0.55 |
| CHEMBL563557 | O=C1N[C@@H](Cc2c[nH]c3c2cccc3)C(=O)N2[C@H]1CCC2 | 283.33 | 2 | 2 | 2 | 86.61 | 65.2 | 1.39 | Yes | No | No | Yes | No | 0 | 0 | 0 | 0 | 0 | 0.55 |
| Conformer3D_CID_194564 | CS[C@]12CC3=CC=C[C@@H]([C@H]3N2C(=O)[C@](N(C1=O)C)(SC)CO)O | 356.46 | 3 | 4 | 2 | 98.53 | 131.68 | 0.33 | No | No | No | No | No | 0 | 1 | 0 | 1 | 0 | 0.55 |
| Conformer3D_CID_199 | NCCCCN=C(N)N | 130.19 | 4 | 2 | 3 | 37.96 | 90.42 | -0.66 | No | No | No | No | No | 0 | 3 | 0 | 0 | 1 | 0.55 |
| CHEMBL266195 | C=CCc1ccccc1OCC(CNC(C)C)O | 249.35 | 8 | 3 | 2 | 75.04 | 41.49 | 2.79 | Yes | No | No | Yes | No | 0 | 0 | 0 | 0 | 0 | 0.55 |
| CHEMBL103489 | NCCCCCN=C(N)N | 144.22 | 5 | 2 | 3 | 42.76 | 90.42 | -0.26 | No | No | No | No | No | 0 | 2 | 0 | 0 | 1 | 0.55 |
| Conformer3D_CID_2346 | S=C=NCc1ccccc1 | 149.21 | 2 | 1 | 0 | 45.38 | 44.45 | 2.91 | No | No | No | No | No | 0 | 2 | 0 | 0 | 1 | 0.55 |
| Conformer3D_CID_24405 | OC[C@H]1O[C@H]([C@@H]([C@@H]1O)O)n1cnc2c1ncnc2NCC=C(C)C | 335.36 | 5 | 7 | 4 | 86.33 | 125.55 | 0.02 | No | No | No | No | No | 0 | 1 | 0 | 0 | 0 | 0.55 |
| CHEMBL1543 | SC[C@@H](C(=O)NCC(=O)O)NC(=O)CC[C@@H](C(=O)O)N | 307.32 | 11 | 7 | 5 | 70.37 | 197.62 | -2.36 | No | No | No | No | No | 0 | 1 | 2 | 1 | 2 | 0.11 |
| Conformer3D_CID_2901 | OC(=O)C1(N)CCCC1 | 129.16 | 1 | 3 | 2 | 33.36 | 63.32 | -0.62 | No | No | No | No | No | 0 | 2 | 0 | 0 | 2 | 0.55 |
| CHEMBL486189 | C=C1CCC[C@]2([C@H]1Cc1c(C2)occ1C)C | 216.32 | 0 | 1 | 0 | 67.1 | 13.14 | 3.81 | No | Yes | No | Yes | No | 0 | 0 | 0 | 0 | 1 | 0.55 |
| CHEMBL456 | CCC(=C)C(=O)c1ccc(c(c1Cl)Cl)OCC(=O)O | 303.14 | 6 | 4 | 1 | 73.67 | 63.6 | 3.14 | Yes | Yes | No | No | No | 0 | 0 | 0 | 0 | 0 | 0.85 |
| Conformer3D_CID_368 | NCCCCNCCCCN | 159.27 | 8 | 3 | 3 | 48.79 | 64.07 | 0.46 | No | No | No | No | No | 0 | 1 | 0 | 0 | 1 | 0.55 |
| Conformer3D_CID_370346 | O=C1c2cc3C(=O)C=CC(=O)c3cc2[C@]2(c3c1occ3C(=O)CC2)C | 332.31 | 0 | 5 | 0 | 86.97 | 81.42 | 2.45 | Yes | Yes | Yes | No | Yes | 0 | 0 | 0 | 0 | 0 | 0.55 |
| CHEMBL277041 | CSC[C@H]1O[C@H]([C@@H]([C@@H]1O)O)n1cnc2c1ncnc2N | 297.33 | 3 | 6 | 3 | 73.91 | 144.61 | -0.5 | No | No | No | No | No | 0 | 1 | 1 | 1 | 0 | 0.55 |
| CHEMBL350221 | OC(=O)[C@H](Cc1c[nH]c2c1cc(O)cc2)N | 220.22 | 3 | 4 | 4 | 59.38 | 99.34 | -0.17 | No | No | No | No | No | 0 | 0 | 0 | 0 | 0 | 0.55 |
| Conformer3D_CID_439463 | O[C@H]1[C@@H](CC(=C[C@H]1OP(=O)(O)O)C(=O)O)OC(=C)C(=O)O | 324.18 | 6 | 10 | 5 | 64.98 | 180.63 | -1.67 | No | No | No | No | No | 0 | 1 | 1 | 1 | 1 | 0.11 |
| Conformer3D_CID_445713 | C/C(=C\COP(=O)(OP(=O)(O)O)O)/CC/C=C(/CCC=C(C)C)\C | 382.33 | 11 | 7 | 3 | 95.78 | 132.91 | -1.67 | No | No | No | No | No | 0 | 1 | 1 | 1 | 1 | 0.11 |
| Conformer3D_CID_446987 | C[C@@H]1O[C@@H]1P(=O)(O)O | 138.06 | 1 | 4 | 2 | 26.49 | 79.87 | -0.76 | No | No | No | No | No | 0 | 3 | 0 | 0 | 2 | 0.56 |
| CHEMBL233929 | OC[C@H]1O[C@@H](Oc2cc(O)c3c(c2)oc(cc3=O)c2ccc(c(c2)O)O)[C@@H]([C@H]([C@@H]1O)O)O | 448.38 | 4 | 11 | 7 | 108.13 | 190.28 | 0.15 | No | No | No | No | No | 2 | 0 | 1 | 1 | 3 | 0.17 |
| CHEMBL86416 | CC1=C[C@H]2OC(=O)C(=C)[C@@H]2CCC(=CCC1)C | 232.32 | 0 | 2 | 0 | 69.85 | 26.3 | 2.97 | No | Yes | Yes | No | No | 0 | 0 | 0 | 0 | 0 | 0.55 |
| CHEMBL8260 | Oc1cc2oc(cc(=O)c2c(c1O)O)c1ccccc1 | 270.24 | 1 | 5 | 3 | 73.99 | 90.9 | 2.24 | Yes | No | No | Yes | Yes | 0 | 0 | 0 | 0 | 0 | 0.55 |
| CHEMBL487258 | O=C(O[C@@H]1C[C@](OC(=O)/C=C/c2ccc(c(c2)O)O)(C[C@H]([C@@H]1O)O)C(=O)O)/C=C/c1ccc(c(c1)O)O | 516.45 | 9 | 12 | 7 | 126.9 | 211.28 | 0.8 | No | No | No | No | No | 3 | 1 | 1 | 1 | 3 | 0.11 |
| CHEMBL6246 | Oc1cc2c(=O)oc3c4c2c(c1O)oc(=O)c4cc(c3O)O | 302.19 | 0 | 8 | 4 | 75.31 | 141.34 | 1 | Yes | No | No | No | No | 0 | 0 | 1 | 1 | 0 | 0.55 |
| CHEMBL1160061 | C/C(=C\CC/C(=C\COP(=O)(OP(=O)(O)O)O)/C)/CC/C=C(/CCC=C(C)C)\C | 450.44 | 14 | 7 | 3 | 119.34 | 132.91 | 1 | Yes | No | No | No | No | 0 | 0 | 1 | 1 | 0 | 0.55 |
| CHEMBL306908 | COc1c(OC/C=C(/CCC=C(C)C)\C)ccc2c1oc(=O)cc2 | 328.4 | 7 | 4 | 0 | 97.78 | 48.67 | 4.49 | Yes | Yes | Yes | No | Yes | 0 | 0 | 0 | 0 | 1 | 0.55 |
| CHEMBL503 | CC[C@@H](C(=O)O[C@H]1C[C@@H](C)C=C2[C@H]1[C@@H](CC[C@@H]1C[C@@H](O)CC(=O)O1)[C@H](C=C2)C)C | 404.54 | 7 | 5 | 1 | 113.92 | 72.83 | 3.88 | No | No | Yes | No | Yes | 0 | 0 | 0 | 0 | 0 | 0.55 |
| Conformer3D_CID_6223 | OC[C@@]12SS[C@@]3(N(C1=O)[C@@H]1[C@@H](O)C=CC=C1C3)C(=O)N2C | 326.39 | 1 | 4 | 2 | 86.8 | 131.68 | -0.19 | No | No | No | No | No | 0 | 1 | 0 | 1 | 0 | 0.55 |
| Conformer3D_CID_6271 | CCC(=O)c1ccc(cc1)O | 150.17 | 2 | 2 | 1 | 43.47 | 37.3 | 1.81 | Yes | No | No | No | No | 0 | 1 | 0 | 0 | 1 | 0.55 |
| CHEMBL285848 | CCCC[C@H](/C=C(/C=C(/C(=O)NC1=C[C@@](O)(/C=C/C=C/C=C/C(=O)NC2=C(O)CCC2=O)[C@H]2[C@@H](C1=O)O2)\C)\C)C | 550.64 | 14 | 7 | 4 | 151.73 | 145.33 | 1.81 | Yes | No | No | No | No | 0 | 1 | 0 | 0 | 1 | 0.55 |
| CHEMBL540445 | CC1=CCC[C@@]2(C)O[C@H]2[C@@H]2[C@@H](CC1)C(=C)C(=O)O2 | 248.32 | 0 | 3 | 0 | 69.34 | 38.83 | 2.7 | No | No | No | No | No | 0 | 0 | 0 | 0 | 0 | 0.55 |
| CHEMBL145 | OC(=O)/C=C/c1ccc(c(c1)O)O | 180.16 | 2 | 4 | 3 | 47.16 | 77.76 | 0.93 | No | No | No | No | No | 0 | 0 | 0 | 0 | 1 | 0.56 |
| CHEMBL221283 | OC/C=C(/C(=O)O[C@@H]1CC(=CCCC(=C[C@@H]2[C@@H]1C(=C)C(=O)O2)C)C)\CO | 362.42 | 5 | 6 | 2 | 97.02 | 93.06 | 1.96 | No | No | No | No | No | 0 | 0 | 0 | 0 | 0 | 0.55 |
| CHEMBL295830 | OC(=O)C1(N)CCCC1 | 129.16 | 1 | 3 | 2 | 33.36 | 63.32 | -0.62 | No | No | No | No | No | 0 | 2 | 0 | 0 | 2 | 0.55 |
| CHEMBL552941 | CN[C@H](C(=O)O)Cc1c[nH]c2c1cccc2 | 218.25 | 4 | 3 | 3 | 62.26 | 65.12 | 0.55 | No | No | No | No | No | 0 | 0 | 0 | 0 | 0 | 0.55 |
| CHEMBL350221 | OC(=O)[C@H](Cc1c[nH]c2c1cc(O)cc2)N | 220.22 | 3 | 4 | 4 | 59.38 | 99.34 | -0.17 | No | No | No | No | No | 0 | 0 | 0 | 0 | 0 | 0.55 |
| CHEMBL136356 | C[C@H]1CCC[C@]2(C1=C[C@H]1[C@@H](C2)OC(=O)C1=C)C | 232.32 | 0 | 2 | 0 | 67.95 | 26.3 | 3.19 | No | Yes | Yes | No | No | 0 | 0 | 0 | 0 | 0 | 0.55 |
| CHEMBL88985 | C=C1C(=O)O[C@H]2[C@H]1CCC(=C)[C@H]1[C@@H]2C(=C)CC1 | 230.3 | 0 | 2 | 0 | 67.74 | 26.3 | 3 | No | Yes | Yes | No | No | 0 | 0 | 0 | 0 | 0 | 0.55 |
| Conformer3D_CID_7419 | Nc1cccc(c1)C(=O)O | 137.14 | 1 | 2 | 2 | 37.81 | 63.32 | 0.58 | No | No | No | No | No | 0 | 3 | 0 | 0 | 1 | 0.85 |
| Conformer3D_CID_75110 | O=C1OC(=O)C(=C)C1 | 112.08 | 0 | 3 | 0 | 25.05 | 43.37 | 0.51 | No | No | No | No | No | 0 | 3 | 0 | 0 | 1 | 0.55 |
| Conformer3D_CID_802 | OC(=O)Cc1c[nH]c2c1cccc2 | 175.18 | 2 | 2 | 2 | 49.84 | 53.09 | 1.51 | No | No | No | No | No | 0 | 0 | 0 | 0 | 1 | 0.85 |
| Conformer3D_CID_8742 | O[C@@H]1CC(=C[C@H]([C@H]1O)O)C(=O)O | 174.15 | 1 | 5 | 4 | 38.43 | 97.99 | -1.1 | No | No | No | No | No | 0 | 2 | 0 | 0 | 1 | 0.56 |
| Conformer3D_CID_9064 | Oc1cc2O[C@H](c3ccc(c(c3)O)O)[C@H](Cc2c(c1)O)O | 290.27 | 1 | 6 | 5 | 74.33 | 110.38 | 0.83 | No | No | No | No | No | 0 | 0 | 0 | 0 | 0 | 0.55 |
| Conformer3D_CID_91457 | C=C1CCC[C@]2([C@H]1C[C@@H](CC2)C(O)(C)C)C | 222.37 | 1 | 1 | 1 | 70.46 | 20.23 | 3.6 | No | No | Yes | No | No | 0 | 0 | 0 | 0 | 1 | 0.55 |
| Conformer3D_CID_97472 | NCc1ccc(cc1)O | 123.15 | 1 | 2 | 2 | 36.14 | 46.25 | 0.73 | No | No | No | No | Yes | 0 | 3 | 0 | 0 | 1 | 0.55 |
| CHEMBL443917 | C[C@H]1C/C=C/[C@H]2[C@H](O)[C@](C)(O)[C@H]([C@@H]3[C@]2(C(=O)N[C@H]3Cc2c[nH]c3c2cccc3)C(=O)/C=C/C(=O)[C@@H](/C(=C/1)/C)O)C | 546.65 | 2 | 6 | 5 | 156.64 | 139.72 | 2.43 | No | No | No | No | Yes | 1 | 3 | 0 | 1 | 0 | 0.55 |
| CHEMBL516538 | OC[C@H]1[C@H](COC(=O)C)[C@H](O[C@@H]1c1cc(OC)c2c(c1)OCO2)c1cc(OC)c2c(c1)OCO2 | 474.46 | 8 | 10 | 1 | 116.07 | 111.14 | 2.45 | No | No | Yes | Yes | Yes | 0 | 0 | 0 | 0 | 0 | 0.55 |
| CHEMBL3185643 | CCCCOC(=O)C(=O)OCCCC | 202.25 | 9 | 4 | 0 | 52.75 | 52.6 | 2.1 | No | No | No | No | No | 0 | 0 | 0 | 0 | 0 | 0.55 |
| CHEMBL448371 | COc1cc(CCc2ccc(cc2)O)cc(c1)O | 244.29 | 4 | 3 | 2 | 71.24 | 49.69 | 2.89 | Yes | No | No | Yes | No | 0 | 0 | 0 | 0 | 0 | 0.55 |
| CHEMBL503867 | [O-]C(=O)[C@H]([N+](C)(C)C)Cc1c[nH]c2c1cccc2 | 246.3 | 4 | 2 | 1 | 69.5 | 55.92 | 0.16 | No | No | No | No | No | 0 | 0 | 0 | 0 | 0 | 0.55 |
| CHEMBL3753562 | COC(=O)[C@]1(Cc2ccc(cc2)O)OC(=O)C(=C1c1ccc(cc1)O)O | 356.33 | 5 | 7 | 3 | 91.17 | 113.29 | 1.93 | No | No | No | No | No | 0 | 0 | 0 | 0 | 0 | 0.55 |
| CHEMBL464817 | CC(=O)O[C@@H]1C/C=C(\C)/CC[C@H]2O[C@@]2(C[C@H]2[C@H](C/C=C/1\C)C(=C)C(=O)O2)C | 374.47 | 2 | 5 | 0 | 103.8 | 65.13 | 1.93 | No | No | No | No | No | 0 | 0 | 0 | 0 | 0 | 0.55 |
| CHEMBL1160785 | NCCc1ccc(c(c1)OC)O | 167.21 | 3 | 3 | 2 | 47.44 | 55.48 | 0.98 | No | No | No | No | No | 0 | 0 | 0 | 0 | 1 | 0.55 |
| CHEMBL2398585 | COc1cc(/C=C/C(=O)/C=C/c2ccc(cc2)O)ccc1O | 296.32 | 5 | 4 | 2 | 86.5 | 66.76 | 3.03 | Yes | No | Yes | No | Yes | 0 | 0 | 0 | 0 | 0 | 0.55 |
| CHEMBL487789 | O[C@H]1C[C@@H](C)[C@@]2(C3=C1[C@]1(C)CC[C@H](C([C@H]1C[C@H]3Oc1c(C2)cc(cc1)C(=O)O)(C)C)O)C | 440.57 | 1 | 5 | 3 | 124 | 86.99 | 3.94 | No | No | No | No | No | 0 | 0 | 0 | 0 | 0 | 0.56 |
| CHEMBL226683 | OC[C@@H]1C[C@H]([C@H]2N1[C@H](CO)[C@H]([C@H]2O)O)O | 219.23 | 2 | 6 | 5 | 53.77 | 104.39 | -1.86 | No | No | No | No | No | 0 | 1 | 0 | 0 | 1 | 0.55 |
| CHEMBL501870 | OC/C=C/c1ccc(c(c1)OC)O | 180.2 | 3 | 3 | 2 | 51.02 | 49.69 | 1.62 | No | No | No | No | No | 0 | 0 | 0 | 0 | 1 | 0.55 |
| CHEMBL518356 | CCCc1cc(=O)oc2c1c1OC(C)(C)C=Cc1c1c2[C@@H](O)[C@@H]([C@@H](O1)C)C | 370.44 | 2 | 5 | 1 | 106.11 | 68.9 | 3.78 | No | Yes | Yes | Yes | Yes | 0 | 0 | 0 | 0 | 0 | 0.55 |
| CHEMBL2252768 | O=C[C@H]1C(=C[C@H]([C@@H]2[C@]1(C)[C@H](OC(=O)/C=C/c1ccccc1)C[C@@H](C2=C)C)O)C=O | 394.46 | 6 | 5 | 1 | 110.86 | 80.67 | 2.87 | No | No | No | No | Yes | 0 | 0 | 0 | 0 | 0 | 0.55 |
| CHEMBL493857 | CC(=O)O[C@H]1CC[C@H]([C@@]23[C@]1(C)[C@@H](OC(=O)c1ccco1)C[C@H]([C@H]3OC(=O)c1ccco1)C(O2)(C)C)C | 500.54 | 8 | 9 | 0 | 125.81 | 114.41 | 3.66 | No | Yes | No | Yes | Yes | 1 | 1 | 0 | 0 | 0 | 0.55 |
| CHEMBL136686 | CO[C@@H]1CC2=CC[C@@H]3[C@@H]([C@]2(C[C@@H]1O)C)CC[C@]1([C@H]3CC=C1[C@@H](N(C)C)C)C | 373.57 | 3 | 3 | 1 | 112.7 | 32.7 | 3.93 | No | No | No | Yes | No | 0 | 0 | 0 | 0 | 0 | 0.55 |
| CHEMBL557501 | CC(=CCc1c2OC(C)(C)C=Cc2c(c2c1oc(c1ccc(cc1)O)c(c2=O)O)O)C | 420.45 | 3 | 6 | 3 | 121.83 | 100.13 | 4.36 | No | Yes | Yes | No | No | 0 | 0 | 0 | 0 | 1 | 0.55 |
| CHEMBL2386323 | OC1=C(C)c2ncc(c3c2c(C1=O)c(C)cc3)C | 239.27 | 0 | 3 | 1 | 71.42 | 50.19 | 2.69 | Yes | Yes | No | No | Yes | 0 | 0 | 0 | 0 | 0 | 0.55 |
| CHEMBL2271696 | O=Cc1c(O)c(O)c(c2c1c(O)cc(c2)C)C(C)C | 260.29 | 2 | 4 | 3 | 74.95 | 77.76 | 2.82 | Yes | No | Yes | No | Yes | 0 | 0 | 0 | 0 | 0 | 0.55 |
| CHEMBL563681 | CC(=O)O[C@@H]1[C@H]2CC[C@@H]3[C@]1([C@H](O)C[C@H]1[C@@]3(C)CCC(=O)C1(C)C)C(=O)C2=C | 374.47 | 2 | 5 | 1 | 101 | 80.67 | 2.66 | No | No | No | No | No | 0 | 0 | 0 | 0 | 0 | 0.55 |
| CHEMBL508961 | COc1cc(OC)c(c(c1)OC)[C@@H]1CC(=O)c2c(O1)cc(cc2O)OC | 360.36 | 5 | 7 | 1 | 93.49 | 83.45 | 2.67 | Yes | Yes | Yes | Yes | Yes | 0 | 0 | 0 | 0 | 0 | 0.55 |
| CHEMBL4216332 | COc1cc(/C=C/C(=O)OC[C@H]2O[C@@H](OCCc3ccc(cc3)O)[C@@H]([C@H]([C@@H]2O)O)O)ccc1O | 476.47 | 10 | 10 | 5 | 119.65 | 155.14 | 1.19 | No | No | No | No | No | 0 | 0 | 1 | 1 | 1 | 0.55 |
| CHEMBL249454 | OC[C@H]1O[C@H]2Oc3c4[C@@H](O[C@@H]2[C@H]([C@@H]1O)O)C[C@H](Oc4c(c(c3C)O)C)c1ccc(cc1)O | 446.45 | 2 | 9 | 5 | 111.01 | 138.07 | 1.17 | No | No | No | No | No | 0 | 0 | 0 | 1 | 0 | 0.55 |
| CHEMBL1835614 | CNCCc1c[nH]c2c1ccc(c2)Br | 253.14 | 3 | 1 | 2 | 63.38 | 27.82 | 2.75 | Yes | No | No | Yes | No | 0 | 0 | 0 | 0 | 0 | 0.55 |
| CHEMBL2204371 | COc1cc(O)c2c(c1C(CC(=C)C)O)oc(cc2=O)C | 290.31 | 4 | 5 | 2 | 80.85 | 79.9 | 2.54 | Yes | No | No | No | No | 0 | 0 | 0 | 0 | 0 | 0.55 |
| CHEMBL2227793 | COC[C@]12CC[C@@H]([C@@]34[C@@H]2[C@@H](OC)[C@@H](C3N(C1)CC)[C@@]1([C@@H]2[C@H]4C[C@@H]([C@@H]2O)[C@H](C1)OC)O)OC | 451.6 | 6 | 7 | 2 | 122.6 | 80.62 | 1.45 | No | No | No | No | No | 0 | 1 | 0 | 0 | 0 | 0.55 |
| CHEMBL2112849 | CCN1C[C@@]2(CC[C@@H]([C@]34C1[C@H](CC23)[C@@]1(O)C[C@@H]([C@H]2CC4[C@]1(O)[C@H]2OC)OC)OC)OC(=O)c1ccccc1NC(=O)C | 584.7 | 9 | 9 | 3 | 157.35 | 126.79 | 1.87 | No | No | No | Yes | No | 1 | 3 | 0 | 0 | 0 | 0.55 |
| CHEMBL3183581 | CC(=O)OC(C1CCC(=CC1)C)(C)C | 196.29 | 3 | 2 | 0 | 58.53 | 26.3 | 3.04 | No | No | Yes | No | No | 0 | 0 | 0 | 0 | 1 | 0.55 |
| CHEMBL520327 | CC(=O)O[C@@H]1/C=C(\C)/C[C@H](OC(=O)C)/C=C(\C)/CC[C@H]2[C@H](/C=C(/C1)\C)OC(=O)C2=C | 416.51 | 4 | 6 | 0 | 115.21 | 78.9 | 3.04 | No | No | Yes | No | No | 0 | 0 | 0 | 0 | 1 | 0.55 |
| CHEMBL517775 | C/C=C(\C(=O)O[C@H]1[C@H]2[C@@H](C[C@H]([C@H]3[C@@]1(C)C(=O)C=C3)C)OC(=O)C2=C)/CO | 360.4 | 4 | 6 | 1 | 94.16 | 89.9 | 1.84 | No | No | No | No | No | 0 | 0 | 0 | 0 | 0 | 0.55 |
| CHEMBL189707 | C/C=C(/C(=O)O[C@@H]1C[C@@]2(C)OC(=CC2=O)C(=C[C@@H]2[C@@H]1C(=C)C(=O)O2)CO)\C | 374.38 | 4 | 7 | 1 | 95.07 | 99.13 | 1.71 | No | No | No | No | No | 0 | 0 | 0 | 0 | 0 | 0.56 |
| CHEMBL45763 | CNCCc1ccccc1 | 135.21 | 3 | 1 | 1 | 43.82 | 12.03 | 1.99 | Yes | No | No | No | No | 0 | 1 | 0 | 0 | 2 | 0.55 |
| CHEMBL454333 | COc1c(OC)cc(cc1OC)[C@H]1[C@@H]2C(=O)OC[C@@H]2[C@H](c2c1cc1OCOc1c2)OC(=O)C | 456.44 | 6 | 9 | 0 | 113.58 | 98.75 | 2.77 | No | No | Yes | Yes | Yes | 0 | 0 | 0 | 0 | 0 | 0.55 |
| CHEMBL387124 | O=C1OC[C@H]2[C@@H]1CO[C@H]2c1ccc2c(c1)OCO2 | 248.23 | 1 | 5 | 0 | 59.65 | 53.99 | 1.47 | No | No | No | Yes | No | 0 | 0 | 0 | 0 | 0 | 0.55 |
| CHEMBL1080598 | COc1cc(ccc1OC)[C@H]1[C@H](C)[C@@H](C)C(=O)c2c1cc(OC)c(c2)O | 356.41 | 4 | 5 | 1 | 99.9 | 64.99 | 3.48 | No | Yes | No | Yes | Yes | 0 | 0 | 0 | 0 | 0 | 0.55 |
| CHEMBL383410 | CO[C@]12CC(=C[C@@H](O)CC(=C)CC[C@H]3C(C2=C(C(=O)O1)CC3)(C)C)C | 346.46 | 1 | 4 | 1 | 98.61 | 55.76 | 3.48 | No | Yes | Yes | No | Yes | 0 | 0 | 0 | 0 | 0 | 0.55 |
| CHEMBL464559 | Clc1cc(Cl)c(c(c1)[C@@H](c1[nH]c(c(c1[N+](=O)[O-])Cl)Cl)O)O | 371.99 | 3 | 4 | 3 | 82.29 | 102.07 | 3.11 | Yes | No | Yes | No | No | 0 | 0 | 0 | 0 | 0 | 0.55 |
| CHEMBL477778 | Oc1ccc2c(c1)OC[C@](C2=O)(O)Cc1ccc(c(c1)O)O | 302.28 | 2 | 6 | 4 | 77.58 | 107.22 | 1.3 | No | No | No | No | No | 0 | 0 | 0 | 0 | 0 | 0.55 |
| CHEMBL266195 | C=CCc1ccccc1OCC(CNC(C)C)O | 249.35 | 8 | 3 | 2 | 75.04 | 41.49 | 2.79 | Yes | No | No | Yes | No | 0 | 0 | 0 | 0 | 0 | 0.55 |
| CHEMBL415768 | OC[C@]1(C)[C@H](O)CC[C@@]2([C@@H]1CCC(=C)[C@H]2CCC1=CCOC1=O)C | 334.45 | 4 | 4 | 2 | 94.05 | 66.76 | 3.15 | No | No | No | No | No | 0 | 0 | 0 | 0 | 0 | 0.55 |
| CHEMBL464574 | CC(=O)O[C@H]1CCC([C@]2([C@@]1(C)[C@H]1Cc3occc3C(=O)[C@@H]1[C@H](C2)O)O)(C)C | 376.44 | 2 | 6 | 2 | 97.95 | 96.97 | 2.23 | No | No | No | Yes | No | 0 | 0 | 0 | 0 | 0 | 0.55 |
| CHEMBL3184590 | CCCCCCCCCC(=O)N(C)C | 199.33 | 9 | 1 | 0 | 62.89 | 20.31 | 3.23 | Yes | No | No | No | No | 0 | 0 | 0 | 0 | 1 | 0.55 |
| CHEMBL3357563 | CC(/C=C/C(=O)c1cocc1)C | 164.2 | 3 | 2 | 0 | 47.66 | 30.21 | 2.19 | Yes | Yes | No | No | No | 0 | 0 | 0 | 0 | 1 | 0.55 |
| CHEMBL229907 | COc1ccc(cc1)/C=C/C(=O)c1ccc(cc1O)OC | 284.31 | 5 | 4 | 1 | 81.26 | 55.76 | 3.21 | Yes | Yes | Yes | No | Yes | 0 | 0 | 0 | 0 | 0 | 0.55 |
| CHEMBL463974 | CC(=C)[C@H]1Cc2c(O1)ccc(c2)C(=O)C | 202.25 | 2 | 2 | 0 | 59.93 | 26.3 | 2.74 | Yes | No | No | No | No | 0 | 0 | 0 | 0 | 0 | 0.55 |
| CHEMBL1976572 | CO[C@@H]1C=C[C@]23[C@H](C1)N(C)C[C@@H]2OC(=O)c1c3cc2OCOc2c1 | 329.35 | 1 | 6 | 0 | 88.17 | 57.23 | 1.9 | No | No | No | Yes | No | 0 | 0 | 0 | 0 | 0 | 0.55 |
| CHEMBL2375789 | C/C=C(/C(=O)O[C@@H]1[C@@H](C)[C@]2(O)[C@@H]3C=C(C(=O)[C@@H]3CC(=C[C@H]2[C@H]2[C@]1(OC(=O)Cc1ccccc1)C2(C)C)CO)C)\C | 548.67 | 8 | 7 | 2 | 151.42 | 110.13 | 3.97 | No | No | Yes | No | Yes | 1 | 3 | 0 | 0 | 0 | 0.55 |
| CHEMBL361362 | COc1cc(cc(c1O)OC)[C@H]1OC[C@H]2[C@@H]1CO[C@@H]2c1cc(OC)c(c(c1)OC)O | 418.44 | 6 | 8 | 2 | 107.89 | 95.84 | 2.33 | No | No | No | Yes | No | 0 | 0 | 0 | 0 | 0 | 0.55 |
| CHEMBL301141 | O=c1cc(O)c2c(o1)cccc2 | 162.14 | 0 | 3 | 1 | 44.51 | 50.44 | 1.43 | Yes | No | No | No | No | 0 | 1 | 0 | 0 | 1 | 0.55 |
| CHEMBL479328 | CC(C[C@@H]1NC(=O)[C@@]23[C@H]1[C@H](C)C(=C[C@@H]3C=C(C)CC[C@@H]([C@@H](C=CC2=O)O)O)C)C | 401.54 | 2 | 4 | 3 | 118.9 | 86.63 | 2.51 | No | No | No | No | Yes | 0 | 0 | 0 | 0 | 0 | 0.55 |
| CHEMBL1080242 | COc1cc2cc(Cc3ccc(cc3)O)c(=O)oc2cc1O | 298.29 | 3 | 5 | 2 | 82.48 | 79.9 | 2.7 | Yes | No | No | Yes | Yes | 0 | 0 | 0 | 0 | 0 | 0.55 |
| CHEMBL506889 | C=C1[C@@H]2CC[C@H](C1(C)C)C2 | 136.23 | 0 | 0 | 0 | 45.22 | 0 | 3.43 | No | No | Yes | No | No | 1 | 1 | 0 | 0 | 2 | 0.55 |
| CHEMBL512489 | CO[C@H]1C[C@H](C)Cc2cc(ccc2O)NC(=O)/C(=C/CC[C@@H]([C@H](/C(=C/[C@@H]([C@H]1O)C)/C)OC(=O)N)OC)/C | 518.64 | 4 | 7 | 4 | 146.66 | 140.34 | 2.55 | No | No | No | No | Yes | 1 | 3 | 1 | 1 | 0 | 0.55 |
| CHEMBL3337754 | S=C=NCCCCC[S@+](C)[O-] | 191.31 | 6 | 2 | 0 | 53.2 | 80.73 | 2.19 | No | No | No | No | No | 0 | 0 | 0 | 0 | 1 | 0.55 |
| CHEMBL2386513 | CC(=C1C[C@H]2[C@@H]([C@](CC1=O)(C)O)CC[C@]2(C)O)C | 252.35 | 0 | 3 | 2 | 72.12 | 57.53 | 2.05 | No | No | No | No | No | 0 | 0 | 0 | 0 | 0 | 0.55 |
| CHEMBL457962 | O=c1ccc2c(o1)cc(c(c2)O)C | 176.17 | 0 | 3 | 1 | 49.47 | 50.44 | 1.85 | Yes | No | No | No | No | 0 | 0 | 0 | 0 | 1 | 0.55 |
| CHEMBL6466 | O=c1ccc2c(o1)cccc2 | 146.14 | 0 | 2 | 0 | 42.48 | 30.21 | 1.82 | Yes | No | No | No | No | 0 | 2 | 0 | 0 | 1 | 0.55 |
| CHEMBL1223695 | CC(=O)CCCC(C(=O)CCC(=O)C)(C)C | 226.31 | 8 | 3 | 0 | 64.94 | 51.21 | 2.13 | No | No | No | No | No | 0 | 0 | 0 | 0 | 0 | 0.55 |
| CHEMBL390320 | COc1cc2oc(=O)ccc2cc1O | 192.17 | 1 | 4 | 1 | 51 | 59.67 | 1.51 | Yes | No | No | No | No | 0 | 0 | 0 | 0 | 1 | 0.55 |
| CHEMBL399672 | C/C=C(\C(=O)O[C@@H]1C[C@]2(CO2)[C@H]2[C@]([C@@H]3[C@@H]1C(=C)C(=O)O3)(O)[C@]1([C@@H]([C@H]2O)O1)C)/C | 392.4 | 3 | 8 | 2 | 93.91 | 118.12 | 0.92 | No | No | No | No | No | 0 | 0 | 0 | 0 | 0 | 0.55 |
| CHEMBL147067 | O=C(c1ccccc1)/C=C/c1ccccc1O | 224.25 | 3 | 2 | 1 | 68.27 | 37.3 | 3.09 | No | Yes | Yes | No | No | 0 | 0 | 0 | 0 | 0 | 0.55 |
| CHEMBL1084170 | O[C@@H]1CCCc2cc(O)cc(c2C(=O)O[C@@H](C1)C)O | 266.29 | 0 | 5 | 3 | 69.82 | 86.99 | 1.78 | No | No | No | No | No | 0 | 0 | 0 | 0 | 0 | 0.55 |
| CHEMBL504850 | OC(=O)c1nc(C)c2c(c1)c1ccccc1[nH]2 | 226.23 | 1 | 3 | 2 | 65.53 | 65.98 | 1.95 | Yes | No | No | No | No | 0 | 0 | 0 | 0 | 0 | 0.85 |
| CHEMBL472877 | CC(=O)/C=C/C1C(=CCCC1(C)C)C | 192.3 | 2 | 1 | 0 | 61.48 | 17.07 | 3.31 | No | No | Yes | No | No | 0 | 0 | 0 | 0 | 2 | 0.55 |
| CHEMBL92528 | CCCc1cc(=O)oc2c1c1OC(C)(C)C=Cc1c1c2[C@@H](OC)[C@@H]([C@H](O1)C)C | 384.47 | 3 | 5 | 0 | 110.84 | 57.9 | 4.28 | No | Yes | Yes | Yes | Yes | 0 | 0 | 0 | 0 | 0 | 0.55 |
| CHEMBL466164 | C=C[C@@]1(C)C=C2CC[C@@H]3[C@@]([C@H]2C[C@H]1O)(C)CCC(=O)[C@@]3(C)CO | 318.45 | 2 | 3 | 2 | 92.71 | 57.53 | 3.16 | No | No | No | No | No | 0 | 0 | 0 | 0 | 0 | 0.55 |
| CHEMBL3233981 | OC[C@@]1(C)[C@@H](O)CC[C@@]2([C@@H]1C[C@@H](O)[C@]13[C@H]2CC(=O)[C@H]([C@H]1O)C(=C)C3=O)C | 364.43 | 1 | 6 | 4 | 93.59 | 115.06 | 0.78 | No | No | No | No | No | 0 | 0 | 0 | 0 | 0 | 0.55 |
| CHEMBL424997 | CC(=O)O[C@@H]1C[C@H]2O[C@@]2(C)C[C@H]2[C@H]([C@H]3C=C1C(=O)O3)C(=C)C(=O)O2 | 334.32 | 2 | 7 | 0 | 79.41 | 91.43 | 1.23 | No | No | No | No | No | 0 | 0 | 0 | 0 | 0 | 0.55 |
| CHEMBL592620 | OCC(=O)c1ccc(c(c1)OC)O | 182.17 | 3 | 4 | 2 | 46.31 | 66.76 | 0.58 | No | No | No | No | No | 0 | 0 | 0 | 0 | 1 | 0.55 |
| CHEMBL87 | COc1c(OC)cc2c(c1OC)c1ccc(c(=O)cc1C(CC2)NC(=O)C)OC | 399.44 | 6 | 6 | 1 | 109.36 | 83.09 | 2.36 | No | No | No | Yes | Yes | 0 | 0 | 0 | 0 | 0 | 0.55 |
| CHEMBL1834810 | CC[C@@H](C(=O)OCCCc1cc(OC)c2c(c1)cc(o2)c1ccc2c(c1)OCO2)C | 410.46 | 9 | 6 | 0 | 114.1 | 67.13 | 4.93 | No | Yes | Yes | Yes | Yes | 0 | 0 | 0 | 0 | 1 | 0.55 |
| CHEMBL490355 | Oc1ccc2c(c1)O/C(=C\c1ccc(c(c1)O)O)/C2=O | 270.24 | 1 | 5 | 3 | 71.89 | 86.99 | 1.9 | No | No | No | No | Yes | 0 | 0 | 0 | 0 | 0 | 0.55 |
| CHEMBL1988530 | CO[C@H]([C@H](/C(=C\C(=O)OC)/OC)C)/C=C/c1csc(n1)c1csc(n1)C(C)C | 422.56 | 10 | 6 | 0 | 114.14 | 127.02 | 4.14 | Yes | Yes | Yes | No | Yes | 0 | 0 | 0 | 0 | 0 | 0.56 |
| CHEMBL442565 | CC1=CCC2CC1C2(C)C | 136.23 | 0 | 0 | 0 | 45.22 | 0 | 3.44 | No | No | Yes | No | No | 1 | 1 | 0 | 0 | 2 | 0.55 |
| CHEMBL519395 | COc1cc(O)c(c(c1)C1=C(C)C[C@H](C1=O)O)C(=O)O | 278.26 | 3 | 6 | 3 | 70.48 | 104.06 | 1.17 | No | No | No | No | No | 0 | 0 | 0 | 0 | 0 | 0.56 |
| CHEMBL14117 | COC[C@H]1O[C@H](O)[C@@H]([C@H]([C@H]1O)O)O | 194.18 | 2 | 6 | 4 | 40.47 | 99.38 | -2.2 | No | No | No | No | No | 0 | 1 | 0 | 0 | 2 | 0.55 |
| CHEMBL1087405 | CC1=C[C@H](O)[C@H]2[C@H](CC(=C)C(=O)CC1)OC(=O)C2=C | 262.3 | 0 | 4 | 1 | 71.22 | 63.6 | 1.59 | No | No | No | No | No | 0 | 0 | 0 | 0 | 0 | 0.55 |
| CHEMBL448874 | Oc1ccc2c(c1)c(=O)c1c(o2)ccc(c1)O | 228.2 | 0 | 4 | 2 | 64.04 | 70.67 | 2.04 | Yes | No | No | Yes | Yes | 0 | 0 | 0 | 0 | 0 | 0.55 |
| CHEMBL1667883 | O=C(/C=C/C1(O)C=CC(=O)C=C1)OC[C@H]1O[C@@H](Oc2ccc(cc2)O)[C@@H]([C@H]([C@@H]1O)O)O | 434.39 | 7 | 10 | 5 | 103.86 | 162.98 | -0.5 | No | No | No | No | No | 0 | 1 | 1 | 1 | 1 | 0.55 |
| CHEMBL1712170 | CCOc1ccc2c3c1O[C@@H]1[C@@]43CCN([C@H](C2)[C@@H]4C=C[C@@H]1O)C | 313.39 | 2 | 4 | 1 | 91.55 | 41.93 | 2.12 | No | No | No | Yes | No | 0 | 0 | 0 | 0 | 0 | 0.55 |
| CHEMBL3348832 | O[C@@H]1CC(=CC(C1O)O)C(=O)O | 174.15 | 1 | 5 | 4 | 38.43 | 97.99 | -1.12 | No | No | No | No | No | 0 | 2 | 0 | 0 | 1 | 0.56 |
| CHEMBL517080 | OCC1=C(C)C[C@@H](OC1=O)[C@H]([C@H]1CC[C@@H]2[C@]1(C)CC[C@H]1[C@H]2C[C@@H]2[C@]3([C@]1(C)C(=O)C=C[C@@H]3O)O2)C | 470.6 | 3 | 6 | 2 | 127.49 | 96.36 | 3.42 | No | No | No | No | No | 0 | 1 | 0 | 0 | 0 | 0.55 |
| CHEMBL497134 | OC[C@H]1O[C@@H](Oc2ccc(cc2CO)O)[C@@H]([C@H]([C@@H]1O)O)OC(=O)c1ccccc1 | 406.38 | 7 | 9 | 5 | 98.38 | 145.91 | 0.4 | No | No | No | No | No | 0 | 0 | 1 | 1 | 0 | 0.55 |
| CHEMBL575429 | OC(=O)/C=C/c1ccc(cc1)OS(=O)(=O)O | 244.22 | 4 | 6 | 2 | 55.33 | 109.28 | 0.8 | No | No | No | No | No | 0 | 0 | 0 | 0 | 0 | 0.56 |
| CHEMBL457713 | O=C(C(=C)C)O[C@H]1C[C@H]2[C@@](C3=CC[C@H]([C@]13C)c1ccoc1)(C)[C@H](O)[C@H]1[C@@H]3[C@]2(C)C(=O)C=C[C@@]3(C)CO1 | 492.6 | 4 | 6 | 1 | 134.55 | 85.97 | 3.99 | No | No | No | No | No | 0 | 3 | 0 | 0 | 0 | 0.55 |
| CHEMBL1094876 | CCO[C@@H]1[C@@H](C)[C@@H](C)Cc2c(c3c1cc(OC)c(c3OC)OC)c(OC)c(c(c2)OC)OC | 460.56 | 8 | 7 | 0 | 128.58 | 64.61 | 4.37 | No | No | No | Yes | No | 0 | 0 | 0 | 0 | 1 | 0.55 |
| CHEMBL444810 | O=C1NCCc2c1[nH]c1c2cccc1 | 186.21 | 0 | 1 | 2 | 58.06 | 44.89 | 1.65 | Yes | No | No | No | No | 0 | 0 | 0 | 0 | 1 | 0.55 |
| CHEMBL326602 | CC(CN)O | 75.11 | 1 | 2 | 2 | 20.4 | 46.25 | -0.39 | No | No | No | No | No | 0 | 4 | 0 | 0 | 2 | 0.55 |
| CHEMBL508813 | O=Cc1ccc2c1cc1c(C)coc1cc2C | 224.25 | 1 | 2 | 0 | 68.16 | 30.21 | 3.41 | Yes | Yes | No | No | No | 0 | 0 | 0 | 0 | 0 | 0.55 |
| CHEMBL463165 | O=C1c2ccccc2N([C@H]2N1CCc1c2[nH]c2c1cccc2)C | 303.36 | 0 | 1 | 1 | 97.67 | 39.34 | 2.7 | Yes | Yes | No | Yes | Yes | 0 | 0 | 0 | 0 | 0 | 0.55 |
| CHEMBL1966898 | COc1cc(OC)c2c(c1)c(OC)c1c(n2)occ1 | 259.26 | 3 | 5 | 0 | 70.99 | 53.72 | 2.53 | Yes | Yes | Yes | Yes | Yes | 0 | 0 | 0 | 0 | 0 | 0.55 |
| CHEMBL250348 | CC(=O)O[C@@H]1C=C(C)[C@@H](CC=C(C[C@H]2[C@H]1C(=C)C(=O)O2)C)OC(=O)C | 348.39 | 4 | 6 | 0 | 91.65 | 78.9 | 2.4 | No | No | No | No | No | 0 | 0 | 0 | 0 | 0 | 0.55 |
| CHEMBL103686 | N[C@@H](C(=O)O)CCCN | 132.16 | 4 | 4 | 3 | 33.34 | 89.34 | -1.76 | No | No | No | No | No | 0 | 3 | 0 | 0 | 2 | 0.55 |
| CHEMBL34869 | O=c1[nH]c2nc(n(c2c(=O)[nH]1)C)O | 182.14 | 0 | 4 | 3 | 44.26 | 103.77 | -0.35 | No | No | No | No | No | 0 | 2 | 0 | 0 | 1 | 0.55 |
| CHEMBL1779811 | C/C=C/C[C@@H]([C@H]([C@H]([C@@H](C[C@H]1OC(=O)C=C[C@H]1CC)O)C)OC)C | 324.45 | 9 | 4 | 1 | 93.92 | 55.76 | 3.51 | No | No | No | Yes | Yes | 0 | 0 | 0 | 0 | 0 | 0.55 |
| CHEMBL254888 | COc1cccc2c1C(=O)c1c(C2=O)cccc1O | 254.24 | 1 | 4 | 1 | 68.26 | 63.6 | 2.36 | Yes | No | No | No | Yes | 0 | 0 | 0 | 0 | 0 | 0.55 |
| CHEMBL2312529 | CC(C(O)C)O | 90.12 | 1 | 2 | 2 | 23.67 | 40.46 | -0.09 | No | No | No | No | No | 0 | 3 | 0 | 0 | 2 | 0.55 |
| CHEMBL105 | CO[C@@]12[C@H]3N[C@H]3CN2C2=C([C@H]1COC(=O)N)C(=O)C(=C(C2=O)C)N | 334.33 | 4 | 6 | 3 | 86.95 | 146.89 | -0.8 | No | No | No | No | No | 0 | 1 | 1 | 1 | 0 | 0.55 |
