# Supplementary material for: In Silico Identification and Analysis of Potentially Bioactive Antiviral Phytochemicals against SARS-CoV-2: A Molecular Docking and Dynamics Simulation Approach
Source: Biomed Res Int. 2023 May 11;2023:5469258. doi: 10.1155/2023/5469258 (PMC10195178; doi:10.1155/2023/5469258)
Supplement: Supplementary 2 — Supplementary Table 2: ADMET properties of the selected phytochemicals. [file 5469258.f2.docx]

**Supplementary table2** ADMET properties of the selected phytochemicals

| **Chemblmid** | **Canonical Smiles** | **Molecular Weight** | **LogP** | **#Rotatable Bonds** | **#Acceptors** | **#Donors** | **Absorption Caco2 permeability** | **Absorption Intestinal absorption (human)** | **Absorption P-glycoprotein substrate** | **Absorption P-glycoprotein I inhibitor** | **Absorption P-glycoprotein II inhibitor** | **Metabolism CYP2D6 substrate** | **Metabolism CYP3A4 substrate** | **Metabolism CYP1A2 inhibitior** | **Metabolism CYP2C19 inhibitior** | **Metabolism CYP2C9 inhibitior** | **Metabolism CYP2D6 inhibitior** | **Metabolism CYP3A4 inhibitior** | **Excretion Total Clearance** | **Toxicity AMES toxicity** | **Toxicity Max. tolerated dose (human)** | **Toxicity hERG I inhibitor** | **Toxicity hERG II inhibitor** | **Toxicity Hepatotoxicity** |
| --- | --- | --- | --- | --- | --- | --- | --- | --- | --- | --- | --- | --- | --- | --- | --- | --- | --- | --- | --- | --- | --- | --- | --- | --- |
| CHEMBL13045 | COc1c(OC)ccc2c1c[n+](C)c1c2ccc2c1cc1OCOc1c2 | 348.378 | 3.7166 | 2 | 4 | 0 | 0.825 | 100 | No | No | Yes | No | Yes | Yes | Yes | No | Yes | No | 1.044 | Yes | 0.232 | No | Yes | Yes |
| CHEMBL459615 | OC[C@@H](C#CC#CC#CC#CCCCCCCCC(=O)O)O | 300.354 | 1.1685 | 8 | 3 | 3 | 0.926 | 47.06 | No | No | No | No | Yes | No | No | No | No | No | 1.715 | No | -0.958 | No | No | No |
| CHEMBL1080979 | COc1ccc2c(c1)O[C@@H](CC2)c1ccc(cc1)O | 256.301 | 3.4671 | 2 | 3 | 1 | 1.821 | 93.165 | No | No | No | No | Yes | Yes | Yes | Yes | No | No | 0.12 | No | 0.078 | No | No | No |
| CHEMBL458391 | C[C@@H]([C@H](c1c(O)c2oc3cc(=O)c(c4c3c(c2c(c1C)O)O[C@@H]([C@H]4C)C)C)C)O | 398.455 | 4.29454 | 2 | 6 | 3 | 0.737 | 88.709 | Yes | Yes | Yes | No | Yes | No | No | No | No | No | 0.354 | No | -0.555 | No | No | No |
| CHEMBL462951 | CN1CCc2c3C1=CC(=O)C(=O)c3[nH]c2 | 202.213 | 0.6089 | 0 | 3 | 1 | 1.196 | 85.882 | No | No | No | No | No | No | No | No | No | No | 0.537 | No | 0.608 | No | No | No |
| CHEMBL2071229 | COc1ccc(c2c1cc(C)cc2O)OC | 218.252 | 2.87102 | 2 | 3 | 1 | 1.506 | 93.126 | Yes | No | No | No | No | Yes | Yes | No | No | No | 0.605 | No | 0.511 | No | No | No |
| CHEMBL1904496 | C1Oc2c(O1)cc(cc2)C1OCC2C1COC2c1ccc2c(c1)OCO2 | 354.358 | 3.2192 | 2 | 6 | 0 | 1.169 | 98.218 | No | Yes | No | No | Yes | Yes | Yes | Yes | Yes | Yes | -0.096 | Yes | 0.372 | No | No | No |
| CHEMBL1651023 | CCO[C@@H]1OC[C@@]2([C@H]3[C@]1(CCC2)c1cc(O)c(cc1C(=O)C3)C(C)C)C | 358.478 | 4.539 | 3 | 4 | 1 | 1.593 | 95.625 | No | Yes | Yes | No | Yes | Yes | No | No | No | No | 0.59 | No | -0.684 | No | No | No |
| CHEMBL465475 | CO[C@@]12CC[C@]3([C@@](C1)(C(=O)C2=C)CC[C@H]1[C@]23CCC[C@]1(C(=O)O2)C)C | 344.451 | 3.5829 | 1 | 4 | 0 | 1.275 | 100 | No | Yes | No | No | Yes | No | No | No | No | No | 0.502 | No | -0.423 | No | No | No |
| CHEMBL517700 | COc1ccc(c(c1)O)c1coc2c(c1=O)cc(c(c2)O)OC | 314.293 | 2.8884 | 3 | 6 | 2 | 1.144 | 94.623 | Yes | No | No | No | Yes | Yes | Yes | Yes | No | No | 0.638 | No | -0.086 | No | No | No |
| CHEMBL2334478 | O[C@H]1C[C@@H]2C[C@]3([C@@H]1[C@]1(C)CC[C@@H](C([C@H]1C(=O)C3)(C)C)O)[C@@H](C2=C)O | 334.456 | 2.0668 | 0 | 4 | 3 | 0.919 | 91.66 | No | No | No | No | Yes | No | No | No | No | No | 0.622 | No | -0.276 | No | No | No |
| CHEMBL463558 | CO[C@@H]1O[C@@H](c2c1c([C@H](C(=O)O)C)c(cc2)[C@@]1(C)CCCC(C1)(C)C)OC | 376.493 | 5.0528 | 5 | 4 | 1 | 1.418 | 95.796 | Yes | No | No | No | No | No | No | No | No | No | 0.572 | No | -0.058 | No | No | No |
| CHEMBL1651069 | CO[C@@H]1OC[C@@]2([C@H]3[C@]1(CCC2)c1cc(O)c(cc1CC3)C(C)C)C | 330.468 | 4.5087 | 2 | 3 | 1 | 0.926 | 96.563 | No | Yes | No | No | Yes | No | No | No | No | No | 0.651 | No | -0.762 | No | No | No |
| CHEMBL517308 | C/C=C(\C(=O)O[C@H]1[C@H](O)[C@H]2C(=C)C(=O)O[C@@H]2[C@@]2(C(=C1C)C(=O)C=C2C)O)/C | 374.389 | 0.9133 | 2 | 7 | 2 | 0.577 | 85.884 | Yes | No | No | No | No | No | No | No | No | No | 1.353 | No | -0.5 | No | No | No |
| CHEMBL3287734 | O[C@@H]1CCc2c([C@@]1(C)O)ccc1c2C(=O)C(=O)c2c1occ2C | 312.321 | 2.14862 | 0 | 5 | 2 | 1.32 | 96.703 | Yes | No | No | No | Yes | Yes | Yes | No | No | No | 0.148 | No | -0.131 | No | Yes | No |
| CHEMBL575533 | COC1=C[C@@H]2CCN3[C@@H]2[C@@H]([C@@H]1O)c1cc2OCOc2cc1C3 | 301.342 | 1.6078 | 1 | 5 | 1 | 1.241 | 96.755 | Yes | No | No | No | Yes | No | No | No | No | No | 0.968 | No | -0.301 | No | No | No |
| CHEMBL512443 | COc1c(O)cc2c3c1c1ccccc1cc3n(c2=O)C | 279.295 | 3.2972 | 1 | 3 | 1 | 1.294 | 96.205 | Yes | No | Yes | No | Yes | Yes | Yes | No | No | No | 0.196 | Yes | 0.12 | No | Yes | No |
| CHEMBL513565 | COc1cc(ccc1O)C[C@H]1C(=O)OC[C@@H]1[C@@H](c1ccc(c(c1)OC)O)O | 374.389 | 2.1803 | 6 | 7 | 3 | -0.163 | 78.707 | Yes | Yes | Yes | No | Yes | No | No | No | No | Yes | 0.099 | No | -0.311 | No | Yes | No |
| CHEMBL2023564 | CC(=CCC[C@@]([C@H]1CCC(=CC1)C)(O[C@@H]1O[C@H](C)[C@@H]([C@@H]([C@H]1O)O)O)C)C | 368.514 | 3.082 | 6 | 5 | 3 | 0.888 | 93.783 | Yes | No | No | No | No | No | No | No | No | No | 1.407 | No | 0.595 | No | No | No |
| CHEMBL449905 | NC1=CC2=NCCc3c2c(C1=O)n(C)c3 | 201.229 | 0.4092 | 0 | 4 | 1 | 1.179 | 86.342 | No | No | No | No | No | No | No | No | No | No | 0.545 | Yes | 0.413 | No | No | Yes |
| CHEMBL453563 | O=C(C(O)(C)C)CCc1c(C)ccc2c1C(=O)C(=O)C(=C2)C(C)C | 328.408 | 3.07242 | 5 | 4 | 1 | 1.102 | 98.039 | Yes | Yes | No | No | Yes | No | Yes | Yes | No | No | 1.217 | No | -0.034 | No | No | No |
| CHEMBL3590541 | O[C@@H]1C=CC(=O)N2[C@H]1[C@H]1CCCN3[C@H]1[C@H](C2)CCC3 | 262.353 | 0.6185 | 0 | 3 | 1 | 1.181 | 77.748 | No | No | No | No | No | No | No | No | No | No | 0.674 | No | 0.235 | No | No | No |
| CHEMBL1170899 | O=Cc1c(C=O)cc(c2c1O[C@]1(C2)[C@H](C)CC[C@@H]2[C@]1(C)CC[C@H](C2(C)C)O)O | 386.488 | 3.9242 | 2 | 5 | 2 | 1.052 | 94.531 | Yes | Yes | Yes | No | Yes | No | No | No | No | No | 0.394 | No | -0.218 | No | No | No |
| CHEMBL2332142 | COc1ccc2c(c1)N(C)C(=O)[C@]12C[C@@H]2N[C@H]1C[C@@H]1[C@H]2COC=C1C(=O)C | 368.433 | 1.7791 | 2 | 5 | 1 | 1.089 | 97.57 | Yes | Yes | No | No | Yes | No | No | No | No | No | 0.919 | No | -0.705 | No | Yes | Yes |
| CHEMBL484231 | CC1=C[C@H]2OC3[C@@]4([C@H]1CC(=O)c1cocc1)[C@@H]2[C@](C)(CCC4)C(=O)O3 | 342.391 | 3.503 | 3 | 5 | 0 | 1.274 | 99.458 | No | Yes | No | No | Yes | No | Yes | No | No | No | 0.874 | No | 0.257 | No | No | Yes |
| CHEMBL172350 | COc1c(O)cc2c(c1O)c(=O)cc(o2)c1ccc(c(c1)O)O | 316.265 | 2.291 | 2 | 7 | 4 | 0.487 | 78.295 | Yes | No | No | No | No | Yes | No | No | No | No | 0.6 | Yes | 0.633 | No | Yes | No |
| CHEMBL2385627 | C=C[C@@]1(C)C[C@@H](O)[C@H]2C(=C1)CC[C@@H]1[C@]2(C)CCC[C@@]1(C)COC(=O)C | 346.511 | 4.6555 | 3 | 3 | 1 | 1.412 | 96.516 | Yes | Yes | No | No | Yes | No | No | No | No | No | 0.766 | No | -0.889 | No | No | No |
| CHEMBL451784 | CC(=O)O[C@H]1CC[C@H]([C@@]23[C@]1(C)[C@H](C[C@H]([C@H]3O)C(O2)(C)C)OC(=O)c1ccccc1)C | 416.514 | 3.5083 | 3 | 6 | 1 | 1.346 | 96.454 | No | Yes | Yes | No | Yes | No | No | No | No | Yes | 0.784 | No | -0.178 | No | No | No |
| CHEMBL483030 | COc1cc(O)c2c(c1)oc(c(c2=O)OC)c1cc(O)c(cc1OC)OC | 374.345 | 2.9056 | 5 | 8 | 2 | 0.756 | 94.879 | Yes | No | Yes | No | No | Yes | Yes | No | No | Yes | 0.782 | No | 0.817 | No | Yes | No |
| CHEMBL513024 | COc1cc2c(cc1O)occ(c2=O)c1ccc(cc1)O | 284.267 | 2.8798 | 2 | 5 | 2 | 1.133 | 94.034 | Yes | No | No | No | Yes | Yes | Yes | Yes | No | Yes | 0.196 | No | 0.002 | No | No | No |
| CHEMBL504766 | COc1cc(OC)cc2c1c(O)c1c(c2)oc(c(c1=O)C)C | 300.31 | 3.28584 | 2 | 5 | 1 | 1.141 | 97.486 | No | Yes | Yes | No | No | Yes | Yes | No | No | No | 0.711 | Yes | 0.473 | No | Yes | No |
| CHEMBL1345092 | C/C=C(\C(=O)O[C@@H]1[C@H](OC(=O)C=C(C)C)c2c(OC1(C)C)ccc1c2oc(=O)cc1)/C | 426.465 | 4.3925 | 4 | 7 | 0 | 0.998 | 98.226 | No | Yes | No | No | Yes | No | No | No | No | Yes | 0.983 | No | 0.68 | No | No | Yes |
| CHEMBL2057716 | OCC1=C2C(=C3CC(=O)[C@@H]([C@]3(CC[C@@]2(CC[C@@H]1O)C)C)C(C)C)O | 334.456 | 3.2935 | 2 | 4 | 3 | 1.021 | 92.09 | Yes | Yes | No | No | Yes | No | No | No | No | No | 0.751 | No | -0.152 | No | No | No |
| CHEMBL3754295 | O=C1C2=C(O[C@]3([C@@H](C2)[C@@H](CC3)C(O)(C)C)C)[C@@H]2C[C@@]1(O)CO2 | 308.374 | 1.3193 | 1 | 5 | 2 | 0.587 | 71.262 | No | No | No | No | No | No | No | No | No | No | 0.814 | Yes | 0.157 | No | No | No |
| CHEMBL3577255 | C=C[C@@](/C=C/c1ccc(cc1)O)(CC[C@H](C(O)(C)C)O)C | 290.403 | 3.5097 | 7 | 3 | 3 | 1.342 | 88.596 | Yes | No | No | No | Yes | Yes | Yes | No | No | No | 1.143 | No | 0.698 | No | Yes | No |
| CHEMBL471071 | C/C/1=C/C(=O)O[C@@H]2C[C@@H](CC[C@@H](C=CCC1)C)O[C@@](C2)(O)[C@H]1CSC(=N1)O | 395.521 | 3.5078 | 1 | 6 | 2 | 0.982 | 92.218 | Yes | No | No | No | No | No | No | No | No | No | 0.172 | No | -0.092 | No | No | Yes |
| CHEMBL1079685 | C=C1CC[C@@H]([C@@]2([C@@H]1C[C@H](CC2)C(O)(C)C)C)O | 238.371 | 2.8908 | 1 | 2 | 2 | 1.599 | 93.482 | No | No | No | No | No | No | No | No | No | No | 1.082 | No | 0.169 | No | No | No |
| CHEMBL511568 | CO[C@@H]1OC[C@]2([C@@H]1[C@]1(CCC2)COC(=O)[C@]23[C@H]1CC[C@H](C2)[C@@H](C3=O)C)C | 362.466 | 2.9602 | 1 | 5 | 0 | 1.601 | 100 | No | Yes | No | No | Yes | No | No | No | No | No | 0.451 | No | 0.235 | No | No | No |
| CHEMBL480873 | N=c1[nH]cc(c2c1cncc2)c1ccccc1 | 221.263 | 2.70937 | 1 | 2 | 2 | 1.307 | 92.766 | Yes | No | No | No | Yes | Yes | Yes | No | No | No | 0.573 | Yes | -0.114 | No | No | No |
| CHEMBL480677 | O[C@H]1C[C@H]2[C@]3(C)[C@@H](O)CCC([C@H]3C[C@H]([C@]32[C@@H]([C@@H]1C(=C)C3=O)O)O)(C)C | 350.455 | 1.0376 | 0 | 5 | 4 | 0.474 | 73.817 | Yes | No | No | No | No | No | No | No | No | No | 0.697 | No | -0.124 | No | No | No |
| CHEMBL1087720 | COc1ccc(cc1)c1cc(=O)c2c(o1)cc(cc2OC)OC | 312.321 | 3.4858 | 4 | 5 | 0 | 1.392 | 98.133 | No | Yes | Yes | No | Yes | Yes | Yes | Yes | No | No | 0.819 | No | 0.281 | No | No | No |
| CHEMBL464176 | C=C[C@](CC[C@H]1[C@](C)(O)CC[C@@H]2[C@]1(C)CCCC2(C)C)(O)C | 308.506 | 4.6972 | 4 | 2 | 2 | 1.77 | 93.739 | No | Yes | No | No | Yes | No | No | No | No | No | 0.914 | No | -0.225 | No | No | No |
| CHEMBL572482 | COc1c2c(CC=C(C)C)c3OCOc3c(c2nc2c1cco2)O | 327.336 | 3.9326 | 3 | 6 | 1 | 1.257 | 95.85 | No | Yes | No | No | Yes | Yes | Yes | Yes | No | No | 0.468 | No | -0.077 | No | Yes | Yes |
| CHEMBL1957109 | COc1ccc2c(c1)[nH]cc2C(=O)c1nccc2c1[nH]c1c2ccc(c1)OC | 371.396 | 4.4456 | 4 | 4 | 2 | 1.191 | 94.496 | Yes | No | Yes | No | Yes | Yes | Yes | Yes | Yes | Yes | 0.782 | Yes | 0.47 | No | Yes | Yes |
| CHEMBL496643 | COc1ccccc1c1coc2c(c1=O)c(O)c(c(c2)O)C | 298.294 | 3.18822 | 2 | 5 | 2 | 1.022 | 95.949 | Yes | No | Yes | No | No | Yes | Yes | Yes | Yes | Yes | 0.402 | No | 0.559 | No | Yes | No |
| CHEMBL550232 | COc1cc2c(cc1O)O[C@@H]1[C@H]2COc2c1cc1c(c2)OC([C@@H](C1)O)(C)C | 370.401 | 3.0849 | 1 | 6 | 2 | 1.246 | 94.138 | Yes | Yes | No | No | Yes | No | Yes | Yes | No | Yes | 0.218 | No | -0.93 | No | Yes | No |
| CHEMBL520763 | OC[C@@]12O[C@@]34O[C@@H]1CC[C@]2(C)[C@@]3(C)CC(=O)C(=C4)C | 264.321 | 1.5683 | 1 | 4 | 1 | 1.22 | 96.64 | No | No | No | No | No | No | No | No | No | No | 0.953 | No | 0.267 | No | No | No |
| CHEMBL1078266 | C#C[C@@H](COc1ccc(cc1)COC(=O)C)O | 234.251 | 1.1226 | 5 | 4 | 1 | 1.231 | 85.062 | No | No | No | No | No | No | No | No | No | No | 0.494 | No | 1.08 | No | No | No |
| CHEMBL3088138 | CO[C@@H]1OC[C@@H]([C@H]1Cc1ccc(c(c1)OC)O)[C@@H](c1ccc(c(c1)OC)O)O | 390.432 | 2.6262 | 7 | 7 | 3 | -0.094 | 75.083 | Yes | Yes | Yes | No | Yes | No | Yes | No | No | Yes | 0.212 | No | -0.125 | No | No | No |
| CHEMBL477855 | COC1=CC(=O)C=C(C1(O)CC(=O)C)OC | 226.228 | 0.3399 | 4 | 5 | 1 | 0.775 | 92.922 | No | No | No | No | No | No | No | No | No | No | 0.739 | No | 0.82 | No | No | No |
| CHEMBL446725 | COc1c(CC=C(C)C)c2OC(C)(C)C=Cc2c(c1C(=O)C)O | 316.397 | 4.2963 | 4 | 4 | 1 | 1.428 | 93.53 | No | Yes | No | No | No | No | Yes | No | No | No | 0.854 | No | 0.578 | No | No | No |
| CHEMBL519603 | COc1cc(cc(c1OC)OC)[C@H]1[C@@H]2C(=O)OC[C@@H]2Cc2c1cc1OCOc1c2 | 398.411 | 2.9183 | 4 | 7 | 0 | 1.304 | 100 | No | Yes | Yes | No | Yes | No | No | Yes | No | Yes | 0.24 | Yes | 0.03 | No | No | No |
| CHEMBL212947 | COc1c2C(=O)c3ccccc3C(=O)c2c(c(c1C=O)O)O | 298.25 | 1.6943 | 2 | 6 | 2 | 0.128 | 91.659 | Yes | No | No | No | No | No | No | No | No | No | -0.022 | Yes | 0.572 | No | No | No |
| CHEMBL479911 | O[C@]12CO[C@@H]([C@H]1CO[C@@H]2c1ccc2c(c1)OCO2)c1ccc2c(c1)OCO2 | 370.357 | 2.3341 | 2 | 7 | 1 | 1.431 | 94.655 | No | Yes | No | No | Yes | No | No | No | No | Yes | -0.181 | Yes | -0.021 | No | No | No |
| CHEMBL165509 | COc1c(oc2c(c1=O)c(O)c(c(c2)OC)OC)c1ccc(cc1)O | 344.319 | 2.897 | 4 | 7 | 2 | 0.086 | 94.079 | Yes | No | Yes | No | No | Yes | Yes | Yes | No | No | 0.637 | No | 0.419 | No | Yes | No |
| CHEMBL3093767 | C=C1[C@@H]2CC[C@@H]3[C@](C1=O)(C2)C(=O)O[C@@H]1[C@]23CO[C@H]([C@@H]2C(CC1)(C)C)O | 346.423 | 2.2246 | 0 | 5 | 1 | 1.336 | 99.073 | No | Yes | No | No | Yes | No | No | No | No | No | 0.41 | No | -0.143 | No | No | No |
| CHEMBL1783110 | CC(=CCOc1cc(C)c2c(c1)oc(=O)cc2OCC=C(C)C)C | 328.408 | 4.79142 | 6 | 4 | 0 | 1.478 | 97.266 | No | Yes | No | No | Yes | Yes | Yes | Yes | No | No | 0.976 | No | 0.695 | No | No | Yes |
| CHEMBL3314602 | Oc1ccc2c(c1)c(=O)c1c(o2)cc2c(c1O)C=CC(O2)(C)C | 310.305 | 3.5416 | 0 | 5 | 2 | 1.071 | 92.108 | Yes | No | Yes | No | Yes | Yes | Yes | Yes | No | No | 0.429 | Yes | -0.288 | No | No | No |
| CHEMBL482243 | COc1cc2c(cc1O)oc1c(c2=O)c(O)cc(c1)O | 274.228 | 2.0716 | 1 | 6 | 3 | 0.16 | 85.244 | Yes | No | No | No | No | Yes | No | No | No | No | 0.592 | Yes | 0.492 | No | No | No |
| CHEMBL465364 | COc1cc(O)c(c(c1OC)OC)C(=O)/C=C/c1ccc(c(c1)O)O | 346.335 | 2.7253 | 6 | 7 | 3 | 0.019 | 80.632 | Yes | No | No | No | No | No | Yes | No | No | No | 0.118 | No | -0.019 | No | Yes | No |
| CHEMBL1079408 | COc1cc2c(cc1O)O[C@@H]1[C@H]2COc2c1cc(c(c2)O)CC=C(C)C | 354.402 | 4.2248 | 3 | 5 | 2 | 0.697 | 92.385 | Yes | Yes | Yes | No | Yes | Yes | Yes | Yes | No | Yes | 0.22 | No | -0.524 | No | Yes | No |
| CHEMBL452224 | OC/C=C(\CC[C@H]1C(=C)[C@H](O)C[C@@H]2[C@]1(C)C[C@@H](O)C[C@]2(C)COC(=O)C)/C | 380.525 | 2.9888 | 6 | 5 | 3 | 1.032 | 92.352 | Yes | Yes | No | No | No | No | No | No | No | Yes | 1.067 | No | -0.849 | No | No | No |
| CHEMBL453193 | CCC[C@H](C1=C(Br)[C@](OC1=O)(CI)OC)O | 405.026 | 2.1309 | 5 | 4 | 1 | 1.295 | 93.216 | No | No | No | No | No | No | No | No | No | No | 0.41 | Yes | 0.67 | No | No | No |
| CHEMBL590549 | O=C1C[C@@H]2[C@]([C@@H]3C1=CC(=CC3)C(C)C)(C)CCC[C@@]2(C)C(=O)O | 316.441 | 4.3852 | 2 | 2 | 1 | 1.394 | 99.133 | No | No | No | No | Yes | No | No | No | No | No | 0.914 | No | -0.079 | No | No | Yes |
| CHEMBL474628 | Oc1cc(CCc2ccccc2O)cc(c1)O | 230.263 | 2.5886 | 3 | 3 | 3 | 1.145 | 89.283 | Yes | No | No | No | Yes | Yes | Yes | Yes | No | Yes | 0.155 | Yes | 0.239 | No | No | No |
| CHEMBL491374 | COc1cc(c(cc1Br)Br)[C@H]1[C@@H](O)N(C(=O)[C@@]21CCC=N2)C | 432.112 | 2.6976 | 2 | 4 | 1 | 0.922 | 91.34 | Yes | No | No | No | Yes | No | No | No | No | No | 0.222 | Yes | 0.202 | No | No | Yes |
| CHEMBL461614 | COc1ccc(c(c1)/C=C/Cc1ccc(c(c1OC)OC)O)O | 316.353 | 3.3795 | 6 | 5 | 2 | 0.949 | 91.271 | Yes | No | No | No | Yes | Yes | Yes | Yes | No | Yes | 0.275 | Yes | 0.582 | No | No | No |
| CHEMBL253688 | COc1c(OC)cc2c(c1OC)c1c(cc(c(c1OC)OC)OC)C[C@@H]([C@@](C2)(C)O)C | 432.513 | 3.8909 | 6 | 7 | 1 | 1.384 | 98.689 | No | Yes | Yes | No | Yes | No | Yes | Yes | No | Yes | 0.375 | No | 0.335 | No | No | No |
| CHEMBL459851 | O=C1OC[C@H](C1=Cc1ccc2c(c1)OCO2)Cc1ccc2c(c1)OCO2 | 352.342 | 2.9431 | 3 | 6 | 0 | 0.994 | 98.072 | No | Yes | Yes | No | Yes | Yes | Yes | Yes | No | Yes | 0.213 | No | -0.192 | No | Yes | No |
| CHEMBL2337574 | C=C1[C@H]2CC[C@@H]3[C@](C1=O)([C@@H]2O)CC[C@H]1[C@@]3(C)CCCC1(C)C | 302.458 | 4.1252 | 0 | 2 | 1 | 1.552 | 99.465 | No | Yes | No | No | Yes | No | No | No | No | No | 0.593 | No | -0.497 | No | No | No |
| CHEMBL2270656 | O=C[C@@H]1C[C@@H](O)C[C@H]([C@]21CC[C@H](C2)C(=C)C)C | 236.355 | 2.9549 | 2 | 2 | 1 | 1.554 | 94.488 | No | No | No | No | No | No | No | No | No | No | 1.161 | No | -0.439 | No | No | No |
| CHEMBL1628279 | OC[C@@]12CC[C@@H](C[C@@]2(O)CC[C@@H]2[C@@H]1CC[C@]1([C@]2(O)CC[C@@H]1C1=CC(=O)OC1)C)O | 406.519 | 1.6916 | 2 | 6 | 4 | 0.515 | 89.722 | Yes | No | No | No | Yes | No | No | No | No | No | 0.666 | No | -0.411 | No | No | No |
| CHEMBL1288770 | CC(C1CC[C@@]2([C@@H](C1)[C@](C)(O)CCC2)C)(O)C | 240.387 | 3.1148 | 1 | 2 | 2 | 1.59 | 92.019 | No | No | No | No | No | No | No | No | No | No | 0.934 | No | 0.188 | No | No | No |
| CHEMBL60533 | CO/C=C(\[C@H]1C[C@@H]2N(C[C@H]1CC)CCc1c2[nH]c2c1cccc2)/C(=O)OC | 368.477 | 3.8165 | 4 | 4 | 1 | 1.121 | 92.839 | Yes | Yes | No | Yes | Yes | No | No | No | Yes | No | 0.993 | Yes | -0.483 | No | Yes | Yes |
| CHEMBL1079671 | O[C@H]1C[C@@H](C)C2=CC(=O)[C@@]3([C@@H]([C@]2([C@@H]1O)C)[C@@H](O)[C@@H]1OC(=O)[C@H]3[C@H]1C)C | 350.411 | 0.4381 | 0 | 6 | 3 | 0.47 | 62.558 | Yes | No | No | No | Yes | No | No | No | No | No | 0.712 | Yes | -0.049 | No | No | No |
| CHEMBL1287907 | C[C@H]1Cc2c(O1)c(O)c1c(c2O)C(=O)C[C@@H]2[C@]1(C)CC[C@@H]1[C@@]2(C)C1 | 328.408 | 3.7015 | 0 | 4 | 2 | 1.734 | 95.641 | Yes | No | No | No | Yes | No | No | No | No | No | 0.216 | Yes | -0.518 | No | No | No |
| CHEMBL482040 | C[C@@H]1CCC2=C3C1=COC[C@@]3(C)[C@@H](C2(C)C)O | 234.339 | 3.034 | 0 | 2 | 1 | 1.597 | 94.873 | No | No | No | No | No | No | No | No | No | No | 0.922 | No | 0.29 | No | No | No |
| CHEMBL272485 | CCCCOC(=O)c1ccccc1C(=O)OCCCC | 278.348 | 3.6004 | 8 | 4 | 0 | 1.622 | 95.044 | No | No | No | No | Yes | Yes | Yes | No | No | No | 0.93 | No | 1.536 | No | No | No |
| CHEMBL463255 | COc1cc(O)c2c(c1CC=C(C)C)O[C@@H](CC2)c1ccc(cc1O)O | 356.418 | 4.387 | 4 | 5 | 3 | 0.849 | 90.784 | Yes | Yes | No | No | Yes | Yes | Yes | Yes | No | Yes | 0.401 | No | 0.005 | No | Yes | No |
| CHEMBL1651328 | COC(=O)/C=C/NC(=O)c1nc(C(=O)C)c2c(c1)c1ccccc1[nH]2 | 337.335 | 2.3352 | 4 | 5 | 2 | 0.673 | 82.993 | Yes | Yes | No | No | No | Yes | No | No | No | No | 0.725 | No | 0.169 | No | Yes | Yes |
| CHEMBL464484 | CC(Cc1ncc(o1)c1c[nH]c2c1cccc2)C | 240.306 | 4.0214 | 3 | 2 | 1 | 1.739 | 91.992 | Yes | No | Yes | Yes | Yes | Yes | Yes | No | No | No | 0.999 | Yes | 0.44 | No | No | No |
| CHEMBL447517 | ClC1=C(N)C(=O)c2c3C1=NCCc3cn2C | 235.674 | 0.9757 | 0 | 4 | 1 | 1.234 | 88.747 | No | No | No | No | No | No | No | No | No | No | 0.19 | Yes | 0.412 | No | No | Yes |
| CHEMBL400142 | CC(=O)OC[C@]1(C)CCC[C@@]23[C@@H]1[C@H](O)[C@](O)(OC2)[C@@]12[C@H]3CC[C@H]([C@H]2O)C(=C)C1=O | 406.475 | 0.948 | 2 | 7 | 3 | 0.633 | 73.836 | Yes | No | No | No | No | No | No | No | No | No | 0.539 | No | -0.897 | No | No | No |
| CHEMBL425900 | C=C1C(=O)O[C@H]2[C@@H]1CC1=C([C@H](C2)C)C(=O)C[C@@H]1C | 246.306 | 2.4196 | 0 | 3 | 0 | 1.342 | 99.007 | No | No | No | No | No | No | No | No | No | No | 0.544 | No | 0.317 | No | No | No |
| CHEMBL2208204 | CO[C@H]1C=C[C@]23[C@H](C1)N(C)C(=O)[C@H]2N(C)Cc1c3cc2OCOc2c1 | 342.395 | 1.2826 | 1 | 5 | 0 | 1.256 | 75.44 | Yes | No | No | No | Yes | No | No | No | No | No | 0.897 | No | -0.191 | No | No | Yes |
| CHEMBL252790 | Oc1ccc(cc1)C[C@@H](C(=O)c1ccc(cc1O)O)O | 274.272 | 1.5897 | 4 | 5 | 4 | -0.233 | 65.956 | Yes | No | No | No | No | No | No | No | No | No | 0.193 | No | 0.337 | No | No | No |
| CHEMBL1087283 | C/C(=C/C=C/C=C/C=C/C=C/C(=O)O)/Cl | 224.687 | 3.4384 | 5 | 1 | 1 | 1.606 | 94.447 | No | No | No | No | No | No | No | No | No | No | 0.301 | No | -0.221 | No | No | No |
| CHEMBL2409055 | COc1cc(ccc1O)[C@@H]1CCc2c(O1)cc(c(c2)O)OC | 302.326 | 3.1813 | 3 | 5 | 2 | 1.157 | 92.092 | Yes | No | No | No | Yes | Yes | Yes | Yes | No | No | 0.08 | Yes | -0.436 | No | No | No |
| CHEMBL243089 | CC(=O)Oc1ccc2c(c1)oc(c(c2=O)c1ccccc1)C | 294.306 | 3.69372 | 2 | 4 | 0 | 1.348 | 99.263 | No | No | Yes | No | Yes | Yes | Yes | Yes | No | No | 0.464 | No | 0.367 | No | No | No |
| CHEMBL552509 | OC/C(=C/1\Oc2c(C1=O)c(OC)ccc2)/[C@@H]1OC(=O)C[C@H]1C | 304.298 | 1.4683 | 3 | 6 | 1 | 1.314 | 99.005 | No | No | No | No | Yes | No | No | No | No | No | 0.26 | No | 0.955 | No | No | No |
| CHEMBL1965265 | COc1ccc2c(c1)c(=O)c1c(o2)c(OC)c(c(c1)OC)OC | 316.309 | 2.9806 | 4 | 6 | 0 | 1.191 | 99.298 | No | No | Yes | No | Yes | Yes | Yes | Yes | No | No | 0.699 | Yes | 0.489 | No | No | No |
| CHEMBL467203 | COC1=C(c2ccccc2)C(=O)C(=C(C1=O)c1ccccc1)OC | 320.344 | 3.2536 | 4 | 4 | 0 | 1.394 | 99.335 | No | Yes | No | No | Yes | No | Yes | Yes | No | No | 0.037 | Yes | 0.57 | No | No | No |
| CHEMBL511511 | COc1c(CC=C(C)C)c(O)c(c(c1C(=O)C)O)C=O | 278.304 | 2.6302 | 5 | 5 | 2 | 1.341 | 93.837 | Yes | No | No | No | No | No | No | No | No | No | 0.257 | No | 0.834 | No | No | No |
| CHEMBL597425 | OCC1(C)CCc2c(O1)cc1c(c2OC)cc(c(=O)o1)c1ccc(cc1O)O | 384.384 | 2.9558 | 3 | 7 | 3 | 0.349 | 82.305 | Yes | No | Yes | No | Yes | Yes | Yes | Yes | No | No | 0.663 | No | 0.219 | No | Yes | No |
| CHEMBL513108 | NCCC1=C(SC)C(=O)c2c3c1nc1ccccc1c3ccn2 | 321.405 | 3.4022 | 3 | 5 | 1 | 1.208 | 96.132 | No | Yes | Yes | No | Yes | Yes | No | No | No | No | 0.962 | No | -0.204 | No | No | Yes |
| CHEMBL254599 | COc1ccc2c(c1O)CCc1c2c(OC)c(c(c1)O)OC | 302.326 | 2.8892 | 3 | 5 | 2 | 1.087 | 96.081 | Yes | No | No | No | Yes | Yes | Yes | Yes | No | Yes | 0.467 | Yes | -0.184 | No | Yes | No |
| CHEMBL1096781 | CC(=O)O[C@H]1CC[C@@](C)(O)[C@@H]2O[C@H](CC1=C)[C@@H]1[C@H]2[C@H](CCC1=C)C(C)C | 362.51 | 4.0312 | 2 | 4 | 1 | 1.415 | 95.926 | Yes | Yes | Yes | No | Yes | No | No | No | No | No | 0.897 | No | -0.316 | No | No | No |
| CHEMBL463922 | CO[C@@H]1OC[C@]2([C@@H]1[C@]1(CCC2)COC(=O)[C@]23[C@H]1[C@H](O)C[C@H](C2)C(=C)C3=O)C | 376.449 | 1.8511 | 1 | 6 | 1 | 1.595 | 100 | Yes | Yes | No | No | Yes | No | No | No | No | No | 0.498 | No | -0.255 | No | No | No |
| CHEMBL141117 | COc1cc(cc(c1O)OC)[C@H]1[C@H]2C(=O)OCC2Cc2c1cc1OCOc1c2 | 384.384 | 2.6153 | 3 | 7 | 1 | 1.345 | 99.145 | Yes | Yes | Yes | No | Yes | No | Yes | No | No | Yes | 0.18 | No | -0.579 | No | Yes | No |
| CHEMBL325752 | COc1c(ccc2c1C(=O)OCc1c(O2)c(O)cc(c1)C)[C@H](CC(C)C)O | 372.417 | 4.25142 | 4 | 6 | 2 | 1.331 | 93.657 | Yes | Yes | Yes | No | Yes | No | Yes | Yes | No | Yes | 0.421 | No | 0.003 | No | Yes | No |
| CHEMBL25308 | OC/C=C(/CC/C=C(/CCC=C(C)C)\C)\C | 222.372 | 4.3979 | 7 | 1 | 1 | 1.495 | 91.531 | No | No | No | No | No | No | No | No | No | No | 1.754 | No | 0.096 | No | No | No |
| CHEMBL464885 | COc1cc2c(cc1OC)OC[C@@H]1[C@H]2C(=O)c2c(O1)c1c(cc2)OC([C@@H]([C@H]1O)O)(C)C | 428.437 | 2.389 | 2 | 8 | 2 | 0.797 | 86.774 | Yes | Yes | No | No | Yes | No | No | No | No | Yes | 0.242 | No | 0.127 | No | No | No |
| CHEMBL2023567 | CC(=CC[C@]12O[C@H]1C(=O)c1c(C2=O)c(O)ccc1)C | 258.273 | 2.2651 | 2 | 4 | 1 | 1.143 | 95.772 | No | No | No | No | No | No | No | No | No | No | 0.153 | Yes | 0.746 | No | No | No |
| CHEMBL165064 | COc1c(oc2c(c1=O)c(O)cc(c2)O)c1ccc(cc1)O | 300.266 | 2.5854 | 2 | 6 | 3 | 0.09 | 92.496 | Yes | No | No | No | No | Yes | No | Yes | No | No | 0.619 | No | 0.592 | No | Yes | No |
| CHEMBL497275 | C/C=C(\C(=O)OCc1nccc2c1C(=O)C(=C(C2=O)C)NC)/C | 314.341 | 1.9634 | 4 | 6 | 1 | 1.158 | 96.017 | No | No | No | No | No | No | No | No | No | No | 0.663 | No | 0.68 | No | No | Yes |
| CHEMBL3401056 | COc1c(ccc2c1C=CC(O2)(C)C)C(=O)/C=C(/c1ccccc1)\O | 336.387 | 4.6612 | 4 | 4 | 1 | 1.404 | 93.23 | No | Yes | Yes | No | Yes | Yes | Yes | Yes | No | No | 0.15 | No | -0.107 | No | Yes | No |
| CHEMBL462956 | COc1ccc2c(c1O)[C@H](Cc1ccc(cc1)O)N(CC2)C | 299.37 | 2.8781 | 3 | 4 | 2 | 1.201 | 90.939 | Yes | No | No | No | Yes | Yes | No | No | Yes | No | 0.939 | Yes | -0.252 | No | Yes | Yes |
| CHEMBL1951300 | CC(=CCc1c(O)ccc2c1O[C@@H](CC2)c1ccc(cc1O)O)C | 326.392 | 4.3784 | 3 | 4 | 3 | 0.735 | 91.091 | Yes | No | No | No | No | Yes | Yes | Yes | No | Yes | 0.24 | No | -0.006 | No | Yes | No |
| CHEMBL485477 | COc1cc2[C@H]3O[C@H]([C@@H]([C@@H]3C)C)c3c(c2c(c1OC)OC)c(OC)c1c(c3)OCO1 | 414.454 | 4.5149 | 4 | 7 | 0 | 1.45 | 99.759 | No | Yes | No | No | Yes | No | Yes | Yes | No | Yes | 0.073 | No | 0.388 | No | No | No |
| CHEMBL465959 | OCC(=O)[C@]1(C)CC[C@H]2C(=C1)CC[C@@H]1[C@]2(C)C=C(O)C(=O)C1(C)C | 332.44 | 3.3576 | 2 | 4 | 2 | 1.061 | 93.333 | No | Yes | No | No | Yes | No | No | No | No | No | 0.62 | Yes | -0.16 | No | No | No |
| CHEMBL515346 | OC[C@]12CC[C@@H](C[C@H]1CC[C@@H]1[C@@H]2CC[C@]2([C@]31O[C@@H]3C[C@@H]2c1ccc(=O)oc1)C)O | 400.515 | 3.2307 | 2 | 5 | 2 | 0.989 | 96.9 | No | Yes | No | No | Yes | No | No | Yes | No | Yes | 0.497 | No | -0.518 | No | No | Yes |
| CHEMBL477533 | COC1=C(OC)C(=O)c2c(C1=O)c(O)cc(c2O)OC | 280.232 | 0.9898 | 3 | 7 | 2 | 0.522 | 48.008 | No | No | No | No | No | No | No | No | No | No | 0.59 | No | 0.414 | No | No | No |
| CHEMBL3600945 | C/C=C(/C(=O)C[C@H]1[C@](C)(O)CC[C@@]2([C@]1(C)[C@@H](O)CCC2(C)C)O)\C | 338.488 | 2.9911 | 3 | 4 | 3 | 0.977 | 95.477 | Yes | No | No | No | No | No | No | No | No | No | 0.985 | No | 0.114 | No | No | No |
| CHEMBL53418 | Oc1cccc2c1C(=O)c1c(C2=O)cccc1O | 240.214 | 1.8732 | 0 | 4 | 2 | 1.128 | 95.901 | Yes | No | No | No | No | Yes | No | No | No | Yes | 0.015 | Yes | 0.194 | No | No | No |
| CHEMBL1172613 | CC[C@H]([C@H]1O[C@H]([C@@H](C[C@@H]1C)C)c1c(O)c(cn(c1=O)OC)c1ccccc1)C | 385.504 | 4.4276 | 5 | 5 | 1 | 1.259 | 94.223 | No | Yes | Yes | No | Yes | No | Yes | Yes | No | Yes | 1.161 | No | 0.391 | No | Yes | Yes |
| CHEMBL487724 | CO[C@@H]1CC(=O)c2c3[C@]41Oc1ccc(c5c1[C@@](O4)(Oc3ccc2)[C@@H]1O[C@@H]1[C@@H]5O)O | 396.351 | 1.6152 | 1 | 8 | 2 | 0.406 | 87.868 | Yes | No | No | No | Yes | No | No | No | No | No | 0.032 | No | -0.302 | No | No | Yes |
| CHEMBL514518 | CC1=CCC[C@@](C)(O)[C@H]2C[C@H](CC[C@]([C@@H](CC1)O)(C)O)[C@H](C(=O)O2)C | 354.487 | 2.7176 | 0 | 5 | 3 | 1.123 | 93.553 | No | No | No | No | Yes | No | No | No | No | No | 1.128 | No | 0.327 | No | No | No |
| CHEMBL521975 | COc1cc2[C@@H](O)[C@@H](C)[C@@H](C)Cc3c(c2c(c1OC)OC)c(OC)c1c(c3)OCO1 | 416.47 | 3.9784 | 4 | 7 | 1 | 1.254 | 99.894 | No | Yes | No | No | Yes | No | Yes | Yes | No | Yes | 0.135 | No | 0.239 | No | Yes | No |
| CHEMBL2419311 | CC(=CC(=Nc1cc2c3n1c1ccccc1c1c3c(c(c2)O)ncc1)O)C | 355.397 | 5.4189 | 2 | 4 | 2 | 0.721 | 93.694 | Yes | Yes | Yes | Yes | Yes | Yes | Yes | Yes | No | No | 0.069 | Yes | 0.526 | No | Yes | No |
| CHEMBL1079510 | C=C1CCC(=O)[C@@]2([C@@H]1C[C@H](CC2)C(O)(C)C)C | 236.355 | 3.099 | 1 | 2 | 1 | 1.195 | 94.644 | No | No | No | No | No | No | No | No | No | No | 1.099 | No | 0.236 | No | No | No |
| CHEMBL385256 | COc1cc(O)cc2c1c1ccc(cc1C2=O)O | 242.23 | 2.3178 | 1 | 4 | 2 | 1.021 | 94.877 | Yes | No | No | No | No | Yes | No | No | No | No | 0.484 | Yes | 0.369 | No | No | No |
| CHEMBL464202 | COc1ccc2c(c1C)O[C@@H]([C@@H](C2)O)c1ccc(cc1)O | 286.327 | 2.74632 | 2 | 4 | 2 | 1.225 | 94.474 | Yes | No | No | No | Yes | Yes | Yes | Yes | No | No | 0.201 | Yes | -0.525 | No | Yes | No |
| CHEMBL462719 | COc1cc(C/C=C/c2ccc(c(c2OC)O)OC)ccc1O | 316.353 | 3.3795 | 6 | 5 | 2 | 1.094 | 91.175 | Yes | Yes | No | No | Yes | Yes | Yes | Yes | No | Yes | 0.184 | No | 0.357 | No | No | No |
| CHEMBL452234 | COc1c(C(=O)CCc2ccccc2)c(O)c(c(c1C)O)C | 300.354 | 3.53874 | 5 | 4 | 2 | 0.874 | 94.285 | Yes | No | No | No | No | Yes | Yes | Yes | No | Yes | 0.381 | Yes | 0.764 | No | Yes | No |
| CHEMBL1097683 | COc1c(oc2c(c1=O)c(O)cc(c2)OC)c1ccccc1 | 298.294 | 3.1828 | 3 | 5 | 1 | 1.046 | 96.841 | No | No | Yes | No | No | Yes | Yes | No | No | No | 0.447 | Yes | 0.554 | No | Yes | No |
| CHEMBL2409150 | CCC[C@H]1O[C@H](CC(=O)OC)[C@@H](C2=C1C(=O)c1c(C2=O)cccc1O)O | 360.362 | 1.5593 | 4 | 7 | 2 | 0.762 | 74.606 | Yes | No | No | No | No | No | No | No | No | No | 1.057 | No | -0.05 | No | No | No |
| CHEMBL1669578 | O=C(N1CCCC1)/C=C/C=C/CC/C=C/c1ccc2c(c1)OCO2 | 325.408 | 3.9435 | 6 | 3 | 0 | 1.894 | 93.876 | Yes | Yes | No | No | Yes | No | Yes | Yes | No | No | 0.308 | No | -0.669 | No | No | No |
| CHEMBL2331810 | COc1c(c(O)cc(c1O)c1ccccc1)c1ccccc1 | 292.334 | 4.4404 | 3 | 3 | 2 | 1.318 | 95.563 | Yes | No | Yes | No | Yes | Yes | Yes | Yes | No | No | 0.291 | Yes | 0.35 | No | Yes | Yes |
| CHEMBL2409054 | COc1cc2O[C@@H](CCc2cc1OC)c1ccc(c(c1)OC)O | 316.353 | 3.4843 | 4 | 5 | 1 | 1.338 | 94.565 | No | Yes | No | No | Yes | Yes | Yes | No | No | No | 0.139 | Yes | 0.737 | No | No | No |
| CHEMBL2332433 | CC(=C1C[C@@]2(OC1=O)[C@@H](C)CC[C@H]2C(=C)C)C | 234.339 | 3.6307 | 1 | 2 | 0 | 1.214 | 96.972 | No | No | No | No | No | No | No | No | No | No | 1.213 | No | 0.363 | No | No | No |
| CHEMBL476679 | COc1cc(cc(c1OC)OC)[C@H]1[C@@H]2C(=O)OC[C@H]2Cc2c1cc1OCOc1c2 | 398.411 | 2.9183 | 4 | 7 | 0 | 1.304 | 100 | No | Yes | Yes | No | Yes | No | No | Yes | No | Yes | 0.24 | Yes | 0.03 | No | No | No |
| CHEMBL1092399 | COC1=CC(=NC1=CNCCC(C)C)c1ccc[nH]1 | 259.353 | 2.8248 | 6 | 3 | 2 | 1.293 | 92.014 | No | No | No | No | No | No | Yes | No | No | No | 1.059 | No | 0.018 | No | No | Yes |
| CHEMBL428082 | C[C@H]([C@H](c1ccc2c(c1)OCO2)O)[C@@H](Cc1ccc2c(c1)OCO2)C | 342.391 | 3.6923 | 5 | 5 | 1 | 1.337 | 94.289 | No | Yes | Yes | No | Yes | Yes | Yes | Yes | No | Yes | -0.154 | No | 0.307 | No | Yes | No |
| CHEMBL463570 | O[C@H]1C[C@H](C)[C@@]2(C(=C1)CC[C@@H]([C@@H]2C(=O)C)O)C | 238.327 | 1.6797 | 1 | 3 | 2 | 1.229 | 93.239 | No | No | No | No | No | No | No | No | No | No | 1.275 | No | 0.658 | No | No | No |
| CHEMBL559282 | CCC/C=C/C=C/C1=C(CO)[C@@H](O)[C@@H]2[C@@](C1=O)(C/C=C(/C(=O)O)\C)O2 | 348.395 | 1.69 | 8 | 5 | 3 | 0.009 | 55.568 | No | No | No | No | Yes | No | No | No | No | No | 1.623 | No | 0.204 | No | No | No |
| CHEMBL2386704 | CC(=O)O[C@H]1CCC(C2=CC(=O)c3c([C@@]12C)cc1occc1c3C)(C)C | 352.43 | 4.87332 | 1 | 4 | 0 | 1.403 | 98.732 | No | Yes | No | No | Yes | Yes | Yes | Yes | No | No | 0.732 | No | -0.066 | No | No | No |
| CHEMBL105184 | COC(=O)c1cc(OC)c2c(c1c1c(cc(c3c1OCO3)OC)C(=O)OC)OCO2 | 418.354 | 2.4014 | 5 | 10 | 0 | 1.466 | 100 | No | Yes | No | No | Yes | No | No | No | No | No | 0.623 | Yes | 0.693 | No | No | No |
| CHEMBL480675 | OC[C@]12CCCC([C@H]2C[C@H]([C@]23[C@H]1C[C@H](O)[C@H]([C@H]2O)C(=C)C3=O)O)(C)C | 350.455 | 1.0392 | 1 | 5 | 4 | 0.291 | 63.195 | Yes | No | No | No | No | No | No | No | No | No | 0.736 | No | -0.862 | No | No | No |
| CHEMBL239211 | C[C@H](c1oc2c(c1)C(=O)c1c(C2=O)cccc1O)O | 258.229 | 1.8139 | 1 | 5 | 2 | 1.255 | 95.387 | Yes | No | No | No | No | No | No | No | No | No | 0.036 | Yes | 0.226 | No | No | No |
| CHEMBL1271888 | Oc1ccc2c(c1)occ(c2=O)c1ccc2c(c1)C=CC(O2)(C)C | 320.344 | 4.3498 | 1 | 4 | 1 | 1.381 | 95.35 | Yes | Yes | Yes | No | Yes | Yes | Yes | Yes | No | Yes | 0.197 | No | -0.218 | No | No | Yes |
| CHEMBL1969511 | COc1cc2oc(c3ccc4c(c3)OCO4)c(c(=O)c2c(c1OC)OC)OC | 386.356 | 3.2231 | 5 | 8 | 0 | 1.196 | 99.045 | No | Yes | Yes | No | Yes | Yes | Yes | No | No | No | 0.584 | No | 0.276 | No | No | No |
| CHEMBL564146 | COCn1c2ccccc2c2c1CC[C@@]1(CC)CCCN(CC2)C1 | 326.484 | 4.2261 | 3 | 3 | 0 | 1.676 | 92.444 | Yes | Yes | No | Yes | Yes | Yes | No | No | Yes | No | 1.025 | Yes | -0.294 | No | Yes | Yes |
| CHEMBL511768 | OC/C=C(\C(=O)O)/CC[C@@H]1C(=C)CC[C@H]2[C@@]1(C)CC[C@H]([C@@]2(C)CO)O | 352.471 | 2.5119 | 6 | 4 | 4 | 0.708 | 49.929 | Yes | No | No | No | Yes | No | No | No | No | No | 1.301 | No | -0.31 | No | No | No |
| CHEMBL2334483 | CC(=O)O[C@H]1CC[C@@]2([C@@H](C1(C)C)C(=O)C[C@]13[C@H]2[C@@H](O)[C@H](O)[C@H](C1)C(=C)C3=O)C | 390.476 | 1.8166 | 1 | 6 | 2 | 1.003 | 82.682 | Yes | No | No | No | Yes | No | No | No | No | No | 0.518 | Yes | -0.608 | No | No | No |
| CHEMBL239028 | COc1ccc(cc1)c1coc2c(c1=O)cc(c(c2)O)O | 284.267 | 2.8798 | 2 | 5 | 2 | 0.96 | 95.098 | Yes | No | No | No | Yes | Yes | Yes | Yes | No | Yes | 0.174 | Yes | 0.141 | No | No | No |
| CHEMBL481057 | COc1cc2c(cc1OC)CC[C@@H]1[C@@]2(O)C(=O)c2c(O1)cc1c(c2)cco1 | 366.369 | 3.2277 | 2 | 6 | 1 | 1.113 | 98.117 | No | Yes | Yes | No | Yes | Yes | Yes | Yes | No | Yes | 0.474 | No | 0.103 | No | Yes | No |
| CHEMBL591023 | Oc1cc(O)c2c(c1)occ(c2=O)c1ccc2c(c1)OCO2 | 298.25 | 2.5999 | 1 | 6 | 2 | 1.069 | 94.022 | No | No | No | No | Yes | Yes | Yes | Yes | No | Yes | 0.189 | No | 0.104 | No | Yes | No |
| CHEMBL457149 | CC(=O)O[C@H]1CCC([C@]2([C@@]1(C)[C@H]1Cc3occc3[C@@H]3[C@@H]1[C@H](C2)OC3=O)O)(C)C | 388.46 | 2.9699 | 1 | 6 | 1 | 1.329 | 96.346 | No | No | No | No | Yes | No | No | No | No | Yes | 0.375 | No | -0.493 | No | No | No |
| CHEMBL1553601 | O=c1ccc2c(o1)c1CC(=O)C(Oc1cc2)(C)C | 244.246 | 2.0756 | 0 | 4 | 0 | 1.287 | 99.124 | Yes | No | No | No | No | Yes | No | No | No | No | 0.614 | Yes | 0.148 | No | No | Yes |
| CHEMBL557698 | C=C[C@@]1(C)CC[C@H]2[C@@](C1)(O)CC[C@@H]1[C@]2(C)CCC[C@]1(C)C(=O)O | 320.473 | 4.401 | 2 | 2 | 2 | 1.442 | 95.971 | No | No | No | No | Yes | No | No | No | No | No | 0.682 | No | 0.526 | No | No | No |
| CHEMBL505286 | O[C@@H](C#C)COc1ccc(cc1)C(=O)O | 206.197 | 0.7577 | 4 | 3 | 2 | 0.465 | 76.572 | No | No | No | No | No | No | No | No | No | No | 0.667 | No | 0.673 | No | No | No |
| CHEMBL517016 | COC1=C[C@@H](C)[C@H]2[C@@](C1=O)(C)[C@H]1C(=O)C(=C([C@H]3[C@@]1([C@@H](C2)OC(=O)C3)C)C)OC | 388.46 | 2.819 | 2 | 6 | 0 | 1.47 | 99.799 | Yes | Yes | No | No | Yes | No | No | No | No | No | 0.58 | No | 0.039 | No | No | No |
| CHEMBL468863 | COC1=CC(=O)c2c(C1=O)c1c(cc2)cc(c(c1O)OC)OC | 314.293 | 2.4719 | 3 | 6 | 1 | 1.333 | 95.47 | No | No | No | No | Yes | Yes | No | No | No | No | 0.209 | Yes | 0.099 | No | No | No |
| CHEMBL425554 | CC(=O)O[C@H]1[C@H]2[C@H](OC(=O)[C@H]2C)C[C@H]([C@H]2[C@@]1(C)C(=O)C=C2)C | 306.358 | 1.897 | 1 | 5 | 0 | 1.45 | 100 | No | No | No | No | Yes | No | No | No | No | No | 1.048 | Yes | 0.168 | No | No | No |
| CHEMBL1970129 | O=C1CC2OCC=C3C4C2C2N1c1ccccc1C12CCN(C1(C4)O)C3 | 350.418 | 1.4125 | 0 | 4 | 1 | 1.043 | 94.009 | Yes | Yes | No | No | Yes | No | No | No | Yes | No | 0.866 | No | -0.511 | No | Yes | Yes |
| CHEMBL1271155 | O[C@@H]1[C@H](Oc2c(C1=O)c(O)c(c(c2C)O)C)c1ccccc1 | 300.31 | 2.39194 | 1 | 5 | 3 | 0.995 | 93.186 | Yes | No | No | No | No | Yes | Yes | No | No | No | 0.177 | Yes | 0.603 | No | No | No |
| CHEMBL604041 | OC(=O)CCC(=O)OC[C@]1(C)CCC[C@]2([C@H]1CCc1c2ccc(c1)C(O)(C)C)C | 402.531 | 4.3323 | 6 | 4 | 2 | 0.87 | 94.338 | No | No | Yes | No | Yes | No | No | No | No | No | 0.612 | No | -0.186 | No | No | No |
| CHEMBL255684 | CC(=O)[C@H]1[C@H](C[C@@H](C2=CCC[C@@H]([C@@]12C)C)O)C(=O)C | 264.365 | 2.524 | 2 | 3 | 1 | 1.32 | 96.392 | No | No | No | No | No | No | No | No | No | No | 1.304 | No | 0.372 | No | No | No |
| CHEMBL2437370 | COc1c(/C=C/C(=O)c2ccc(c(c2)O)O)ccc(c1CC=C(C)C)O | 354.402 | 4.2168 | 6 | 5 | 3 | 1.246 | 88.604 | Yes | Yes | Yes | No | Yes | Yes | Yes | Yes | No | Yes | 0.294 | No | -0.109 | No | Yes | No |
| CHEMBL512767 | COc1c(oc2c(c1=O)c(O)c(c(c2OC)OC)OC)c1ccc(cc1)O | 374.345 | 2.9056 | 5 | 8 | 2 | 0.053 | 99.325 | Yes | No | Yes | No | No | Yes | No | Yes | No | Yes | 0.627 | No | 0.415 | No | Yes | Yes |
| CHEMBL556677 | OC/C(=C\1/Oc2c(C1=O)c(OC)ccc2)/[C@@H]1OC(=O)C[C@H]1C | 304.298 | 1.4683 | 3 | 6 | 1 | 0.85 | 96.56 | Yes | No | No | No | No | Yes | No | No | No | No | 0.166 | Yes | 0.424 | No | No | Yes |
| CHEMBL1084896 | O[C@H]1CCCc2cc(O)cc(c2C(=O)O[C@@H](C1)C)O | 266.293 | 1.7304 | 0 | 5 | 3 | 0.525 | 89.312 | No | No | No | No | No | No | No | No | No | No | 0.93 | No | 0.164 | No | No | No |
| CHEMBL1079033 | O=C1OCC(=C1)CC[C@@]1(O)[C@H](C)C[C@H]([C@@H]2[C@]1(C)CCCC2(C)C)O | 336.472 | 3.2142 | 3 | 4 | 2 | 1.089 | 96.16 | Yes | Yes | No | No | Yes | No | No | No | No | No | 0.791 | No | -0.452 | No | No | No |
| CHEMBL1916186 | Oc1ccc2c(c1)OC[C@@H](C2=O)Cc1ccc(c(c1)O)O | 286.283 | 2.2374 | 2 | 5 | 3 | 1.1 | 91.662 | Yes | No | No | No | Yes | Yes | No | No | No | No | -0.072 | Yes | -0.223 | No | Yes | No |
| CHEMBL26687 | NCCc1cc(OC)c(c(c1)OC)OC | 211.261 | 1.2136 | 5 | 4 | 1 | 1.228 | 79.093 | No | No | No | No | No | Yes | No | No | No | No | 0.808 | Yes | 1.022 | No | No | No |
| CHEMBL1956008 | OC1=NC[C@]2(C1)C=Cc1c(O2)c2ccccc2oc1=O | 269.256 | 2.2976 | 0 | 4 | 1 | 0.971 | 95.444 | No | No | No | No | Yes | Yes | No | No | No | No | 0.615 | Yes | -0.359 | No | No | No |
| CHEMBL1451874 | Oc1cc(C)c(c(c1)O)C(=O)Oc1cc(C)c(c(c1)O)C(=O)O | 318.281 | 2.33764 | 3 | 6 | 4 | -0.302 | 44.165 | Yes | No | No | No | No | No | No | No | No | No | 0.648 | No | 0.488 | No | No | No |
| CHEMBL490356 | O=C1CCCC[C@@H]1Cc1c(Br)c(O)c(c(c1Br)Br)O | 456.956 | 4.6871 | 2 | 3 | 2 | 1.357 | 88.969 | Yes | No | No | No | No | Yes | Yes | Yes | No | No | -0.374 | No | 0.25 | No | No | No |
| CHEMBL226338 | O=C1C2=C(C[C@H](O2)C(O)(C)C)C(=O)c2c1cccc2 | 258.273 | 1.8795 | 1 | 4 | 1 | 1.09 | 96.125 | No | No | No | No | No | No | No | No | No | No | 0.096 | No | 0.415 | No | No | No |
| CHEMBL475652 | COc1cc(O)cc2c1c1c(O)cccc1CC2 | 242.274 | 2.872 | 1 | 3 | 2 | 1.218 | 94.923 | Yes | No | No | No | No | Yes | Yes | Yes | No | No | 0.366 | Yes | 0.141 | No | Yes | No |
| CHEMBL463760 | O=CC(=C(C)C)CCC1=C[C@H](OC1=O)[C@H]([C@]1(OC)OC(=O)C(=C1)C)C(=C)C | 374.433 | 3.1918 | 8 | 6 | 0 | 1.377 | 98.153 | Yes | Yes | No | No | Yes | No | No | No | No | No | 1.505 | No | 0.31 | No | No | No |
| CHEMBL1972346 | Oc1cc(C=Cc2ccc(c(c2)O)O)cc(c1)O | 244.246 | 2.6794 | 2 | 4 | 4 | 0.924 | 86.165 | Yes | No | No | No | Yes | Yes | Yes | No | No | No | 0.049 | No | 0.127 | No | No | No |
| CHEMBL1797816 | CCCC[C@H](c1nc(O)c2c(n1)cccc2)CC | 244.338 | 4.0192 | 5 | 3 | 1 | 1.383 | 91.002 | No | No | No | No | No | Yes | Yes | Yes | No | No | 1.307 | No | 0.916 | No | No | No |
| CHEMBL176570 | COc1cc(C(=O)O)c2c(c1C)OC(=O)c1c(O2)c(C=O)c(cc1C)O | 358.302 | 2.85324 | 3 | 7 | 2 | 1.335 | 84.836 | Yes | No | No | No | No | No | No | No | No | No | 0.679 | No | 0.727 | No | No | No |
| CHEMBL499143 | O=C1C(=C[C@H]2[C@](C1=C)(C)CC[C@@H]1[C@]2(C)CC[C@](C1)(C)C(=O)O)O | 318.413 | 3.8808 | 1 | 3 | 2 | 0.988 | 99.153 | No | No | No | No | Yes | No | No | No | No | No | 0.765 | No | -0.154 | No | No | Yes |
| CHEMBL254807 | COc1cc2cc(O)c(c(c2c2c1cccc2O)OC)O | 286.283 | 3.127 | 2 | 5 | 3 | 0.843 | 95.164 | Yes | No | No | No | No | Yes | Yes | Yes | No | No | 0.244 | Yes | 0.541 | No | Yes | No |
| CHEMBL443832 | CCc1ccc(cc1)c1ccc(cc1)C(=O)O | 226.275 | 3.6142 | 3 | 1 | 1 | 1.76 | 100 | No | No | No | No | No | Yes | No | No | No | No | 0.676 | No | 0.645 | No | No | No |
| CHEMBL3747112 | COc1cc(CCc2ccccc2O)cc(c1OC)OC | 288.343 | 3.2032 | 6 | 4 | 1 | 1.318 | 95.091 | Yes | No | No | No | Yes | Yes | Yes | Yes | No | Yes | 0.209 | No | 0.508 | No | Yes | No |
| CHEMBL516549 | C=C1CC[C@@H]2O[C@]2(CC[C@H]2[C@H]1COC(=O)C2=CC=CC(O)(C)C)C | 332.44 | 3.3168 | 2 | 4 | 1 | 1.362 | 96.771 | No | Yes | No | No | Yes | No | No | No | No | No | 1.118 | Yes | -0.204 | No | No | No |
| CHEMBL2338628 | OCC1=C[C@@]2(O[C@H](/C=C(/CCC(=O)C(C)C)\C)CC(=C2)C)O[C@H]2[C@@H]1CC(=O)C(=C2)C | 414.542 | 4.2223 | 6 | 5 | 1 | 0.861 | 96.619 | No | Yes | Yes | No | Yes | No | No | No | No | No | 0.939 | No | -0.37 | No | No | No |
| CHEMBL253558 | OC[C@@]1(C)CCC[C@@]2([C@@H]1CC[C@]13[C@H]2CC[C@H](C1)C(=C)C3=O)C | 302.458 | 4.1268 | 1 | 2 | 1 | 1.61 | 97.564 | No | Yes | No | No | Yes | Yes | Yes | Yes | No | Yes | 0.619 | No | -1.013 | No | Yes | No |
| CHEMBL515179 | O=C1CN[C@H]2N1c1ccccc1[C@]12C[C@@H]2C(=N[C@@]1(C)c1n2c(=O)c2c(n1)cccc2)O | 413.437 | 1.7405 | 0 | 6 | 2 | 0.279 | 98.993 | Yes | No | No | No | Yes | No | No | No | No | No | 0.861 | No | -0.662 | No | Yes | Yes |
| CHEMBL335955 | C[C@H]1Oc2c3c(O[C@H]4[C@@H]3C4(C)C)c3c(c2[C@H]([C@H]1C)O)oc(=O)cc3c1ccccc1 | 404.462 | 4.7948 | 1 | 5 | 1 | 1.274 | 98.209 | No | Yes | Yes | No | Yes | No | Yes | Yes | No | No | 0.042 | No | -0.001 | No | Yes | Yes |
| CHEMBL3289674 | COc1cc2OCC=C(c2cc1O)c1ccccc1 | 254.285 | 3.2249 | 2 | 3 | 1 | 1.365 | 95.472 | No | No | No | No | No | Yes | Yes | Yes | No | No | 0.24 | Yes | 0.057 | No | No | No |
| CHEMBL517085 | O=C1O[C@@H]2C[C@]([C@@H]3[C@@]42[C@@H](C1)[C@@H](C)[C@H]([C@@]3(OC4)O)O)(C)[C@@H]1OC(=O)C=C1C | 364.394 | 0.5318 | 1 | 7 | 2 | 0.78 | 88.521 | Yes | No | No | No | Yes | No | No | No | No | No | 0.543 | Yes | -0.4 | No | No | No |
| CHEMBL223942 | CC([C@@H]1CCc2coc(c2)C[C@]2([C@H](CC[C@](/C=C/1)(C)O)O2)C)C | 318.457 | 4.2854 | 1 | 3 | 1 | 1.44 | 94.36 | No | No | No | No | Yes | No | No | No | No | No | 0.916 | No | 0.124 | No | No | No |
| CHEMBL2334889 | COc1cc2CCN3[C@@H](c2cc1OC)Cc1c(C3)c(OC)c(cc1)OC | 355.434 | 3.3765 | 4 | 5 | 0 | 0.928 | 96.993 | Yes | Yes | Yes | No | Yes | Yes | No | No | Yes | No | 1.17 | No | -0.178 | No | Yes | No |
| CHEMBL2386503 | O=c1cc(C)c2c(cc1C(O)(C)C)[C@@](CC2)(C)O | 248.322 | 1.73632 | 1 | 3 | 2 | 1.154 | 95.701 | No | No | No | No | No | No | No | No | No | No | 0.914 | No | 0.526 | No | No | Yes |
| CHEMBL560374 | Oc1cc(/C=C/c2ccccc2)c2c(c1)O[C@@H](CC2=O)c1ccccc1 | 342.394 | 5.2691 | 3 | 3 | 1 | 1.052 | 93.448 | Yes | Yes | Yes | No | Yes | Yes | Yes | Yes | No | Yes | 0.026 | No | 0.183 | No | Yes | No |
| CHEMBL589343 | COC(=O)/C=C/1\CC[C@@]2(O1)[C@H](C)CC[C@@H]1[C@]2(C)CC[C@@H](C1(C)C)OC(=O)C | 378.509 | 4.3966 | 2 | 5 | 0 | 1.35 | 97.197 | No | Yes | No | No | Yes | No | No | No | No | No | 0.76 | No | -0.262 | No | No | No |
| CHEMBL469424 | COc1cc(O)cc(c1Oc1cc(C)cc(c1C(=O)O)O)C(=O)OC | 348.307 | 2.69192 | 5 | 7 | 3 | -0.115 | 49.805 | Yes | No | No | No | No | No | No | No | No | No | 0.779 | No | 1.047 | No | No | No |
| CHEMBL486961 | C=C1CCC[C@]2([C@H]1CC1=C(C)C(=O)O[C@]1(C2)O)C | 248.322 | 2.7047 | 0 | 3 | 1 | 1.311 | 95.311 | No | Yes | No | No | No | Yes | No | No | No | No | 0.991 | No | 0.112 | No | No | No |
| CHEMBL1807146 | COc1cc(O)c(c(c1)c1oc2c(c1)c(O)cc(c2)OC)C | 300.31 | 3.83662 | 3 | 5 | 2 | 0.945 | 94.467 | Yes | No | Yes | No | No | Yes | Yes | Yes | No | No | 0.759 | No | 0.595 | No | Yes | No |
| CHEMBL487081 | CCCCC/C=C/C=C/C(=O)O[C@H]1C(=C[C@]23[C@]1(O)[C@H](O)C(=C[C@H](C3=O)[C@H]1[C@@H](C[C@H]2C)C1(C)C)CO)C | 498.66 | 4.0588 | 8 | 6 | 3 | 0.814 | 75.76 | Yes | Yes | No | No | Yes | No | No | No | No | No | 0.71 | No | -0.82 | No | Yes | Yes |
| CHEMBL497243 | OC[C@@]1(C)CCC[C@]2([C@H]1CCC(=C)[C@@H]2CCC1=CCOC1=O)C | 318.457 | 4.021 | 4 | 3 | 1 | 1.39 | 97.77 | No | Yes | No | No | Yes | Yes | No | No | No | No | 1.115 | No | -0.724 | No | No | Yes |
| CHEMBL512552 | C=CC1=C(C)[C@H]2[C@H](CC1)[C@@]1(C)CCC[C@@]([C@@H]1[C@@H](C2)O)(C)CO | 304.474 | 4.0847 | 2 | 2 | 2 | 1.622 | 94.337 | No | Yes | No | No | Yes | No | Yes | No | No | No | 1.081 | No | -0.341 | No | No | Yes |
| CHEMBL182992 | COc1cc2oc(cc(=O)c2c(c1OC)OC)c1ccccc1 | 312.321 | 3.4858 | 4 | 5 | 0 | 1.317 | 97.755 | No | No | Yes | No | Yes | Yes | Yes | No | No | No | 0.465 | No | 0.399 | No | No | No |
| CHEMBL1668468 | CC(=O)O[C@H]1CC[C@@]2([C@@H]([C@]1(C)CO[C@@H]1CCC(=N1)O)CC[C@]13[C@H]2[C@@H](O)C[C@H](C1)C(=C)C3=O)C | 459.583 | 3.7399 | 4 | 6 | 2 | 0.852 | 96.277 | Yes | Yes | Yes | No | Yes | No | No | No | No | No | 0.43 | No | -1.34 | No | No | No |
| CHEMBL508537 | CC(=O)CC[C@H]1C(=C)C(=O)O[C@@H]1C1=C(C)[C@H](CC1=O)O | 278.304 | 1.1036 | 4 | 5 | 1 | 0.35 | 72.632 | No | No | No | No | No | No | No | No | No | No | 1.347 | Yes | 0.051 | No | No | No |
| CHEMBL2152481 | COCC1=CCc2c(c3c1cc(OC)c(c3)O)ccc(c2)OC | 312.365 | 3.6623 | 4 | 4 | 1 | 1.295 | 98.142 | No | No | No | No | Yes | Yes | Yes | No | No | Yes | 0.225 | Yes | -0.172 | No | Yes | No |
| CHEMBL1950971 | O[C@H]1C[C@@H](O)c2c3C1=C1c4cccc(c4C(=O)C[C@@H]1c3ccc2O)O | 336.343 | 2.4902 | 0 | 5 | 4 | 0.313 | 89.473 | Yes | No | No | No | Yes | No | Yes | No | No | No | 0.076 | No | -0.274 | No | Yes | Yes |
| CHEMBL1950969 | CCO[C@@H]1C[C@H](O)C2=C3[C@@H](c4c2c1c(O)cc4)CC(=O)c1c3cccc1O | 364.397 | 3.5344 | 2 | 5 | 3 | 0.8 | 96.008 | Yes | Yes | No | No | No | Yes | Yes | Yes | No | No | 0.281 | Yes | -0.171 | No | Yes | No |
| CHEMBL2164941 | COc1cc2c(cc1OC)N1[C@@H]3[C@@]42CCN2[C@@]4(O)C[C@@H]4[C@H]3[C@H](CC1=O)OCC=C4C2 | 410.47 | 1.4297 | 2 | 6 | 1 | 0.258 | 97.214 | Yes | No | No | No | Yes | No | No | No | No | No | 0.99 | No | -0.596 | No | No | Yes |
| CHEMBL479018 | CC(C[C@H]1C(=O)N(C(=O)[C@@H]1c1ccc(cc1)OCC=C(C)C)O)C | 331.412 | 3.5355 | 6 | 4 | 1 | 1.155 | 95.278 | No | Yes | No | No | Yes | Yes | Yes | No | No | No | 0.013 | No | 0.62 | No | Yes | Yes |
| CHEMBL1773909 | O[C@H]1C=CC(=O)C(C1)(CC=C(C)C)CC=C(C)C | 248.366 | 3.5753 | 4 | 2 | 1 | 1.1 | 95.413 | No | No | No | No | No | No | No | No | No | No | 1.479 | No | 0.547 | No | No | No |
| CHEMBL2270648 | O=C1O[C@@H]2[C@@]3([C@H]1[C@]1(CO3)C(=O)C=C3[C@]([C@H]1[C@H]2O)(C)C(=O)C=C3C)C | 330.336 | 0.3384 | 0 | 6 | 1 | -0.049 | 82.21 | No | No | No | No | Yes | No | No | No | No | No | -0.072 | Yes | -0.103 | No | No | No |
| CHEMBL103410 | COc1cc(/C=C/C(=O)CC(=O)/C=C/c2ccc(c(c2)O)O)ccc1O | 354.358 | 3.0669 | 7 | 6 | 3 | -0.128 | 80.59 | Yes | Yes | Yes | No | Yes | Yes | Yes | Yes | No | Yes | -0.015 | No | -0.351 | No | Yes | No |
| CHEMBL448815 | OC(=O)C(=O)C1=C(O)/C(=C/c2ccc(c(c2)Cl)O)/N=C1O | 309.661 | 1.8224 | 3 | 5 | 4 | -0.096 | 46.384 | Yes | No | No | No | No | No | No | No | No | No | 0.123 | No | 0.053 | No | No | No |
| CHEMBL253433 | COc1cc(ccc1O)[C@H]([C@@H]([C@@H](Cc1ccc2c(c1)OCO2)C)C)O | 344.407 | 3.6778 | 6 | 5 | 2 | 1.006 | 92.586 | Yes | Yes | Yes | No | Yes | Yes | Yes | Yes | No | Yes | -0.038 | No | 0.016 | No | Yes | No |
| CHEMBL516799 | CSC1=CC(=O)C(=C[C@@]21C=CNc1c2c2nccc3c2c(c1=O)n(c3)C)Br | 440.322 | 3.6924 | 1 | 6 | 1 | 1.294 | 96.52 | No | No | No | No | Yes | No | No | No | No | Yes | -0.05 | No | -0.612 | No | No | Yes |
| CHEMBL376042 | Brc1ccc2c(c1)[nH]cc2[C@@H]1CN=C([C@@H](N1)c1c[nH]c2c1cccc2)O | 409.287 | 4.7538 | 2 | 2 | 4 | 1.042 | 88.819 | Yes | Yes | Yes | Yes | Yes | Yes | Yes | No | Yes | Yes | 0.917 | No | 0.562 | No | Yes | No |
| CHEMBL491544 | COC1C(=O)CCC2C1CN1CCc3c(C1C2)cc1c(c3)OCO1.Cl | 365.857 | 2.7503 | 1 | 5 | 0 | 1.156 | 95.362 | No | No | No | No | Yes | No | No | No | No | No | 1.145 | No | -0.755 | No | No | Yes |
| CHEMBL496894 | C/C=C\1/C[C@@H](C)[C@](O)(CO)C(=O)OCC2=CCN3[C@H]2[C@H](OC1=O)CC3 | 351.399 | 0.1652 | 1 | 7 | 2 | 0.115 | 72.182 | Yes | No | No | No | Yes | No | No | No | No | No | 0.539 | Yes | 0.101 | No | No | Yes |
| CHEMBL2337110 | COC1=Cc2ccnc3c2c(C1=O)ncc3N | 227.223 | 1.3956 | 1 | 5 | 1 | 1.186 | 97.93 | No | No | No | No | No | Yes | No | No | No | No | 0.376 | No | 0.081 | No | No | Yes |
| CHEMBL251978 | Oc1ccc(cc1)[C@@H]1CCc2c(O1)c(C)c(cc2)O | 256.301 | 3.47252 | 1 | 3 | 2 | 1.263 | 92.595 | Yes | No | No | No | Yes | Yes | Yes | Yes | No | No | 0.096 | Yes | 0.294 | No | No | No |
| CHEMBL3578258 | COc1cc(C[C@H]2COC(=O)[C@@H]2Cc2ccc(c(c2)OC)O)cc(c1OC)OC | 402.443 | 3.001 | 8 | 7 | 1 | 1.338 | 95.849 | No | Yes | Yes | No | Yes | No | Yes | Yes | No | Yes | 0.226 | No | 0.349 | No | No | No |
| CHEMBL194378 | CN(CCc1c[nH]c2c1c(ccc2)OP(=O)(O)O)C | 284.252 | 1.7435 | 5 | 3 | 3 | 0.937 | 91.78 | Yes | No | No | No | No | No | No | No | No | No | 0.596 | No | 0.286 | No | No | No |
| CHEMBL229454 | CC(=CCc1cc(cc(c1O)O)[C@@H]1CC(=O)c2c(O1)cc(cc2O)O)C | 356.374 | 3.7242 | 3 | 6 | 4 | 0.275 | 74.847 | Yes | No | No | No | No | Yes | No | Yes | No | No | 0.14 | No | 0.199 | No | No | No |
| CHEMBL507293 | COc1ccc(c2c1CCC(O2)(C)C)C(=O)/C=C/c1ccc(cc1)O | 338.403 | 4.4005 | 4 | 4 | 1 | 1.291 | 93.981 | No | Yes | Yes | No | Yes | Yes | Yes | Yes | No | Yes | 0.067 | No | -0.158 | No | Yes | No |
| CHEMBL556684 | COc1cc(O)c2c(c1)C(=O)c1c(C2=O)c(O)c(c(c1)C)O | 300.266 | 1.89582 | 1 | 6 | 3 | 0.726 | 74.657 | Yes | No | No | No | No | No | No | No | No | No | 0.449 | Yes | 0.754 | No | No | No |
| CHEMBL2419350 | COc1c(O)cc2c(c1C(=O)O)c(=O)c1c(o2)cc(nc1)C | 301.254 | 2.06202 | 2 | 6 | 2 | 1.079 | 58.632 | Yes | No | No | No | No | No | No | No | No | No | 0.758 | No | 1.347 | No | No | Yes |
| CHEMBL464767 | CCCCC[C@]1(CC)OO[C@@H](C(=C1)CC)CC(=O)OC | 284.396 | 3.9454 | 8 | 4 | 0 | 1.533 | 95.576 | No | No | No | No | No | No | No | No | No | No | 1.853 | No | 0.456 | No | No | No |
| CHEMBL1215502 | COc1c2O[C@@H]3[C@H](c2cc(c1OC)O)COc1c3ccc(c1)O | 316.309 | 2.7247 | 2 | 6 | 2 | 1.207 | 94.032 | Yes | No | No | No | Yes | No | Yes | No | No | No | 0.334 | Yes | -0.199 | No | No | No |
| CHEMBL1699927 | COc1c2ccoc2c(c2c1ccc(=O)o2)OCC(C(=C)C)O | 316.309 | 2.8636 | 5 | 6 | 1 | 0.948 | 96.667 | Yes | No | No | No | Yes | Yes | Yes | No | No | No | 0.77 | Yes | -0.198 | No | No | No |
| CHEMBL452243 | C=C([C@@H]1CC[C@@]2([C@@](C1)(O)[C@](C)(O)CCC2)C)C(=O)O | 268.353 | 2.0996 | 2 | 3 | 3 | 0.859 | 96.218 | No | No | No | No | No | No | No | No | No | No | 1.116 | No | 1.123 | No | No | No |
| CHEMBL1956348 | COC1=CC(=O)C[C@H]([C@@]21Oc1c(C2=O)c(OC)cc(c1Cl)O)C | 338.743 | 2.5073 | 2 | 6 | 1 | 1.206 | 96.229 | No | No | No | No | No | No | No | No | No | No | 0.522 | No | 0.636 | No | No | No |
| CHEMBL446639 | COc1ccc(cc1)[C@]1(O)[C@@H](O)C(=Nc2c1cccc2)O | 285.299 | 1.8936 | 2 | 4 | 3 | 1.085 | 95.991 | Yes | No | No | No | Yes | Yes | No | No | No | No | 0.25 | No | 0.096 | No | No | No |
| CHEMBL2164627 | COc1cc2c(cc1OC)N1[C@@H]3[C@@]42CCN(C)CC2=CCO[C@H]([C@@H]3[C@H]2CC4=O)CC1=O | 424.497 | 1.9264 | 2 | 6 | 0 | 1.342 | 97.824 | Yes | Yes | No | No | Yes | No | No | No | No | No | 0.806 | No | -0.715 | No | Yes | Yes |
| CHEMBL406378 | OC/C=C/c1cc2c(c(c1)OC)O[C@@H]([C@H]2CO)c1ccc(c(c1)OC)O | 358.39 | 2.6245 | 6 | 6 | 3 | 1.088 | 92.097 | Yes | Yes | Yes | No | Yes | No | Yes | Yes | No | Yes | 0.108 | No | 0.078 | No | Yes | No |
| CHEMBL1802168 | COC[C@@H]1[C@@H]2CC[C@@H]3[C@]4(C1=O)[C@H]2O[C@@H]1[C@@]23[C@@H](O)CCC([C@H]2[C@@H]([C@@]4(O)O1)O)(C)C | 394.464 | 0.446 | 2 | 7 | 3 | 1.147 | 73.332 | Yes | No | No | No | No | No | No | No | No | No | 0.495 | No | -0.62 | No | No | No |
| CHEMBL463464 | OC[C@]1(/C=C/C=C(C)C)OC(=O)[C@@H]2[C@@H]1CC[C@]1(C)O[C@H]1CCC2=C | 332.44 | 3.3168 | 3 | 4 | 1 | 1.324 | 95.633 | No | Yes | No | No | Yes | No | No | No | No | No | 1.159 | No | -0.374 | No | No | No |
| CHEMBL2409057 | COc1cc2O[C@@H](CCc2cc1O)c1ccc(c(c1)O)O | 288.299 | 2.8783 | 2 | 5 | 3 | 1.207 | 90.133 | Yes | No | No | No | No | Yes | Yes | No | No | No | 0.019 | No | -0.228 | No | No | No |
| CHEMBL480278 | CC(=C)[C@@H]1[C@@H]2OC(=O)C(=C2)CC[C@H]([C@@H](c2oc1cc2C)O)C(=C)C | 328.408 | 4.11902 | 2 | 4 | 1 | 1.325 | 94.778 | No | No | No | No | No | No | No | No | No | No | 1.079 | No | 0.274 | No | No | No |
| CHEMBL460647 | CC(=CCc1c(O)cc(c2c1OC(CC2=O)c1ccc(cc1)O)O)C | 340.375 | 4.0186 | 3 | 5 | 3 | 1.057 | 90.998 | Yes | Yes | No | No | No | Yes | Yes | Yes | No | Yes | 0.111 | No | -0.334 | No | Yes | No |
| CHEMBL512755 | C[C@H]1CCC(=O)C(=C)CC[C@H](C(C=C1)(C)C)O | 236.355 | 3.2651 | 0 | 2 | 1 | 1.59 | 94.38 | No | No | No | No | No | No | No | No | No | No | 1.321 | No | 0.618 | No | No | No |
| CHEMBL1083820 | Oc1cc2O[C@@H](CC(=O)c2c(c1)O)c1ccccc1O | 272.256 | 2.5099 | 1 | 5 | 3 | 1.213 | 88.74 | Yes | No | No | No | No | Yes | Yes | No | No | No | 0.185 | No | -0.443 | No | No | No |
| CHEMBL2287145 | COC(=O)C=Cc1ccc(c(c1)OC)O | 208.213 | 1.587 | 3 | 4 | 1 | 1.214 | 94.701 | No | No | No | No | No | No | No | No | No | No | 0.631 | No | 1.045 | No | No | No |
| CHEMBL471473 | COc1cc2[C@H]3O[C@@H]3CCCC(=O)CCC[C@@H](OC(=O)c2c(c1)O)C | 348.395 | 3.3093 | 1 | 6 | 1 | 1.426 | 94.422 | No | No | No | No | No | No | No | No | No | No | 0.94 | No | -0.393 | No | No | No |
| CHEMBL478951 | CC1=CCCC(=C)[C@H]2[C@H](CC1)[C@H](C/C=C/C(O)(C)C)C(=O)OC2 | 318.457 | 4.1855 | 3 | 3 | 1 | 1.421 | 94.601 | No | Yes | No | No | Yes | No | No | Yes | No | Yes | 1.24 | No | 0.119 | No | No | No |
| CHEMBL1256379 | Oc1cccc(c1)C(=O)c1cc(O)c(c(c1)O)O | 246.218 | 1.74 | 2 | 5 | 4 | -0.465 | 64.039 | Yes | No | No | No | No | No | No | Yes | No | No | 0.077 | No | 0.628 | No | No | No |
| CHEMBL1689080 | CC1=CC[C@@]2(C)C(=O)C[C@@H]([C@@H]2CC[C@]2([C@H](CC1)O2)C)C(=C)C | 302.458 | 4.8419 | 1 | 2 | 0 | 1.51 | 96.784 | No | Yes | No | No | Yes | No | Yes | No | No | No | 1.081 | No | -0.056 | No | No | No |
| CHEMBL2087217 | C/C=C(/C(=O)O[C@H]1CC(=C2[C@@H]([C@@H]3[C@@H]1[C@H](C)C(=O)O3)C(=CC2=O)C)C)\C | 344.407 | 2.9075 | 2 | 5 | 0 | 1.173 | 99.481 | No | Yes | No | No | Yes | No | No | No | No | No | 1.22 | No | -0.184 | No | No | No |
| CHEMBL29711 | OC[C@@]1(O)CC[C@]23C[C@H]1C[C@@H]2CC[C@@H]1[C@]3(C)CC[C@H]([C@@]1(C)CO)O | 338.488 | 2.0858 | 2 | 4 | 4 | 0.928 | 90.525 | Yes | No | No | No | Yes | No | No | No | No | No | 0.767 | No | -1.163 | No | No | No |
| CHEMBL2431871 | O[C@H]1OC[C@@]2(C1)CC[C@@]1(O2)[C@H](C)CC[C@@H]2[C@]1(C)CCCC2(C)C | 322.489 | 4.2756 | 0 | 3 | 1 | 1.133 | 95.344 | No | Yes | No | No | Yes | No | No | No | No | No | 0.608 | No | -0.146 | No | No | No |
| CHEMBL1821996 | NC(=N)NCCCCc1nccc2c1n(O)c1c2ccc(c1)Br | 376.258 | 2.99507 | 5 | 4 | 4 | 0.946 | 79.447 | Yes | No | No | Yes | Yes | Yes | No | No | No | No | 0.863 | Yes | 0.241 | No | Yes | Yes |
| CHEMBL481041 | O=C[C@@]12C[C@@H](OC(=O)C)[C@H](C(C)C)[C@H](OC(=O)C)[C@H]3[C@](CC[C@H]2O1)(C)O3 | 368.426 | 1.7999 | 4 | 7 | 0 | 0.615 | 88.752 | No | No | No | No | Yes | No | No | No | No | No | 0.993 | Yes | 0.167 | No | No | No |
| CHEMBL464742 | COc1oc(C/C=C(/CC/C=C(\C/C(=C/Cc2ccccc2)/C)/C)\C)c(c(=O)c1OC)C | 436.592 | 6.75982 | 11 | 4 | 0 | 1.155 | 94.179 | Yes | Yes | Yes | No | Yes | No | No | Yes | No | No | 1.559 | No | 0.689 | No | Yes | Yes |
| CHEMBL1095333 | CC(=O)O[C@H]1CC(=CC[C@]2([C@H](CC[C@]3([C@H]1O3)C)C(=C(C)C)C(=O)C2)C)C | 360.494 | 4.5276 | 1 | 4 | 0 | 1.189 | 97.283 | No | Yes | No | No | Yes | No | No | No | No | No | 0.694 | No | -0.138 | No | No | No |
| CHEMBL1915271 | OC(=O)CC[C@H]([C@H]1CC[C@@H]2[C@]1(C)[C@@H](O)C[C@H]1[C@H]2[C@H](O)CC2=CC(=O)C=C[C@]12C)C | 402.531 | 3.353 | 4 | 4 | 3 | 0.976 | 71.688 | Yes | No | Yes | No | Yes | No | No | No | No | No | 0.591 | No | -1.515 | No | No | No |
| CHEMBL3039487 | Cc1oc2cc(O)c(c(c2c(=O)c1)O)C | 206.197 | 1.82104 | 0 | 4 | 2 | 1.072 | 93.848 | Yes | No | No | No | No | Yes | No | No | No | No | 0.64 | No | 0.307 | No | No | No |
| CHEMBL1651024 | O=C1C[C@H]2C(C)(C)CCC[C@@]2(c2c1cc(C(C)C)c(c2)O)O | 302.414 | 4.1159 | 1 | 3 | 2 | 1.283 | 91.163 | No | No | No | No | Yes | No | Yes | No | No | No | 0.575 | No | -0.439 | No | No | No |
| CHEMBL3400306 | OC[C@H]1O[C@@H](O[C@H](CC[C@H]2C(=CC(=O)CC2(C)C)C)C)[C@@H]([C@H]([C@@H]1O)O)O | 372.458 | 0.5331 | 6 | 7 | 4 | -0.088 | 41.326 | Yes | No | No | No | No | No | No | No | No | No | 1.318 | No | 0.627 | No | No | No |
| CHEMBL253982 | COc1c2c(CCc3c2ccc(c3)O)cc(c1OC)O | 272.3 | 2.8806 | 2 | 4 | 2 | 1.225 | 94.074 | Yes | No | No | No | Yes | Yes | Yes | No | No | No | 0.106 | Yes | -0.071 | No | Yes | No |
| CHEMBL477898 | CO[C@@H]1Oc2c(O)cccc2[C@]2([C@H]1Oc1cc(OC)c(c(c1C2=O)O)C)O | 374.345 | 1.61122 | 2 | 8 | 3 | 0.168 | 76.794 | Yes | No | No | No | No | No | No | No | No | No | 0.206 | Yes | 0.306 | No | No | No |
| CHEMBL1081411 | COc1cc2OC(=O)c3c(Oc2c(c1Cl)C)cc(c(c3C)Cl)OC | 369.2 | 4.95234 | 2 | 5 | 0 | 1.192 | 94.957 | No | Yes | No | No | Yes | No | Yes | Yes | No | No | 0.471 | No | 0.846 | No | No | No |
| CHEMBL504463 | OC(=NCc1ccccc1)NCc1ccccc1 | 240.306 | 2.8904 | 4 | 1 | 2 | 1.308 | 89.316 | No | No | No | No | Yes | Yes | Yes | No | No | No | 0.76 | No | 0.449 | No | No | No |
| CHEMBL471281 | COc1ccc2c(c1)c1c3OCOc3cc3c1c(C2=O)ncc3 | 305.289 | 3.1835 | 1 | 5 | 0 | 1.265 | 99.967 | No | No | Yes | No | Yes | Yes | Yes | No | No | Yes | 0.436 | Yes | 0.116 | No | No | Yes |
| CHEMBL183745 | COc1cc(O)c2c(c1)oc(cc2=O)c1ccc(c(c1)O)O | 300.266 | 2.5854 | 2 | 6 | 3 | 0.535 | 88.035 | Yes | No | No | No | No | Yes | Yes | Yes | No | No | 0.645 | No | 0.552 | No | Yes | No |
| CHEMBL369474 | COc1cc2O[C@H](Cc2c2c1c(=O)c1c(o2)c(O)ccc1)[C@@]1(C)CO1 | 340.331 | 2.7528 | 2 | 6 | 1 | 1.134 | 100 | No | No | Yes | No | No | Yes | Yes | No | No | No | 0.343 | No | 0.269 | No | Yes | No |
| CHEMBL490162 | COc1cc(ccc1OC(=O)C(C)C)[C@@H]1CC(=O)c2c(O1)cc(cc2O)O | 372.373 | 3.3743 | 4 | 7 | 2 | 0.219 | 82.82 | Yes | Yes | No | No | Yes | No | Yes | Yes | No | Yes | 0.192 | No | 0.436 | No | No | No |
| CHEMBL563622 | C[C@@H]1C[C@H](C)[C@H]([C@@H]([C@@](C1)(C)O)C)c1c(O)ccnc1O | 279.38 | 3.0295 | 1 | 4 | 3 | 1.1 | 93.566 | No | No | No | No | No | No | No | No | No | No | 0.856 | No | -0.322 | No | No | Yes |
| CHEMBL225011 | CC(=CC(=O)OC[C@H]1CC[C@@H]2[C@H]([C@]3([C@@]1(O)CCC3=O)C)OC(=O)C2=C)C | 362.422 | 2.1039 | 3 | 6 | 1 | 1.377 | 97.741 | No | No | No | No | No | No | No | No | No | No | 1.206 | Yes | 0.141 | No | No | No |
| CHEMBL1773905 | CC(=CCc1cc(O)cc(c1O)C=CC(O)(C)C)C | 262.349 | 3.3906 | 4 | 3 | 3 | 1.232 | 93.158 | Yes | No | No | No | No | Yes | No | No | No | No | 0.384 | No | -0.222 | No | No | No |
| CHEMBL489353 | CO[C@@]1(CC=C(C)C)C(=C(CC=C(C)C)C(=O)[C@](C1=O)(O)C(=O)/C=C/c1ccccc1)O | 438.52 | 4.0617 | 8 | 6 | 2 | 0.345 | 76.266 | Yes | Yes | Yes | No | Yes | No | No | No | No | Yes | 1.391 | No | -0.199 | No | No | Yes |
| CHEMBL487996 | O=c1oc(C(C)C)c2c(c1)[C@@]1(C)[C@@H]3O[C@@H]3[C@@H]([C@]3([C@@H]1[C@@H]([C@@H]2O)OC3=O)C)O | 362.378 | 0.7577 | 1 | 7 | 2 | 1.353 | 77.7 | Yes | No | No | No | Yes | No | No | No | No | No | 0.503 | No | -0.428 | No | No | No |
| CHEMBL2334482 | CC(=O)O[C@H]1C[C@@H]2C[C@]3([C@@H]1[C@]1(C)[C@@H](O)C[C@@H](C([C@H]1C(=O)C3)(C)C)O)C(=O)C2=C | 390.476 | 1.8166 | 1 | 6 | 2 | 1.068 | 91.706 | Yes | No | No | No | Yes | No | No | No | No | No | 0.53 | Yes | -0.311 | No | No | No |
| CHEMBL517330 | OC[C@H]1O[C@@H](Oc2ccc3c(c2)OC[C@@H]2[C@H]3Oc3c2ccc(c3)OC)[C@@H]([C@H]([C@@H]1O)O)O | 432.425 | 0.4836 | 4 | 9 | 4 | -0.035 | 63.312 | Yes | Yes | No | No | Yes | No | No | No | No | No | 0.258 | No | 0.083 | No | No | No |
| CHEMBL465969 | COc1ccc(cc1)[C@]1(O)[C@H](O)C(=Nc2c1cccc2)O | 285.299 | 1.8936 | 2 | 4 | 3 | 1.085 | 95.991 | Yes | No | No | No | Yes | Yes | No | No | No | No | 0.25 | No | 0.096 | No | No | No |
| CHEMBL2047326 | COc1cc(OC(=O)C)c2c(c1OC(=O)C)C(=O)c1c(C2=O)cc(c(c1)C)OC(=O)C | 426.377 | 2.55492 | 4 | 9 | 0 | 0.989 | 98.916 | No | Yes | No | No | Yes | No | No | No | No | No | 0.876 | No | 0.929 | No | No | No |
| CHEMBL498039 | CC1=CCCC(=CCC/C(=C/[C@H]2[C@@H](CC1)C(=C)C(=O)O2)/C)C(=O)O | 330.424 | 4.342 | 1 | 3 | 1 | 0.802 | 95.632 | No | No | No | No | No | No | No | No | No | No | 1.374 | No | 0.436 | No | No | No |
| CHEMBL2022669 | OCCCc1ccc(cc1)OCC=C(C)C | 220.312 | 2.9565 | 6 | 2 | 1 | 1.877 | 93.986 | No | No | No | No | No | Yes | No | No | No | No | 0.39 | No | 0.845 | No | No | No |
| CHEMBL2165236 | CC(=CCc1c(O)cc2c(c1O)C(=O)C[C@H](O2)c1ccccc1)C | 324.376 | 4.313 | 3 | 4 | 2 | 1.212 | 91.768 | Yes | Yes | No | No | Yes | Yes | Yes | Yes | No | Yes | 0.234 | Yes | -0.252 | No | Yes | No |
| CHEMBL478710 | CCCC(=O)c1c(O)c2c(cc(=O)oc2c2c1O[C@@H](C2)C(O)(C)C)c1ccccc1 | 408.45 | 4.2228 | 5 | 6 | 2 | 1.166 | 96.847 | Yes | Yes | Yes | No | No | No | Yes | Yes | No | No | 0.639 | No | 0.509 | No | Yes | Yes |
| CHEMBL517338 | O=C1C=C(O)C(=O)C(=C1)C[C@]1(C)[C@@H](C)CC[C@]2([C@H]1CCC=C2C)C | 328.452 | 4.6954 | 2 | 3 | 1 | 1.459 | 95.694 | Yes | Yes | No | No | Yes | No | No | No | No | No | 1.027 | No | 0.078 | No | No | No |
| CHEMBL330320 | COc1c(OC)cc2c(c1OC)c1ccc(c(=O)cc1[C@@H](CC2)N=C(O)C)OC | 399.443 | 3.7119 | 5 | 6 | 1 | 1.129 | 98.587 | Yes | Yes | No | No | Yes | No | Yes | Yes | No | No | 0.556 | No | 0.21 | No | No | No |
| CHEMBL129992 | C[C@H]1Oc2c3c(O[C@@H]4[C@H]3C4(C)C)c3c(c2[C@H]([C@H]1C)O)oc(=O)cc3c1ccccc1 | 404.462 | 4.7948 | 1 | 5 | 1 | 1.274 | 98.209 | No | Yes | Yes | No | Yes | No | Yes | Yes | No | No | 0.042 | No | -0.001 | No | Yes | Yes |
| CHEMBL400327 | COc1cc(OC)c2c(c1)C(=O)C1=C(C2=O)COC(=C1)C | 286.283 | 2.3134 | 2 | 5 | 0 | 1.313 | 98.464 | No | Yes | No | No | Yes | Yes | No | No | No | No | 0.53 | Yes | 0.804 | No | No | No |
| CHEMBL516668 | C=C1[C@@H]2CC[C@@H]3[C@](C1=O)(C2)C(=O)[C@@H](O)[C@H]1[C@@]23CO[C@@H](C1(C)C)CC2=O | 344.407 | 1.472 | 0 | 5 | 1 | 0.165 | 99.59 | Yes | No | No | No | Yes | No | No | No | No | No | 0.511 | No | -0.71 | No | No | No |
| CHEMBL116438 | COc1cc(/C=C/C(=C/C(=O)/C=C/c2ccc(c(c2)OC)O)/O)ccc1O | 368.385 | 3.8526 | 7 | 6 | 3 | 0.196 | 71.716 | Yes | Yes | Yes | No | Yes | No | Yes | Yes | No | Yes | 0.117 | No | -0.057 | No | No | No |
| CHEMBL2431351 | CC(CCC(=O)c1c(O)cc(cc1O)CC(=O)c1ccccc1)C | 326.392 | 4.1421 | 7 | 4 | 2 | 0.909 | 93.012 | Yes | Yes | No | No | No | Yes | Yes | Yes | No | Yes | 0.363 | No | 0.257 | No | Yes | No |
| CHEMBL267742 | Oc1ccc2c(c1)OC[C@@H]1[C@H]2Oc2c1ccc1c2cc(o1)C(=O)C | 322.316 | 3.9507 | 1 | 5 | 1 | 0.91 | 95.896 | No | Yes | Yes | No | Yes | Yes | Yes | Yes | No | No | 0.226 | No | -0.418 | No | No | No |
| CHEMBL1835967 | COc1cc(O)c2c(c1Cc1ccccc1O)O[C@@H](CC2=O)c1ccccc1 | 376.408 | 4.4037 | 4 | 5 | 2 | 0.952 | 94.932 | Yes | Yes | Yes | No | Yes | Yes | Yes | Yes | No | Yes | 0.207 | No | -0.143 | No | Yes | Yes |
| CHEMBL2431352 | COc1ccc(cc1)C(=O)Cc1cc(O)c(c(c1)O)C(=O)CCC(C)C | 356.418 | 4.1507 | 8 | 5 | 2 | 0.924 | 94.054 | Yes | Yes | Yes | No | No | No | Yes | Yes | No | Yes | 0.294 | No | 0.154 | No | Yes | No |
| CHEMBL571616 | COc1cc(C=O)cc2c1oc(c2C)c1ccc2c(c1)OCO2 | 310.305 | 3.95802 | 3 | 5 | 0 | 1.317 | 98.597 | No | Yes | Yes | No | Yes | Yes | Yes | Yes | No | No | 0.518 | No | -0.031 | No | No | Yes |
| CHEMBL2207726 | COC1(OC)COc2cc(O)ccc2c2c(C1)cc(O)c(c2)O | 318.325 | 2.3944 | 2 | 6 | 3 | 1.015 | 94.775 | Yes | No | No | No | No | Yes | Yes | No | No | No | 0.591 | Yes | 0.485 | No | No | No |
| CHEMBL1256380 | Oc1cc(O)c(c(c1)O)C(=O)c1cc(O)cc(c1)O | 262.217 | 1.4456 | 2 | 6 | 5 | -0.573 | 64.917 | Yes | No | No | No | No | Yes | No | No | No | No | 0.599 | Yes | 0.388 | No | No | No |
| CHEMBL3580736 | CCOC(=O)/C=C/c1cc(OC)c(c(c1)OC)O | 252.266 | 1.9857 | 5 | 5 | 1 | 1.223 | 95.697 | No | No | No | No | No | Yes | Yes | No | No | No | 0.837 | No | 1.262 | No | No | No |
| CHEMBL233040 | C/C=C\1/C(=O)C[C@@H]2[C@]1(C)CC[C@H]1[C@H]2CC=C2[C@]1(C)CC[C@@H]([C@@H]2O)O | 330.468 | 3.4062 | 0 | 3 | 2 | 1.341 | 93.123 | Yes | Yes | No | No | Yes | No | No | Yes | No | No | 0.714 | No | 0.084 | No | No | Yes |
| CHEMBL110370 | COc1cc(/C=C/c2cc(O)cc(c2)O)ccc1O | 258.273 | 2.9824 | 3 | 4 | 3 | 1.097 | 90.475 | Yes | No | No | No | No | Yes | Yes | Yes | No | Yes | 0.158 | Yes | 0.005 | No | No | No |
| CHEMBL457680 | CC(=CCc1cc(ccc1O)C1CC(=O)c2c(O1)cc(cc2O)O)C | 340.375 | 4.0186 | 3 | 5 | 3 | 1.197 | 90.07 | Yes | No | No | No | Yes | Yes | Yes | Yes | No | No | 0.135 | No | 0.002 | No | No | No |
| CHEMBL1824574 | O[C@@H](C[C@H](OC(=O)C)CCc1ccc(c(c1)O)O)CCc1ccc(c(c1)O)O | 390.432 | 2.7571 | 9 | 7 | 5 | -0.717 | 68.471 | Yes | Yes | Yes | No | Yes | No | Yes | No | No | Yes | 0.625 | No | 0.011 | No | Yes | No |
| CHEMBL462737 | O=C(C(C)C)O[C@@H]1C[C@H](C[C@]21C(=CC(=O)[C@@H]([C@H]2C)O)C)C(=C)C | 320.429 | 3.0527 | 3 | 4 | 1 | 1.006 | 96.384 | No | No | No | No | No | No | No | No | No | No | 1.247 | No | -0.056 | No | No | No |
| CHEMBL464481 | CC(=O)O[C@@H]1C[C@@H](C)[C@@]2([C@@]3([C@@H]1C(C)(C)CCC3)C)CCC(=O)O2 | 322.445 | 3.8663 | 1 | 4 | 0 | 1.488 | 97.276 | No | Yes | No | No | Yes | No | No | No | No | No | 0.645 | No | 0.01 | No | No | No |
| CHEMBL485479 | CC(C1=Cc2ccc3c(c2C(=O)C1=O)C(=O)CCC3(C)C)C | 296.366 | 3.7455 | 1 | 3 | 0 | 1.439 | 96.793 | No | Yes | No | No | Yes | No | Yes | No | No | No | 0.933 | No | 0.384 | No | No | No |
| CHEMBL560841 | C/C=C/[C@H]1C(=C[C@@H]2[C@H]([C@]1(C)/C=C(/C=C/C(=O)O)\C)C[C@H]([C@H](C2)C)O)C | 344.495 | 4.7552 | 4 | 2 | 2 | 1.162 | 96.334 | No | No | No | No | Yes | No | No | No | No | No | 1.332 | No | -0.389 | No | No | Yes |
| CHEMBL2087212 | O[C@H]1CC[C@]2([C@H]([C@@H]1O)CC[C@@H]1[C@@H]2CC[C@]2([C@H]1CC[C@@H]2[C@@H](N(C)C)C)C)C | 363.586 | 3.9271 | 2 | 3 | 2 | 1.291 | 90.368 | Yes | Yes | Yes | No | Yes | No | No | No | No | No | 0.273 | No | -1.439 | No | No | Yes |
| CHEMBL2419335 | CC1=C[C@@]23O[C@]3(CC(C2=O)(C)C)[C@@H](CC[C@@H]1O)C | 250.338 | 2.2303 | 0 | 3 | 1 | 1.255 | 95.63 | No | No | No | No | No | Yes | No | No | No | No | 0.94 | No | 0.437 | No | No | No |
| CHEMBL496046 | CC(=CC(=O)O)CC[C@]1(C)[C@@H](C)CC[C@]2([C@H]1CC(=O)C=C2C)C | 318.457 | 4.7753 | 4 | 2 | 1 | 1.432 | 98.55 | Yes | No | No | No | Yes | No | No | Yes | No | No | 1.158 | No | -0.052 | No | No | No |
| CHEMBL1076002 | Oc1ccc(cc1)C[C@@H]1N(C)C(=O)[C@@](C1=O)(C)O | 249.266 | 0.0954 | 2 | 4 | 2 | 0.726 | 73.312 | No | No | No | No | No | No | No | No | No | No | 0.209 | No | 0.729 | No | No | No |
| CHEMBL1098658 | COc1ccc(cc1)/C=C/c1cc(OC)cc(=O)o1 | 258.273 | 2.8274 | 4 | 4 | 0 | 1.338 | 95.953 | No | No | No | No | Yes | Yes | Yes | No | No | No | 0.882 | Yes | 0.398 | No | No | No |
| CHEMBL3426666 | COc1cc(OC)c2c(c1C=CC(=C)C)oc(=O)cc2 | 272.3 | 3.3995 | 4 | 4 | 0 | 1.393 | 96.892 | No | No | No | No | Yes | Yes | Yes | No | No | No | 1.038 | Yes | 0.652 | No | No | No |
| CHEMBL486801 | COc1cccc2c1ccc1c2c2OCOc2cc1C(=O)O | 296.278 | 3.4285 | 2 | 4 | 1 | 1.124 | 100 | No | No | No | No | No | No | No | No | No | No | 0.55 | Yes | 0.989 | No | No | No |
| CHEMBL61004 | OC(=O)c1ccc2c(c1)CCC(O2)(C)C | 206.241 | 2.4884 | 1 | 2 | 1 | 1.278 | 93.203 | No | No | No | No | No | No | No | No | No | No | 0.486 | No | 0.499 | No | No | No |
| CHEMBL464725 | C/C=C/c1ccc(c(c1)OC)O[C@H]([C@H](c1ccc(c(c1)OC)OC)O)C | 358.434 | 4.2464 | 8 | 5 | 1 | 1.415 | 92.724 | Yes | Yes | Yes | No | Yes | Yes | Yes | Yes | No | Yes | 0.208 | No | 0.765 | No | Yes | No |
| CHEMBL445274 | O=C1CC[C@]2(C=C1)C(=C)C[C@H]([C@H](C2(C)C)Br)O | 299.208 | 3.0024 | 0 | 2 | 1 | 1.624 | 94.978 | No | No | No | No | No | Yes | No | No | No | No | 0.184 | Yes | 0.371 | No | No | No |
| CHEMBL491882 | CC(=O)OCc1nccc2c1C(=O)C(=C(C2=O)C)O | 261.233 | 1.3557 | 2 | 6 | 1 | 0.526 | 81.49 | No | No | No | No | No | No | No | No | No | No | 0.586 | No | 1.068 | No | No | Yes |
| CHEMBL455764 | CO[C@@H]1O[C@@H]2[C@H]([C@@H]1C)[C@]1(C)[C@@H](C)C[C@@H](C=C1CC2)O | 266.381 | 2.7372 | 1 | 3 | 1 | 1.77 | 96.482 | No | No | No | No | No | No | No | No | No | No | 1.103 | No | 0.422 | No | No | No |
| CHEMBL478084 | CC(CC(=O)c1c(O)c2C[C@H](Oc2c2c1oc(=O)cc2c1ccccc1)C(O)(C)C)C | 422.477 | 4.4688 | 5 | 6 | 2 | 0.972 | 94.08 | Yes | Yes | Yes | No | Yes | No | Yes | Yes | No | Yes | 0.215 | No | 0.332 | No | Yes | Yes |
| CHEMBL1099251 | CC1=C2C[C@]3(C)[C@@H](C)CC[C@H]([C@]3(C[C@H]2OC1=O)O)O | 266.337 | 1.5503 | 0 | 4 | 2 | 0.569 | 95.393 | No | No | No | No | No | No | No | No | No | No | 1.09 | Yes | 0.554 | No | No | No |
| CHEMBL53343 | COc1c(C)c2O[C@@H](CC(=O)c2c(c1C)O)c1ccccc1 | 298.338 | 3.72414 | 2 | 4 | 1 | 1.352 | 94.477 | No | No | No | No | Yes | Yes | Yes | Yes | No | No | 0.236 | No | 0.836 | No | No | No |
| CHEMBL1568181 | COc1c(OC)c(O)cc2c1oc(=O)cc2c1ccccc1 | 298.294 | 3.1828 | 3 | 5 | 1 | 1.077 | 98.209 | No | No | Yes | No | No | Yes | Yes | No | No | No | 0.885 | No | 0.601 | No | Yes | Yes |
| CHEMBL521934 | COc1ccc(cc1)c1oc2cc(OC)c(c(c2c(=O)c1OC)O)OC | 358.346 | 3.2 | 5 | 7 | 1 | 1.274 | 96.939 | Yes | Yes | Yes | No | Yes | Yes | Yes | Yes | No | Yes | 0.693 | No | 0.433 | No | No | No |
| CHEMBL380697 | CN(CCCOc1c(Br)cc(cc1Br)CCN(C)C)C | 408.178 | 3.6462 | 8 | 3 | 0 | 1.536 | 89.352 | Yes | No | No | Yes | Yes | No | No | No | Yes | No | 0.481 | No | 0.46 | No | No | Yes |
| CHEMBL1458 | OC(=NC1CCCCC1)NC1CCCCC1 | 224.348 | 3.1554 | 2 | 1 | 2 | 1.325 | 89.875 | Yes | No | No | No | No | No | No | No | No | No | 1.295 | No | 0.495 | No | No | No |
| CHEMBL484847 | CC(=O)[C@H]1[C@H](O)C[C@@]2([C@]1(C)CC(=O)[C@@]1([C@H]2CC=C2[C@H]1C[C@H](O)[C@H](C2(C)C)O)C)C | 404.547 | 2.6621 | 1 | 5 | 3 | 1.01 | 77.478 | Yes | No | No | No | No | No | No | No | No | No | 0.504 | Yes | -0.836 | No | No | No |
| CHEMBL304564 | CC([C@@H]1CCC(=O)C2=C1[C@H](O)[C@](CC2)(C)O)C | 238.327 | 1.8238 | 1 | 3 | 2 | 1.255 | 95.365 | No | No | No | No | No | No | No | No | No | No | 1.172 | No | 0.673 | No | No | No |
| CHEMBL3126956 | OC[C@]1(O)CC[C@H]([C@@H]1c1nccc2c1[nH]c1c2c(OC)ccc1)C(O)(C)C | 370.449 | 2.7126 | 4 | 5 | 4 | 1.226 | 78.415 | Yes | No | No | No | Yes | Yes | No | No | No | Yes | 0.419 | Yes | 0.351 | No | Yes | Yes |
| CHEMBL1162275 | CNCCc1c[nH]c2c1c(=O)c1c(c[nH]c1c2=O)c1ccc(cc1)O | 335.363 | 2.2527 | 4 | 4 | 4 | 0.449 | 82.449 | Yes | No | No | Yes | Yes | Yes | No | No | No | No | 1.13 | No | 0.057 | No | Yes | Yes |
| CHEMBL1094939 | CC1=CC[C@@H](OC(=O)c2ccc(cc2)O)C(=C[C@@H](C(CCC1)(C)C)O)C | 358.478 | 4.7713 | 2 | 4 | 2 | 0.813 | 89.02 | Yes | No | No | No | Yes | No | Yes | No | No | No | 1.016 | No | -0.236 | No | No | No |
| CHEMBL537954 | COc1ccc(cc1)[C@@H]1CC(=O)c2c(O1)cc(cc2OC)O | 300.31 | 3.1159 | 3 | 5 | 1 | 1.048 | 94.147 | No | No | No | No | Yes | Yes | Yes | No | No | No | 0.218 | No | 0.84 | No | No | No |
| CHEMBL480866 | OC/C(=C/C)/C1=C(C=C)C(C[C@]1(C)CO)(C)C | 236.355 | 2.8361 | 4 | 2 | 2 | 1.604 | 92.269 | No | No | No | No | No | No | No | No | No | No | 0.36 | No | 0.431 | No | No | No |
| CHEMBL524490 | Oc1ccc(c(c1)O)c1oc2c(c1)cc1c(c2)OCO1 | 270.24 | 3.2397 | 1 | 5 | 2 | 0.952 | 91.135 | Yes | No | No | No | Yes | Yes | Yes | Yes | No | No | 0.417 | No | -0.331 | No | Yes | No |
| CHEMBL86075 | COc1cc(/C=C/C(=O)OCC(=O)/C=C/c2ccc(c(c2)OC)O)ccc1O | 384.384 | 2.9539 | 8 | 7 | 2 | -0.013 | 72.646 | Yes | Yes | Yes | No | Yes | No | Yes | Yes | No | Yes | 0.195 | No | -0.072 | No | No | Yes |
| CHEMBL1414826 | COc1cc(cc(c1OC)OC)C(=S)N1CCOCC1 | 297.376 | 1.7201 | 4 | 5 | 0 | 1.88 | 94.804 | No | No | No | No | No | No | No | No | No | No | 0.971 | No | 0.45 | No | No | Yes |
| CHEMBL3234602 | CC(=O)O[C@@H]1[C@H](O)[C@@H](C)[C@@]2([C@@]3([C@@H]1C(C)(C)CCC3)C)CC[C@]1(O2)CO[C@H](C1)O | 396.524 | 2.788 | 1 | 6 | 2 | 1.398 | 96.263 | Yes | No | No | No | No | No | No | No | No | No | 0.599 | No | -0.901 | No | No | No |
| CHEMBL2380793 | O=C1C[C@H](C)C2=C[C@H]3[C@@H](C[C@]2(C1)C)OC(=O)C3=C | 246.306 | 2.4196 | 0 | 3 | 0 | 1.306 | 100 | No | No | No | No | No | Yes | No | No | No | No | 0.594 | No | 0.248 | No | No | No |
| CHEMBL595489 | COc1cc2CCN3C(c2cc1O)Cc1c(C3)c(OC)c(cc1)O | 327.38 | 2.7705 | 2 | 5 | 2 | 1.09 | 96.158 | Yes | No | No | No | Yes | Yes | Yes | No | Yes | No | 1.134 | No | -0.721 | No | Yes | No |
| CHEMBL2437372 | COc1c(/C=C/C(=O)c2ccc(cc2)O)ccc(c1O)O | 286.283 | 2.7081 | 4 | 5 | 3 | 1.201 | 89.882 | Yes | No | No | No | No | Yes | Yes | No | No | No | 0.533 | No | -0.127 | No | No | No |
| CHEMBL255297 | COC1=CC(=O)C(=CC1=O)[C@H](c1ccccc1)C=C | 254.285 | 2.5647 | 4 | 3 | 0 | 1.418 | 97.01 | No | No | No | No | Yes | No | Yes | No | No | No | 0.328 | No | 0.605 | No | No | No |
| CHEMBL519725 | O=C1C[C@H](C/C=C/2\C(=O)O[C@H](C[C@]3(O[C@@H](C1)C(=O)C3)C)[C@H]2O)C(=C)C | 348.395 | 1.6512 | 1 | 6 | 1 | 0.571 | 98.219 | No | No | No | No | No | No | No | No | No | No | 1.224 | Yes | -0.033 | No | No | No |
| CHEMBL478952 | OC/C=C/CC[C@@H]1C(=O)OC[C@@H]2[C@@H]1CCC(=CCCC2=C)C | 304.43 | 3.797 | 4 | 3 | 1 | 1.435 | 95.214 | No | Yes | No | No | Yes | No | No | No | No | Yes | 1.442 | No | 0.062 | No | No | No |
| CHEMBL484901 | OC(=O)C(Cc1c[nH]c2c1cccc2)N | 204.229 | 1.1223 | 3 | 2 | 3 | 0.374 | 62.886 | Yes | No | No | No | No | No | No | No | No | No | 0.634 | No | -0.343 | No | No | No |
| CHEMBL2409058 | COc1cc2O[C@@H](CCc2c(c1)OC)c1ccc(c(c1)O)O | 302.326 | 3.1813 | 3 | 5 | 2 | 1.268 | 92.982 | Yes | No | No | No | Yes | Yes | Yes | No | No | No | 0.242 | Yes | -0.157 | No | No | No |
| CHEMBL28457 | CC(=CC[C@@H](C1=CC(=O)c2c(C1=O)c(O)ccc2O)O)C | 288.299 | 2.1204 | 3 | 5 | 3 | 0.651 | 58.395 | Yes | No | No | No | No | No | No | No | No | No | 0.133 | No | 0.199 | No | No | No |
| CHEMBL451707 | COc1cc(ccc1OC)c1cc(=O)c2c(o1)c(OC)c(cc2OC)OC | 372.373 | 3.503 | 6 | 7 | 0 | 1.094 | 99.745 | No | Yes | Yes | No | Yes | Yes | Yes | No | No | Yes | 0.822 | No | 0.55 | No | Yes | No |
| CHEMBL1807753 | O=C1CC(C)(C)C2=CC=C3C(=C(O)C(=O)C(=C3)C(C)C)[C@@]2(C1)C | 312.409 | 4.2254 | 1 | 3 | 1 | 1.365 | 95.394 | No | Yes | No | No | Yes | No | No | No | No | No | 0.876 | No | -0.035 | No | No | No |
| CHEMBL423178 | CCCCC[C@@H](/C=C/C=C/C#CC#CCCCO)O | 260.377 | 2.8193 | 8 | 2 | 2 | 1.6 | 94.174 | No | No | No | No | Yes | Yes | No | No | No | No | 1.949 | No | 0.093 | No | No | No |
| CHEMBL2334863 | COc1c2c(cc(c1OC)O)[C@H](O)[C@@H](C)[C@H](Cc1c2c(OC)c2c(c1)OCO2)C | 402.443 | 3.6754 | 3 | 7 | 2 | 1.181 | 98.682 | Yes | Yes | Yes | No | Yes | No | Yes | Yes | No | Yes | 0.047 | No | -0.177 | No | Yes | No |
| CHEMBL53757 | O=Cc1c(O)c(C)c2c(c1O)C(=O)C[C@H](O2)c1ccccc1 | 298.294 | 2.92522 | 2 | 5 | 2 | 1.313 | 94.256 | Yes | No | No | No | Yes | Yes | Yes | No | No | No | 0.056 | Yes | -0.245 | No | No | No |
| CHEMBL399742 | C=C1C[C@]23[C@H]([C@@H]1CC[C@@H]3[C@@]1([C@@H](CC2=O)C(C)(C)CCC1)C)O | 302.458 | 4.1252 | 0 | 2 | 1 | 1.734 | 97.229 | No | Yes | No | No | Yes | No | Yes | No | No | No | 0.587 | No | -0.126 | No | Yes | No |
| CHEMBL3596258 | COC1=C(O)c2c(O)cc(c3c2c(C1=O)c(O)cc3O)C | 288.255 | 2.33432 | 1 | 6 | 4 | 0.993 | 63.915 | Yes | No | No | No | No | Yes | No | No | No | No | 0.516 | Yes | 0.818 | No | No | No |
| CHEMBL303062 | O=C1C=C2[C@@]3(O1)C[C@@H](C=C2)N1[C@@H]3CCCC1 | 217.268 | 1.405 | 0 | 3 | 0 | 1.584 | 96.574 | Yes | No | No | No | No | No | No | No | No | No | 1.064 | No | 0.003 | No | No | Yes |
| CHEMBL522898 | COc1cc2C[C@@H](Oc2c2c1C=COC=C2)C(O)(C)C | 274.316 | 2.7412 | 2 | 4 | 1 | 1.291 | 95.017 | No | No | No | No | Yes | No | No | No | No | No | 0.504 | No | 0.237 | No | No | Yes |
| CHEMBL274510 | CC(=O)O[C@@H]1[C@@H](Cl)C2=C([C@@]3([C@@H]1C(C)(C)CCC3)C)COC2=O | 326.82 | 3.2251 | 1 | 4 | 0 | 1.518 | 98.402 | No | Yes | No | No | Yes | No | No | No | No | No | 0.15 | No | 0.042 | No | No | No |
| CHEMBL180970 | O=C1OC[C@H]([C@@H]1Cc1ccc2c(c1)OCO2)Cc1ccc2c(c1)OCO2 | 354.358 | 2.7184 | 4 | 6 | 0 | 0.995 | 98.589 | No | Yes | Yes | No | Yes | Yes | Yes | Yes | No | Yes | -0.065 | No | -0.158 | No | Yes | No |
| CHEMBL1213003 | CC(=CC(=O)O[C@H]1[C@@H]2[C@H](C)C(=O)O[C@@H]2[C@@H]([C@H]([C@H]2[C@@]1(C)C(=O)C=C2)C)O)C | 362.422 | 1.8141 | 2 | 6 | 1 | 1.372 | 98.752 | Yes | No | No | No | Yes | No | No | No | No | No | 1.121 | Yes | -0.141 | No | No | No |
| CHEMBL154 | COc1ccc2c(c1)ccc(c2)[C@@H](C(=O)O)C | 230.263 | 3.0365 | 3 | 2 | 1 | 1.302 | 96.113 | No | No | No | No | No | Yes | No | No | No | No | 0.225 | No | 0.859 | No | No | No |
| CHEMBL2063124 | O[C@H]1c2c(ccc(c2[C@@H](C[C@@]1(C)O)O)C)C(C)C | 250.338 | 2.33992 | 1 | 3 | 3 | 1.254 | 90.329 | Yes | No | No | No | Yes | No | No | No | No | No | 0.894 | No | 0.481 | No | No | No |
| CHEMBL467620 | CC(=O)CC[C@H]1C(=C)C(=O)O[C@@H]1[C@H]1C(=O)C=C[C@@]1(C)O | 278.304 | 0.9595 | 4 | 5 | 1 | 0.512 | 74.254 | No | No | No | No | No | No | No | No | No | No | 0.458 | Yes | 0.209 | No | No | No |
| CHEMBL491987 | CC[C@@H](C[C@]12[C@@H]3C[C@@H]4N1CC[C@@H]2[C@]1(O3)[C@@H]4[C@@H](/C(=C\2/OC(=O)C(=C2OC)C)/O1)C)O | 403.475 | 2.0604 | 4 | 7 | 1 | 0.932 | 94.834 | Yes | Yes | No | No | Yes | No | No | No | No | No | 0.982 | No | -0.646 | No | Yes | Yes |
| CHEMBL1375951 | CC(=O)C1C(=O)C=C2C(C1=O)(C)c1c(O)c(C)c(c(c1O2)C(=O)C)O | 344.319 | 1.49982 | 2 | 7 | 2 | 0.675 | 81.273 | Yes | No | No | No | No | No | No | No | No | No | 0.366 | No | 0.423 | No | No | No |
| CHEMBL1760591 | OCCCc1ccc(c(c1)OC)O[C@@H]([C@@H](c1ccc(c(c1)OC)O)O)CO | 378.421 | 1.8076 | 10 | 7 | 4 | -0.051 | 63.657 | Yes | Yes | Yes | No | Yes | No | No | Yes | No | No | 0.23 | No | 0.679 | No | No | No |
| CHEMBL2207535 | OC1=N[C@@H](Cc2c[nH]c3c2cccc3)C(=O)N([C@H]1C)C | 271.32 | 1.896 | 2 | 2 | 2 | 0.851 | 91.568 | Yes | No | No | No | Yes | Yes | No | No | No | No | 0.843 | No | -0.19 | No | Yes | No |
| CHEMBL1095257 | OCC1=C2C[C@]3(C)[C@@H](C)CC[C@H]4[C@]3(C[C@H]2OC1=O)O4 | 264.321 | 1.5683 | 1 | 4 | 1 | 1.211 | 96.483 | No | No | No | No | Yes | No | No | No | No | No | 1.033 | Yes | 0.263 | No | No | No |
| CHEMBL1172876 | COC(=O)C(=C)[C@@H]1[C@@H](OC(=O)C(=C)CO)C[C@]2([C@@H]([C@H]1O)C(=C)C(=O)OC2)C=C | 392.404 | 0.4583 | 6 | 8 | 2 | 0.326 | 68.558 | Yes | No | No | No | No | No | No | No | No | No | 0.751 | Yes | 0.279 | No | No | No |
| CHEMBL2011652 | COc1cc(O)c2c(c1[C@H]1CC(=O)[C@@H]([C@H](O1)C)O)oc(cc2=O)c1ccc(cc1)O | 412.394 | 2.6597 | 3 | 8 | 3 | 0.753 | 97.186 | Yes | No | Yes | No | No | No | No | No | No | Yes | 0.634 | No | 0.271 | No | Yes | Yes |
| CHEMBL1934198 | CC1(C)CCc2c(O1)cc1c(c2)ccc(=O)o1 | 230.263 | 2.8966 | 0 | 3 | 0 | 1.192 | 98.11 | No | No | No | No | No | Yes | No | No | No | No | 0.6 | No | -0.007 | No | No | No |
| CHEMBL1441727 | C[C@@H]1[C@@H](O)C[C@@H]2[C@H]1[C@H]1OC(=O)[C@H]([C@@H]1[C@H](CC2=C)O)C | 266.337 | 1.118 | 0 | 4 | 2 | 0.056 | 70.685 | No | No | No | No | No | No | No | No | No | No | 1.095 | Yes | 0.213 | No | No | No |
| CHEMBL513640 | CC(=CC[C@@H](C1=CC(=O)c2c(C1=O)c(O)ccc2O)OC(=O)C=C(C)C)C | 370.401 | 3.6375 | 5 | 6 | 2 | 0.92 | 68.315 | Yes | Yes | No | No | No | No | No | No | No | No | 0.417 | No | 0.317 | No | No | No |
| CHEMBL1092163 | COc1ccc(cc1OC)C1=C(C[C@@H]2N(C1)CCC2)c1ccc(c(c1)OC)OC | 395.499 | 4.4999 | 6 | 5 | 0 | 0.915 | 94.248 | No | Yes | Yes | No | Yes | No | No | No | Yes | Yes | 0.959 | No | 0.029 | No | Yes | No |
| CHEMBL480663 | COc1cc2C[C@@H]3N([C@H](c2cc1O)Cc1c3cc2OCOc2c1)C | 325.364 | 2.9559 | 1 | 5 | 1 | 1.221 | 94.614 | Yes | No | No | No | Yes | Yes | No | No | Yes | No | 1.143 | No | -0.816 | No | Yes | Yes |
| CHEMBL2262631 | C/C=C\1/CN(C)CC[C@@]23C(=O)C[C@@H]1[C@H]1CO[C@@H](C(=O)N([C@H]21)c1c3cccc1)O | 382.46 | 1.4752 | 0 | 5 | 1 | 0.746 | 96.653 | Yes | No | No | No | Yes | No | No | No | No | No | 0.4 | No | -0.115 | No | No | Yes |
| CHEMBL562256 | C=C1[C@@H]2CC[C@@H]3[C@](C1=O)([C@@H]2O)[C@H](O)C[C@H]1[C@@]3(C)[C@@H](O)C[C@@H](C1(C)C)O | 350.455 | 1.0376 | 0 | 5 | 4 | 0.513 | 65.497 | Yes | No | No | No | No | No | No | No | No | No | 0.738 | No | -0.309 | No | No | No |
| CHEMBL327134 | CO/C=C(\[C@H]1C[C@H]2N(C[C@@H]1CC)CCc1c2[nH]c2c1cccc2)/C(=O)OC | 368.477 | 3.8165 | 4 | 4 | 1 | 1.121 | 92.839 | Yes | Yes | No | Yes | Yes | No | No | No | Yes | No | 0.993 | Yes | -0.483 | No | Yes | Yes |
| CHEMBL1823040 | COc1cc(C#N)nc(c1)c1ccccn1 | 211.224 | 2.02388 | 2 | 4 | 0 | 1.235 | 100 | No | No | No | No | No | Yes | No | No | No | No | 0.862 | No | 0.077 | No | No | Yes |
| CHEMBL252519 | COc1cc(/C=C/C(=O)Oc2cccc(c2)O)cc(c1O)OC | 316.309 | 2.7338 | 5 | 6 | 2 | 1.392 | 90.331 | Yes | Yes | No | No | Yes | Yes | Yes | No | No | No | 0.47 | Yes | -0.424 | No | No | No |
| CHEMBL2289484 | COc1ccc(cc1)c1cc(O)c(c(c1OC)OC)c1ccc(cc1)OC | 366.413 | 4.7606 | 6 | 5 | 1 | 1.046 | 94.871 | Yes | Yes | Yes | No | Yes | Yes | Yes | Yes | No | Yes | 0.329 | No | 0.447 | No | Yes | No |
| CHEMBL596254 | Clc1ccc(cc1)/C=C/1\Oc2c(C1=O)cccc2 | 256.688 | 3.9562 | 1 | 2 | 0 | 1.258 | 95.12 | No | No | No | No | Yes | Yes | Yes | Yes | No | No | -0.067 | No | 0.738 | No | No | No |
| CHEMBL430628 | CC(=O)O[C@H]1[C@H](OC(=O)C)C2=C(C)C(=O)C[C@H](C2(C)C)C[C@H]2[C@@]1(C)[C@@H](OC(=O)C)C[C@@H](C2=C)O | 476.566 | 3.0602 | 3 | 8 | 1 | 0.99 | 100 | Yes | Yes | No | No | Yes | No | No | No | No | No | 0.632 | No | -0.039 | No | No | No |
| CHEMBL3594097 | COc1cc2c(cc1O)C[C@@]12COc2c(C1=O)c(O)cc(c2)O | 314.293 | 1.8812 | 1 | 6 | 3 | 0.439 | 85.556 | Yes | No | No | No | No | Yes | Yes | No | No | No | 0.27 | Yes | 0.148 | No | No | No |
| CHEMBL499359 | COC(=O)c1cc(O)c(c(c1)C(=O)C=C(C)C)O | 250.25 | 2.0333 | 3 | 5 | 2 | 1.097 | 93.193 | Yes | No | No | No | No | No | No | No | No | No | 0.388 | No | 0.886 | No | No | No |
| CHEMBL253779 | COc1cc(OC)ccc1CCC(=O)c1ccc(cc1)O | 286.327 | 3.2249 | 6 | 4 | 1 | 1.298 | 94.346 | No | No | No | No | Yes | Yes | Yes | Yes | No | Yes | 0.416 | No | 0.657 | No | No | No |
| CHEMBL251229 | COc1cc2CC[n+]3c(c2cc1O)cc1c(c3)c(OC)c(cc1)O | 324.356 | 2.7788 | 2 | 4 | 2 | 1.12 | 97.234 | Yes | No | Yes | No | Yes | Yes | No | No | Yes | No | 1.214 | No | -0.012 | No | Yes | No |
| CHEMBL1909923 | Oc1ccc(c(c1)O)[C@H](CCc1ccc2c(c1)OCO2)O | 288.299 | 2.4927 | 4 | 5 | 3 | 0.935 | 87.709 | Yes | No | No | No | Yes | Yes | No | No | No | Yes | 0.07 | No | -0.095 | No | No | No |
| CHEMBL2409059 | COc1ccc2c(c1)O[C@@H](CC2)c1ccc(c(c1)OC)O | 286.327 | 3.4757 | 3 | 4 | 1 | 1.351 | 92.985 | No | No | No | No | Yes | Yes | Yes | No | No | No | 0.114 | No | 0.486 | No | No | No |
| CHEMBL1797766 | OC[C@@H]1[C@H]2COc3c([C@@H]1c1c(C2)cc(c(c1OC)O)OC)c(OC)c(c(c3)OC)O | 418.442 | 2.4373 | 5 | 8 | 3 | 0.621 | 80.149 | Yes | Yes | No | No | Yes | No | No | No | No | Yes | 0.345 | No | -0.141 | No | No | No |
| CHEMBL103552 | Oc1ccc(c(c1)CN(c1ccc(c(c1)C(=O)O)O)Cc1ccccc1O)O | 381.384 | 3.414 | 6 | 6 | 5 | -0.871 | 49.945 | Yes | No | No | No | No | No | No | No | No | No | 0.287 | No | 0.533 | No | No | No |
| CHEMBL491542 | COc1cc2O[C@H](c3ccc(c(c3)O)O)[C@H](C(=O)c2c(c1)O)OC(=O)C | 360.318 | 2.0601 | 3 | 8 | 3 | 0.324 | 79.379 | Yes | No | No | No | No | No | No | No | No | No | 0.28 | No | -0.146 | No | No | No |
| CHEMBL1277662 | O=C[C@]1(C)CCC[C@]2([C@H]1CCc1c2cc(c(c1)C(C)C)O)C | 300.442 | 4.7248 | 2 | 2 | 1 | 1.738 | 95.802 | No | No | Yes | No | Yes | Yes | Yes | No | No | No | 0.743 | No | -0.22 | No | Yes | No |
| CHEMBL563881 | C=C[C@@]1(C)[C@H](Cl)C[C@H]2[C@@]3([C@]1([N+]#[C-])[C@H](O)C(=O)C(c1c3c3c(C2(C)C)cccc3[nH]1)(C)C)O | 452.982 | 4.34559 | 1 | 3 | 3 | 1.218 | 96.253 | Yes | Yes | Yes | No | Yes | No | No | No | No | Yes | 1.093 | No | -1.101 | No | Yes | Yes |
| CHEMBL1928001 | COc1cc(ccc1OC)[C@H]1O[C@@H]([C@H]([C@H]1C)C)c1cc(OC)c(c(c1)OC)OC | 402.487 | 4.8144 | 7 | 6 | 0 | 1.341 | 97.192 | No | Yes | Yes | No | Yes | No | Yes | Yes | No | Yes | 0.12 | No | 0.743 | No | No | No |
| CHEMBL257745 | COc1c2OCOc2c2c3c1CCN[C@@H]3Cc1c2cccc1 | 295.338 | 2.8338 | 1 | 4 | 1 | 1.379 | 95.826 | No | No | No | No | Yes | Yes | No | No | Yes | No | 1.017 | Yes | -0.296 | No | Yes | Yes |
| CHEMBL513705 | COc1cc(ccc1OC)[C@@H]1O[C@@H]([C@H]([C@@H]1C)C)c1ccc(c(c1)OC)O | 358.434 | 4.5028 | 5 | 5 | 1 | 1.24 | 93.285 | No | Yes | Yes | No | Yes | No | Yes | Yes | No | Yes | 0.024 | No | 0.407 | No | Yes | No |
| CHEMBL1172874 | OCC(=C)[C@]12O[C@@H]2[C@]2(C(=CC1=O)CC[C@H]([C@@H]2C)O)C | 264.321 | 0.9787 | 2 | 4 | 2 | 0.488 | 72.818 | No | No | No | No | No | No | No | No | No | No | 1.22 | Yes | 0.224 | No | No | No |
| CHEMBL480480 | CO[C@H]1OC(=O)C=C2C1=C[C@H]1OC(=O)[C@@]3([C@H]1[C@]2(C)CCC3)C | 304.342 | 2.1202 | 1 | 5 | 0 | 1.453 | 100 | No | Yes | No | No | Yes | No | No | No | No | No | 0.929 | Yes | 0.458 | No | No | No |
| CHEMBL607579 | Oc1ccc2c(c1)c1c3OCOc3cc3c1c(c2)N=C3O | 279.251 | 3.377 | 0 | 4 | 2 | 1.115 | 90.973 | Yes | No | No | No | Yes | Yes | Yes | Yes | No | No | 0.149 | Yes | -0.187 | No | Yes | No |
| CHEMBL462853 | Cc1c(O)cc(cc1O)c1cc2c(o1)cccc2 | 240.258 | 3.81942 | 1 | 3 | 2 | 1.288 | 93.063 | Yes | No | No | No | No | Yes | Yes | Yes | No | No | 0.419 | Yes | 0.108 | No | No | No |
| CHEMBL1651090 | OC[C@@H]1CSC(=N1)c1ccccc1O | 209.27 | 1.2465 | 2 | 4 | 2 | 1.173 | 83.568 | No | No | No | No | No | Yes | No | No | No | No | 0.519 | No | 0.085 | No | No | No |
| CHEMBL1165343 | CC(=O)OC[C@]12CCCC([C@H]2C[C@H]([C@]23[C@H]1[C@@H](O)[C@H](O)[C@H]([C@H]2O)C(=C)C3=O)O)(C)C | 408.491 | 0.5808 | 2 | 7 | 4 | 0.361 | 63.045 | Yes | No | No | No | No | No | No | No | No | No | 0.693 | No | -0.92 | No | No | No |
| CHEMBL567819 | OCCc1c(C)cc2c(c1C)C(=O)C(C2)(C)C | 232.323 | 2.60324 | 2 | 2 | 1 | 1.492 | 95.809 | No | No | No | No | No | Yes | No | No | No | No | 0.267 | No | 0.458 | No | No | No |
| CHEMBL465444 | O[C@H]1CC[C@]2([C@](C1)(O)CC[C@@H]1[C@@H]2CC[C@]2([C@]31O[C@@H]3[C@@H]([C@@H]2c1ccc(=O)oc1)O)C)C | 416.514 | 2.344 | 1 | 6 | 3 | 1.527 | 94.697 | No | No | No | No | Yes | No | No | No | No | No | 0.461 | No | -1.225 | No | No | No |
| CHEMBL447997 | COc1cccc2c1n(C)c1c(c2=O)cco1 | 229.235 | 2.2933 | 1 | 4 | 0 | 1.322 | 100 | No | No | No | No | Yes | Yes | No | No | No | No | 0.384 | Yes | -0.502 | No | No | No |
| CHEMBL448821 | COc1cc(cc(c1OC)O)[C@@H]1CCc2c(O1)c(O)c(cc2)OC | 332.352 | 3.1899 | 4 | 6 | 2 | 1.122 | 93.55 | Yes | No | No | No | No | No | Yes | Yes | No | No | 0.043 | No | 0.73 | No | No | No |
| CHEMBL498428 | CCC[C@H](C1=C(Br)C(=CBr)OC1=O)OC(=O)C | 368.021 | 3.1603 | 4 | 4 | 0 | 1.386 | 94.425 | No | No | No | No | No | No | No | No | No | No | 0.142 | No | 0.436 | No | No | No |
| CHEMBL484662 | Oc1ccc(cc1)c1cc(=O)c2c(o1)cccc2O | 254.241 | 2.8712 | 1 | 4 | 2 | 0.985 | 92.759 | Yes | No | No | No | Yes | Yes | Yes | Yes | No | No | 0.278 | Yes | -0.02 | No | No | No |
| CHEMBL478599 | CC(C[C@@H]1N=C([C@@]23[C@H]1[C@H](C)C(=C[C@@H]3C=C(C)CC[C@@H]([C@@H](C=CC(=O)O2)O)O)C)O)C | 417.546 | 3.4997 | 2 | 5 | 3 | 0.753 | 85.408 | Yes | Yes | No | No | No | No | No | No | No | No | 0.902 | No | -0.305 | No | No | No |
| CHEMBL448376 | O[C@H]1CC(=C)[C@]2(C([C@@H]1Br)(C)C)CC[C@@](C=C2)(C)O | 315.251 | 3.1843 | 0 | 2 | 2 | 1.61 | 90.926 | No | No | No | No | No | Yes | No | No | No | No | 0.248 | No | 0.415 | No | No | No |
| CHEMBL3311043 | O=c1c(coc2c1ccc1c2C=CC(O1)(C)C)c1ccc2c(c1)OCO2 | 348.354 | 4.3729 | 1 | 5 | 0 | 1.001 | 96.35 | Yes | Yes | Yes | No | Yes | Yes | Yes | Yes | No | Yes | -0.065 | Yes | -0.179 | No | No | No |
| CHEMBL479432 | O=C(Cn1cnc2c(c1=O)cccc2)C[C@@H]1NCCC[C@H]1O | 301.346 | 0.4686 | 4 | 6 | 2 | 0.106 | 59.479 | Yes | No | No | No | Yes | Yes | No | No | No | No | 1.129 | Yes | -0.005 | No | Yes | Yes |
| CHEMBL13473 | COc1ccc2c(c1)oc(cc2=O)c1ccc(c(c1)OC)OC | 312.321 | 3.4858 | 4 | 5 | 0 | 1.362 | 97.626 | No | Yes | Yes | No | Yes | Yes | Yes | No | No | No | 0.716 | Yes | 0.086 | No | No | No |
| CHEMBL2375657 | COc1cc2OC[C@](C(=O)c2c(c1OC)OC)(O)Cc1ccc2c(c1)OCO2 | 388.372 | 1.99 | 5 | 8 | 1 | 1.464 | 90.535 | No | Yes | No | No | Yes | No | No | No | No | Yes | 0.084 | No | 0.669 | No | No | No |
| CHEMBL1078441 | CC(=CCOc1ccc2c(c1)oc(=O)cc2)CC[C@H](C(O)(C)C)O | 332.396 | 3.0301 | 7 | 5 | 2 | 0.808 | 93.586 | Yes | Yes | No | No | Yes | Yes | Yes | No | No | No | 0.911 | No | 0.748 | No | Yes | No |
| CHEMBL249669 | Clc1cccc(c1)C(=O)[C@@H](NC(C)(C)C)C | 239.746 | 3.2993 | 3 | 2 | 1 | 1.558 | 89.543 | No | No | No | No | No | Yes | No | No | Yes | No | 0.668 | No | 0.381 | No | No | No |
| CHEMBL249664 | COc1c2C(=O)C(=O)C=C(c2cc(c1O)C)C(C)C | 260.289 | 2.51402 | 2 | 4 | 1 | 1.263 | 95.146 | No | No | No | No | No | Yes | No | No | No | No | 0.016 | Yes | 0.173 | No | No | No |
| CHEMBL1456697 | COc1cc(C=CC(=O)N2CCC=CC2=O)cc(c1OC)OC | 317.341 | 2.0407 | 5 | 5 | 0 | 1.137 | 94.757 | No | No | No | No | Yes | Yes | No | No | No | No | 0.238 | No | 0.451 | No | No | No |
| CHEMBL448932 | CCC[C@H]1OC(=O)CCCC=C[C@@H](C1)O | 212.289 | 2.1894 | 2 | 3 | 1 | 1.286 | 94.188 | No | No | No | No | No | No | No | No | No | No | 1.483 | No | 0.764 | No | No | No |
| CHEMBL460271 | C[C@H]([C@H](C(=O)C)O)C/C=C/[C@H]1[C@H](O)C(=C([C@@H]2[C@@]1(O)C(=N[C@H]2Cc1ccccc1)O)C)C | 427.541 | 2.7745 | 7 | 5 | 4 | 0.546 | 73.18 | Yes | Yes | Yes | No | Yes | No | No | No | No | Yes | 1.12 | No | -1.209 | No | Yes | Yes |
| CHEMBL1223628 | CC(=O)/C=C/C[C@]1(C)CC[C@@H](OO1)[C@H](C(=O)O)C | 256.298 | 2.1117 | 5 | 4 | 1 | 1.027 | 93.455 | No | No | No | No | No | No | No | No | No | No | 1.558 | No | 0.965 | No | No | No |
| CHEMBL15075 | O/N=C(/C(=NCCc1cnc[nH]1)O)\Cc1ccc(c(c1)Br)OC | 381.23 | 2.7527 | 7 | 5 | 3 | -0.227 | 71.565 | Yes | No | No | No | No | No | No | Yes | Yes | Yes | 0.394 | Yes | 0.56 | No | No | No |
| CHEMBL1813343 | CCO[C@@H]1C[C@H]2C(C)(C)CCC[C@@]2(C2=C1C(=O)C(=C(C2=O)O)C(C)C)C | 360.494 | 4.5443 | 3 | 4 | 1 | 0.844 | 96.556 | No | Yes | Yes | No | Yes | No | No | No | No | No | 0.629 | No | -0.253 | No | No | No |
| CHEMBL489133 | COC(=O)c1cc(O)c2c(c1)C=CC(O2)(C)C | 234.251 | 2.363 | 1 | 4 | 1 | 1.255 | 94.417 | No | No | No | No | No | No | No | No | No | No | 0.538 | No | 0.641 | No | No | No |
| CHEMBL485810 | O[C@H]1CC[C@]2([C@@H](C1)CC[C@@H]1[C@@H]2CC[C@]2([C@]31O[C@@H]3C[C@@H]2[C@]12C=CC(=O)O[C@@H]2O1)C)C | 400.515 | 3.3456 | 1 | 5 | 1 | 1.152 | 98.242 | No | Yes | No | No | Yes | No | No | No | No | No | 0.341 | No | -0.391 | No | No | No |
| CHEMBL1946217 | CC(CC(=O)OC(c1coc(c1)c1cc(O)ccc1O)CC=C(C)C)C | 358.434 | 5.3445 | 7 | 5 | 2 | 0.938 | 89.32 | Yes | No | No | No | No | Yes | Yes | Yes | No | Yes | 0.697 | No | 0.559 | No | Yes | No |
| CHEMBL1080425 | O=Cc1c(O)cc(c2c1Oc1c(OC2=O)c(O)c2c(c1C)C(=O)CC(O2)(C)C)C | 398.367 | 3.59584 | 1 | 8 | 2 | 1.183 | 82.538 | Yes | Yes | No | No | No | No | No | Yes | No | Yes | 0.391 | Yes | 0.525 | No | No | No |
| CHEMBL182906 | COc1cc(O)cc(c1Cc1ccc(cc1)O)CCc1cccc(c1)O | 350.414 | 4.188 | 6 | 4 | 3 | 0.587 | 94.141 | Yes | Yes | Yes | No | Yes | Yes | Yes | Yes | No | Yes | 0.316 | No | 0.121 | No | Yes | No |
| CHEMBL2204390 | COc1cc(cc2c1OCO2)[C@H]1O[C@@]2([C@]([C@@H]1C)(O2)C)c1cc(OC)c2c(c1)OCO2 | 414.41 | 3.5105 | 4 | 8 | 0 | 1.41 | 98.917 | No | Yes | Yes | No | Yes | No | Yes | No | No | Yes | -0.102 | No | 0.383 | No | No | No |
| CHEMBL2011665 | COc1cc2[C@H](O)[C@H]3C[C@](C)(O)[C@@H](C[C@@H]3C(=O)c2c(c1)O)O | 308.33 | 0.7686 | 1 | 6 | 4 | 0.711 | 68.829 | Yes | No | No | No | No | No | No | No | No | No | 0.282 | No | -0.736 | No | No | No |
| CHEMBL62448 | CC(=O)O[C@H]1CC[C@]2([C@H](C1(C)C)CC[C@]1([C@@H]2Cc2c(O)cc3c(c2O1)COC3=O)C)C | 428.525 | 4.5404 | 1 | 6 | 1 | 0.973 | 94.578 | Yes | Yes | Yes | No | Yes | No | No | No | No | Yes | 0.128 | No | -0.779 | No | No | No |
| CHEMBL512938 | COc1cc(O)c(c(c1)CO)Oc1cc(C)cc(c1C(=O)O)O | 320.297 | 2.39762 | 5 | 6 | 4 | -0.011 | 59.576 | Yes | No | No | No | No | No | No | No | No | No | 0.688 | No | 0.997 | No | No | No |
| CHEMBL1214423 | O[C@H]1CC[C@@]2(C3=C(OC[C@@H]13)C(=O)c1c2cc2C(=O)CC[C@@H](c2c1)O)C | 340.375 | 2.2057 | 0 | 5 | 2 | 1.509 | 95.84 | No | Yes | No | No | Yes | No | No | No | No | No | 0.698 | No | -0.728 | No | No | No |
| CHEMBL66466 | O=Cc1cc(O)c2c(c1C=O)O[C@@]1([C@H](C2)[C@@]2(C)CC[C@@H](C([C@@H]2CC1)(C)C)OC(=O)C)C | 428.525 | 4.495 | 3 | 6 | 1 | 0.934 | 93.968 | Yes | Yes | Yes | No | Yes | No | Yes | No | No | Yes | 0.287 | No | -0.932 | No | No | No |
| CHEMBL218105 | COc1cc(/C=C/C(=O)O)ccc1OCC=C(C)C | 262.305 | 3.138 | 6 | 3 | 1 | 1.35 | 96.865 | No | No | No | No | No | No | No | No | No | No | 0.774 | No | 0.827 | No | No | No |
| Conformer3D_CID_119093 | OCC(=C)C(=O)O[C@H]1CC(=C)[C@H]2[C@@H]([C@@H]3[C@@H]1C(=C)C(=O)O3)C(=C)[C@H](C2)O | 346.379 | 1.0576 | 3 | 6 | 2 | 0.33 | 94.509 | Yes | No | No | No | Yes | No | No | No | No | No | 0.542 | Yes | -0.076 | No | No | No |
| Conformer3D_CID_160511 | CN[C@H](C(=O)O)Cc1c[nH]c2c1cccc2 | 218.256 | 1.383 | 4 | 2 | 3 | -0.02 | 94.224 | Yes | No | No | No | No | No | No | No | No | No | 0.806 | No | 0.37 | No | No | No |
| CHEMBL563557 | O=C1N[C@@H](Cc2c[nH]c3c2cccc3)C(=O)N2[C@H]1CCC2 | 283.331 | 1.1998 | 2 | 2 | 2 | 0.624 | 74.045 | Yes | No | No | Yes | Yes | Yes | No | No | No | No | 0.503 | No | -0.63 | No | Yes | No |
| CHEMBL266195 | C=CCc1ccccc1OCC(CNC(C)C)O | 249.354 | 2.1528 | 8 | 3 | 2 | 1.785 | 93.226 | Yes | No | No | Yes | No | Yes | No | No | Yes | No | 0.981 | No | 0.204 | No | No | No |
| CHEMBL456 | CCC(=C)C(=O)c1ccc(c(c1Cl)Cl)OCC(=O)O | 303.141 | 3.6057 | 6 | 3 | 1 | 0.941 | 91.763 | No | No | No | No | No | No | No | No | No | No | 0.227 | No | 1.28 | No | No | No |
| Conformer3D_CID_370346 | O=C1c2cc3C(=O)C=CC(=O)c3cc2[C@]2(c3c1occ3C(=O)CC2)C | 332.311 | 3.0417 | 0 | 5 | 0 | 1.018 | 99.122 | No | Yes | No | No | Yes | Yes | Yes | Yes | No | No | 0.07 | Yes | -0.82 | No | No | No |
| CHEMBL350221 | OC(=O)[C@H](Cc1c[nH]c2c1cc(O)cc2)N | 220.228 | 0.8279 | 3 | 3 | 4 | -0.43 | 58.489 | Yes | No | No | No | No | No | No | No | No | No | 0.62 | No | -0.3 | No | No | No |
| CHEMBL86416 | CC1=C[C@H]2OC(=O)C(=C)[C@@H]2CCC(=CCC1)C | 232.323 | 3.5508 | 0 | 2 | 0 | 1.224 | 97.087 | No | No | No | No | No | No | No | No | No | No | 1.334 | No | 0.575 | No | No | No |
| CHEMBL8260 | Oc1cc2oc(cc(=O)c2c(c1O)O)c1ccccc1 | 270.24 | 2.5768 | 1 | 5 | 3 | 0.956 | 90.751 | Yes | No | No | No | Yes | Yes | Yes | Yes | No | Yes | 0.295 | No | 0.281 | No | No | No |
| CHEMBL503 | CC[C@@H](C(=O)O[C@H]1C[C@@H](C)C=C2[C@H]1[C@@H](CC[C@@H]1C[C@@H](O)CC(=O)O1)[C@H](C=C2)C)C | 404.547 | 4.1955 | 6 | 5 | 1 | 0.924 | 94.656 | Yes | Yes | Yes | No | Yes | No | No | No | No | Yes | 0.928 | No | -0.254 | No | Yes | No |
| CHEMBL540445 | CC1=CCC[C@@]2(C)O[C@H]2[C@@H]2[C@@H](CC1)C(=C)C(=O)O2 | 248.322 | 2.762 | 0 | 3 | 0 | 1.65 | 98.029 | No | No | No | No | No | No | No | No | No | No | 1.158 | Yes | 0.31 | No | No | No |
| CHEMBL221283 | OC/C=C(/C(=O)O[C@@H]1CC(=CCCC(=C[C@@H]2[C@@H]1C(=C)C(=O)O2)C)C)\CO | 362.422 | 1.9835 | 4 | 6 | 2 | 0.964 | 78.679 | No | No | No | No | No | No | No | No | No | No | 1.569 | Yes | 0.107 | No | No | No |
| CHEMBL552941 | CN[C@H](C(=O)O)Cc1c[nH]c2c1cccc2 | 218.256 | 1.383 | 4 | 2 | 3 | -0.02 | 94.224 | Yes | No | No | No | No | No | No | No | No | No | 0.806 | No | 0.37 | No | No | No |
| CHEMBL350221 | OC(=O)[C@H](Cc1c[nH]c2c1cc(O)cc2)N | 220.228 | 0.8279 | 3 | 3 | 4 | -0.43 | 58.489 | Yes | No | No | No | No | No | No | No | No | No | 0.62 | No | -0.3 | No | No | No |
| CHEMBL136356 | C[C@H]1CCC[C@]2(C1=C[C@H]1[C@@H](C2)OC(=O)C1=C)C | 232.323 | 3.2406 | 0 | 2 | 0 | 1.603 | 97.057 | No | Yes | No | No | Yes | Yes | Yes | No | No | Yes | 1.076 | No | 0.042 | No | No | No |
| CHEMBL88985 | C=C1C(=O)O[C@H]2[C@H]1CCC(=C)[C@H]1[C@@H]2C(=C)CC1 | 230.307 | 3.0166 | 0 | 2 | 0 | 1.795 | 98.77 | No | Yes | No | No | No | Yes | No | No | No | No | 0.691 | No | 0.565 | No | No | No |
| Conformer3D_CID_9064 | Oc1cc2O[C@H](c3ccc(c(c3)O)O)[C@H](Cc2c(c1)O)O | 290.271 | 1.5461 | 1 | 6 | 5 | -0.411 | 66.773 | Yes | No | No | No | No | No | No | No | No | No | 0.179 | No | 0.244 | No | No | No |
| CHEMBL516538 | OC[C@H]1[C@H](COC(=O)C)[C@H](O[C@@H]1c1cc(OC)c2c(c1)OCO2)c1cc(OC)c2c(c1)OCO2 | 474.462 | 2.7616 | 7 | 10 | 1 | 0.972 | 89.987 | No | Yes | No | No | Yes | No | No | No | No | Yes | 0.435 | No | 0.737 | No | No | No |
| CHEMBL3185643 | CCCCOC(=O)C(=O)OCCCC | 202.25 | 1.673 | 6 | 4 | 0 | 1.295 | 96.474 | No | No | No | No | No | No | No | No | No | No | 0.967 | No | 0.876 | No | No | No |
| CHEMBL448371 | COc1cc(CCc2ccc(cc2)O)cc(c1)O | 244.29 | 2.8916 | 4 | 3 | 2 | 1.245 | 91.349 | Yes | No | No | No | Yes | Yes | Yes | Yes | No | Yes | 0.222 | No | -0.079 | No | No | No |
| CHEMBL503867 | [O-]C(=O)[C@H]([N+](C)(C)C)Cc1c[nH]c2c1cccc2 | 246.31 | 0.5351 | 4 | 2 | 1 | 1.155 | 76.593 | Yes | No | No | No | No | No | No | No | No | No | 0.715 | No | 0.595 | No | No | No |
| CHEMBL3753562 | COC(=O)[C@]1(Cc2ccc(cc2)O)OC(=O)C(=C1c1ccc(cc1)O)O | 356.33 | 2.0782 | 4 | 7 | 3 | -0.316 | 81.71 | Yes | No | No | No | Yes | No | No | No | No | No | 0.218 | No | -0.372 | No | Yes | Yes |
| CHEMBL464817 | CC(=O)O[C@@H]1C/C=C(\C)/CC[C@H]2O[C@@]2(C[C@H]2[C@H](C/C=C/1\C)C(=C)C(=O)O2)C | 374.477 | 4.0301 | 1 | 5 | 0 | 1.005 | 98.119 | No | Yes | No | No | Yes | No | No | No | No | No | 1.206 | No | -0.28 | No | No | No |
| CHEMBL2398585 | COc1cc(/C=C/C(=O)/C=C/c2ccc(cc2)O)ccc1O | 296.322 | 3.4021 | 5 | 4 | 2 | 1.028 | 90.637 | Yes | Yes | No | No | Yes | Yes | Yes | Yes | No | Yes | 0.065 | No | -0.391 | No | Yes | No |
| CHEMBL487789 | O[C@H]1C[C@@H](C)[C@@]2(C3=C1[C@]1(C)CC[C@H](C([C@H]1C[C@H]3Oc1c(C2)cc(cc1)C(=O)O)(C)C)O)C | 440.58 | 4.599 | 1 | 4 | 3 | 0.788 | 99.619 | No | No | No | No | No | No | No | No | No | No | 0.134 | No | 0.414 | No | No | No |
| CHEMBL518356 | CCCc1cc(=O)oc2c1c1OC(C)(C)C=Cc1c1c2[C@@H](O)[C@@H]([C@@H](O1)C)C | 370.445 | 4.3801 | 2 | 5 | 1 | 0.794 | 94.939 | Yes | Yes | Yes | No | Yes | Yes | Yes | No | No | No | 0.51 | No | 0.065 | No | Yes | No |
| CHEMBL2252768 | O=C[C@H]1C(=C[C@H]([C@@H]2[C@]1(C)[C@H](OC(=O)/C=C/c1ccccc1)C[C@@H](C2=C)C)O)C=O | 394.467 | 3.145 | 5 | 5 | 1 | 1.084 | 98.575 | No | Yes | Yes | No | Yes | No | Yes | No | No | Yes | 0.473 | No | -0.022 | No | No | Yes |
| CHEMBL136686 | CO[C@@H]1CC2=CC[C@@H]3[C@@H]([C@]2(C[C@@H]1O)C)CC[C@]1([C@H]3CC=C1[C@@H](N(C)C)C)C | 373.581 | 4.4214 | 3 | 3 | 1 | 1.173 | 92.839 | Yes | Yes | No | No | Yes | No | No | No | No | No | 0.369 | No | -0.537 | No | Yes | Yes |
| CHEMBL2386323 | OC1=C(C)c2ncc(c3c2c(C1=O)c(C)cc3)C | 239.274 | 3.33694 | 0 | 3 | 1 | 1.326 | 95.402 | No | No | No | No | No | Yes | Yes | No | No | No | 0.254 | Yes | 0.436 | No | No | No |
| CHEMBL2271696 | O=Cc1c(O)c(O)c(c2c1c(O)cc(c2)C)C(C)C | 260.289 | 3.20092 | 2 | 4 | 3 | 0.835 | 95.166 | Yes | No | No | No | No | Yes | Yes | Yes | No | No | 0.253 | No | 0.509 | No | No | No |
| CHEMBL563681 | CC(=O)O[C@@H]1[C@H]2CC[C@@H]3[C@]1([C@H](O)C[C@H]1[C@@]3(C)CCC(=O)C1(C)C)C(=O)C2=C | 374.477 | 2.8458 | 1 | 5 | 1 | 1.349 | 99.379 | Yes | No | No | No | Yes | No | No | No | No | No | 0.553 | No | -0.573 | No | No | No |
| CHEMBL508961 | COc1cc(OC)c(c(c1)OC)[C@@H]1CC(=O)c2c(O1)cc(cc2O)OC | 360.362 | 3.1331 | 5 | 7 | 1 | 1.358 | 95.538 | No | Yes | No | No | Yes | Yes | Yes | Yes | No | Yes | 0.728 | No | 0.634 | No | No | No |
| CHEMBL1835614 | CNCCc1c[nH]c2c1ccc(c2)Br | 253.143 | 2.6923 | 3 | 1 | 2 | 1.489 | 92.107 | Yes | No | No | Yes | No | Yes | No | No | No | No | 1.079 | No | 0.079 | No | No | No |
| CHEMBL2204371 | COc1cc(O)c2c(c1C(CC(=C)C)O)oc(cc2=O)C | 290.315 | 2.81522 | 4 | 5 | 2 | 1.057 | 93.219 | Yes | No | No | No | No | Yes | No | No | No | No | 0.445 | No | 0.465 | No | No | No |
| CHEMBL517775 | C/C=C(\C(=O)O[C@H]1[C@H]2[C@@H](C[C@H]([C@H]3[C@@]1(C)C(=O)C=C3)C)OC(=O)C2=C)/CO | 360.406 | 1.7358 | 3 | 6 | 1 | 0.637 | 98.81 | Yes | No | No | No | Yes | No | No | No | No | No | 0.393 | Yes | -0.038 | No | No | No |
| CHEMBL189707 | C/C=C(/C(=O)O[C@@H]1C[C@@]2(C)OC(=CC2=O)C(=C[C@@H]2[C@@H]1C(=C)C(=O)O2)CO)\C | 374.389 | 1.5265 | 3 | 7 | 1 | 0.576 | 100 | Yes | No | No | No | No | No | No | No | No | No | 1.334 | Yes | -0.215 | No | No | No |
| CHEMBL454333 | COc1c(OC)cc(cc1OC)[C@H]1[C@@H]2C(=O)OC[C@@H]2[C@H](c2c1cc1OCOc1c2)OC(=O)C | 456.447 | 2.98 | 5 | 9 | 0 | 1.007 | 100 | No | Yes | No | No | Yes | No | No | Yes | No | Yes | 0.38 | No | 0.441 | No | No | No |
| CHEMBL387124 | O=C1OC[C@H]2[C@@H]1CO[C@H]2c1ccc2c(c1)OCO2 | 248.234 | 1.2758 | 1 | 5 | 0 | 1.239 | 89.468 | No | Yes | No | No | Yes | Yes | No | No | No | No | -0.002 | Yes | 0.167 | No | No | Yes |
| CHEMBL1080598 | COc1cc(ccc1OC)[C@H]1[C@H](C)[C@@H](C)C(=O)c2c1cc(OC)c(c2)O | 356.418 | 4.0184 | 4 | 5 | 1 | 1.243 | 97.698 | No | Yes | Yes | No | Yes | Yes | Yes | Yes | No | Yes | 0.205 | No | 0.168 | No | Yes | No |
| CHEMBL383410 | CO[C@]12CC(=C[C@@H](O)CC(=C)CC[C@H]3C(C2=C(C(=O)O1)CC3)(C)C)C | 346.467 | 4.0561 | 1 | 4 | 1 | 1.312 | 94.928 | No | No | No | No | Yes | No | No | No | No | No | 1.054 | No | -0.434 | No | No | No |
| CHEMBL464559 | Clc1cc(Cl)c(c(c1)[C@@H](c1[nH]c(c(c1[N+](=O)[O-])Cl)Cl)O)O | 371.991 | 4.3238 | 3 | 4 | 3 | -0.511 | 88.643 | Yes | No | No | No | Yes | Yes | Yes | No | No | No | 0.236 | Yes | 0.618 | No | Yes | No |
| CHEMBL477778 | Oc1ccc2c(c1)OC[C@](C2=O)(O)Cc1ccc(c(c1)O)O | 302.282 | 1.3523 | 2 | 6 | 4 | -0.22 | 72.468 | Yes | No | No | No | Yes | No | No | No | No | No | -0.01 | No | -0.298 | No | No | No |
| CHEMBL266195 | C=CCc1ccccc1OCC(CNC(C)C)O | 249.354 | 2.1528 | 8 | 3 | 2 | 1.785 | 93.226 | Yes | No | No | Yes | No | Yes | No | No | Yes | No | 0.981 | No | 0.204 | No | No | No |
| CHEMBL415768 | OC[C@]1(C)[C@H](O)CC[C@@]2([C@@H]1CCC(=C)[C@H]2CCC1=CCOC1=O)C | 334.456 | 2.9918 | 4 | 4 | 2 | 1.062 | 94.997 | Yes | Yes | No | No | Yes | No | No | No | No | No | 1.171 | No | -0.798 | No | No | No |
| CHEMBL464574 | CC(=O)O[C@H]1CCC([C@]2([C@@]1(C)[C@H]1Cc3occc3C(=O)[C@@H]1[C@H](C2)O)O)(C)C | 376.449 | 2.5045 | 1 | 6 | 2 | 0.889 | 91.372 | Yes | No | No | No | No | No | No | No | No | No | 0.57 | No | -0.221 | No | No | No |
| CHEMBL229907 | COc1ccc(cc1)/C=C/C(=O)c1ccc(cc1O)OC | 284.311 | 3.3055 | 5 | 4 | 1 | 1.28 | 92.153 | No | No | No | No | Yes | Yes | Yes | Yes | No | Yes | 0.232 | Yes | 0.636 | No | No | No |
| CHEMBL463974 | CC(=C)[C@H]1Cc2c(O1)ccc(c2)C(=O)C | 202.253 | 2.7688 | 2 | 2 | 0 | 1.26 | 97.22 | No | No | No | No | No | Yes | No | No | No | No | 0.263 | No | 0.724 | No | No | No |
| CHEMBL1976572 | CO[C@@H]1C=C[C@]23[C@H](C1)N(C)C[C@@H]2OC(=O)c1c3cc2OCOc2c1 | 329.352 | 1.481 | 1 | 6 | 0 | 1.261 | 98.101 | No | No | No | No | Yes | No | No | No | No | No | 0.931 | No | -0.219 | No | No | No |
| CHEMBL361362 | COc1cc(cc(c1O)OC)[C@H]1OC[C@H]2[C@@H]1CO[C@@H]2c1cc(OC)c(c(c1)OC)O | 418.442 | 3.2074 | 6 | 8 | 2 | 0.557 | 90.653 | Yes | Yes | No | No | Yes | No | No | Yes | No | Yes | 0.283 | No | -0.291 | No | No | No |
| CHEMBL479328 | CC(C[C@@H]1NC(=O)[C@@]23[C@H]1[C@H](C)C(=C[C@@H]3C=C(C)CC[C@@H]([C@@H](C=CC2=O)O)O)C)C | 401.547 | 2.9329 | 2 | 4 | 3 | 1.238 | 96.705 | Yes | No | Yes | No | No | No | No | No | No | No | 0.886 | No | -0.56 | No | No | No |
| CHEMBL1080242 | COc1cc2cc(Cc3ccc(cc3)O)c(=O)oc2cc1O | 298.294 | 2.8036 | 3 | 5 | 2 | 1.128 | 93.792 | Yes | No | No | No | Yes | Yes | Yes | Yes | No | No | 0.726 | Yes | -0.069 | No | No | No |
| CHEMBL2386513 | CC(=C1C[C@H]2[C@@H]([C@](CC1=O)(C)O)CC[C@]2(C)O)C | 252.354 | 2.2139 | 0 | 3 | 2 | 1.273 | 94.839 | No | No | No | No | No | No | No | No | No | No | 1.068 | No | 0.514 | No | No | No |
| CHEMBL1223695 | CC(=O)CCCC(C(=O)CCC(=O)C)(C)C | 226.316 | 2.7102 | 8 | 3 | 0 | 1.421 | 97.374 | No | No | No | No | No | No | No | No | No | No | 1.452 | No | 0.647 | No | No | No |
| CHEMBL399672 | C/C=C(\C(=O)O[C@@H]1C[C@]2(CO2)[C@H]2[C@]([C@@H]3[C@@H]1C(=C)C(=O)O3)(O)[C@]1([C@@H]([C@H]2O)O1)C)/C | 392.404 | 0.0142 | 2 | 8 | 2 | 0.87 | 100 | Yes | No | No | No | No | No | No | No | No | No | 0.829 | No | -0.792 | No | No | No |
| CHEMBL147067 | O=C(c1ccccc1)/C=C/c1ccccc1O | 224.259 | 3.2883 | 3 | 2 | 1 | 1.769 | 92.528 | No | No | No | No | Yes | Yes | Yes | Yes | No | No | 0.191 | No | 0.657 | No | No | No |
| CHEMBL1084170 | O[C@@H]1CCCc2cc(O)cc(c2C(=O)O[C@@H](C1)C)O | 266.293 | 1.7304 | 0 | 5 | 3 | 0.525 | 89.312 | No | No | No | No | No | No | No | No | No | No | 0.93 | No | 0.164 | No | No | No |
| CHEMBL504850 | OC(=O)c1nc(C)c2c(c1)c1ccccc1[nH]2 | 226.235 | 2.72272 | 1 | 2 | 2 | 1.175 | 91.659 | Yes | No | No | No | No | Yes | No | No | No | No | 0.878 | No | 0.331 | No | No | No |
| CHEMBL92528 | CCCc1cc(=O)oc2c1c1OC(C)(C)C=Cc1c1c2[C@@H](OC)[C@@H]([C@H](O1)C)C | 384.472 | 5.0342 | 3 | 5 | 0 | 1.354 | 96.13 | No | Yes | Yes | No | Yes | Yes | Yes | Yes | No | No | 0.72 | No | -0.051 | No | No | No |
| CHEMBL466164 | C=C[C@@]1(C)C=C2CC[C@@H]3[C@@]([C@H]2C[C@H]1O)(C)CCC(=O)[C@@]3(C)CO | 318.457 | 3.2637 | 2 | 3 | 2 | 1.314 | 95.116 | No | Yes | No | No | Yes | No | No | No | No | No | 1.122 | No | -0.416 | No | No | No |
| CHEMBL3233981 | OC[C@@]1(C)[C@@H](O)CC[C@@]2([C@@H]1C[C@@H](O)[C@]13[C@H]2CC(=O)[C@H]([C@H]1O)C(=C)C3=O)C | 364.438 | 0.2182 | 1 | 6 | 4 | 0.3 | 66.424 | Yes | No | No | No | No | No | No | No | No | No | 0.775 | No | -0.523 | No | No | No |
| CHEMBL424997 | CC(=O)O[C@@H]1C[C@H]2O[C@@]2(C)C[C@H]2[C@H]([C@H]3C=C1C(=O)O3)C(=C)C(=O)O2 | 334.324 | 0.819 | 1 | 7 | 0 | 0.862 | 100 | No | No | No | No | Yes | No | No | No | No | No | 1.099 | Yes | -0.203 | No | No | No |
| CHEMBL87 | COc1c(OC)cc2c(c1OC)c1ccc(c(=O)cc1C(CC2)NC(=O)C)OC | 399.443 | 2.8716 | 5 | 6 | 1 | 1.191 | 100 | Yes | No | No | No | Yes | No | Yes | Yes | No | No | 0.284 | No | 0.164 | No | No | No |
| CHEMBL490355 | Oc1ccc2c(c1)O/C(=C\c1ccc(c(c1)O)O)/C2=O | 270.24 | 2.4196 | 1 | 5 | 3 | 1.056 | 88.194 | Yes | Yes | No | No | No | No | No | No | No | No | -0.086 | Yes | 1.036 | No | No | No |
| CHEMBL1988530 | CO[C@H]([C@H](/C(=C\C(=O)OC)/OC)C)/C=C/c1csc(n1)c1csc(n1)C(C)C | 422.572 | 4.7576 | 9 | 8 | 0 | 0.869 | 94.271 | No | No | No | No | Yes | No | Yes | Yes | No | No | 1.041 | No | 0.503 | No | No | Yes |
| CHEMBL519395 | COc1cc(O)c(c(c1)C1=C(C)C[C@H](C1=O)O)C(=O)O | 278.26 | 1.2062 | 3 | 5 | 3 | 0.74 | 58.622 | Yes | No | No | No | No | No | No | No | No | No | 0.541 | No | 1.482 | No | No | No |
| CHEMBL1087405 | CC1=C[C@H](O)[C@H]2[C@H](CC(=C)C(=O)CC1)OC(=O)C2=C | 262.305 | 1.7006 | 0 | 4 | 1 | 0.543 | 97.529 | No | No | No | No | No | No | No | No | No | No | 0.573 | No | 0.559 | No | No | No |
| CHEMBL448874 | Oc1ccc2c(c1)c(=O)c1c(o2)ccc(c1)O | 228.203 | 2.3574 | 0 | 4 | 2 | 1.01 | 92.836 | Yes | No | No | No | Yes | Yes | No | No | No | No | 0.499 | Yes | 0.028 | No | No | No |
| CHEMBL1712170 | CCOc1ccc2c3c1O[C@@H]1[C@@]43CCN([C@H](C2)[C@@H]4C=C[C@@H]1O)C | 313.397 | 1.8912 | 2 | 4 | 1 | 1.558 | 95.242 | Yes | No | No | Yes | Yes | No | No | No | Yes | No | 0.887 | No | -0.532 | No | No | No |
| CHEMBL575429 | OC(=O)/C=C/c1ccc(cc1)OS(=O)(=O)O | 244.224 | 0.966 | 4 | 4 | 2 | 0.171 | 28.998 | No | No | No | No | No | No | No | No | No | No | 0.622 | No | 1.008 | No | No | No |
| CHEMBL508813 | O=Cc1ccc2c1cc1c(C)coc1cc2C | 224.259 | 3.96694 | 1 | 2 | 0 | 1.458 | 96.033 | No | No | No | No | Yes | Yes | Yes | No | No | No | 0.297 | No | 0.167 | No | No | No |
| CHEMBL463165 | O=C1c2ccccc2N([C@H]2N1CCc1c2[nH]c2c1cccc2)C | 303.365 | 3.3148 | 0 | 2 | 1 | 1.682 | 93.845 | Yes | No | Yes | Yes | Yes | Yes | Yes | Yes | Yes | Yes | 0.283 | Yes | 0.237 | No | Yes | No |
| CHEMBL1966898 | COc1cc(OC)c2c(c1)c(OC)c1c(n2)occ1 | 259.261 | 3.0068 | 3 | 5 | 0 | 1.41 | 99.394 | No | No | No | No | Yes | Yes | Yes | No | No | Yes | 0.85 | Yes | 0.462 | No | No | No |
| CHEMBL250348 | CC(=O)O[C@@H]1C=C(C)[C@@H](CC=C(C[C@H]2[C@H]1C(=C)C(=O)O2)C)OC(=O)C | 348.395 | 2.634 | 2 | 6 | 0 | 1.23 | 99.705 | No | No | No | No | Yes | No | No | No | No | No | 1.371 | No | -0.026 | No | No | No |
| CHEMBL1779811 | C/C=C/C[C@@H]([C@H]([C@H]([C@@H](C[C@H]1OC(=O)C=C[C@H]1CC)O)C)OC)C | 324.461 | 3.4986 | 9 | 4 | 1 | 1.453 | 94.346 | No | No | No | No | No | No | No | No | No | No | 1.767 | No | 0.32 | No | No | No |
| CHEMBL254888 | COc1cccc2c1C(=O)c1c(C2=O)cccc1O | 254.241 | 2.1762 | 1 | 4 | 1 | 1.16 | 99.195 | No | No | No | No | No | Yes | Yes | Yes | No | No | 0.092 | Yes | 0.265 | No | No | No |
