# Supplementary material for: In Silico Identification and Analysis of Potentially Bioactive Antiviral Phytochemicals against SARS-CoV-2: A Molecular Docking and Dynamics Simulation Approach
Source: Biomed Res Int. 2023 May 11;2023:5469258. doi: 10.1155/2023/5469258 (PMC10195178; doi:10.1155/2023/5469258)
Supplement: Supplementary 3 — Supplementary Table 3a: binding affinity of the main protease (PDB ID: 7NT3) and the phytochemicals and control drugs. Supplementary Table 3b: binding affinity of the NSP3 (PDB ID: 7KQP) and the phytochemicals and control drugs. Supplementary Table 3c: binding affinity of human ACE2 receptor(PDB ID: 1R4L) and phytochemicals. [file 5469258.f3.docx]

Supplementary table 3a. Binding affinity of the main protease (PDB ID: 7NT3) and the phytochemicals and control drugs

| **Chembbl id** | **Affinity(k cal/mol)** |
| --- | --- |
| CHEMBL487789 | -8.4 |
| CHEMBL493857 | -7.1 |
| CHEMBL1835614 | -5.1 |
| CHEMBL520327 | -6.8 |
| CHEMBL3183581 | -5.2 |
| CHEMBL45763 | -4.6 |
| CHEMBL2271696 | -6 |
| CHEMBL2252768 | -7.3 |
| CHEMBL2204371 | -5.7 |
| CHEMBL415768 | -7 |
| CHEMBL516538 | -7.1 |
| CHEMBL4216332 | -7 |
| CHEMBL464574 | -6.9 |
| CHEMBL464817 | -6.9 |
| CHEMBL2386323 | -6.5 |
| CHEMBL477778 | -6.9 |
| CHEMBL1080598 | -6.3 |
| CHEMBL563681 | -6.9 |
| CHEMBL508961 | -5.9 |
| CHEMBL501870 | -5 |
| CHEMBL503867 | -5.5 |
| CHEMBL383410 | -7.3 |
| CHEMBL557501 | -7.4 |
| CHEMBL3185643 | -4.4 |
| CHEMBL1160785 | -4.9 |
| CHEMBL226683 | -4.9 |
| CHEMBL2227793 | -6.1 |
| CHEMBL454333 | -7.3 |
| CHEMBL249454 | -7.9 |
| CHEMBL519395 | -5.9 |
| CHEMBL2312529 | -3.9 |
| CHEMBL517080 | -8.2 |
| CHEMBL3184590 | -4.3 |
| CHEMBL87 | -6.8 |
| CHEMBL466164 | -6.6 |
| CHEMBL1223695 | -4.6 |
| CHEMBL506889 | -4.9 |
| CHEMBL463165 | -7.6 |
| CHEMBL2386513 | -6 |
| CHEMBL14117 | -5 |
| CHEMBL361362 | -7.1 |
| CHEMBL424997 | -6.6 |
| CHEMBL1080242 | -7.1 |
| CHEMBL479328 | -7.9 |
| CHEMBL92528 | -7.1 |
| CHEMBL1087405 | -6.7 |
| CHEMBL1094876 | -5.8 |
| CHEMBL1084170 | -6.1 |
| CHEMBL3348832 | -5.1 |
| CHEMBL3337754 | -3.4 |
| CHEMBL250348 | -6.6 |
| CHEMBL399672 | -6.8 |
| CHEMBL472877 | -5.3 |
| CHEMBL3357563 | -4.8 |
| CHEMBL1712170 | -7.5 |
| CHEMBL147067 | -6.4 |
| CHEMBL504850 | -6.5 |
| CHEMBL1834810 | -7.4 |
| CHEMBL575429 | -5.7 |
| CHEMBL1779811 | -6 |
| CHEMBL508813 | -6.6 |
| CHEMBL442565 | -5.1 |
| CHEMBL390320 | -5.6 |
| CHEMBL103686 | -3.9 |
| CHEMBL463974 | -5.8 |
| CHEMBL592620 | -5.3 |
| CHEMBL3233981 | -7.1 |
| CHEMBL1079033 | -6.7 |
| CHEMBL1080979 | -6.7 |
| CHEMBL1087720 | -7.2 |
| CHEMBL1092163 | -7 |
| CHEMBL1095333 | -6.6 |
| CHEMBL1096781 | -6.6 |
| CHEMBL1170899 | -6.8 |
| CHEMBL1277662 | -6.2 |
| CHEMBL141117 | -6.6 |
| CHEMBL1456697 | -5.6 |
| CHEMBL1651023 | -6.9 |
| CHEMBL1651024 | -6.8 |
| CHEMBL1651069 | -6.9 |
| CHEMBL1669578 | -6.6 |
| CHEMBL1689080 | -7.4 |
| CHEMBL1807753 | -6.9 |
| CHEMBL182992 | -6.2 |
| CHEMBL1909923 | -6.6 |
| CHEMBL1915271 | -7.2 |
| CHEMBL1969511 | -6.3 |
| CHEMBL1972346 | -6.5 |
| CHEMBL2057716 | -7 |
| CHEMBL2087217 | -7.7 |
| CHEMBL2204390 | -7.7 |
| CHEMBL223942 | -7 |
| CHEMBL2334478 | -7.1 |
| CHEMBL2334889 | -7.5 |
| CHEMBL2337574 | -7.4 |
| CHEMBL2385627 | -7.3 |
| CHEMBL2386704 | -6.9 |
| CHEMBL2431871 | -7.8 |
| CHEMBL251229 | -6.2 |
| CHEMBL253688 | -6.5 |
| CHEMBL274510 | -6.5 |
| CHEMBL29711 | -6.8 |
| CHEMBL3093767 | -7.3 |
| CHEMBL325752 | -6.3 |
| CHEMBL3287734 | -7.2 |
| CHEMBL330320 | -6.8 |
| CHEMBL399742 | -4.8 |
| CHEMBL423178 | -4.5 |
| CHEMBL430628 | -7.5 |
| CHEMBL446639 | -7.1 |
| CHEMBL451784 | -4.8 |
| CHEMBL453193 | -4.4 |
| CHEMBL453563 | -6.4 |
| CHEMBL457149 | -4.8 |
| CHEMBL459615 | -6.3 |
| CHEMBL463464 | -6.5 |
| CHEMBL463760 | -6.7 |
| CHEMBL463922 | -6.2 |
| CHEMBL464176 | -6.6 |
| CHEMBL464481 | -6.6 |
| CHEMBL465444 | -7.1 |
| CHEMBL465475 | -7.1 |
| CHEMBL465969 | -6.7 |
| CHEMBL478951 | -6.3 |
| CHEMBL478952 | -7.6 |
| CHEMBL485479 | -7.7 |
| CHEMBL485810 | -7.2 |
| CHEMBL487996 | -6.5 |
| CHEMBL496046 | -6.5 |
| CHEMBL504463 | -6.4 |
| CHEMBL511568 | -7.1 |
| CHEMBL514518 | -7.3 |
| CHEMBL517016 | -7.4 |
| CHEMBL517338 | -7 |
| CHEMBL517700 | -7.3 |
| CHEMBL550232 | -6.8 |
| CHEMBL575533 | -6.6 |
| CHEMBL589343 | -7 |
| CHEMBL62448 | -7.8 |
| CHEMBL1214423 | -7.5 |
| CHEMBL66466 | -8.5 |
| CHEMBL218105 | -5.9 |
| CHEMBL563557 | -6.5 |
| CHEMBL266195 | -5.3 |
| CHEMBL456 | -5.8 |
| CHEMBL86416 | -6.9 |
| CHEMBL503 | -7.2 |
| CHEMBL136356 | -6.3 |
| CHEMBL88985 | -6.3 |
| Molnupiravir | -6.4 |
| paxlovid | -6.6 |

Supplementary table 3b. Binding affinity of the NSP3 (PDB ID: 7KQP) and the phytochemicals and control drugs.

| **Chembbl id** | **Affinity(kcal/mol)** |
| --- | --- |
| CHEMBL516538 | -7.800 |
| CHEMBL3185643 | -8.900 |
| CHEMBL503867 | -6.700 |
| CHEMBL464817 | -7.500 |
| CHEMBL1160785 | -6.200 |
| CHEMBL487789 | -8.700 |
| CHEMBL226683 | -7.600 |
| CHEMBL501870 | -6.900 |
| CHEMBL2252768 | -7.000 |
| CHEMBL493857 | -8.700 |
| CHEMBL557501 | -7.400 |
| CHEMBL2386323 | -8.200 |
| CHEMBL2271696 | -9.000 |
| CHEMBL563681 | -7.900 |
| CHEMBL508961 | -6.600 |
| CHEMBL4216332 | -9.200 |
| CHEMBL249454 | -7.400 |
| CHEMBL1835614 | -8.000 |
| CHEMBL2204371 | -7.700 |
| CHEMBL2227793 | -7.900 |
| CHEMBL3183581 | -7.400 |
| CHEMBL520327 | -6.700 |
| CHEMBL45763 | -6.400 |
| CHEMBL454333 | -8.300 |
| CHEMBL1080598 | -7.200 |
| CHEMBL383410 | -6.000 |
| CHEMBL477778 | -6.200 |
| CHEMBL266195 | -6.700 |
| CHEMBL415768 | -8.000 |
| CHEMBL464574 | -8.700 |
| CHEMBL3184590 | -6.500 |
| CHEMBL3357563 | -7.200 |
| CHEMBL463974 | -8.100 |
| CHEMBL361362 | -7.800 |
| CHEMBL479328 | -6.700 |
| CHEMBL1080242 | -8.000 |
| CHEMBL506889 | -9.700 |
| CHEMBL3337754 | -6.200 |
| CHEMBL2386513 | -7.500 |
| CHEMBL1223695 | -5.800 |
| CHEMBL390320 | -6.200 |
| CHEMBL399672 | -7.100 |
| CHEMBL147067 | -7.300 |
| CHEMBL1084170 | -8.100 |
| CHEMBL504850 | -7.200 |
| CHEMBL472877 | -8.100 |
| CHEMBL92528 | -7.200 |
| CHEMBL466164 | -6.500 |
| CHEMBL3233981 | -6.700 |
| CHEMBL424997 | -5.400 |
| CHEMBL592620 | -3.400 |
| CHEMBL87 | -3.500 |
| CHEMBL1834810 | -9.100 |
| CHEMBL442565 | -6.400 |
| CHEMBL519395 | -5.600 |
| CHEMBL14117 | -7.600 |
| CHEMBL1087405 | -7.400 |
| CHEMBL1712170 | -7.000 |
| CHEMBL3348832 | -6.700 |
| CHEMBL517080 | -7.700 |
| CHEMBL575429 | -7.000 |
| CHEMBL1094876 | -7.400 |
| CHEMBL508813 | -6.600 |
| CHEMBL463165 | -6.500 |
| CHEMBL250348 | -4.900 |
| CHEMBL103686 | -6.900 |
| CHEMBL1779811 | -7.700 |
| CHEMBL2312529 | -6.500 |
| CHEMBL1079033 | -8.4 |
| CHEMBL1080979 | -7.8 |
| CHEMBL1087720 | -7.1 |
| CHEMBL1092163 | -8 |
| CHEMBL1095333 | -7.4 |
| CHEMBL1096781 | -7.9 |
| CHEMBL1170899 | -6.8 |
| CHEMBL1277662 | -6.9 |
| CHEMBL141117 | -7.8 |
| CHEMBL1456697 | -5.9 |
| CHEMBL1651023 | -6.6 |
| CHEMBL1651024 | -7.8 |
| CHEMBL1651069 | -8 |
| CHEMBL1669578 | -7.1 |
| CHEMBL1689080 | -7.4 |
| CHEMBL1807753 | -8.3 |
| CHEMBL182992 | -7.3 |
| CHEMBL1909923 | -7.7 |
| CHEMBL1915271 | -8.1 |
| CHEMBL1969511 | -8 |
| CHEMBL1972346 | -8 |
| CHEMBL2057716 | -8.3 |
| CHEMBL2087217 | -9.3 |
| CHEMBL2204390 | -8.7 |
| CHEMBL490355 | -8.8 |
| CHEMBL2334478 | -7.6 |
| CHEMBL2334889 | -8 |
| CHEMBL2337574 | -7.5 |
| CHEMBL2385627 | -8.3 |
| CHEMBL2386704 | -7.9 |
| CHEMBL2431871 | -7.4 |
| CHEMBL251229 | -6.9 |
| CHEMBL253688 | -6.8 |
| CHEMBL274510 | -8.1 |
| CHEMBL29711 | -8.8 |
| CHEMBL3093767 | -8.9 |
| CHEMBL325752 | -6.5 |
| CHEMBL3287734 | -8 |
| CHEMBL330320 | -7.6 |
| CHEMBL399742 | -6.3 |
| CHEMBL423178 | -6.1 |
| CHEMBL430628 | -7.8 |
| CHEMBL446639 | -8.3 |
| CHEMBL451784 | -5.7 |
| CHEMBL453193 | -4.8 |
| CHEMBL453563 | -8.1 |
| CHEMBL457149 | -5.9 |
| CHEMBL459615 | -7.9 |
| CHEMBL463464 | -8 |
| CHEMBL463760 | -8.1 |
| CHEMBL463922 | -5.9 |
| CHEMBL464176 | -6.4 |
| CHEMBL464481 | -7 |
| CHEMBL465444 | -7.7 |
| CHEMBL465475 | -7.7 |
| CHEMBL465969 | -7.2 |
| CHEMBL478951 | -7.7 |
| CHEMBL478952 | -8.3 |
| CHEMBL485479 | -8.6 |
| CHEMBL485810 | -7.9 |
| CHEMBL487996 | -7.7 |
| CHEMBL496046 | -7.6 |
| CHEMBL504463 | -7.8 |
| CHEMBL511568 | -6.7 |
| CHEMBL514518 | -7.6 |
| CHEMBL517016 | -7.7 |
| CHEMBL517338 | -8.5 |
| CHEMBL517700 | -8.2 |
| CHEMBL575533 | -6.4 |
| CHEMBL589343 | -7.6 |
| CHEMBL62448 | -8.4 |
| CHEMBL1214423 | -8.5 |
| CHEMBL66466 | -8.9 |
| CHEMBL218105 | -7 |
| CHEMBL563557 | -8.4 |
| CHEMBL266195 | -6.9 |
| CHEMBL456 | -6.6 |
| CHEMBL86416 | -7.3 |
| CHEMBL503 | -7.9 |
| CHEMBL136356 | -7.5 |
| CHEMBL88985 | -7.5 |
| Molnupiravir | -7.7 |
| paxlovid | -7.5 |

Supplementary table 3c. Binding affinity of Human ACE2 (PDB ID: 1R4L) and phytochemicals.

| **Chembbl id** | **Affinity(k cal/mol)** |
| --- | --- |
| CHEMBL487789 | -9.7 |
| CHEMBL493857 | -9 |
| CHEMBL1835614 | -5.5 |
| CHEMBL520327 | -8.7 |
| CHEMBL3183581 | -6.1 |
| CHEMBL45763 | -5.4 |
| CHEMBL2271696 | -6.9 |
| CHEMBL2252768 | -8 |
| CHEMBL2204371 | -7 |
| CHEMBL415768 | -7.6 |
| CHEMBL516538 | -6.7 |
| CHEMBL4216332 | -7.8 |
| CHEMBL464574 | -6.5 |
| CHEMBL464817 | -8.5 |
| CHEMBL2386323 | -8.1 |
| CHEMBL477778 | -8.2 |
| CHEMBL1080598 | -6 |
| CHEMBL563681 | -9 |
| CHEMBL508961 | -7.5 |
| CHEMBL501870 | -5.6 |
| CHEMBL503867 | -7 |
| CHEMBL383410 | -7.5 |
| CHEMBL557501 | -9.7 |
| CHEMBL3185643 | -4.6 |
| CHEMBL1160785 | -5.6 |
| CHEMBL226683 | -5.9 |
| CHEMBL2227793 | -7.8 |
| CHEMBL454333 | -8.3 |
| CHEMBL249454 | -9.8 |
| CHEMBL519395 | -7.2 |
| CHEMBL2312529 | -4.1 |
| CHEMBL517080 | -8.7 |
| CHEMBL3184590 | -4.9 |
| CHEMBL87 | -5.6 |
| CHEMBL466164 | -6.2 |
| CHEMBL1223695 | -5.6 |
| CHEMBL506889 | -5.6 |
| CHEMBL463165 | -8.8 |
| CHEMBL2386513 | -7.1 |
| CHEMBL14117 | -5.1 |
| CHEMBL361362 | -7.8 |
| CHEMBL424997 | -8.8 |
| CHEMBL1080242 | -8.2 |
| CHEMBL479328 | -9.7 |
| CHEMBL92528 | -8.3 |
| CHEMBL1087405 | -7.6 |
| CHEMBL1094876 | -6.2 |
| CHEMBL1084170 | -7.8 |
| CHEMBL3348832 | -5.4 |
| CHEMBL3337754 | -4.3 |
| CHEMBL250348 | -7.4 |
| CHEMBL399672 | -7.9 |
| CHEMBL472877 | -6.5 |
| CHEMBL3357563 | -5.6 |
| CHEMBL1712170 | -7.7 |
| CHEMBL147067 | -7.3 |
| CHEMBL504850 | -7.5 |
| CHEMBL1834810 | -7 |
| CHEMBL575429 | -7 |
| CHEMBL1779811 | -6.7 |
| CHEMBL508813 | -7.5 |
| CHEMBL442565 | -5.5 |
| CHEMBL390320 | -6.2 |
| CHEMBL103686 | -4.2 |
| CHEMBL463974 | -6.6 |
| CHEMBL592620 | -5.7 |
| CHEMBL3233981 | -8.3 |
| CHEMBL62448 | -8.6 |
| CHEMBL1214423 | -10.7 |
| CHEMBL66466 | -6.1 |
| CHEMBL218105 | -7.9 |
| CHEMBL563557 | -5.5 |
| CHEMBL266195 | -6.1 |
| CHEMBL456 | -7.7 |
| CHEMBL86416 | -6 |
| CHEMBL503 | -8 |
| CHEMBL136356 | -8 |
| CHEMBL88985 | -8.6 |
| CHEMBL1080979 | -7.6 |
| CHEMBL1087720 | -9.3 |
| CHEMBL1092163 | -8.2 |
| CHEMBL1095333 | -8.5 |
| CHEMBL1096781 | -7 |
| CHEMBL1170899 | -7.7 |
| CHEMBL1277662. | -5.6 |
| CHEMBL141117 | -8.4 |
| CHEMBL1456697 | -7.3 |
| CHEMBL1651023 | -8.3 |
| CHEMBL1651024 | -8.2 |
| CHEMBL1651069. | -7.6 |
| CHEMBL1669578 | -7.8 |
| CHEMBL1689080. | -8.6 |
| CHEMBL1807753 | -7.7 |
| CHEMBL182992 | -8.3 |
| CHEMBL1909923 | -6.6 |
| CHEMBL1915271 | -8.2 |
| CHEMBL1969511 | -7.8 |
| CHEMBL1972346 | -7.7 |
| CHEMBL2057716 | -8.3 |
| CHEMBL2087217 | -8.2 |
| CHEMBL2204390 | -8.1 |
| CHEMBL223942 | -7.7 |
| CHEMBL2334478 | -8.5 |
| CHEMBL2334889 | -8.4 |
| CHEMBL2337574 | -8.2 |
| CHEMBL2385627 | -9 |
| CHEMBL2386704 | -8.5 |
| CHEMBL2431871 | -9.5 |
| CHEMBL251229. | -7 |
| CHEMBL253688 | -7.8 |
| CHEMBL274510 | -7.6 |
| CHEMBL29711 | -8.2 |
| CHEMBL3093767 | -7.9 |
| CHEMBL325752 | -7.8 |
| CHEMBL3287734 | -8.4 |
| CHEMBL330320. | -8.8 |
| CHEMBL399742 | -6.5 |
| CHEMBL423178 | -4.6 |
| CHEMBL430628 | -7.7 |
| CHEMBL446639. | -8.6 |
| CHEMBL451784 | -5.2 |
| CHEMBL453193 | -4.9 |
| CHEMBL453563 | -8.9 |
| CHEMBL457149 | -5.4 |
| CHEMBL459615 | -7.4 |
| CHEMBL463464 | -5.8 |
| CHEMBL463760 | -6 |
| CHEMBL463922 | -7 |
| CHEMBL464176 | -7.9 |
| CHEMBL464481 | -8.1 |
| CHEMBL465444 | -9 |
| CHEMBL465475 | -7.8 |
| CHEMBL465969 | -7.8 |
| CHEMBL478951 | -7.6 |
| CHEMBL478952 | -8.3 |
| CHEMBL485479 | -8.5 |
| CHEMBL485810. | -8.5 |
| CHEMBL487996 | -7.2 |
| CHEMBL496046 | -7.4 |
| CHEMBL504463 | -7.4 |
| CHEMBL511568. | -8.4 |
| CHEMBL514518 | -8.6 |
| CHEMBL517016 | -8.6 |
| CHEMBL517338 | -8.8 |
| CHEMBL517700 | -8.8 |
| CHEMBL550232 | -7.9 |
| CHEMBL575533. | -7.8 |
| CHEMBL589343 | -8 |
| Molnupiravir | -7.6 |
| paxlovid | -7 |
