# Supplementary material for: In Silico Identification and Analysis of Potentially Bioactive Antiviral Phytochemicals against SARS-CoV-2: A Molecular Docking and Dynamics Simulation Approach
Source: Biomed Res Int. 2023 May 11;2023:5469258. doi: 10.1155/2023/5469258 (PMC10195178; doi:10.1155/2023/5469258)
Supplement: Supplementary 4 — Supplementary Table 4a: XP Gscore and MM-GBSA values between the main protease (PDB ID: 7NT3) and phytochemicals and control drugs. Supplementary Table 4b: XP Gscore and MM-GBSA values between the NSP3 (PDB ID: 7KQP) and the phytochemicals and control drugs. Supplementary Table 4c: XP Gscore and MM-GBSA values between the human ACE2 receptor (PDB ID: 1R4L) and phytochemicals and control drugs. [file 5469258.f4.docx]

Supplementary table 4a. XP gscore and MM-GBSA values between the main protease (PDB ID: 7NT3) and phytochemicals and control drugs

| **Chembbl id** | **Glide score** | **mmgbsa** | **No of H bonds** | **Interacting H bonds** |
| --- | --- | --- | --- | --- |
| CHEMBL1079033 | -1.187 | -16.6 | 1 | Gln110(2.53014) |
| CHEMBL1080979 | -5.458 | -40.52 | 2 | Gly71, Lys97(2.32119) |
| CHEMBL1087720 | -5.317 | -52.92 | 2 | His246(1.9709), Phe294 |
| CHEMBL1092163 | -4.675 | -55.23 | 4 | Gln110(3.24072), Ile249, Asp295 |
| CHEMBL1095333 | -3.285 | -31.03 | 1 | Phe294(2.2454) |
| CHEMBL1170899 | -1.713 | -13.61 | 0 | None |
| CHEMBL1651023 | -3.939 | -25.51 | 1 | Thr25(2.75405) |
| CHEMBL1651024 | -4.691 | -34.2 | 2 | Glu240, His246(2.33965) |
| CHEMBL1651069 | -4.077 | -36.8 | 0 | None |
| CHEMBL1669578 | -5.353 | -53.87 | 1 | Asn142(2.20786) |
| CHEMBL1689080 | -3.591 | -19.76 | 0 | None |
| CHEMBL1807753 | -5.331 | -36.04 | 2 | Gln110(1.88089) |
| CHEMBL182992 | -6.274 | -54.3 | 1 | Leu287(2.49856) |
| CHEMBL1909923 | -6.588 | -41.95 | 1 | Thr24 (3.054) |
| CHEMBL1915271 | -3.953 | -32.31 | 2 | Thr292(1.88018), Pro108 |
| CHEMBL1969511 | -5.88 | -54.97 | 1 | Asp289(2.06923) |
| CHEMBL1972346 | -6.599 | -43.41 | 1 | His246(2.93357) |
| CHEMBL2057716 | -4.03 | -29.97 | 2 | Arg131(2.40632), Asn238 |
| CHEMBL2087217 | -4.752 | -42.07 | 3 | His246(2.53308), Pro108 |
| CHEMBL2204390 | -3.549 | -52.21 | 3 | His246(2.07766), Pro293, Pro108 |
| CHEMBL223942 | -3.156 | -33.3 | 0 | None |
| CHEMBL2334478 | -5.19 | -38.11 | 5 | Thr26(2.02144), His41, Thr24 |
| CHEMBL2334889 | -3.626 | -40.25 | 0 | None |
| CHEMBL2337574 | -3.582 | -26.71 |  | No ligand definition was found |
| CHEMBL2385627 | -3.665 | -37.94 | 0 | None |
| CHEMBL2386704 | -0.908 | -1.712 | 2 | Thr24, Thr25(2.3169) |
| CHEMBL2431871 | -4.095 | 35.05 | 1 | Phe294(2.0667) |
| CHEMBL251229 | -4.43 | 41.94 | 4 | Thr199(2.08202), Leu287, Tyr237, |
| CHEMBL253688 | -3.467 | -45.47 | 2 | Thr26 (2.12068), Gly143 |
| CHEMBL274510 | 5.663 | -39.087 | 3 | His246(2.24627), Gln110 |
| CHEMBL29711 | -3.287 | -20.22 | 1 | Gln107(2.35592) |
| CHEMBL3093767 | -5.715 | -29.28 | 2 | Gln110(2.16019) |
| CHEMBL325752 | -4.5 | -48.88 | 3 | Gln (2.14799) |
| CHEMBL3287734 | -5.72 | -43 | 2 | Phe294, Gln107(2.80621) |
| CHEMBL330320 | -4.21 | -35.94 | 1 | Phe294(2.19553) |
| CHEMBL399742 | -3 | -26.39 | 0 | None |
| CHEMBL423178 | -3.88 | -44.4 | 3 | Arg131(2.77332), Glu290 |
| CHEMBL430628 | -4.228 | -37.21 | 3 | Asn221(2.18444), Lys269, Thr225 |
| CHEMBL446639 | -5.851 | -50.23 | 0 | None |
| CHEMBL451784 | -4.37 | -38.9 | 3 | Arg131,Asp289, Leu287(1.95447) |
| CHEMBL453193 | -4.44 | -34.31 | 0 | None |
| CHEMBL453563 | -5.7 | -52.45 | 3 | Lys269(1.96598), Gln273, Thr225 |
| CHEMBL457149 | -3.83 | 36.37 | 2 | Asn151,Asn203(2.25925) |
| CHEMBL459615 | -3.73 | -34.94 | 2 | Gln189(2.35899), Thr25 |
| CHEMBL463464 | -4.223 | -37.43 | 4 | Gln107, His246 (2.0442), Pro108 |
| CHEMBL463760 | -4.783 | -50.52 | 5 | His246(1.99085), Thr292, Gln107, Pro108, Ile249 |
| CHEMBL463922 | -4.35 | -31.99 | 3 | Thr199, Tyr239, Tyr237(1.94715) |
| CHEMBL464176 | -2.095 | -28.98 | 2 | Thr26 (2.29468), Gln189 |
| CHEMBL464481 | 1.007 | -23.55 | 2 | Gln110, Thr292(2.38632) |
| CHEMBL465444 | -3.683 | -29.88 | 3 | Gln110, His246 (2.04549), Asp245 |
| CHEMBL465475 | -4.202 | -34.07 | 3 | Asn221, Lys269 (2.2085), Thr225 |
| CHEMBL465969 | -4.815 | -40.07 | 1 | Thr199(2.0844) |
| CHEMBL478951 | -3.47 | -37.4 | 2 | Thr26, Thr24 (1.93214) |
| CHEMBL478952 | -3.323 | -28.46 | 0 | None |
| CHEMBL485479 | -3.517 | -36.89 | 0 | None |
| CHEMBL485810 | -2.744 | -31.24 | 5 | His41 (2.27843), Asn142, Gly143, Cys145, Asn142 |
| CHEMBL487996 | -4.862 | -31.61 | 2 | Asn119 (2.1768), Gly143 |
| CHEMBL496046 | -2.239 | -21.18 | 1 | Gln110(2.87241) |
| CHEMBL504463 | -5.233 | -44.94 | 0 | None |
| CHEMBL511568 | -3.712 | -37.29 | 0 | None |
| CHEMBL514518 | -4.342 | -26.63 | 3 | Arg131, Leu287(1.78246), Tyr 237 |
| CHEMBL517016 | -3.217 | -30.01 | 1 | Glu240(2.65837) |
| CHEMBL517338 | -1.191 | -23.71 | 2 | Phe294, Gln110(2.15289) |
| CHEMBL517700 | -5.459 | -32.64 | 1 | Gln107(3.55155) |
| CHEMBL550232 | -4.555 | -356.39 | 2 | Thr26(2.08768), Asn142 |
| CHEMBL575533 | -5.507 | -52.36 | 2 | The243(2.05114), Phe294 |
| CHEMBL589343 | 4.624 | -36.52 | 2 | Gln110, Phe294(2.3362) |
| CHEMBL266195 | -3.55 | -41.52 | 4 | GLN110 (2.31354),THR111,GLN110 |
| CHEMBL456 | -4.821 | -34.44 | 3 | HIS41 (2.36465),SER46,CYS145 |
| CHEMBL503 | -6.01 | -52.85 | 1 | ARG131(2.30102) |
| CHEMBL136356 | -2.91 | -11.56 | 1 | GLN189 (1.95033) |
| CHEMBL88985 | -3.88 | -25.3 | 1 | GLN189 (1.90695) |
| CHEMBL563557 | -4.96 | -42.3 | 0 | None |
| CHEMBL86416 | -3.472 | -28.04 | 1 | TYR237 (2.14629) |
| CHEMBL62448 | -2.76 | -35.39 | 1 | HIS246 (2.24381) |
| CHEMBL1214423 | -5.24 | -45.18 | 3 | TYR237,GLN273 (2.14278) |
| CHEMBL66466 | 0.78 | -41.75 | 2 | HIS246 (2.22959) |
| CHEMBL218105 | -4.1 | -25.27 | 2 | ARG131 (2.3697) |
| CHEMBL249454 | -7.05 | -52.74 | 1 | Phe294(3.3362) |
| CHEMBL477778 | -7.02 | -48.15 | 2 | PHE294 (2.14386) |
| CHEMBL557501 | -6.798 | -51.61 | 0 | None |
| CHEMBL4216332 | -6.496 | -47.83 | 0 | None |
| CHEMBL516538 | -5.445 | -50 | 1 | PRO293 |
| CHEMBL508813 | -5.265 | -33.54 | 1 | LYS269 (1.80854) |
| CHEMBL383410 | -5.225 | -32.32 | 1 | HIS41 (3.321) |
| CHEMBL415768 | -5.206 | -34.82 | 2 | GLN110 (2.88), PRO108 |
| CHEMBL1080242 | -5.218 | -48.15 | 2 | GLN110 (2.22), THR292 |
| CHEMBL361362 | -5.119 | -41.41 | 3 | ARG131, LEU287 (2.41991), LYS137 |
| CHEMBL3233981 | -4.982 | -34.53 | 2 | HIS41 (1.69478), GLY143 |
| CHEMBL399672 | -4.924 | -44.45 | 2 | THR199, LEU287 (2.345) |
| CHEMBL479328 | -4.675 | -37.17 | 1 | GLN110 (2.08499) |
| CHEMBL1834810 | -4.645 | -47.57 | 5 | LYS102,LYS102, GLN110,GLN110,SER158 (2.14347) |
| CHEMBL2386323 | -5.156 | -35.73 | 0 | None |
| CHEMBL454333 | -4.399 | -49.12 | 5 | CYS145, GLN189 (2.318), GLY143,THR26, ASN142 |
| CHEMBL1087405 | -4.387 | -30.53 | 0 | None |
| CHEMBL463165 | -4.265 | -38.69 | 2 | HIS246, GLN110 (2.55) |
| CHEMBL92528 | -4.264 | -39.65 | 0 | None |
| CHEMBL147067 | -4.232 | -40.58 | 1 | PHE294 (1.91769) |
| CHEMBL87 | -4.21 | -35.94 | 1 | THR199 (2.27648) |
| CHEMBL424997 | -4.183 | -32.34 | 1 | PHE294 (2.235) |
| CHEMBL520327 | -4.107 | -38.8 | 3 | GLN110, GLN110, PHE294 (2.28389) |
| CHEMBL466164 | -4.046 | -15.77 | 1 | ASN221(2.37813) |
| CHEMBL504850 | -3.976 | -37.98 | 1 | THR25 (2.236) |
| CHEMBL1080242 | -5.185 | -33.7 | 2 | GLN110 (2.22115), THR292 |
| CHEMBL1712170 | -3.747 | -42.26 | 3 | GLN273, GLN273 (2.481), THR224 |
| CHEMBL2252768 | -3.601 | -50.12 | 3 | THR199, THR199 ,THR199 (2.03774) |
| CHEMBL2386323 | -3.93 | -36.54 | 0 | None |
| CHEMBL487789 | -3.574 | -20.85 | 0 | None |
| CHEMBL517080 | -3.567 | -40.7 | 2 | ARG131, ARG131 (2.537) |
| CHEMBL563681 | -3.475 | -25.92 | 2 | THR26 (1.78929), HIS41 |
| CHEMBL250348 | -3.19 | -28.81 | 1 | PHE294(3.07878) |
| CHEMBL504850 | -4.778 | -9.2 | 0 | None |
| CHEMBL464817 | -3.119 | -27.15 | 2 | GLN110(2.59311), GLN110 |
| CHEMBL2386323 | -4.052 | -18.14 | 0 | None |
| CHEMBL1712170 | -2.113 | -35.95 | 1 | GLN273 (2.48193) |
| CHEMBL1712170 | -3.553 | -30.98 | 1 | GLN273 (2.48193) |
| CHEMBL2252768 | -3.429 | -41.31 | 3 | THR199, THR199, THR199 (2.03) |
| CHEMBL464574 | -0.657 | -42.39 | 1 | PRO108 |
| CHEMBL493857 | 0.122 | -34.45 | 3 | GLN110,HIS246 (2.26284),THR292: |
| CHEMBL517080 | -1.96 | -18.89 | 1 | Thr24 (1.93214) |
| Molnupiravir | -5.035 | -43.48 | 3 | ASP197, THR199 (2.04463), LEU287 |
| Paxlovid | -5.185 | -43.34 | 6 | THR26, HIS41, ASN119,ASN142,GLY143(1.98365),CYS145 |

Supplementary table 4b. XP gscore and MM-GBSA values between the NSP3 (PDB ID: 7KQP) and the phytochemicals and control drugs

| **Chembbl id** | **Glide score** | **MMGBSA** | **No of H bonds** | **Interacting H bonds** |
| --- | --- | --- | --- | --- |
| CHEMBL266195 | -3.592 | -41.86 | 1 | LEU126 |
| CHEMBL456 | -4.408 | -23.74 | 1 | PHE156(2.20111) |
| CHEMBL503 | -2.848 | -31.27 | 3 | ASP157(1.93788),ALA38,GLY48 |
| CHEMBL136356 | -1.561 | 1.16 | 2 | ILE23(2.05023),PHE156 |
| CHEMBL88985 | -2.102 | -9.67 | 2 | ILE23(2.05329),PHE156 |
| CHEMBL563557 | -8.069 | -44.12 | 0 | None |
| CHEMBL86416 | -2.354 | -7.74 | 1 | ILE23(2.04309) |
| CHEMBL62448 | -1.995 | -14.28 | 4 | VAL49,SER128,ALA129,GLY130(2.41715) |
| CHEMBL1214423 | -3.704 | -24.2 | 2 | ILE23,UNL1:H(2.16045) |
| CHEMBL66466 | -2.365 | -8.48 | 4 | VAL49,SER128,ALA129(2.26587),GLY130 |
| CHEMBL218105 | -1.583 | -35.26 | 4 | ASN40,GLY46(2.3554),GLY47,GLY130 |
| CHEMBL1079033 | -1.93 | -21.76 | 1 | VAL49 |
| CHEMBL1080979 | -3.85 | -30.97 | 3 | LEU126 (2.44917), PHE156, ASP157 |
| CHEMBL1092163 | -1.73 | -25.22 | 3 | ASP22, ALA38, VAL49 (2.93323) |
| CHEMBL141117 | -4.04 | -21.4 | 5 | VAL49 (2.26389), SER128, ALA129, GLY48, ALA38 |
| CHEMBL1807753 | -3.83 | -21.02 | 2 | LEU126, PHE132 (2.00156) |
| CHEMBL1969511 | -3.95 | -27.21 | 4 | VAL49, ALA50 (2.00252), GLY47, ALA154 |
| CHEMBL1972346 | -6.4 | -40.77 | 2 | PHE156 (2.25309), ALA154 |
| CHEMBL2204390 | -2.96 | -23.21 | 3 | ASN40 (2.1242), ASN99, GLY48 |
| CHEMBL2334889 | -2.3 | -21.25 | 0 | None |
| CHEMBL2385627 | -0.97 | -13.76 | 1 | LEU126 |
| CHEMBL2431871 | -0.57 | -22.01 | 0 | None |
| CHEMBL251229 | -2.9 | -9.77 | 1 | LEU126 |
| CHEMBL253688 | -2.24 | -10.51 | 1 | LEU126 |
| CHEMBL274510 | -5.34 | -21.88 | 2 | ILE131 (1.86631), GLY48 |
| CHEMBL29711 | -3.28 | -3.28 | 3 | ILE23, VAL49, ILE131 (2.17304) |
| CHEMBL3093767 | -4.04 | -20.32 | 4 | SER128, ALA129, GLY130 (2.42718), GLY48 |
| CHEMBL325752 | -4.82 | -24.17 | 6 | PHE156, PHE156, ASP157 (2.0379), ALA129, ALA154, ALA129 |
| CHEMBL3287734 | -3.02 | -17.76 | 1 | LEU126 |
| CHEMBL430628 | -3.19 | -11.01 | 4 | LEU126, PHE156 (1.97579), ALA154, LEU126 |
| CHEMBL446639 | -5.75 | -41.97 | 3 | ALA129, GLY130 (2.2329), PHE156 |
| CHEMBL453563 | -3.98 | -26.091 | 2 | PHE156 (1.99081), VAL155 |
| CHEMBL459615 | -4.47 | -35.96 | 2 | LEU126, PHE156 (2.06612) |
| CHEMBL463464 | -2.82 | -22.7 | 4 | SER128 (2.69289), ALA129, ASP157, ALA154 |
| CHEMBL463760 | -3.51 | -24.59 | 5 | SER128, ALA129, GLY130 (2.27633), ASP157, ALA154 |
| CHEMBL465444 | -3.42 | -16.42 | 0 | None |
| CHEMBL465475 | -0.34 | -8.24 | 3 | ILE23, LEU126 (1.94146), LEU126 |
| CHEMBL478951 | -3.67 | -20.24 | 4 | ALA129, GLY130 (2.35227), PHE156, ALA154 |
| CHEMBL478952 | -3.8 | -20.26 | 4 | ILE131 (2.44738), GLY48, GLY130, VAL49 |
| CHEMBL485479 | -3.88 | -21.66 | 3 | GLY48, GLY48, GLY130 (3.24601) |
| CHEMBL485810 | -1.81 | -8.39 | 3 | PHE156 (2.22546), ASP157, ALA129 |
| CHEMBL487996 | -3.74 | -12.22 | 3 | PHE156 (2.06954), ASP22, VAL49 |
| CHEMBL504463 | -5.4 | -44.64 | 4 | SER128, ALA129, GLY130 (2.17615), LEU126 |
| CHEMBL517016 | -1.58 | -57.54 | 2 | ILE23 (2.27496), PHE156 |
| CHEMBL517338 | -3.65 | -19.12 | 0 | None |
| CHEMBL517700 | -2.57 | -12.53 | 2 | GLY48, ALA154 (3.51685) |
| CHEMBL550232 | -4.23 | -14.21 | 4 | VAL49, SER128 (2.68999), LEU126, LEU126 |
| CHEMBL517080 | -1.747 | -11.56 | 1 | LEU126 |
| CHEMBL1779811 | -3.647 | -29.17 | 4 | ALA50 (1.86463), ALA129, GLY30, ALA129 |
| CHEMBL1834810 | -5.034 | -36.91 | 3 | ASN40, GLY47, LEU126 (2.06339) |
| CHEMBL14117 | -7.659 | -37.28 | 1 | PHE132 |
| CHEMBL490355 | -9.563 | -46.31 | 1 | ALA129 |
| CHEMBL516538 | -3.775 | -39.34 | 4 | PHE156, ASP157, LEU126 (2.21913), GLY48 |
| CHEMBL3185643 | -2.456 | -48.67 | 4 | SER128, SER128, ALA129, PHE132 (1.80896) |
| CHEMBL464817 | -2.12 | -9.97 | 1 | LYS44 |
| CHEMBL487789 | -0.799 | -10.94 | 3 | SER128 (2.0045), GLY48, PHE132 |
| CHEMBL226683 | -9.161 | -40.05 | 0 | None |
| CHEMBL493857 | -1.245 | -12.26 | 2 | GLY48 (2.96684), ALA154 |
| CHEMBL2386323 | -3.006 | -23.04 | 2 | ILE23 (2.18813), ALA154 |
| CHEMBL2271696 | -4.2 | -19.06 | 2 | ILE23 (2.31342), PHE156 |
| CHEMBL563681 | -1.554 | -15.01 | 3 | ALA129, GLY130, PHE156 (2.07929) |
| CHEMBL4216332 | -5.273 | -24.36 | 6 | GLY47, SER128, ALA129, GLY130, LEU126 (1.90716), LEU126 |
| CHEMBL1835614 | -4.851 | -26.35 | 0 | None |
| CHEMBL2204371 | -4.413 | -15.82 | 3 | ILE23 (2.32179), ALA154, PHE156 |
| CHEMBL2227793 | -2.088 | 28.24 | 2 | LEU126 (3.55887), ASP22 |
| CHEMBL454333 | -3.111 | -26.31 | 4 | ILE23 (2.49462), LEU126, GLY47, VAL49 |
| CHEMBL415768 | -2.981 | -23.6 | 4 | SER128, ALA129, GLY130 (1.91283), PHE156 |
| CHEMBL464574 | -3.396 | -20.04 | 4 | ILE23, PHE156, ASP157, :UNL1:H - :UNL1:O (1.8863) |
| CHEMBL463974 | -4.627 | -33.14 | 2 | ASN40, ASN40 (2.40258) |
| CHEMBL361362 | -3.531 | -39.11 | 1 | LEU126 |
| CHEMBL1080242 | -8.909 | -52.47 | 3 | ILE23 (2.22239), SER128, GLY47 |
| CHEMBL506889 | -1.555 | -9.99 | 0 | None |
| CHEMBL2386513 | -2.25 | -12.28 | 1 | VAL49 |
| CHEMBL1084170 | -3.448 | -4.3 | 1 | VAL49 |
| CHEMBL472877 | -3.985 | -18.02 | 0 | None |
| Molnupiravir | -7.604 | -40.45 | 6 | ASN40, GLY47, VAL49, ALA50, LYS44, ALA38 (1.90623) |
| Paxlovid | -2.727 | -20.25 | 4 | LYS158,LEU160,TYR161(1.23877) |

Supplementary table 4c. XP gscore and MM-GBSA values between the Human ACE2 (PDB ID: 1R4L) and phytochemicals and control drugs.

| **Chembbl id** | **Glide score** | **MMGBSA** | **No of H-bonds** | **Interacting H-bonds** |
| --- | --- | --- | --- | --- |
| CHEMBL266195 | -3.941 | -37.47 | 4 | GLU208 ; GLY205 (2.17975) ; GLU208 ; GLN98. |
| CHEMBL456 | -5.625 | -27.59 | 3 | GLN98, GLU208 (1.87196), GLY205 |
| CHEMBL503 | -7.482 | -32.88 | 0 | None |
| CHEMBL136356 | -6.148 | -35.73 | 1 | GLY211(2.67667) |
| CHEMBL88985 | -4.78 | -32.54 | 1 | GLY211 (2.68052) |
| CHEMBL563557 | -6.051 | -22.02 | 2 | SER47; ASP350 (2.85032) |
| CHEMBL86416 | -4.796 | -17.4 | 1 | ARG518 (2.51645) |
| CHEMBL62448 | -2.843 | -42.71 | 4 | ASN210, VAL212 (2.12726), GLN98, GLY205 |
| CHEMBL1214423 | -5.887 | -38.52 | 1 | LEU392 (3.34741) |
| CHEMBL66466 | -3.059 | 13.73 | 3 | GLN98 (2.2995), TRP203, TRP203 |
| CHEMBL218105 | -6.667 | -35.26 | 2 | ASP206 (3.53431); GLU208 |
| CHEMBL1079033 | -2.32 | -23.56 | 3 | TYR515 (2.02852); HIS378; GLU402 |
| CHEMBL1087720 | -5.7 | -40.59 | 2 | ASN210 (2.36446); VAL212 |
| CHEMBL1092163 | -4.76 | -46.4 | 1 | ALA396 (3.51859) |
| CHEMBL1095333 | -3.54 | -22.93 | 5 | ASN394; LYS562; LYS562 (2.15884); LYS562; GLU208 |
| CHEMBL1170899 | -5.76 | -26.67 | 0 | None |
| CHEMBL1651023 | -5.29 | -26.5 | 1 | ASP206 (2.23899) |
| CHEMBL1651024 | -3.98 | -28.35 | 3 | THR347; ASP350; ALA348 (2.12505) |
| CHEMBL1669578 | -4.06 | -37.18 | 1 | PRO346 (3.58381) |
| CHEMBL1689080 | -4.63 | -25.5 | 1 | ASN210 (2.22983) |
| CHEMBL1807753 | -6.79 | -34.92 | 2 | THR347(2.84025) ; TYR515 |
| CHEMBL1909923 | -6.69 | -50.17 | 3 | TRP566 (2.85943); PRO565; ALA396 |
| CHEMBL1915271 | -4.74 | -9.44 | 4 | THR347; ALA348 (2.25276); TRP349; TYR510 |
| CHEMBL1969511 | -3.36 | -38.96 | 1 | HIS345 (1.98993) |
| CHEMBL1972346 | -6.65 | -35.86 | 5 | GLU564; ASP206 (2.32321); LYS562; LYS562; GLU208 |
| CHEMBL2057716 | -5.47 | -26.35 | 1 | GLU564 (2.87708) |
| CHEMBL2087217 | -5.55 | -42.19 | 5 | ASN154; ASN277; LYS363 (2.36374); LYS363; ASN277 |
| CHEMBL2204390 | -4.92 | -46.2 | 2 | ASP206 (2.53074); GLN102 |
| CHEMBL223942 | -4.74 | -21.19 | 0 | None |
| CHEMBL2334478 | -4.09 | -22.43 | 3 | LYS363; ASP367 (1.77099) |
| CHEMBL2334889 | -5 | -48.76 | 1 | PRO565 (3.50093) |
| CHEMBL2337574 | -3.51 | -22.47 | 0 | None |
| CHEMBL2385627 | -3.78 | -18.99 | 4 | GLN102 (2.29396); GLY211; GLN98; ASN210 |
| CHEMBL2386704 | 0.58 | -14.61 | 1 | GLU208 (3.35753) |
| CHEMBL2431871 | -4.08 | 10.35 | 2 | THR371; ASP269 (1.73802) |
| CHEMBL253688 | -4.68 | -38.78 | 2 | ASN149 ; HIS345 (2.6082) |
| CHEMBL29711 | -4.99 | -26.04 | 4 | TYR385 (2.28999), HIS401, SER44, ASP350 |
| CHEMBL3093767 | -3.66 | -29.45 | 3 | SER124; TYR510(2.04025); ASN121 |
| CHEMBL325752 | -5.28 | -36.01 | 2 | GLN98 (2.07383); LYS562 |
| CHEMBL3287734 | -5.2 | -45.1 | 3 | LYS562(2.32514); VAL209; ASP206 |
| CHEMBL330320 | -5.83 | -24.71 | 0 | None |
| CHEMBL423178 | -3.65 | -36.95 | 0 | None |
| CHEMBL430628 | -5.16 | -40.42 | 3 | GLU208 (1.87974); ASP206; GLU208 |
| CHEMBL446639 | -5 | -45.27 | 2 | GLN98 (2.63788); TYR196 |
| CHEMBL453563 | -5.8 | -32.1 | 1 | ASP206 (2.33074) |
| CHEMBL464176 | -4.16 | -13.38 | 0 | None |
| CHEMBL464481 | 4.46 | -15.96 | 1 | PRO346 (3.43123) |
| CHEMBL465444 | -5.66 | -26.1 | 1 | GLN102 (3.69998) |
| CHEMBL465475 | -3.59 | -18.87 | 1 | GLU208 (2.4323) |
| CHEMBL465969 | -5.89 | -37.88 | 0 | None |
| CHEMBL478952 | -6.13 | -46.02 | 2 | LYS562 (2.19613); LYS562 |
| CHEMBL485479 | -2.85 | -28.22 | 3 | ASN51; THR347 (2.72544); TYR510 |
| CHEMBL485810 | -3.86 | -30.38 | 2 | GLN98; ASP206 (2.14561) |
| CHEMBL511568 | -3.13 | -22.62 | 1 | GLU208 (2.66222) |
| CHEMBL514518 | -4.17 | -24.83 | 1 | GLU208 (1.92813) |
| CHEMBL517016 | -3.92 | -28.45 | 2 | GLN98 (2.16465); GLU564 |
| CHEMBL517338 | -2.2 | -18.07 | 1 | GLU406 (3.33652) |
| CHEMBL517700 | -7 | -42.27 | 4 | ARG514; TYR385 (1.98111); HIS378; ASN394 |
| CHEMBL550232 | -4.63 | -37.22 | 1 | TYR515 (2.99695) |
| CHEMBL575533 | -4.66 | -49.64 | 1 | ASN210 (2.19474) |
| CHEMBL589343 | -2.35 | 23.44 | 0 | None |
| CHEMBL487789 | -4.432 | -14.91 | 1 | HIS345 |
| CHEMBL493857 | -1.092 | -33.86 | 2 | ALA348 (2.20995), ALA348 |
| CHEMBL520327 | -4.21 | -27.91 | 2 | GLN98 (2.22566), LEU392 |
| CHEMBL2252768 | -3.028 | -26.78 | 0 | None |
| CHEMBL2204371 | -6.257 | -33.94 | 5 | ASN210 (1.86088), GLY211, LYS562, GLY205 |
| CHEMBL415768 | -7.295 | -32.24 | 1 | GLU564 |
| CHEMBL4216332 | -7.87 | -63.54 | 3 | ARG273, ARG273, GLU406 (1.84129) |
| CHEMBL464817 | -3.444 | -34.11 | 0 | None |
| CHEMBL2386323 | -5.068 | -27.57 | 2 | SER47 (2.0981), ASN51 |
| CHEMBL477778 | -7.716 | -39.56 | 4 | ASP367, GLU402 (2.64677), HIS505, ARG273 |
| CHEMBL563681 | 0.022 | -31.39 | 2 | THR371 (2.3484), THR371 |
| CHEMBL508961 | -5.656 | -44.38 | 2 | HIS345, THR371 (2.27029) |
| CHEMBL503867 | -3.252 | -33.09 | 0 | None |
| CHEMBL383410 | -4.337 | -17.47 | 2 | ASN210 (2.10555), GLU208 |
| CHEMBL557501 | -4.909 | -28.97 | 2 | TYR196 (3.08692), VAL209 |
| CHEMBL2227793 | -6.266 | -45.38 | 2 | ASN121 (1.8794), ASN51 |
| CHEMBL454333 | -4.542 | -56.67 | 6 | LYS187, ASP206, SER511 (1.83647), SER511, ARG514, ASP206 |
| CHEMBL249454 | -7.48 | -48.29 | 4 | GLU402, GLU402 (2.29003), ALA348, TRP349 |
| CHEMBL519395 | -6.955 | -24.31 | 5 | ARG273, HIS345 (2.4816), HIS345, ASP368, ASP368 |
| CHEMBL517080 | -5.449 | -25.85 | 2 | GLU140 (2.35749), TRP163 |
| CHEMBL463165 | -4.272 | -27.58 | 4 | ASN394, ALA348 (2.04552), ALA348, ASP382 |
| CHEMBL2386513 | -3.696 | -27.63 | 4 | ARG273, ARG518 (2.12767), GLU375, HIS345 |
| CHEMBL361362 | -5.549 | -41.55 | 3 | TYR385, ASN51, TYR385 (1.7689) |
| CHEMBL424997 | 1.628 | -45.95 | 0 | None |
| CHEMBL1080242 | -6.077 | -48.68 | 1 | ALA348 |
| CHEMBL479328 | -4.561 | -26.85 | 1 | ASN210 |
| CHEMBL92528 | -1.965 | 19.97 | 5 | SER47, ASN51, ASN51 (2.0631), THR347, ASN508 |
| CHEMBL1087405 | -4.51 | -32.52 | 3 | ASN149, HIS345, GLU145 (2.19864) |
| CHEMBL1084170 | -6.037 | -31.2 | 4 | GLY211, TRP566 (2.2291), GLU564, ASP206 |
| CHEMBL250348 | -6.465 | -41.78 | 0 | None |
| CHEMBL399672 | -6.465 | -41.78 | 3 | TYR385, HIS401, (2.00491) |
| CHEMBL1712170 | -5.806 | -39.92 | 1 | LYS562 |
| CHEMBL147067 | -4.556 | -50.45 | 2 | ALA348 (1.91681), HIS378 |
| CHEMBL504850 | -4.947 | -16.41 | 5 | GLN98, ASN210, GLY211, LYS562 (1.85689), GLN98 |
| CHEMBL1834810 | -4.075 | -49.8 | 3 | THR371 (2.71991), GLU406, HIS345 |
| CHEMBL575429 | -4.977 | -10.58 | 3 | ASP269, GLU406 (1.80064), THR371 |
| CHEMBL508813 | -4.906 | -34.55 | 0 | None |
| CHEMBL3233981 | -5.6 | -22.45 | 3 | GLU564, LYS562 (2.85168), LYS562 |
| Molnupiravir | -6.02 | -40.53 | 5 | ASP206, HIS378, ASN394, ARG514, LYS562 |
| Paxlovid | -5.679 | -32.02 | 6 | ASP206,ALA348,TRP349ASP350,HIS378,ARG514 |
