# Supplementary material for: In Silico Identification and Analysis of Potentially Bioactive Antiviral Phytochemicals against SARS-CoV-2: A Molecular Docking and Dynamics Simulation Approach
Source: Biomed Res Int. 2023 May 11;2023:5469258. doi: 10.1155/2023/5469258 (PMC10195178; doi:10.1155/2023/5469258)
Supplement: Supplementary 5 — Supplementary Table 5a: coordinates of geometry-optimized ligand (Lovastatin) after molecular docking study. Supplementary Table 5b: coordinates of geometry-optimized ligand (Sulfuretin) after molecular docking study. Supplementary Table 5c: coordinates of geometry-optimized ligand (Grayanoside A) after molecular docking study. [file 5469258.f5.docx]

Table 5a. Coordinates of Geometry-optimized ligand (Lovastatin) after molecular docking study

| Atom | X | Y | Z |
| --- | --- | --- | --- |
| C | -7.049 | 4.105 | -36.8 |
| C | -6.258 | 4.069 | -35.45 |
| C | -8.491 | 3.538 | -36.69 |
| C | -4.821 | 4.646 | -35.644 |
| C | -7.042 | 5.511 | -37.4 |
| C | -9.274 | 3.777 | -37.988 |
| C | -9.348 | 5.255 | -38.398 |
| C | -8.074 | 6.003 | -38.11 |
| C | -4.847 | 5.944 | -36.407 |
| C | -5.864 | 6.329 | -37.186 |
| C | -3.837 | 3.695 | -36.334 |
| C | -10.531 | 5.981 | -37.755 |
| C | -6.263 | 2.666 | -34.794 |
| C | -6.334 | 2.708 | -33.26 |
| C | -5.322 | 1.75 | -32.613 |
| O | -4.206 | 1.616 | -33.509 |
| C | -3.062 | 1.015 | -33.108 |
| O | -2.282 | 0.543 | -33.931 |
| C | -2.679 | 1.137 | -31.656 |
| C | -3.831 | 1.333 | -30.692 |
| C | -4.872 | 2.276 | -31.259 |
| O | -4.426 | 0.07 | -30.4 |
| H | -4.538 | 0.004 | -29.436 |
| O | -9.168 | 4.143 | -35.568 |
| C | -9.904 | 3.312 | -34.778 |
| O | -9.579 | 2.18 | -34.446 |
| C | -11.198 | 3.992 | -34.37 |
| C | -12.335 | 3.419 | -35.213 |
| C | -11.472 | 3.798 | -32.873 |
| C | -12.929 | 3.469 | -32.579 |

Table 5b. Coordinates of Geometry-optimized ligand (Sulfuretin) after molecular docking study

| Atom | X | Y | Z |
| --- | --- | --- | --- |
| O | -18.324 | -1.269 | -2.626 |
| O | -18.49 | 0.834 | -5.474 |
| C | -19.575 | -1.099 | -4.562 |
| C | -19.346 | -1.798 | -3.379 |
| C | -17.853 | -0.173 | -3.342 |
| C | -18.617 | -0.002 | -4.606 |
| C | -16.866 | 0.653 | -2.969 |
| C | -20.556 | -1.463 | -5.464 |
| C | -20.1 | -2.902 | -3.048 |
| C | -21.097 | -3.284 | -3.946 |
| C | -21.331 | -2.581 | -5.143 |
| C | -16.087 | 0.547 | -1.729 |
| C | -15.832 | 1.712 | -0.994 |
| C | -15.071 | 1.642 | 0.165 |
| C | -14.545 | 0.43 | 0.603 |
| C | -14.771 | -0.733 | -0.121 |
| C | -15.537 | -0.67 | -1.29 |
| O | -14.838 | 2.782 | 0.88 |
| H | -14.079 | 3.202 | 0.429 |
| O | -13.804 | 0.435 | 1.752 |
| H | -14.36 | 0.087 | 2.471 |
| O | -21.845 | -4.378 | -3.62 |
| H | -22.749 | -4.092 | -3.406 |

Table 5c. Coordinates of Geometry-optimized ligand (Greyanoside) after molecular docking study

| O | 64.641 | 17.482 | 14.847 |
| --- | --- | --- | --- |
| C | 65.651 | 17.373 | 17.076 |
| C | 65.184 | 15.922 | 17.194 |
| C | 64.721 | 18.136 | 16.123 |
| C | 65.067 | 15.285 | 15.814 |
| C | 64.163 | 16.144 | 14.913 |
| C | 65.201 | 19.574 | 15.884 |
| O | 65.191 | 20.293 | 17.13 |
| C | 64.832 | 21.594 | 17.019 |
| O | 65.349 | 22.543 | 17.581 |
| C | 63.656 | 21.662 | 16.129 |
| C | 62.621 | 22.454 | 16.447 |
| C | 61.398 | 22.535 | 15.64 |
| C | 60.164 | 22.236 | 16.236 |
| C | 58.98 | 22.268 | 15.489 |
| C | 59.053 | 22.587 | 14.125 |
| C | 60.273 | 22.872 | 13.517 |
| C | 61.444 | 22.844 | 14.275 |
| O | 57.73 | 22.003 | 15.982 |
| C | 57.363 | 20.64 | 16.141 |
| O | 57.903 | 22.614 | 13.381 |
| H | 58.108 | 22.332 | 12.476 |
| O | 65.639 | 17.965 | 18.379 |
| H | 65.976 | 17.267 | 18.978 |
| O | 64.201 | 15.525 | 13.62 |
| C | 64.358 | 16.47 | 12.559 |
| C | 64.777 | 15.734 | 11.286 |
| C | 63.98 | 16.181 | 10.08 |
| C | 63.919 | 15.387 | 8.927 |
| C | 63.157 | 15.791 | 7.829 |
| C | 62.451 | 16.987 | 7.883 |
| C | 62.494 | 17.788 | 9.018 |
| C | 63.256 | 17.383 | 10.115 |
| O | 61.713 | 17.345 | 6.792 |
| H | 62.319 | 17.445 | 6.039 |
| O | 66.119 | 15.19 | 18.004 |
| H | 66.993 | 15.353 | 17.594 |
| O | 64.553 | 13.95 | 15.958 |
| H | 63.599 | 14.025 | 15.753 |
