# Supplementary material for: In Silico Identification and Analysis of Potentially Bioactive Antiviral Phytochemicals against SARS-CoV-2: A Molecular Docking and Dynamics Simulation Approach
Source: Biomed Res Int. 2023 May 11;2023:5469258. doi: 10.1155/2023/5469258 (PMC10195178; doi:10.1155/2023/5469258)
Supplement: Supplementary 6 — Supplementary Table 6a: predicted biological activities of Lovastatin. Supplementary table 6b: predicted biological activities of Sulfuretin. Supplementary Table 6c: predicted biological activities of Grayanoside A. [file 5469258.f6.docx]

**Supplementary table 6a**. Predicted biological activities of Lovastatin.

| **Serial no** | **Biological activity** | **Lovastatin** | |
| --- | --- | --- | --- |
|  |  | **Pa** | **Pi** |
| 1 | Antihypercholesterolemic | 0.990 | 0.001 |
| 2 | Lipid metabolism regulator | 0.989 | 0.001 |
| 3 | Hypolipemic | 0.987 | 0.001 |
| 4 | Vasodilator, coronary | 0.969 | 0.003 |
| 5 | HMOX1 expression enhancer | 0.958 | 0.002 |
| 6 | APOA1 expression enhancer | 0.949 | 0.001 |
| 7 | CYP3A4 substrate | 0.935 | 0.004 |
| 8 | CYP3A5 substrate | 0.927 | 0.001 |
| 9 | CYP3A substrate | 0.926 | 0.004 |
| 10 | HMG CoA reductase inhibitor | 0.863 | 0.001 |
| 11 | Antifungal | 0.834 | 0.004 |
| 12 | Reductase inhibitor | 0.811 | 0.000 |
| 13 | Cholesterol synthesis inhibitor | 0.799 | 0.000 |
| 14 | Immunosuppressant | 0.790 | 0.006 |
| 15 | Antieczematic | 0.792 | 0.020 |
| 16 | CYP2D substrate | 0.740 | 0.006 |
| 17 | CYP2D6 substrate | 0.740 | 0.006 |
| 18 | Angiogenesis stimulant | 0.729 | 0.003 |

**Supplementary table 6b**. Predicted biological activities of Sulfuretin.

| **Serial no** | **Biological activity** | **Sulfuretin** | |
| --- | --- | --- | --- |
|  |  | **Pa** | **Pi** |
| 1 | Membrane integrity agonist | 0.946 | 0.004 |
| 2 | Aspulvinone dimethylallyltransferase inhibitor | 0.939 | 0.004 |
| 3 | HIF1A expression inhibitor | 0.905 | 0.005 |
| 4 | Chlordecone reductase inhibitor | 0.902 | 0.005 |
| 5 | 1-Acylglycerol-3-phosphate O-acyltransferase inhibitor | 0.882 | 0.002 |
| 6 | CYP2C12 substrate | 0.887 | 0.015 |
| 7 | Aldehyde oxidase inhibitor | 0.826 | 0.008 |
| 8 | Peroxidase inhibitor | 0.815 | 0.004 |
| 9 | MAP kinase stimulant | 0.804 | 0.003 |
| 10 | Kinase inhibitor | 0.792 | 0.006 |
| 11 | Antineoplastic | 0.795 | 0.012 |
| 12 | Aryl-alcohol dehydrogenase (NADP+) inhibitor | 0.779 | 0.003 |
| 13 | Prostate cancer treatment | 0.769 | 0.004 |
| 14 | 2-Dehydropantoate 2-reductase inhibitor | 0.766 | 0.010 |
| 15 | Antiseborrheic | 0.775 | 0.024 |
| 16 | JAK2 expression inhibitor | 0.762 | 0.011 |
| 17 | Membrane permeability inhibitor | 0.763 | 0.017 |
| 18 | P-benzoquinone reductase (NADPH) inhibitor | 0.746 | 0.004 |
| 19 | Insulysin inhibitor | 0.743 | 0.005 |
| 20 | Alkane 1-monooxygenase inhibitor | 0.740 | 0.012 |
| 21 | Mucomembranous protector | 0.755 | 0.033 |
| 22 | TP53 expression enhancer | 0.736 | 0.019 |
| 23 | Feruloyl esterase inhibitor | 0.733 | 0.018 |
| 24 | Ubiquinol-cytochrome-c reductase inhibitor | 0.757 | 0.048 |
| 25 | Antimutagenic | 0.709 | 0.006 |
| 26 | NADPH-ferrihemoprotein reductase inhibitor | 0.707 | 0.007 |
| 27 | Histidine kinase inhibitor | 0.708 | 0.008 |

**Supplementary table 6c**. Predicted biological activities of Grayanoside A.

| **Serial no** | **Biological activity** | **Grayanoside A** | |
| --- | --- | --- | --- |
|  |  | **Pa** | **Pi** |
| 1 | Free radical scavenger | 0.968 | 0.001 |
| 2 | Membrane integrity agonist | 0.969 | 0.002 |
| 3 | Monophenol monooxygenase inhibitor | 0.959 | 0.002 |
| 4 | Membrane permeability inhibitor | 0.954 | 0.002 |
| 5 | Antiprotozoal | 0.938 | 0.002 |
| 6 | Chemopreventive | 0.923 | 0.002 |
| 7 | Benzoate-CoA ligase inhibitor | 0.909 | 0.004 |
| 8 | Cholesterol antagonist | 0.895 | 0.003 |
| 9 | Anticarcinogenic | 0.894 | 0.003 |
| 10 | Vasoprotector | 0.877 | 0.003 |
| 11 | CDP-glycerol glycerophosphotransferase inhibitor | 0.865 | 0.016 |
| 12 | Hepatoprotectant | 0.846 | 0.003 |
| 13 | 4-Coumarate-CoA ligase inhibitor | 0.838 | 0.001 |
| 14 | Alkenylglycerophosphocholine hydrolase inhibitor | 0.813 | 0.016 |
| 15 | Proliferative diseases treatment | 0.790 | 0.004 |
| 16 | Caspase 3 stimulant | 0.768 | 0.007 |
| 17 | Radioprotector | 0.766 | 0.006 |
| 18 | Lipid peroxidase inhibitor | 0.761 | 0.004 |
| 19 | GABA aminotransferase inhibitor | 0.754 | 0.004 |
| 20 | Antihypoxic | 0.754 | 0.005 |
| 21 | Mycothiol-S-conjugate amidase inhibitor | 0.749 | 0.004 |
| 22 | UDP-glucuronosyltransferase substrate | 0.747 | 0.011 |
| 23 | Lactase inhibitor | 0.729 | 0.005 |
| 24 | Fibrinolytic | 0.728 | 0.014 |
| 25 | Vasodilator | 0.720 | 0.008 |
| 26 | Skin whitener | 0.714 | 0.002 |
| 27 | Anaphylatoxin receptor antagonist | 0.724 | 0.020 |
| 28 | Antineoplastic | 0.713 | 0.024 |
| 29 | Sugar-phosphatase inhibitor | 0.718 | 0.030 |
| 30 | CYP2H substrate | 0.713 | 0.033 |
